# Why is the lawn buzzing?

**DOI:** 10.3897/BDJ.2.e1101

**Published:** 2014-04-24

**Authors:** Timothy Mark Jones

**Affiliations:** † Louisiana State University, Baton Rouge, United States of America

**Keywords:** Honey bees, *Apis mellifera*, centipede grass, turf grass, *Eremochloa ophiuroides*, pollination, *
Poaceae
*, anemophilous, entomophilous

## Abstract

Graminoids, including grasses, are frequently described in the botanical literature as being wind-pollinated. This paper offers visual evidence for insect pollination of a grass. Three of the bees involved were found to have 100% grass pollen in their pollen sacs. In reviewing the literature for this paper, it was evident that those working with bees are well aware that these insects often pollinate graminoids. It is not clear why this information has not been incorporated into the botanical literature.

## Introduction

### Two taxa interacting half a world away

Centipede grass (*Eremochloa ophiuroides*) (Figs 5, 7) is a turf grass that originated in Asia, that is now found world-wide (Thieret 2003). The popularity of centipede grass is no doubt the result of its small leaves, prostrate growth habit, and ground-hugging mats of long stolons. When blooming, this low-growing grass will produce an inconspicuous inflorescence that is hard to see from a distance. This minimal amount of maintenance and visibility, has inspired another common name, lazy man’s grass, as it requires only an occasional mow to keep in check.

*Apis mellifera*, or honey bees, were introduced to North America by European settlers in the 1700's and are not native to the North American continent. They are now best described as being ubiquitous worldwide. Agricultural necessity has fostered this expansion as bees help to pollinate crops. Their evolutionary and phylogenetic origins appear to be multiple radiations out of Africa, with later expansions to Asia and Europe (Whitfield et al. 2006).

## Material and methods

**Figure 2.**
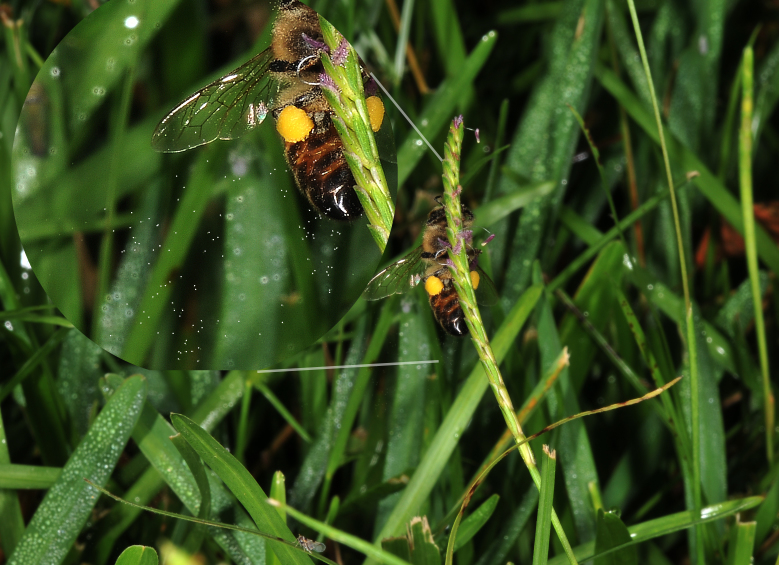
Honey bee moving pollen up the culm, while also spreading pollen through biotic winds; here with pollen visible at left and below bee

**Figure 3.**
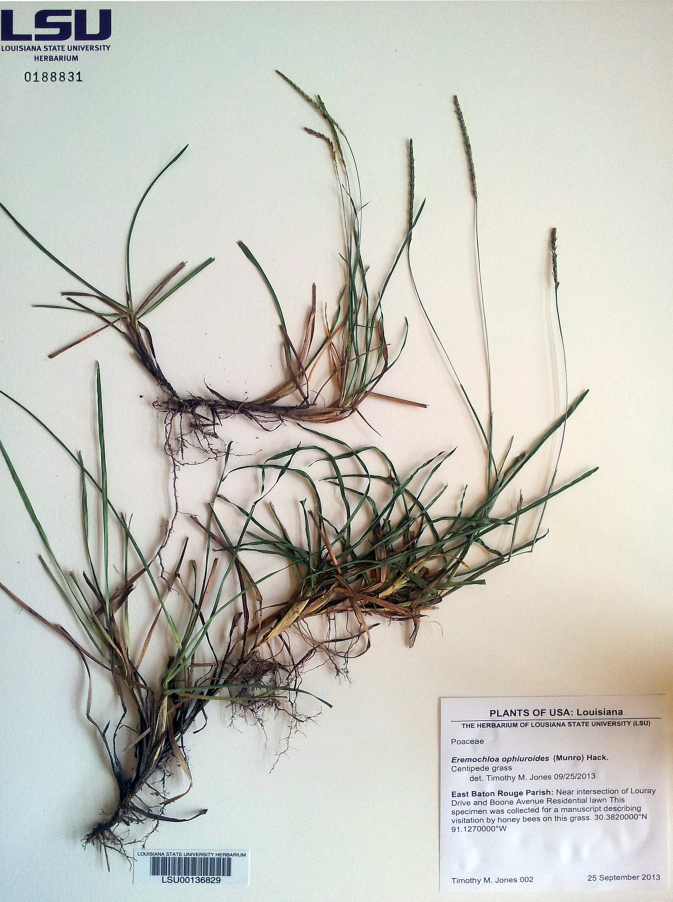
*Eremochloa ophiuroides* specimen collected at observation locality

Observations were made by sitting/walking in a residential lawn in Baton Rouge, Louisiana, USA, during late September 2013 through early October 2013 (Fig. 1). Occurrences of honey bees visiting centipede grass were documented with both video and still imagery. All observations were between 10:00 AM and 2:00 PM, consisting of video, and one session of macro-photography (Fig. 2). The grass was sampled for identification and a specimen sheet was created at Louisiana State University Herbarium (Fig. 3). Other plants at anthesis that could provide potential forage for bees were also noted. (Table 1).

**Figure 4.**
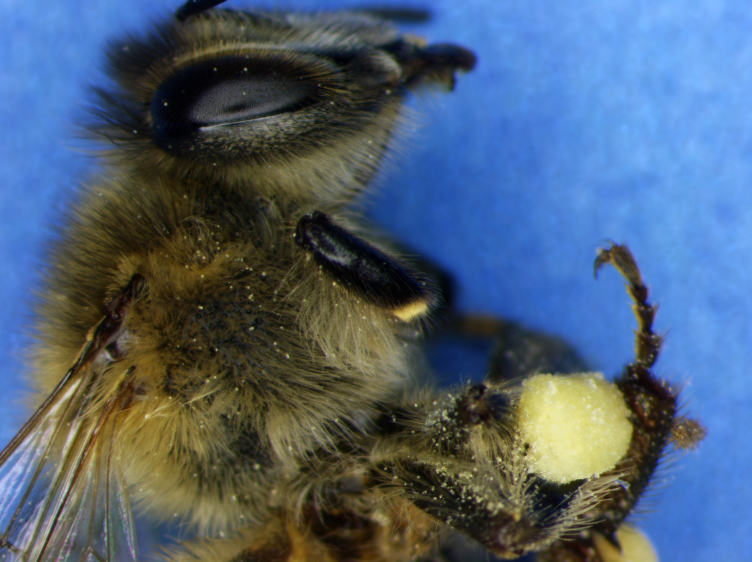
Collected honey bee with body dusted in pollen and packed pollen baskets or corbiculae

**Figure 5.**
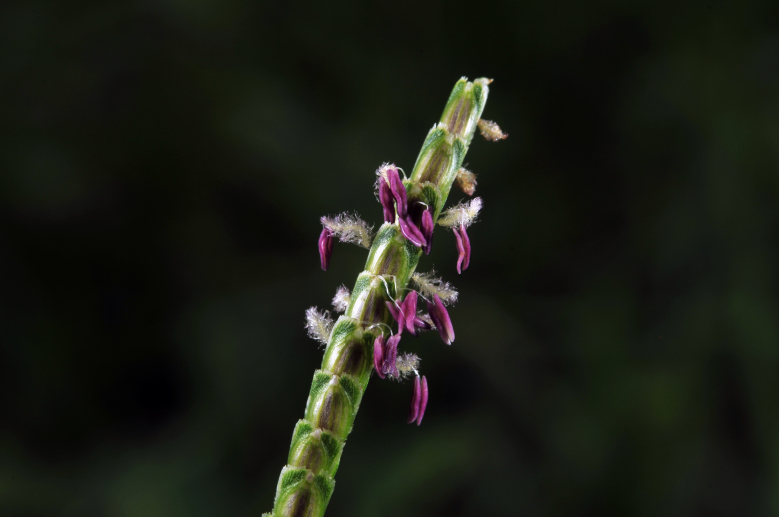
Centipede grass at anthesis

**Figure 6.**
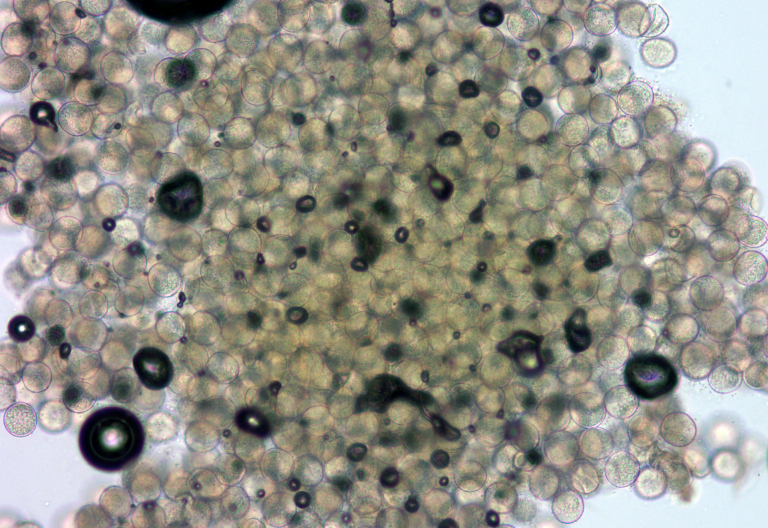
Pollen sample at 20× from one bee corbicula demonstrating homogeneity. Image by: Sophie Warny

A total of three bees were sampled for taxonomic identification and examined by curators at the Louisiana State University Arthropod Museum (Fig. 4). One honey bee pollen basket was then sampled for homogeneity at Louisiana State University Center for Excellence in Palynology (Fig. 6). Three honey bee corbiculae pollen contents were then sent to and processed by at Washington State University via acetylosis. Pollen identification was later performed by the Palynology consultants at University of Arizona.

Equipment used:

- Galaxy Note I cell phone for video
- Nikon D300 DSLR camera with a 1:1 macro lens for still images
- Olympus Microscope with slaved digital camera for microscopy images

## Data resources

Rainfall prior and post-observations; a wet summer/early fall and not a time of drought stress/starvation (Table 3, Suppl. materials 1, 2, 3, 4, 5, 6)

## Results

The honey bees were exclusively gathering unifloral *Poaceae* pollen (Table 2). Macro-photography revealed that as the bees traveled from inflorescence to inflorescence, they generated biotic winds that moved the *Poaceae* pollen significant distances (Fig. 8).

## Discussion

The graminoids are treated in botanical literature as using the pollination syndrome of anemophily (Niklas 1985), or abiotic wind pollination (c.e.g., Walters and Keil 1996). A reason for this abiotic relationship are that the flowers are small and drab in appearance rather than showy (Knuth 1909). In contrast, though diminutive and lacking petals and sepals, most graminoid inflorescences are quite colorful when blooming, plus present ultraviolet visual cues that are visible to the bees but cannot be seen by humans (Baby et al. 2013). This dichotomy reveals an interesting question: is this just a scale problem for attractiveness? Insects are resourceful feeders, and will take advantage of pollen feeding opportunities that are acceptable and provide visual signatures of readiness for anther dehiscence (Fig. 7).

Honey, a well documented economic commodity that is studied and sampled for purity and origins, tells a different story from botanical literature. Melissopalynology, or the study of pollen in honey, describes the collection of graminoid pollen by honey bees as commonplace. This literature is not isolated but found from across the globe, describing collection of pollen from all graminoids: *Poaceae* (Keller et al. 2005), *Cyperaceae*​ (Song et al. 2012), and *Juncaceae* (Huang et al. 2013). Interestingly, additional observations of other non-graminoid anemophilous plants of the *Angiospermae*, are also found to have associations with honey bees; including willows (*Salicaceae*) (Puusepp and Koff 2014, Salonen et al. 2009), oaks (*Fagaceae*) (Stawiarz and Wroblewska 2010, Bryant 2001), and even glassworts (*Sarcocornia*) (Adam et al. 1987).

## Supplementary Material

Supplementary material 1 Climatological Data for Louisiana, June 2013 Data type: PDF
Brief description: Flat file for June 2013 precipitation
File: oo_6602.pdf National Oceanic and Atmospheric Administration

Supplementary material 2 Climatological Data for Louisiana, July 2013 Data type: PDF
Brief description: Flat file for July 2013 precipitation
File: oo_6603.pdf National Oceanic and Atmospheric Administration

Supplementary material 3 Climatological Data for Louisiana, August 2013 Data type: PDF
Brief description: Flat file for August 2013 precipitation
File: oo_6600.pdf National Oceanic and Atmospheric Administration

Supplementary material 4 Climatological Data for Louisiana, September 2013 Data type: PDF
Brief description: Flat file for September 2013 precipitation
File: oo_6599.pdf National Oceanic and Atmospheric Administration

Supplementary material 5 Climatological Data for Louisiana, October 2013 Data type: PDF
Brief description: Flat file for October 2013 precipitation
File: oo_6601.pdf National Oceanic and Atmospheric Administration

Supplementary material 6 Derived wet summer data from NOAA Data type: xls
Brief description: "How-did" converting inches to centimeters across three weather points in Baton Rouge, La., from manual NOAA PDF amalgamation
File: oo_6604.xlsx NOAA and Tim Jones

## Figures and Tables

**Figure 1.** Video observations of multiple bees collecting pollen from centipede grass

**Figure 7a.**
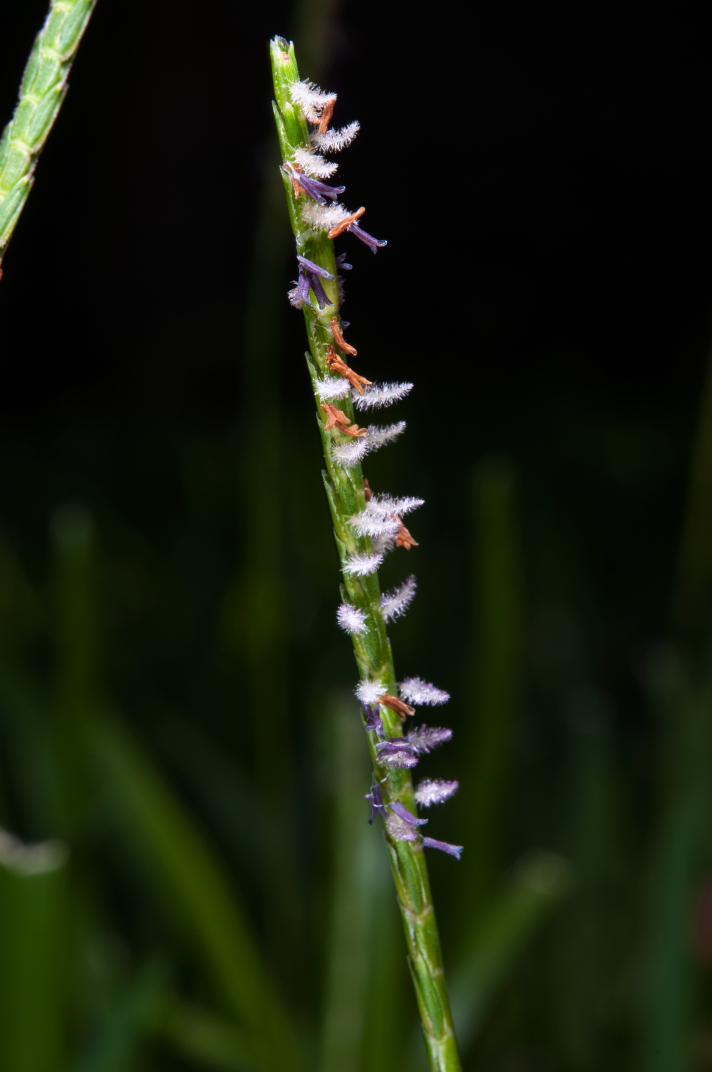
anthers before and after dehiscing

**Figure 7b.**
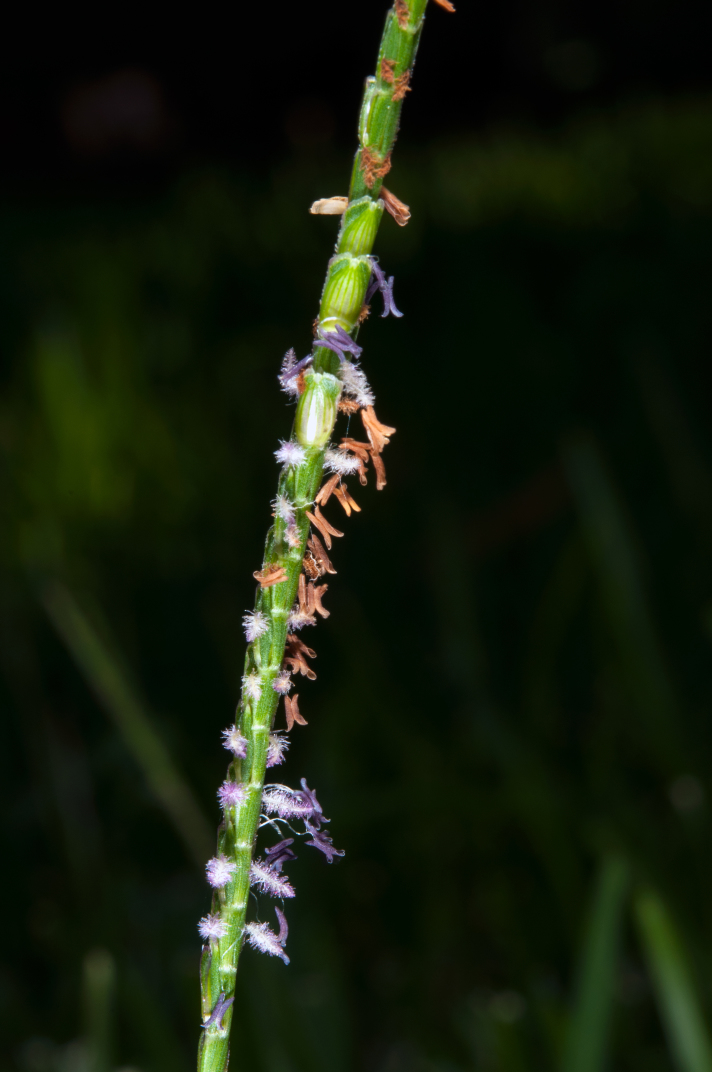
another culm showing same anther color changes

**Figure 8a.**
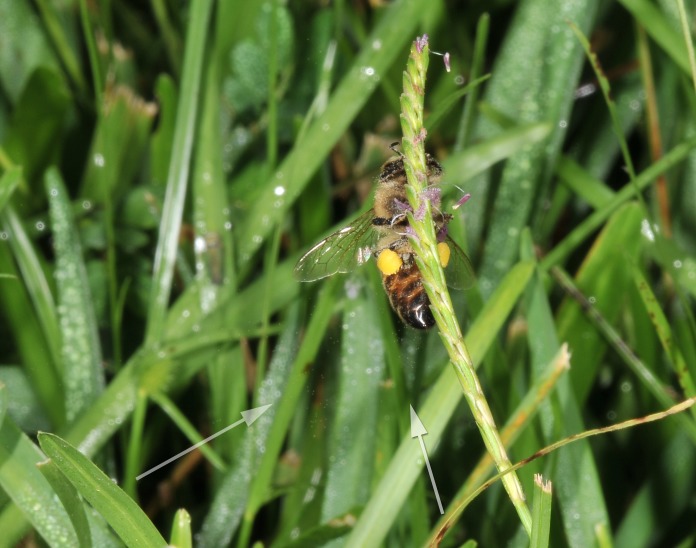
Pollen dispersal caused by bee

**Figure 8b.**
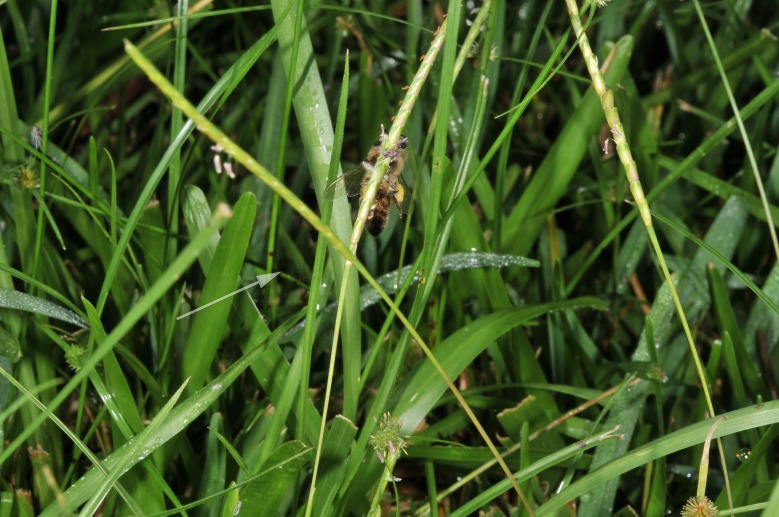
Spreading of pollen as result of bee

**Figure 8c.**
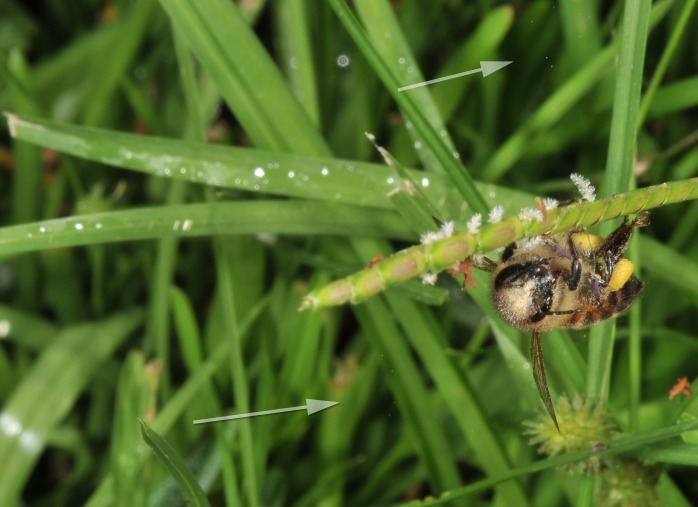
Pollen both above and below bee

**Figure 8d.**
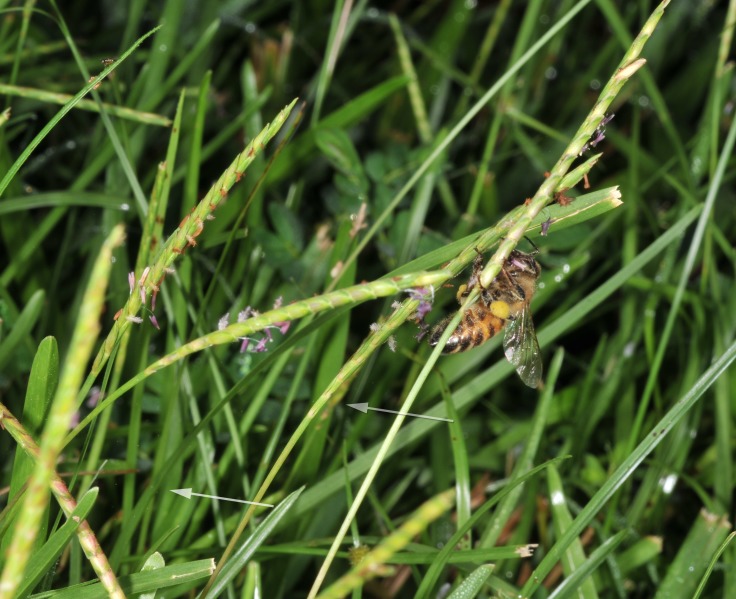
Distribution and distance of pollen travel from bee

**Table 1.** Other plants at anthesis in association with *Eremochloa ophiuroides*.

Species	Family
*Duchesnea indica* (Andrews) Focke	* Rosaceae *
*Mikania scandens* B.L.Rob.	* Asteraceae *
*Ligustrum sinense* Lour.	* Oleaceae *
*Lablab purpureus* (l.) Sweet	* Fabaceae *
*Kyllinga brevifolia* Rottb.	* Cyperaceae *
*Oplismenus hirtellus* (L.) P. Beauv.	* Poaceae *
*Digitaria ciliaris* (Retz.) Koeler	* Poaceae *
*Ruellia simplex* C.Wright	* Acanthaceae *
*Brugmansia* sp. Pers.	* Solanaceae *

**Table 2.** Pollen analysis after acetolysis from bee corbiculae

Bees – using one corbicula	Sampled pollen grains	Percentage *Poaceae* pollen
1	252	100%
2	266	100%
3	270	100%

**Table 3.** Precipitation amounts for summer and early fall 2013, in Baton Rouge, Louisiana, USA. http://www.ncdc.noaa.gov/

**Month (2013)**	**Precipitation (cm)**
June	10.4
July	11.9
August	10.9
September	19.3
October	7.9
**Total**	**60.4**
