# Supplementary material for: Why is the lawn buzzing?
Source: Biodivers Data J. 2014 Apr 24;(2):e1101. doi: 10.3897/BDJ.2.e1101 (PMC4040422; doi:10.3897/BDJ.2.e1101)
Supplement: Supplementary material 1 — Climatological Data for Louisiana, June 2013 [file biodiversity_data_journal-2-e1101-s001.pdf]

CLIMATOLOGICAL DATA

LOUISIANA

JUNE 2013

VOLUME 118 NUMBER 06
ISSN 0145-0409
GHCND Ver: 3.12-upd-2013102505

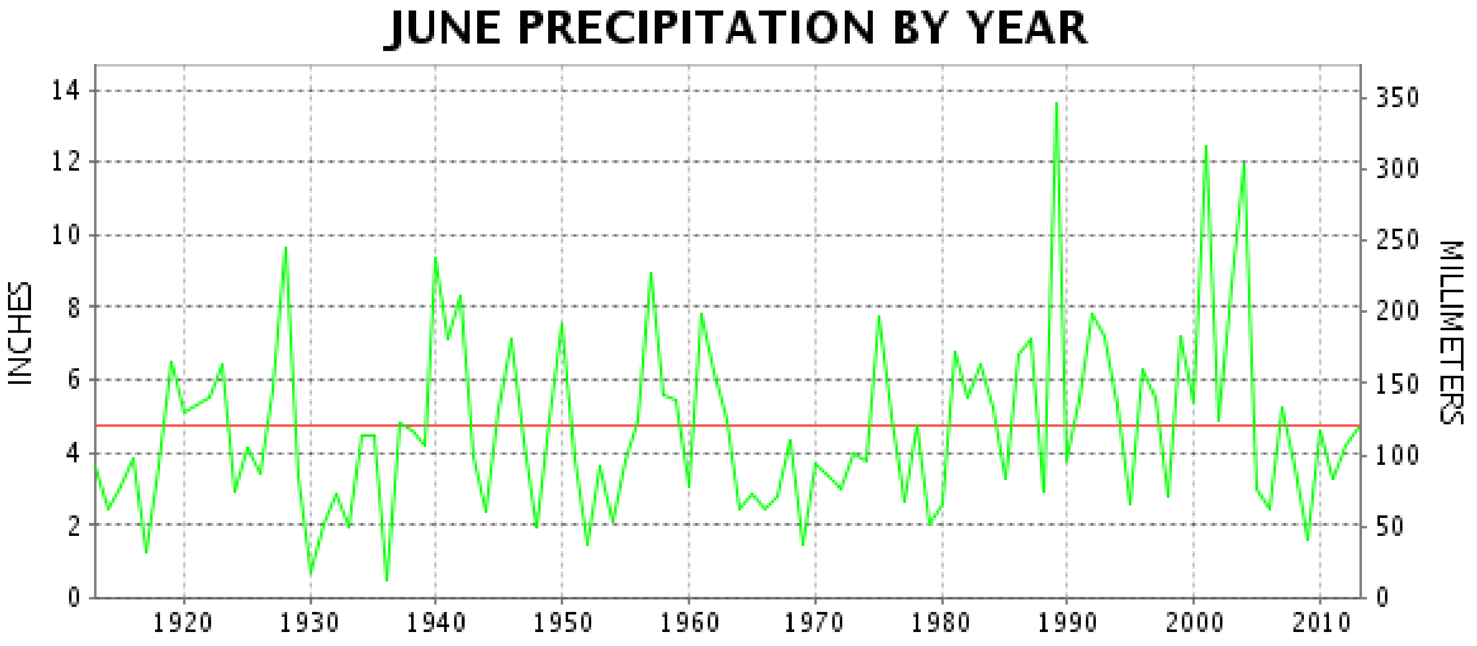

TEMPERATURE AND PRECIPITATION EXTREMES

**LOUISIANA**			
HIGHEST TEMPERATURE	102	JUNE 30+	MOORINGSPORT 1 N
LOWEST TEMPERATURE	57	JUNE 08+	2 STATIONS
GREATEST TOTAL PRECIPITATION	13.16		LAKE CHARLES 7 NW
LEAST TOTAL PRECIPITATION	1.19		GONZALES
GREATEST 1 DAY PRECIPITATION	5.15	JUNE 10	MORGAN CITY

"I certify that this is an official publication of the National Oceanic and Atmospheric Administration (NOAA) It is compiled using information from weather observing sites supervised by NOAA/National Weather Service and received at the National Climatic Data Center(NCDC), Asheville, North Carolina 28801."

Director
National Climatic Data Center

noaa

National
Oceanic and
Atmospheric Administration

National
Environmental Satellite, Data
and Information Service

National
Climatic Data Center
Asheville, North Carolina

MONTHLY STATION AND DIVISION SUMMARY

STATION	TEMPERATURE (°F)														PRECIPITATION (IN)									
											NO. OF DAYS								ICE PELLETS, SNOW			NO. OF DAYS		
											MAX		MIN											
	AVERAGE MAXIMUM	AVERAGE MINIMUM	AVERAGE	DEPARTURE FROM NORMAL	HIGHEST	DATE	LOWEST	DATE	HEATING DEG. DAYS	COOLING DEG. DAYS	>=90	<=32	<=32	<=0	TOTAL	DEPARTURE FROM NORMAL	GREATEST 24 HOURS	DATE	TOTAL	MAX DEPTH ON GROUND	DATE	.10 OR MORE	.50 OR MORE	1.00 OR MORE
**LOUISIANA**																								
**NORTHWEST 01**																								
BENTON 5E	88.7	70.7	79.7	0.3	97	29	61	08	0	447	18	0	0	0	4.04	-1.37	1.80	19	0.0	0		6	2	1
HOSSTON															3.52		1.77	18	0.0			5	3	1
JAMESTOWN															A 6.17		1.86	18	0.0			4	3	2
KEITHVILLE															6.40		2.73	19	0.0	0		5	3	3
KORAN															2.49		1.79	19	0.0			4	1	1
LOGANSPORT															M 6.00		2.55	07	0.0	0		4	3	2
MANSFIELD 7 NW	89.7M	69.8M	79.7M		98	29	61	08+	0	449E	19	0	0	0	M 5.83		1.81	07	0.0			6	4	3
MINDEN	89.4	69.9	79.6	0.8	97	29	61	09	0	446	19	0	0	0	10.78	5.79	4.85	18	0.0	0		5	4	3
MOORINGSPORT 1 N	90.0M	70.7M	80.4M	0.8	102	30+	61	09+	0	469E	19	0	0	0	M 3.66	-1.38	1.90	19	M 0.0	0		4	3	1
RED RIVER RSCH STN	89.9M	71.2M	80.5M	0.4	98	29	63	08	0	474E	18	0	0	0	M 5.36	0.48	1.79	19	0.0			4	3	3
SHREVEPORT DWTN															A 5.57		2.27	19	0.0	0		2	1	1
SHREVEPORT DWTN AP	89.9	71.7	80.8	0.8	98	28	62	08	0	481	19	0	0	0	6.23	1.26	2.84	18	0.0			6	3	2
SHREVEPORT AP	91.1	71.6	81.4	1.6	100	28	63	08	0	498	21	0	0	0	7.75	2.35	3.58	06	0.0	0		6	2	2
SHREVEPORT STHRN HILLS	91.1	69.5	80.3	0.9	100	29	62	08	0	463	22	0	0	0	7.52	2.53	3.90	19	0.0	0		5	3	2
SHREVEPORT WFO	89.7	72.0	80.8	1.3	95	13	65	08	0	484	22	0	0	0	7.98	3.06	3.95	06	0.0	0		6	2	2
SPRINGHILL															4.23		1.21	19	0.0			5	4	3
VIVIAN															M				0.0					
**--DIVISIONAL DATA------->**			80.4	1.6B											6.06	1.17B								
**NORTH CENTRAL 02**																								
ARCADIA															6.68		2.25	02	0.0	0		5	3	3
BIENVILLE 3 NE	90.1	70.4	80.3	1.6	95	28	61	08	0	465	19	0	0	0	6.25	1.32	3.40	18	0.0			5	3	3
CALHOUN RSCH STN	90.0	67.0	78.5	0.5	95	13	57	08	0	409	20	0	0	0	9.90	5.24	4.83	02	0.0			7	5	3
COLUMBIA LOCK	90.3M	69.6M	79.9M	1.2	94	29+	65	15+	0	454E	21	0	0	0	M 4.62	-0.44	1.74	19	M 0.0	0		6	5	1
FARMERVILLE	87.9	70.0	79.0	0.4	94	29	63	08+	0	427	15	0	0	0	5.78	-0.09	3.50	02	0.0	0		5	3	1
HOMER 1N	88.2M	66.9M	77.6M	0.5	97	29	57	09+	0	384E	13	0	0	0	FM 5.38	0.76	2.85	02	0.0			5	3	2
JONESBORO 4 ENE															M				0.0					
MONROE REGIONAL AP	90.4	69.8M	80.1M	-0.5	95	28+	63	08+	0	459E	23	0	0	0	5.45	0.77	1.15	18	0.0	0		8	5	3
MONROE DELTA CC															5.21	0.64	1.93	02	0.0	0		7	5	1
RUSTON LA TECH	91.2M	71.8M	81.5M	3.9	100	02	64	30+	0	503E	20	0	0	0	M 5.60	1.13	4.35	02	M 0.0	0		3	2	1
SAILES FIRE TWR															6.29		2.04	18	0.0	0		5	3	3
WEST MONROE															7.31		3.80	02	0.0			6	5	2
WINNFIELD 3 N	M	M	M								0	0	0	0	M				0.0					
**--DIVISIONAL DATA------->**			79.6	0.8B											6.61	1.86B								
**NORTHEAST 03**																								
BASTROP	87.7	68.8	78.3	0.1	94	29	60	08	0	406	12	0	0	0	6.01	1.55	1.78	18	0.0	0		5	4	3
LAKE PROVIDENCE	89.2	70.6	79.9	0.3	96	28	65	09+	0	454	18	0	0	0	6.04	1.84	1.99	02	0.0	0		7	5	3

MONTHLY STATION AND DIVISION SUMMARY

STATION	TEMPERATURE (°F) AVERAGE MAXIMUM	AVERAGE MINIMUM	AVERAGE	DEPARTURE FROM NORMAL	HIGHEST	DATE	LOWEST	DATE	HEATING DEG. DAYS	COOLING DEG. DAYS	NO. OF DAYS MAX >=90	MAX <=32	MIN <=32	MIN <=0	PRECIPITATION (IN) TOTAL	DEPARTURE FROM NORMAL	GREATEST 24 HOURS	DATE	ICE PELLETS, SNOW TOTAL	MAX DEPTH ON GROUND	DATE	NO. OF DAYS .10 OR MORE	.50 OR MORE	1.00 OR MORE
OAK GROVE															5.12		2.42	02	0.0	0		7	2	1
OAK RIDGE															M 5.76		1.52	18	0.0	0		7	5	2
PIONEER 6 W															M 3.51		1.20	02	0.0	0		6	3	1
RAYVILLE	89.6	71.4	80.5	0.9	95	28	65	09+	0	470	20	0	0	0	3.12	-1.05	1.06	02	0.0	0		6	3	1
ST JOSEPH 3 N	M	70.6M	MM		96	28	61	28	0	500E	16	0	0	0	M 2.74	-1.40	2.59	02	0.0	0		1	1	1
TALLULAH	88.4M	70.4M	79.4M	0.0	93	25+	63	10+	0	438E	12	0	0	0	M 5.73	1.33	3.35	02	0.0	0		5	4	1
TALLULAH VICKSBURG RGN	89.9	69.1	79.5	0.6	94	27+	62	15	0	441	19	0	0	0	5.33	1.34	1.88	02	0.0	0		8	4	2
WINNSBORO 2 SE	90.7	70.7	80.7	0.6	96	28	65	15+	0	480	23	0	0	0	3.96	-0.77	1.02	18	0.0	0		6	4	1
WINNSBORO 5 SSE	M	M	M		95	14	63	15	0	433E	12	0	0	0	M				0.0	0				
**--DIVISIONAL DATA------->**			79.7	0.3B											4.93	0.49B								
**WEST CENTRAL 04**																								
GORUM FIRE TWR															M				0.0					
HODGES GARDENS	90.4M	70.4M	80.4M	0.4	96	30+	64	08+	0	470E	22	0	0	0	M 3.68	-0.99	2.00	07	0.0			4	2	1
LEESVILLE	91.6	68.9	80.3	1.5	100	30	61	30	0	467	22	0	0	0	3.67	-1.42	1.80	07	0.0			6	2	1
LEESVILLE 6 SSW															M				0.0					
MANY 9 WSW															5.66		2.39	07	0.0	0		5	3	2
NATCHITOCHES #2	89.0	70.5	79.8	-0.6	95	28	63	06	0	452	18	0	0	0	4.37	-0.66	1.60	06	0.0	0		6	4	2
TOLEDO BEND LAKE	90.8	70.4	80.6	1.0	98	30	65	11+	0	473	25	0	0	0	3.71	-1.27	1.43	07	0.0	0		5	3	2
ZWOLLE 2 NW															3.44		1.37	07	0.0			4	3	1
**--DIVISIONAL DATA------->**			80.3	1.5B											4.17	-0.50B								
**CENTRAL 05**																								
ALEXANDRIA	91.6	71.6	81.6	0.9	98	30	58	19	0	506	22	0	0	0	3.42	-1.96	1.33	19	0.0			5	3	1
ALEXANDRIA 5 SSE	91.3M	71.1M	81.2M	2.3	98	29	67	10+	0	493E	24	0	0	0	M 4.18	-1.66	2.57	06	M 0.0	0		4	2	1
BEAVER FIRE TWR															3.45		2.39	19	0.0			4	1	1
BOYCE 3 WNW	89.7	72.6	81.1	1.1	96	29	67	10	0	492	22	0	0	0	3.26	-1.75	1.64	18	M 0.0	0		3	2	2
BUNKIE	90.5	72.1	81.3	1.1	97	30	68	10+	0	500	22	0	0	0	2.32	-3.12	1.13	19	0.0			6	1	1
CLAYTON															M				0.0					
ELMER 2 SW															M				0.0					
EUNICE	91.3	72.2	81.8	0.1	96	15	67	09+	0	511	22	0	0	0	3.97	-1.38	0.85	21	0.0			9	3	0
GRAND COTEAU	90.5	72.2	81.4	1.4	94	28+	67	08	0	499	22	0	0	0	2.91	-4.29	1.41	09	0.0			7	1	1
JENA 4 WSW	M	M	M		95	30+	64	30+	0	482E	14	0	0	0	3.96	-1.06	1.52	02	0.0			5	4	2
JONESVILLE LOCKS	89.5	72.3	80.9	0.7	95	30+	68	11+	0	487	17	0	0	0	MA 1.85	-3.04	0.90	02	0.0	0		3	2	0
LSU DEAN LEE RSCH STN	M	M	M		95	17	67	10	0	510E	15	0	0	0	M				0.0					
MARKSVILLE															5.09		2.39	10	0.0			4	3	2
NEW ROADS 5 NE	92.4	72.7	82.5	1.7	97	28	68	02	0	532	23	0	0	0	2.62	-3.01	1.69	29	0.0			3	2	1
OPELOUSAS															3.49		0.87	20	0.0			6	4	0
PORT ALLEN															6.58		1.60	07	0.0			7	5	3
RED RIVER LOCK #1															2.52		0.60	10	0.0	0		7	1	0

MONTHLY STATION AND DIVISION SUMMARY

STATION	TEMPERATURE (°F) AVERAGE MAXIMUM	AVERAGE MINIMUM	AVERAGE	DEPARTURE FROM NORMAL	HIGHEST	DATE	LOWEST	DATE	HEATING DEG. DAYS	COOLING DEG. DAYS	NO. OF DAYS MAX >=90	MAX <=32	MIN <=32	MIN <=0	PRECIPITATION (IN) TOTAL	DEPARTURE FROM NORMAL	GREATEST 24 HOURS	DATE	ICE PELLETS, SNOW TOTAL	MAX DEPTH ON GROUND	DATE	NO. OF DAYS .10 OR MORE	.50 OR MORE	1.00 OR MORE
RED RIVER LOCK # 2															M 4.49		1.71	10	0.0			5	4	1
VILLE PLATTE															M				0.0					
**--DIVISIONAL DATA------->**			81.5	1.7B											3.63	-1.18B								
**EAST CENTRAL 06**																								
ABITA RVR COVINGTON															M 6.18		2.78	29	0.0			10	3	1
ABITA SPRINGS 1 SW															A 5.65		1.39	29	M 0.0			7	2	2
ABITA SPRING FIRE TWR															M				0.0					
ANGIE															M				0.0					
BAKER															A* 3.84		0.56	18	0.0			2	1	0
BATON ROUGE CONCORD															2.92		1.13	03	M 0.0			6	2	1
BATON ROUGE METRO AP	90.7	72.3	81.5	0.4	95	14	68	02	0	502	21	0	0	0	6.47	0.06	2.18	02	0.0	0		7	5	2
BATON ROUGE SHERWOOD															2.82		1.54	20	0.0			6	1	1
BOGALUSA	M	M	M								0	0	0	0	M				0.0					
CLINTON FORESTRY HQ															3.50		1.40	19	0.0			5	3	2
CLINTON 5 SE	88.4	69.8	79.1	1.2	91	28+	64	10+	0	429	11	0	0	0	5.85	-0.06	2.23	10	0.0			6	4	2
COVINGTON 3 NE															M				0.0					
DENHAM SPRINGS															M				0.0					
HAMMOND 5 E	90.4	70.5	80.5	0.9	94	29+	66	30	0	470	18	0	0	0	2.66	-3.19	0.65	03	0.0			8	1	0
KILLIAN															M				0.0					
LIVERPOOL 6W															M 3.68		0.76	10	0.0			10	3	0
LIVINGSTON															3.97		0.99	25	0.0			8	4	0
LSU BEN-HUR FARM	89.8	72.6	81.2	1.0	97	16	69	07+	0	494	18	0	0	0	5.46	-1.57	1.55	23	0.0			9	5	2
MOUNT HERMON 2W															2.26		1.10	19	0.0			5	2	1
NORWOOD															3.52		1.75	19	0.0			4	2	2
OAKNOLIA 2N															4.77		1.61	10	0.0			9	4	1
PINE GROVE FIRE TWR															4.78		1.83	21	0.0			6	4	1
PONCHATOULA 4 SE															M 3.49		1.40	21	0.0			5	4	1
ST FRANCISVILLE															4.44		1.08	02	0.0			12	4	1
SLIDELL	88.3	72.9	80.6	0.4	92	29+	67	03+	0	475	11	0	0	0	6.38	0.81	2.27	07	0.0			8	4	2
SLIDELL AP	90.2	71.8	81.0	1.5	94	14	69	30+	0	486	19	0	0	0	3.48	-2.14	1.37	08	0.0			7	2	1
SUN															3.57		0.80	29	0.0			9	2	0
TALISHEEK															A 2.33		0.67	10	0.0			8	1	0
TICKFAW 3 ENE															M				0.0					
**--DIVISIONAL DATA------->**			80.7	1.3B											4.14	-0.99B								
**SOUTHWEST 07**																								
ABBEVILLE															M 4.55		1.31	07	0.0			10	2	1
BELL CITY 13 SW															7.16		1.97	03	0.0			8	5	3
CROWLEY 2 NE	90.2	73.5	81.9	1.1	96	30	68	07	0	515	19	0	0	0	2.94	-4.09	1.35	07	0.0			5	2	1

LOUISIANA
201306

MONTHLY STATION AND DIVISION SUMMARY

STATION	TEMPERATURE (°F)														PRECIPITATION (IN)									
											NO. OF DAYS								ICE PELLETS, SNOW			NO. OF DAYS		
											MAX		MIN											
	AVERAGE MAXIMUM	AVERAGE MINIMUM	AVERAGE	DEPARTURE FROM NORMAL	HIGHEST	DATE	LOWEST	DATE	HEATING DEG. DAYS	COOLING DEG. DAYS	>=90	<=32	<=32	<=0	TOTAL	DEPARTURE FROM NORMAL	GREATEST 24 HOURS	DATE	TOTAL	MAX DEPTH ON GROUND	DATE	.10 OR MORE	.50 OR MORE	1.00 OR MORE
DE RIDDER	90.7	70.4M	80.6M	0.5	100	30	60	15	0	474E	20	0	0	0	4.00	-2.16	1.59	07	0.0			8	2	2
DRY CREEK 8NW															M				0.0					
HACKBERRY 8 SSW	89.2	75.7	82.5	0.7	97	30	68	07	0	530	13	0	0	0	3.57	-3.46	1.09	03	0.0			8	4	1
JENNINGS	90.6	73.3	82.0	1.5	96	30	68	07	0	517	20	0	0	0	5.35	-1.31	1.65	10	0.0			10	3	2
KAPLAN															4.15		1.13	07	0.0			9	4	1
LAKE ARTHUR 10 SW	91.9	73.9	82.9	1.0	98	29	64	16	0	542	25	0	0	0	4.62	-2.24	2.30	07	0.0			5	3	1
LAKE CHARLES 7 NW															13.16		5.00	03	0.0			10	6	4
LAKE CHARLES 2 N															M 5.34		0.89	03	0.0			7	7	0
LAKE CHARLES PORT															8.30		2.37	02	0.0			9	6	3
LAKE CHARLES AP	91.9	74.0	82.9	1.9	99	29	68	07+	0	545	25	0	0	0	4.06	-2.79	1.13	23	M 0.0	0		8	4	1
LELAND BOWMAN LOCK	89.9	74.6	82.3	1.6	96	15	69	08+	0	526	18	0	0	0	7.33	0.21	2.26	22	M 0.0	0		12	5	3
MOSS BLUFF															5.00		1.57	15	0.0			8	3	2
MOSS BLUFF 2 NNW	92.1	71.6	81.8	2.0	99	29	66	30+	0	513	24	0	0	0	5.75	-1.08	2.28	06	0.0			7	4	3
OAKDALE															3.97		2.80	18	0.0			4	2	1
OBERLIN FIRE TWR	89.4	71.5	80.5	0.7	94	30+	66	09	0	472	17	0	0	0	2.99	-3.64	1.25	09	0.0			5	2	1
OLD TOWN BAY															2.55		0.78	15	0.0			6	2	0
ROCKEFELLER WL REFUGE	87.1	75.0	81.1	1.2	89	30+	68	08+	0	491	0	0	0	0	2.79	-3.44	0.96	22	0.0			5	2	0
SULPHUR															7.86		2.40	08	0.0			11	6	3
VINTON 5W	91.6	71.5	81.6		99	30	65	07	0	506	25	0	0	0	5.63		1.56	07	0.0			8	4	1
**--DIVISIONAL DATA------->**			81.8	1.7B											5.33	-0.53B								
**SOUTH CENTRAL 08**																								
BAYOU SORREL LOCK															3.80		2.40	29	0.0			6	1	1
BUTTE LA ROSE															M				0.0					
CARENCRO															3.04		1.38	07	0.0			5	2	1
CARVILLE 2 SW	90.3	74.3	82.3	1.8	95	26+	71	29+	0	528	21	0	0	0	2.40	-5.13	1.70	29	0.0			4	1	1
DONALDSONVILLE 4 SW	89.6	72.0	80.8	0.7	94	15	69	29+	0	480	19	0	0	0	3.17	-3.98	0.98	21	M 0.0			8	2	0
FRANKLIN 3 NW	88.6	72.9	80.7	0.8	93	14	68	09+	0	481	11	0	0	0	9.39	0.88	4.65	09	0.0			7	4	3
JEANERETTE 5 NW	89.9	72.7	81.3	0.7	95	15	67	07	0	496	19	0	0	0	8.40	0.42	2.32	09	0.0			8	5	4
LAFAYETTE	91.6	73.9	82.7	2.6	96	30+	70	08+	0	542	25	0	0	0	6.50	0.02	2.30	18	0.0			9	4	3
LAFAYETTE FCWOS	91.4	74.2	82.8	1.2	96	29+	69	06	0	541	22	0	0	0	3.16	-3.92	1.93	06	0.0	0		4	1	1
MORGAN CITY	89.1	73.9	81.5	1.2	94	15	70	10	0	504	14	0	0	0	7.70	0.92	5.15	10	0.0			6	3	2
NAPOLEONVILLE															4.57		1.33	09	0.0			6	5	2
NEW IBERIA AP ACADIANA RGNL	92.2	74.9	83.5	2.8	97	14	70	07	0	563	25	0	0	0	6.19	-0.71	1.53	06	0.0			11	4	2
PLAQUEMINE 2 N															3.22		1.76	29	0.0			6	1	1
ST GABRIEL															2.53		0.69	29	0.0			9	1	0
ST MARTINVILLE 3 SW	89.3	72.1	80.7	0.8	94	27+	68	30+	0	480	16	0	0	0	3.30	-5.08	1.08	07	0.0			7	2	1
**--DIVISIONAL DATA------->**			81.8	1.6B											4.81	-1.43B								
**SOUTHEAST 09**																								

LOUISIANA
201306

MONTHLY STATION AND DIVISION SUMMARY

STATION	TEMPERATURE (°F)										NO. OF DAYS				PRECIPITATION (IN)									
											MAX		MIN						ICE PELLETS, SNOW			NO. OF DAYS		
	AVERAGE MAXIMUM	AVERAGE MINIMUM	AVERAGE	DEPARTURE FROM NORMAL	HIGHEST	DATE	LOWEST	DATE	HEATING DEG. DAYS	COOLING DEG. DAYS	>=90	<=32	<=32	<=0	TOTAL	DEPARTURE FROM NORMAL	GREATEST 24 HOURS	DATE	TOTAL	MAX DEPTH ON GROUND	DATE	.10 OR MORE	.50 OR MORE	1.00 OR MORE
BOOTHVILLE ASOS	86.9M	77.0M	81.9M	1.1	90	28+	71	06	0	514E	2	0	0	0	5.59	-0.26	1.82	02	0.0			9	4	2
CONVENT 2S															3.13		1.90	21	0.0			6	1	1
DUTCHTOWN #2															1.28		0.51	20	0.0			4	1	0
GALLIANO	88.7M	75.7M	82.2M	1.5	94	15	70	03	0	524E	11	0	0	0	M 5.93	-1.31	1.40	07	0.0			10	6	3
GONZALES															1.19		0.45	20	0.0			5	0	0
GRAND ISLE															M 2.35		0.47	21	0.0			10	0	0
HOUMA	88.9M	72.2M	80.5M	-0.6	93	29+	68	10+	0	473E	14	0	0	0	M 5.92	-1.40	1.54	03	0.0			8	5	2
LUTCHER															1.95		0.95	23	0.0			4	2	0
MARRERO 9 SSW	M	M	M		95	30+	68	19	0	540E	14	0	0	0	M				0.0					
NEW ORLEANS AP	90.4	75.3	82.8	1.3	95	28+	70	02	0	541	19	0	0	0	4.39	-3.67	1.80	29	0.0	0		8	3	1
NEW ORLEANS AUDUBON	91.8	74.6	83.2	0.8	96	28+	68	02	0	553	26	0	0	0	3.70	-3.44	1.01	20	0.0			7	3	1
NEW ORLEANS ALGIERS															M 7.41		2.21	01	0.0			10	7	2
NEW ORLEANS LKFRNT AP	90.6	77.9	84.3	1.8	95	14	71	02	0	586	19	0	0	0	4.77	-1.83	1.14	29	0.0			7	5	1
TERRYTOWN 3S	90.9	75.3	83.1	1.2	97	15	71	07	0	549	20	0	0	0	4.39	-2.74	1.66	07	M 0.0			10	2	1
THIBODAUX 4 SE	89.8	71.6	80.7	0.5	95	29	67	10+	0	475	18	0	0	0	1.62	-6.97	0.79	09	0.0			3	1	0
**--DIVISIONAL DATA------->**			82.3	1.8B											3.20	-2.66B								

DAILY PRECIPITATION (INCHES)

STATION	TOTAL	01	02	03	04	05	06	07	08	09	10	11	12	13	14	15	16	17	18	19	20	21	22	23	24	25	26	27	28	29	30	31
**LOUISIANA**																																
**NORTHWEST 01**																																
BENTON 5E	4.04		0.42	0.04			0.31	0.42			0.33								0.72	1.80												
HOSSTON	3.52		0.76					0.62			0.14							0.08	1.77	0.15												
JAMESTOWN	A 6.17	*	1.55$_a$				0.57	0.33										0.01	1.86	1.85										T		
KEITHVILLE	6.40		1.50	0.10				1.90			0.04		0.01						0.12	2.73												
KORAN	2.49						0.10	0.30	0.05										0.25	1.79												
LOGANSPORT	M 6.00		2.47	0.02				2.55	0.02		0.81						0.02			0.11	-							-				
MANSFIELD 7 NW	M 5.83	0.01	1.46	0.40	-		-	1.81			0.70								0.35	1.10												
MINDEN	10.78		3.70					0.61			0.10								4.85	1.52												
MOORINGSPORT 1 N	M 3.66		0.07					0.65										0.11	0.93	1.90			-									
RED RIVER RSCH STN	M 5.36		1.74	0.05				1.55			0.08					-			0.14	1.79					0.01				-			
SHREVEPORT DWTN	A 5.57		*	1.13$_a$			*	1.79$_a$			0.35								0.03	2.27												
SHREVEPORT DWTN AP	6.23	0.95	0.12				1.80			0.36							0.16	T	2.84													
SHREVEPORT AP	7.75	0.35	0.21				3.58			0.43								0.19	2.99						T							
SHREVEPORT STHRN HILLS	7.52		0.92	0.16			T	2.40			0.04								0.10	3.90												
SHREVEPORT WFO	7.98	0.18	0.22				3.95			0.27								0.19	3.17													
SPRINGHILL	4.23		1.03								0.30							1.06	0.63	1.21												
VIVIAN	M		-	0.32		-		0.45	-	-	0.31	-	-	-	-	-	-	-	-	-	-	-	-	-	-	-	-	-	-	-	-	
**NORTH CENTRAL 02**																																
ARCADIA	6.68	0.03	2.25				0.26	0.18											2.15	1.81												
BIENVILLE 3 NE	6.25	0.20	1.20				0.30			1.10								0.05	3.40													
CALHOUN RSCH STN	9.90		4.83				1.28	0.05			0.43								0.56	0.54						1.86				0.35		
COLUMBIA LOCK	M 4.62		0.95				0.50	0.02			0.50								0.30	1.74		-				0.01				0.60		
FARMERVILLE	5.78	T	3.50				0.04	0.06										0.71	0.83	0.34										0.30		
HOMER 1N	FM 5.38		2.85	0.12			0.09	0.21								-	-	0.36^{L}	1.60	0.51	-					-						
JONESBORO 4 ENE	M	-	-	-	-	-	-	-	-	-	-	-	-	-	-	-	-	-	-	-	-	-	-	-	-	-	-	-	-	-	-	
MONROE REGIONAL AP	5.45	1.08	0.64			0.23	0.31	T		0.82							T	0.13	1.15	T									1.09			
MONROE DELTA CC	5.21	T	1.93				0.28	0.10			0.81								0.89	0.56										0.64		
RUSTON LA TECH	M 5.60	-	4.35				0.31	-										0.02	0.07	0.78	-		0.02		-					0.05		
SAILES FIRE TWR	6.29		1.82				0.47	0.30											2.04	1.66												
WEST MONROE	7.31		3.80	0.02			1.13				0.55							0.15	0.95	0.70	0.01											
WINNFIELD 3 N	M	-	-	-	-	-	-	-	-	-	-	-	-	-	-	-	-	-	-	-	-	-	-	-	-	-	-	-	-	-	-	
**NORTHEAST 03**																																
BASTROP	6.01		1.42				1.54	0.07										0.32	1.78	0.75				T	0.05					0.08		
LAKE PROVIDENCE	6.04		1.99				1.38	0.10	0.12		T					0.88		1.00												0.57		
OAK GROVE	5.12		2.42				0.95	0.36			T							0.23	0.47	0.31										0.38		
OAK RIDGE	M 5.76		1.40				0.80	0.34			0.27							-	1.52	0.93										0.50		
PIONEER 6 W	M 3.51		1.20				-	-										0.10	0.72	0.32				0.44						0.73		
RAYVILLE	3.12		1.06				0.18	0.01			0.18								0.26	0.64						T				0.79		
ST JOSEPH 3 N	M 2.74		2.59					0.06			0.05								0.04	-										-		
TALLULAH	M 5.73		3.35	0.05			0.83	0.15											0.75	0.60										-		

LOUISIANA
201306

DAILY PRECIPITATION (INCHES)

STATION	TOTAL	01	02	03	04	05	06	07	08	09	10	11	12	13	14	15	16	17	18	19	20	21	22	23	24	25	26	27	28	29	30	31
TALLULAH VICKSBURG RGN	5.33	1.21	1.88			0.26	0.34	0.04	T	0.14							0.50		0.76						0.08				0.12			
WINNSBORO 2 SE	3.96			0.79			0.71	0.16											1.02	0.79						0.49						
WINNSBORO 5 SSE	M		1.30	0.08			1.24	0.03											0.23	-	-	-	-	-	-	-	-	-	-	-	-	
**WEST CENTRAL 04**																																
GORUM FIRE TWR	M	-	-	-	-	-	-	-	-	-	-	-	-	-	-	-	-	-	-	-	-	-	-	-	-	-	-	-	-	-	-	
HODGES GARDENS	M 3.68		0.97				-	2.00			T									0.25	0.07		0.30		0.07	0.02						
LEESVILLE	3.67	0.05	0.89					1.80		0.01	0.16		0.01						0.02	0.17	0.15				0.41							
LEESVILLE 6 SSW	M	-	-	0.78	-	-	-	0.96	-	-	0.10	-	-	-	-	-	-	0.06	-	0.34	0.02	-	-	-	-	-	-	-	-	-	-	
MANY 9 WSW	5.66		0.80	0.01				2.39	0.01		0.24								0.26	1.87	0.07			0.01								
NATCHITOCHES #2	4.37	0.13	0.64	T			1.60	0.60		0.01	0.03								0.13	1.23						T						
TOLEDO BEND LAKE	3.71	0.02	0.77	0.23				1.43			1.04					0.02					0.03					0.17						
ZWOLLE 2 NW	3.44		0.93					1.37			0.06								0.10	0.98												
**CENTRAL 05**																																
ALEXANDRIA	3.42		0.66				0.99	0.27			0.15									1.33			0.02									
ALEXANDRIA 5 SSE	M 4.18		0.47				2.57			0.07									-		0.29				0.77	0.01						
BEAVER FIRE TWR	3.45		0.09				0.05	0.26		0.09	0.30									2.39	0.21						0.06					
BOYCE 3 WNW	3.26		1.10				0.30											0.06	1.64	0.09	0.07											
BUNKIE	2.32		0.21					0.20		0.20	0.39									1.13	0.10					0.09						
CLAYTON	M	-	-	-	-	-	-	-	-	-	-	-	-	-	-	-	-	-	-	-	-	-	-	-	-	-	-	-	-	-	-	
ELMER 2 SW	M	-	-	-	-	-	-	-	-	-	-	-	-	-	-	-	-	-	-	-	-	-	-	-	-	-	-	-	-	-	-	
EUNICE	3.97		0.15					0.42		0.76	0.19	0.01								0.43	0.80	0.85	0.14		0.22							
GRAND COTEAU	2.91		0.12				0.26	0.25		1.41	0.21									0.31											0.35	
JENA 4 WSW	3.96		1.52			0.57						0.32								0.55		1.00	T									
JONESVILLE LOCKS	MA 1.85		0.90				*	0.10a			0.60									-		-				0.25						
LSU DEAN LEE RSCH STN	M	-	-	0.12			-	-	-	-	1.23				-	-	-			0.41	1.11	0.74	-	-		0.76				-	-	
MARKSVILLE	5.09			0.56			0.04			0.35	2.39										1.68										0.07	
NEW ROADS 5 NE	2.62		0.75				0.05			0.10										0.03										1.69		
OPELOUSAS	3.49			0.75				0.27	0.86										0.20	0.01	0.87										0.53	
PORT ALLEN	6.58	T		0.90				1.60		0.21	0.22										0.78			1.52						1.35		
RED RIVER LOCK #1	2.52		0.40							0.20	0.60									0.41	0.45	0.13				0.33						
RED RIVER LOCK # 2	M 4.49		0.06	0.03			0.57	0.82			1.71		-							0.44	0.86		-									
VILLE PLATTE	M	-	-	-	-	-	-	-	-	-	-	-	-	-	-	-	-	-	-	-	-	-	-	-	-	-	-	-	-	-	-	
**EAST CENTRAL 06**																																
ABITA RVR COVINGTON	M 6.18	0.11	0.22	0.30	0.52	0.48	0.19	0.11		0.95	0.03	0.40										0.06	T	-	-	-	-	-		2.78	0.03	
ABITA SPRINGS 1 SW	A 5.65	0.01	*	1.10a	0.20	0.29	0.23	0.09	0.01	1.02	T	0.23								T	0.01	0.27	0.02		*	0.78a				1.39		
ABITA SPRING FIRE TWR	M	-	-	-	-	-	-	-	-	-	-	-	-	-	-	-	-	-	-	-	-	-	-	-	-	-	-	-	-	-	-	
ANGIE	M	-	-	-	-	-	-	-	-	-	-	-	-	-	-	-	-	-	-	-	-	-	-	-	-		-	-	-	-	-	
BAKER	A* 3.84	*	*	*	*	*	1.45a		*	*	1.19a	0.02			*	*	Ta		0.56		0.33		*	*	0.29a					-	-	
BATON ROUGE CONCORD	2.92	0.02	T	1.13				0.65		0.18	0.21										0.42	T								0.31		
BATON ROUGE METRO AP	6.47		2.18				0.43	T	T	0.59	0.04	T					0.01		0.06	0.74				1.40	0.77					0.25		
BATON ROUGE SHERWOOD	2.82	0.03	0.05	0.18				0.37		0.20	0.16									0.03	1.54	T			0.20					0.06		
BOGALUSA	M	-	-	-	-	-	-	-	-	-	-	-	-	-	-	-	-	-	-	-	-	-	-	-	-	-	-	-	-	-	-	

DAILY PRECIPITATION (INCHES)

STATION	TOTAL	01	02	03	04	05	06	07	08	09	10	11	12	13	14	15	16	17	18	19	20	21	22	23	24	25	26	27	28	29	30	31
CLINTON FORESTRY HQ	3.50	0.20	0.55							1.12	0.18									1.40		0.05										
CLINTON 5 SE	5.85			0.95				0.25			2.23							1.45		0.75	0.20									0.02		
COVINGTON 3 NE	M	-	-	-	-	-	-	-	-	-	-	-	-	-	-	-	-	-	-	-	-	-	-	-	-	-	-	-	-	-	-	
DENHAM SPRINGS	M	1.50			0.20	-	0.43	0.60		0.70	0.04	-	-	-	-				-		0.41	2.70			0.62	-	-		-	0.05	-	
HAMMOND 5 E	2.66	T		0.65	0.08	0.01			0.01	0.37	0.40										0.20	0.31		0.41		0.10				0.12		
KILLIAN	M			0.30						0.60								0.60	0.40	-	-	-	-	-	-		-	-	-	-	-	
LIVERPOOL 6W	M 3.68	0.15		0.50	-			0.35		0.30	0.76									0.55	-	0.22		0.10	0.45	0.05					0.25	
LIVINGSTON	3.97	0.50		0.61				0.17		0.21	0.11							0.41			0.01	0.96				0.99						
LSU BEN-HUR FARM	5.46	0.01	T	0.69				0.55		0.14	0.32										0.32	0.63		1.55		0.19				1.05	0.01	
MOUNT HERMON 2W	2.26		0.11								0.25									1.10	0.58	0.06								0.16		
NORWOOD	3.52		1.15								0.39									1.75	0.06					0.17						
OAKNOLIA 2N	4.77	0.72		0.16				0.71		0.43	1.61									0.67	0.24					0.12				0.11		
PINE GROVE FIRE TWR	4.78	0.08	0.40					0.90		0.28	0.68									0.61		1.83										
PONCHATOULA 4 SE	M 3.49	0.02	T	0.57	0.07		T	0.08	-	0.60			-		-			-	-	0.13	0.05	1.40								-	0.57	
ST FRANCISVILLE	4.44	0.13	1.08	0.11				0.51		0.10	0.15	0.33							0.04	0.12	0.58	0.37	0.14		0.01					0.77		
SLIDELL	6.38		0.78	0.10	0.04			2.27	0.01	1.55	0.14	T							0.33			0.82								0.34		
SLIDELL AP	3.48	T	0.06		0.01	0.36	0.19	0.05	1.37	0.11	0.63							0.02		T	0.27	0.07	T	0.31						0.03		
SUN	3.57	0.01	0.07	0.16	0.02		0.12	0.40		0.06									0.36		0.66	0.06				0.48	0.25			0.80	0.12	
TALISHEEK	A 2.33		0.18		0.06	0.20	0.14	0.07		0.17	0.67									0.12	0.01		0.31		0.18	0.02	*	0.08a		*	0.12a	
TICKFAW 3 ENE	M	-	-	-	-	-	-	-	-	-	-	-	-	-	-	-	-	-	-	-	-	-	-	-	-	-	-	-	-	-	-	
**SOUTHWEST 07**																																
ABBEVILLE	M 4.55	-		0.30		0.31	0.03	1.31		0.20	0.24	0.24				0.40	0.25	T			0.02	0.38			0.09	T					0.78	
BELL CITY 13 SW	7.16	0.01		1.97				1.28			1.91	0.01	0.01	0.01	0.01	0.50	0.10	0.01		0.70	0.02				0.19	0.40	0.03					
CROWLEY 2 NE	2.94			0.03				1.35		0.20	0.02					0.80			0.17	0.04	0.07	0.06				0.07				0.13		
DE RIDDER	4.00	0.14	0.38					1.59		0.22	T					1.19	0.03			0.10	T		0.10			0.25						
DRY CREEK 8NW	M	-	-	-	-	-	-	1.13	-	0.09	-	-	-	-	-	0.78	-	-	-	-	-	-	-	-	-	0.58	-	-	-	-	-	
HACKBERRY 8 SSW	3.57	0.20		1.09		0.02		0.77			0.51					0.53				0.14	0.13	0.01				0.17						
JENNINGS	5.35			0.04				1.31		0.07	1.65					0.61	0.04		0.24	0.15	0.18	0.33	0.31	0.11		0.31						
KAPLAN	4.15			0.30				1.13		0.15	0.55					0.90	0.05					0.30	0.10			0.56				0.11		
LAKE ARTHUR 10 SW	4.62			0.06				2.30	0.01		0.74	0.07				0.71				0.03	0.27	0.40			0.02	0.01						
LAKE CHARLES 7 NW	13.16	0.47	2.90	5.00				1.15	0.02	0.28		T				0.83	0.08			0.35	1.43	T	0.15		0.50	T						
LAKE CHARLES 2 N	M 5.34	0.66	-	0.89				0.73		0.03	0.01					0.80				0.76	0.71	0.05			0.68	0.02						
LAKE CHARLES PORT	8.30	0.25	2.37	0.08		0.01	0.01	1.04		0.11	0.01	0.01				0.88	0.61			2.00	0.06	0.20			0.61	0.02			0.01	0.01	0.01	
LAKE CHARLES AP	4.06		0.59				0.88			0.61					0.36			T	0.20	0.12	0.01	T		1.13	0.05	0.11						
LELAND BOWMAN LOCK	7.33			0.13		0.05		1.12		0.10	0.77					0.45					0.61	0.12	2.26		1.00	0.17				0.12	0.43	
MOSS BLUFF	5.00	0.38	0.41	0.32				1.12		0.17	T					1.57			0.05	0.15	0.83	T	T		T							
MOSS BLUFF 2 NNW	5.75	0.02	0.17				2.28	0.02	0.57	0.01					1.02	0.03			0.26	1.10	0.04	0.15		0.08								
OAKDALE	3.97		T							0.51									2.80		0.20	0.46										
OBERLIN FIRE TWR	2.99		0.15					0.43		1.25	0.01									0.07	0.37		0.01		0.69	0.01						
OLD TOWN BAY	2.55	T		0.36				0.65	0.15	0.15						0.78				0.30	0.05	0.05	0.03			0.03						
ROCKEFELLER WL REFUGE	2.79			0.09		0.19		0.72				0.01				0.45						T	0.96	0.31	0.06	T						
SULPHUR	7.86		0.20				1.15	0.01	2.40		0.80				0.20				0.50	0.80	0.20	1.30		0.10	0.20							

LOUISIANA
201306

DAILY PRECIPITATION (INCHES)

STATION	TOTAL	01	02	03	04	05	06	07	08	09	10	11	12	13	14	15	16	17	18	19	20	21	22	23	24	25	26	27	28	29	30	31
		DAY OF MONTH																														
VINTON 5W	5.63	0.02	0.40	0.06				1.56			0.94					0.44	0.29			0.82		0.66		0.44								
**SOUTH CENTRAL 08**																																
BAYOU SORREL LOCK	3.80			0.15				0.15		T	0.04										0.14	0.47	0.45							2.40		
BUTTE LA ROSE	M	-	-	-	-	-	-	0.42	-	0.31	-	-	-	-	-	-	-	-	-	-	-	0.77	-	-	-	-	-	-	-	0.57	-	
CARENCRO	3.04			0.02				1.38								0.30	0.01		0.04		0.38				0.80						0.11	
CARVILLE 2 SW	2.40		0.10				0.05													0.30	0.25									1.70		
DONALDSONVILLE 4 SW	3.17	0.15		0.46			0.08	0.02		0.03	0.11	0.02	0.62							0.01	0.06	0.98			0.03	0.11				0.29	0.20	
FRANKLIN 3 NW	9.39		0.89		0.27		0.31		0.03	4.65	0.02									0.10	1.37	0.02			0.02			0.02		1.69		
JEANERETTE 5 NW	8.40			0.10				2.12		2.32	1.25	0.08									0.05	1.10			0.38					0.27	0.73	
LAFAYETTE	6.50							1.11		0.13	0.01					0.17	1.26		2.30		0.71	0.45			0.21						0.15	
LAFAYETTE FCWOS	3.16		0.07				1.93		0.06	0.01			T		T	0.08		T		0.49	0.06	0.23		T	0.03	0.02				0.18		
MORGAN CITY	7.70			0.20		0.10	0.39			0.03	5.15					0.71						0.08		0.03							1.01	
NAPOLEONVILLE	4.57	T		0.66						1.33							0.60						0.50		1.02					0.46		
NEW IBERIA AP ACADIANA RGNL	6.19		0.22				1.53		0.38	0.23	0.30				0.18	0.60				1.17	0.28	0.85			0.39					0.05	0.01	
PLAQUEMINE 2 N	3.22	T		0.01				0.35		0.22	0.30										0.20	0.30		0.08						1.76		
ST GABRIEL	2.53	0.17	T	0.20				0.11		0.11	0.22									T	0.24	0.44		0.35		T				0.69		
ST MARTINVILLE 3 SW	3.30	0.07		0.07				1.08		0.37		0.89					0.07				0.11	0.30			0.22					0.12		
**SOUTHEAST 09**																																
BOOTHVILLE ASOS	5.59		1.82	0.01	0.01	0.04	0.29			0.57	T				0.21	T	0.74			T	0.42		T				0.13		0.13	0.01	1.21	
CONVENT 2S	3.13		0.23				T	0.40		0.05	0.30											1.90								0.15	0.10	
DUTCHTOWN #2	1.28	0.03		0.02				0.03		0.14	0.15										0.51	0.09	0.02							0.24	0.05	
GALLIANO	M 5.93			1.20	0.03			1.40		0.50	0.20					1.12			0.12			0.10		0.60		0.10		0.56			-	
GONZALES	1.19		0.10			0.04		0.02		0.16	0.20							0.10			0.45									0.08	0.04	
GRAND ISLE	M 2.35			0.08				0.10			0.20					0.10	0.40				0.30	0.47	-		0.10			0.10		0.20	0.30	
HOUMA	M 5.92			1.54						0.58	0.98	0.37	0.38	1.20			0.52					0.01	0.03				-				0.31	
LUTCHER	1.95		0.12				T	T		T	0.18								0.03			0.05		0.95							0.62	
MARRERO 9 SSW	M	0.02	-	0.54	0.04		-	-	-	-	-	-	-			-		0.07	-			0.31	-	-	-		T	0.27		-	0.07	
NEW ORLEANS AP	4.39		0.57		0.09	T	0.24			0.06	0.63					0.40	0.18	0.20			0.17		T	0.03	0.02		T			1.80		
NEW ORLEANS AUDUBON	3.70		0.36				0.64			0.25	0.23						0.22	0.01			1.01			0.06			0.01			0.91		
NEW ORLEANS ALGIERS	M 7.41	2.21		0.23			0.35	0.41	1.05		0.50							0.73				0.73			0.52		0.52	0.08	0.08		-	
NEW ORLEANS LKFRNT AP	4.77	T	0.80		T	0.17	0.08		0.09	0.81	0.67							0.64						T	T	0.37				1.14		
TERRYTOWN 3S	4.39	0.21		0.72			0.16	1.66		0.27	0.23		0.05									0.25		0.29	0.30						0.25	
THIBODAUX 4 SE	1.62			0.36				T		0.79	T		T				T		0.03			0.09			0.03			T		0.03	0.29	

LOUISIANA
201306

DAILY TEMPERATURES (°F)

STATION	OB.TIME	MAX/MIN	01	02	03	04	05	06	07	08	09	10	11	12	13	14	15	16	17	18	19	20	21	22	23	24	25	26	27	28	29	30	31	AVERAGE
**LOUISIANA**																																		
**NORTHWEST 01**																																		
BENTON 5E	08	MAX	90	91	79	83	88	89	77	81	85	84	91	93	91	93	92	91	88	86	76	87	91	92	92	92	92	91	92	94	97	92		88.7
		MIN	76	69	63	64	67	69	66	61	65	68	71	77	75	75	68	70	74	69	67	69	74	71	73	73	76	77	78	77	73	67		70.7
MANSFIELD 7 NW	08	MAX	91	91	82		89		78	83	86	88	90	92	91	93	93	90	90	87	83	87	91	92	91	92	92	91	94	94	98	92		89.7
		MIN	76	68	61		68		65	61	65	66	69	74	74	73	67	70	72	75	68	68	71	69	70	70	74	76	74	74	72	63		69.8
MINDEN	07	MAX	91	92	82	84	89	90	78	83	85	86	91	93	92	95	91	90	88	88	79	88	91	92	91	92	92	91	92	95	97	93		89.4
		MIN	75	70	65	66	67	71	67	62	61	66	67	72	73	75	67	69	73	70	68	68	71	72	72	73	73	74	75	75	74	65		69.9
MOORINGSPORT 1 N	08	MAX	89	92	82	84	91	90	77	83	85	86	93	93	92	96	94	91	92	88	81	86	90		91	91	91	90	93	96	102	102		90.0
		MIN	76	68	62	66	69	73	66	61	61	67	72	74	75	74	71	75	74	71	67	69	75		73	74	75	74	74	74	73	68		70.7
RED RIVER RSCH STN	07	MAX	93	94	84	85	89	92	79	84	85	87	91	92	91	94		93	91	89	82	88	91	92	91	91	92	91	94		98	93		89.9
		MIN	77	70	64	64	69	71	66	63	69	68	72	76	76	77		69	76	71	67	70	74	72	74	74	76	75	76		73	65		71.2
SHREVEPORT DWTN AP	24	MAX	93	82	86	88	90	80	83	85	86	92	93	92	95	94	91	91	88	82	88	91	92	92	92	91	92	93	95	98	93	88		89.9
		MIN	71	66	65	64	70	66	64	62	68	69	75	77	77	77	69	76	75	69	67	72	75	72	75	75	76	77	77	79	74	71		71.7
SHREVEPORT AP	24	MAX	92	83	86	90	92	80	85	86	86	93	94	93	96	96	92	93	89	85	89	92	93	93	93	93	92	94	96	100	95	91		91.1
		MIN	72	66	65	66	71	67	67	63	68	68	73	76	76	76	71	77	74	69	68	73	73	73	74	74	76	76	76	76	77	68		71.6
SHREVEPORT STHRN HILLS	07	MAX	92	93	83	86	90	92	79	84	85	87	93	94	93	95	95	92	89	86	90	93	92	94	94	93	93	93	94	96	100	94		91.1
		MIN	75	69	64	64	64	71	66	62	63	67	67	71	75	75	69	70	74	68	68	69	70	68	68	72	73	75	74	74	74	66		69.5
SHREVEPORT WFO	24	MAX	91	82	84	90	91	82	82	86	86	91	93	91	95	93	91	91	90	82	88	91	91	93	91	91	91	93	93	93	93	91		89.7
		MIN	72	69	66	66	73	68	66	65	68	69	73	76	76	78	72	76	74	69	69	73	74	73	74	74	75	76	76	76	76	68		72.0
**NORTH CENTRAL 02**																																		
BIENVILLE 3 NE	23	MAX	91	82	86	90	92	80	84	87	88	91	94	92	94	94	92	89	89	84	88	92	93	92	92	93	91	93	94	95	93	88		90.1
		MIN	72	68	64	65	72	69	68	61	66	66	72	75	75	77	68	74	74	68	65	69	73	71	73	73	73	74	75	75	73	64		70.4
CALHOUN RSCH STN	08	MAX	88	90	81	87	90	88	90	83	85	90	91	94	95	94	91	94	87	88	86	89	91	91	91	91	92	91	92	94	94	91		90.0
		MIN	72	67	61	62	67	66	60	57	63	65	65	68	77	74	63	62	67	61	68	68	68	69	71	70	70	71	72	73	69	63		67.0
COLUMBIA LOCK	07	MAX	88	91	90	84	89	93	81	82	84	92	92	94	94	94	88	91	91	91	91	87		90	90	93	93	93	94	94	94	90		90.3
		MIN	74	67	66	65	66	67	68	65	65	66	66	74	75	75	65	67	73	71	67	68		71	72	73	73	72	74	75	70	67		69.6
FARMERVILLE	07	MAX	88	90	79	82	87	90	78	82	84	88	91	92	91	93	88	88	87	88	77	86	90	90	91	89	91	90	91	92	94	91		87.9
		MIN	75	67	63	63	67	70	65	63	65	67	72	75	75	75	66	69	70	68	70	70	71	73	73	73	73	74	75	76	71	67		70.0
HOMER 1N	07	MAX	89	91	80	83	87	87	78	82	84	86	92	92	91	94			90	86	78		89	91	89	90		91	92	94	97	91		88.2
		MIN	72	68	64	58	61	70	65	57	57	65	66	72	69	74			63	69	66		67	69	69	69		72	72	74	69	62		66.9
MONROE REGIONAL AP	24	MAX	91	82	86	91	92	81	85	87	91	92	93	93	95	91	92	90	92	84	90	93	93	92	91	92	92	92	95	95	91	88		90.4
		MIN	68	68	65	63	71	70	66	63	66	66	73	76	75	69	65	70	73	70	72	69	73	71	71	73	73	74	75	68		68		69.8
RUSTON LA TECH	08	MAX		100	91	90	92	94		91	90	94	95	94	91	94		90	87	87	82		91	91	89		91	89	91	92	94	91		91.2
		MIN		76	73	68	73	74		69	71	74	76	75	73	75		64	73	69	70		70	70	72		72	73	74	75	71	64		71.8
WINNFIELD 3 N	24	MAX																																M
		MIN																																M
**NORTHEAST 03**																																		
BASTROP	07	MAX	88	88	78	82	88	88	78	81	84	88	91	92	92	93	87	88	88	87	78	86	90	89	90	89	90	90	92	93	94	90		87.7
		MIN	74	65	64	62	64	69	67	60	62	65	67	73	74	74	64	65	71	69	69	70	71	71	71	72	73	73	74	76	70	64		68.8
LAKE PROVIDENCE	07	MAX	88	85	78	84	90	88	82	82	83	91	92	93	93	94	86	88	89	90	91	92	92	90	89	91	91	90	93	96	95	91		89.2
		MIN	73	67	66	66	67	70	69	65	65	68	68	70	71	71	71	71	71	71	72	70	72	74	73	75	73	75	75	75	72	71		70.6

LOUISIANA
201306

DAILY TEMPERATURES (°F)

STATION	OB.TIME	MAX/MIN	01	02	03	04	05	06	07	08	09	10	11	12	13	14	15	16	17	18	19	20	21	22	23	24	25	26	27	28	29	30	31	AVERAGE
			DAY OF MONTH																															
RAYVILLE	07	MAX	90	91	82	84	89	91	81	83	84	91	92	93	93	94	89	90	90	92	83	87	91	91	88	91	93	93	93	95	94	91		89.6
		MIN	75	67	68	67	67	74	70	65	65	67	68	75	77	76	66	66	75	72	71	73	73	74	74	73	74	75	75	78	72	69		71.4
ST JOSEPH 3 N	08	MAX	90	92	92	93	94	95									94	95	83	95		89	93	93		94	92	93	95	96				M
		MIN	66	67	70	69	71	76		65	68	72	72	75	75	77	65	66	75	76		69	72	74		72	73	72	66	61				70.6
TALLULAH	08	MAX	89	88	82	83	89	90	84	82	84	89	90	92	92	93	87		90	90	86	87	89	90	90	91	93	90						88.4
		MIN	74	68	68	68	75	75	70	63	63	63	70	75	75	74	65		72	70	70	70	71	72	72	72	73	72						70.4
TALLULAH VICKSBURG RGN	24	MAX	89	84	85	91	93	85	84	87	89	91	92	92	94	89	91	91	91	85	88	92	92	91	92	91	91	92	94	92	92	88		89.9
		MIN	67	68	67	67	72	68	65	63	66	67	71	72	73	67	62	70	71	71	72	69	70	71	68	72	71	73	74	73	68	64		69.1
WINNSBORO 2 SE	08	MAX	90	90	85	84	91	94	85	83	86	90	92	93	93	95	91	93	92	92	87	89	92	92	91	92	92	92	93	96	93	93		90.7
		MIN	75	69	69	68	69	71	70	65	65	68	69	74	74	75	65	67	74	71	70	69	72	73	72	73	73	73	74	77	70	67		70.7
WINNSBORO 5 SSE	07	MAX	90	91	85	85	91	89	83	83	81	90	92	93	94	95	90	92	92	93														M
		MIN	69	67	68	66	70	68	69	64	65	67	72	73	73	75	63	71	72	69														M
**WEST CENTRAL 04**																																		
HODGES GARDENS	08	MAX	88	90	80	85	92		74	83	85	87	91	93	92	93	94	91	93	91	91	91	91	93	92	93	93	92	95	96	96	96		90.4
		MIN	75	66	64	67	69		65	64	66	66	67	73	73	75	70	71	71	75	75	67	72	72	72	72	72	73	73	72	75	71		70.4
LEESVILLE	08	MAX	89	92	81	87	91	92	78	87	87	88	92	94	93	94	96	93	93	94	92	89	93	94	93	94	91	93	94	98	97	100		91.6
		MIN	75	67	64	65	67	73	66	63	65	63	68	71	72	71	67	74	72	75	69	68	68	67	69	70	69	73	70	72	74	61		68.9
NATCHITOCHES #2	07	MAX	88	91	81	84	88	90	77	83	83	90	91	92	92	93	91	87	92	90	87	85	91	90	88	88	91	91	93	95	94	94		89.0
		MIN	74	68	65	66	71	63	65	66	66	65	71	73	74	76	68	72	74	70	69	71	72	72	73	73	72	74	74	76	75	68		70.5
TOLEDO BEND LAKE	08	MAX	89	90	90	90	90	91	91	83	85	85	90	91	90	92	93	92	91	90	91	85	91	92	92	93	93	91	93	95	96	98		90.8
		MIN	73	68	68	68	69	69	67	67	67	65	65	71	73	73	71	71	73	74	69	70	70	72	72	73	71	72	72	72	76	72		70.4
**CENTRAL 05**																																		
ALEXANDRIA	08	MAX	91	91	85	89	93	94	87	88	88	92	94	93	95	96	97	91	88	94	85	85	93	91	92	91	91	91	94	96	96	98		91.6
		MIN	75	70	70	71	72	68	69	69	69	68	69	75	73	75	73	72	76	76	58	68	71	71	72	74	73	76	75	75	75	69		71.6
ALEXANDRIA 5 SSE	24	MAX	91	91	86	92	93	80	87	88	90	92	93	93	95	94	90	94	95		83	92	92	91	91	91	91	93	95	95	98	91		91.3
		MIN	75	70	69	68	73	71	69	67	67	67	72	73	74	73	68	74	73		70	70	72	70	71	73	71	74	73	73	75	68		71.1
BOYCE 3 WNW	24	MAX	90	80	84	90	90	81	83	87	88	91	93	92	93	92	92	93	91	85	83	90	91	90	90	91	92	93	95	94	96	90		89.7
		MIN	76	69	68	69	75	70	68	69	68	67	73	75	76	77	69	77	75	71	70	71	73	71	74	74	73	76	75	77	78	73		72.6
BUNKIE	08	MAX	90	90	84	86	91	92	87	87	87	89	91	92	92	93	94	91	93	91	89	82	91	91	91	91	92	91	93	94	94	97		90.5
		MIN	75	70	69	69	74	75	70	69	68	68	71	71	74	76	70	74	73	76	72	71	71	71	72	74	73	73	72	75	77	70		72.1
EUNICE	08	MAX	88	91	88	89	93	93	88	86	88	88	90	92	94	94	96	93	92	90	92	85	91	94	93	92	91	92	94	94	94	95		91.3
		MIN	74	71	70	70	75	74	67	68	67	69	73	74	74	76	72	76	73	74	70	70	71	71	72	71	72	75	73	75	77	71		72.2
GRAND COTEAU	17	MAX	89	86	87	92	90	90	85	87	88	89	90	91	92	94	93	91	91	91	89	91	91	91	90	90	90	93	94	94	93	92		90.5
		MIN	74	72	71	71	73	70	68	67	68	70	74	73	73	75	72	75	72	74	72	73	72	72	71	72	72	72	73	76	78	72		72.2
JENA 4 WSW	08	MAX															92	91	92	93	89	88	90	91	91	92	92	92	94	95	94	95		M
		MIN															64	67	72	73	68	69	69	68	69	72	70	73	71	73	73	64		M
JONESVILLE LOCKS	06	MAX	88	88	89	84	88	92	92	84	86	88	88	86	90	91	92	91	90	91	91	85	87	88	92	91	90	90	92	95	92	95		89.5
		MIN	75	72	69	71	71	75	69	70	69	68	68	75	76	76	76	70	69	76	76	71	71	73	74	69	73	73	75	75	75	70		72.3
LSU DEAN LEE RSCH STN	08	MAX			90	88	92					93	90	91	93				95	92	88	94	93			91	90	90	93	94				M
		MIN			69	69	72					67	72	74	75				69	77	71	74	71			70	71	73	73	76				M
NEW ROADS 5 NE	24	MAX	92	82	89	90	94	86	86	92	94	90	95	96	94	95	94	95	95	96	84	89	95	94	96	93	96	96	96	97	89	91		92.4
		MIN	74	68	72	72	74	73	72	70	71	70	75	74	74	76	70	70	73	73	71	72	72	74	72	74	74	74	75	77	72	72		72.7

LOUISIANA
201306

DAILY TEMPERATURES (°F)

STATION	OB.TIME	MAX/MIN	01	02	03	04	05	06	07	08	09	10	11	12	13	14	15	16	17	18	19	20	21	22	23	24	25	26	27	28	29	30	31	AVERAGE
**EAST CENTRAL 06**																																		
BATON ROUGE METRO AP	24	MAX	90	87	89	91	91	88	87	88	89	90	93	93	93	95	94	93	93	91	83	90	93	92	92	88	91	92	93	93	88	90		90.7
		MIN	73	68	72	72	73	69	70	69	70	72	75	74	74	74	70	76	74	72	72	72	71	72	71	72	73	72	75	77	72	72		72.3
BOGALUSA	08	MAX																																M
		MIN																																M
CLINTON 5 SE	08	MAX	88	87	88	87	89	88	86	87	88	88	82	85	91	89	90	91	91	90	89	83	88	90	89	90	86	90	91	91	90	89		88.4
		MIN	73	67	67	70	71	71	70	67	64	64	74	72	72	74	67	72	67	74	69	69	68	70	69	69	70	69	71	74	73	67		69.8
HAMMOND 5 E	08	MAX	86	89	87	90	91	88	89	87	88	88	92	92	93	93	94	94	92	91	92	86	90	93	89	90	89	90	94	93	94	89		90.4
		MIN	72	67	67	72	72	72	68	67	70	72	72	71	70	73	72	71	72	73	70	69	68	70	70	70	70	70	72	74	73	66		70.5
LSU BEN-HUR FARM	08	MAX	86	89	85	90	91	90	89	86	87	88	88	92	92	91	94	97	91	90	90	84	89	93	90	90	88	90	94	93	92	86		89.8
		MIN	72	69	70	72	74	73	69	70	72	71	74	74	72	76	74	77	73	75	73	71	72	72	71	73	72	72	74	77	71	73		72.6
SLIDELL	08	MAX	85	86	86	89	88	87	88	84	87	85	87	90	90	90	92	90	90	88	90	85	87	87	88	89	89	90	91	92	92	88		88.3
		MIN	75	67	67	74	74	71	69	71	70	70	74	74	75	76	72	74	76	74	72	72	70	73	72	75	75	75	77	80	72	71		72.9
SLIDELL AP	24	MAX	88	88	91	89	88	90	85	88	87	89	92	92	91	94	92	93	90	91	86	90	91	90	89	91	91	93	93	93	89	92		90.2
		MIN	71	69	70	73	72	70	69	69	73	71	74	73	74	72	69	72	74	75	72	70	69	71	70	73	72	73	75	77	73	69		71.8
**SOUTHWEST 07**																																		
CROWLEY 2 NE	08	MAX	88	90	83	88	92	93	89	84	87	88	88	91	91	92	94	92	90	87	90	87	88	93	93	91	90	91	93	93	94	96		90.2
		MIN	76	75	72	71	74	74	68	70	69	72	75	74	74	75	74	76	75	75	72	74	73	72	73	75	73	74	74	76	78	73		73.5
DE RIDDER	08	MAX	88	91	81	88	92	93	79	85	87	87	91	93	93	93	91	90	93	88	88	88	94	94	90	94	94	94	94	94	95	100		90.7
		MIN	75	69	69	69	72	76	67	68	67	67	72	74	74	77	60	63	69	69	69	70	70	69	71		71	74	73	74	76	68		70.4
HACKBERRY 8 SSW	08	MAX	86	87	85	89	90	90	87	85	86	88	88	89	89	91	92	90	89	89	89	88	90	89	89	90	90	90	90	91	93	97		89.2
		MIN	77	75	71	73	75	79	68	69	75	74	76	80	77	77	73	77	77	77	75	74	76	77	78	79	75	81	77	77	77	75		75.7
JENNINGS	08	MAX	87	91	86	89	93	93	90	85	87	89	89	92	91	94	95	95	91	90	90	86	88	90	92	90	89	91	92	94	94	96		90.6
		MIN	76	74	71	71	73	75	68	69	70	70	74	75	73	75	73	73	74	74	72	73	74	72	73	76	74	74	75	76	79	73		73.3
LAKE ARTHUR 10 SW	08	MAX	90	85	85	91	94	93	91	88	90	88	90	92	92	96	95	93	92	92	93	88	90	93	91	92	93	93	94	97	98	97		91.9
		MIN	77	71	72	72	74	76	67	68	72	73	74	78	76	71	74	64	78	78	74	76	77	75	75	78	75	77	76	77	74	69		73.9
LAKE CHARLES AP	24	MAX	90	87	91	94	94	89	86	88	90	90	91	92	95	97	93	91	92	92	88	93	91	92	91	91	91	93	94	96	99	95		91.9
		MIN	77	71	72	73	76	68	68	69	72	72	76	75	75	72	75	76	75	74	74	75	74	74	75	76	75	76	74	80	78	73		74.0
LELAND BOWMAN LOCK	08	MAX	86	87	86	87	90	91	91	87	86	86	88	89	91	95	96	94	92	91	92	89	88	90	90	90	86	91	94	90	94	91		89.9
		MIN	78	77	69	73	74	74	69	69	72	72	75	77	76	78	75	76	77	76	75	76	75	73	74	76	77	70	73	79	80	73		74.6
MOSS BLUFF 2 NNW	24	MAX	92	83	89	92	92	88	85	87	91	93	95	93	95	96	94	94	94	92	84	94	92	92	90	94	93	95	96	95	99	93		92.1
		MIN	78	69	70	70	73	68	66	66	68	69	74	72	72	74	73	73	72	73	72	72	72	72	72	73	72	75	71	75	76	66		71.6
OBERLIN FIRE TWR	09	MAX	86	88	83	86	91	91	85	84	86	87	89	91	91	92	94	91	92	90	89	85	89	92	90	90	89	90	92	93	93	94		89.4
		MIN	75	69	69	69	73	74	67	68	66	68	69	73	72	75	71	75	72	74	70	72	72	71	71	72	72	74	71	75	76	69		71.5
ROCKEFELLER WL REFUGE	08	MAX	85	86	82	89	87	89	87	83	84	86	86	88	87	88	89	88	88	87	87	87	88	86	86	87	89	88	89	89	88	89		87.1
		MIN	78	76	71	72	74	76	68	68	72	73	75	76	77	78	72	77	77	79	75	77	75	74	75	76	76	78	78	78	75	75		75.0
VINTON 5W	08	MAX	88	91	84	88	94	92	90	87	90	90	90	92	93	95	95	95	94	92	90	84	91	94	91	91	91	92	94	96	96	99		91.6
		MIN	77	71	68	69	71	72	65	66	69	68	71	71	73	74	72	72	73	74	72	72	71	73	72	72	72	75	72	74	76	69		71.5
**SOUTH CENTRAL 08**																																		
CARVILLE 2 SW	24	MAX	89	90	90	91	90	90	85	88	88	87	92	91	93	95	93	92	92	91	81	88	92	91	91	89	91	95	93	93	87	92		90.3
		MIN	74	74	72	72	74	72	71	72	75	76	73	75	74	78	74	78	78	76	74	74	71	74	74	75	76	74	77	78	71	74		74.3
DONALDSONVILLE 4 SW	08	MAX	87	89	81	90	91	90	89	86	88	89	88	92	91	91	94	92	90	90	90	83	89	91	90	91	90	91	92	93	93	86		89.6

LOUISIANA
201306

DAILY TEMPERATURES (°F)

STATION	OB.TIME	MAX/MIN	01	02	03	04	05	06	07	08	09	10	11	12	13	14	15	16	17	18	19	20	21	22	23	24	25	26	27	28	29	30	31	AVERAGE
		MIN	72	72	70	72	73	72	69	69	72	74	74	72	72	74	73	75	73	73	72	71	71	71	72	72	73	71	72	75	69	70		72.0
FRANKLIN 3 NW	24	MAX	88	80	89	92	89	88	84	86	87	87	86	89	90	93	91	89	89	89	85	87	90	90	89	90	88	91	92	92	87	91		88.6
		MIN	74	69	70	73	75	71	68	69	68	73	73	74	74	76	74	76	74	75	74	70	72	72	74	75	74	74	76	77	71	71		72.9
JEANERETTE 5 NW	08	MAX	89	89	81	89	91	91	89	85	87	89	88	91	91	92	95	91	90	90	91	86	89	90	90	90	90	90	93	94	94	91		89.9
		MIN	73	74	70	72	73	72	67	69	69	68	73	74	74	76	75	75	74	76	73	73	71	71	73	73	73	72	74	76	78	71		72.7
LAFAYETTE	22	MAX	90	91	89	91	93	91	86	87	87	90	91	92	93	94	95	94	92	92	92	89	92	92	91	91	92	92	92	94	96	96		91.6
		MIN	75	75	72	72	72	72	70	70	71	72	72	75	74	74	75	75	75	75	75	75	75	74	74	74	75	75	75	75	80	74		73.9
LAFAYETTE FCWOS	24	MAX	91	85	91	94	93	91	86	89	89	88	92	91	94	96	93	92	91	92	86	91	94	92	89	89	91	94	95	94	96	94		91.4
		MIN	76	75	73	73	74	69	70	70	71	72	75	74	75	79	74	78	75	74	74	74	74	73	73	75	74	74	75	80	77	75		74.2
MORGAN CITY	08	MAX	90	90	85	90	89	90	88	85	89	88	88	91	89	86	94	90	91	90	90	88	83	89	84	87	88	93	93	93	93	89		89.1
		MIN	74	75	73	73	75	75	73	71	74	70	75	74	74	75	75	76	71	73	73	73	72	72	73	75	75	75	76	78	77	71		73.9
NEW IBERIA AP ACADIANA RGNL	24	MAX	91	86	92	95	94	92	87	89	90	89	92	92	94	97	93	92	92	93	88	90	95	94	93	91	92	94	96	95	94	94		92.2
		MIN	76	75	73	74	77	72	70	71	71	73	76	76	75	77	76	78	76	75	75	76	75	74	76	76	75	76	76	78	76	72		74.9
ST MARTINVILLE 3 SW	08	MAX	85	88	82	89	92	91	88	83	87	87	86	90	89	92	94	93	91	90	90	85	87	91	91	90	88	89	94	93	93	92		89.3
		MIN	74	74	70	70	72	71	68	68	70	71	73	73	72	76	73	75	72	74	73	72	71	71	72	74	73	72	73	77	72	68		72.1
**SOUTHEAST 09**																																		
BOOTHVILLE ASOS	24	MAX	85	83	86	86	85	87	84	86	83	86	87	88	88	90	88	88	88			88	88	86	86	89	87	88	89	90	86	89		86.9
		MIN	75	72	78	76	77	71	74	78	74	79	78	78	78	78	78	77	81			72	76	78	78	78	78	78	80	81	81	73		77.0
GALLIANO	08	MAX	86	87	81	86	87	87	89	87	87	85	88	88	90	91	94	90	90	88	91	91	88	89	87	88	89	91	91	93	93			88.7
		MIN	75	76	70	73	77	75	71	74	76	74		73	75	76	78	78	76	78	74	75		75	75	76	76	77	77	82	82			75.7
HOUMA	08	MAX	85	88	82	88	90	87	91	86	87	86	87	88	91	90	90	90	91	90	91	89	91	89	89	88	90		91	93	93	86		88.9
		MIN	71	73	68	72	74	71	69	69	68	68	75	72	72	76	76	75	74	74	71	72	70	71	72	73	72		74	76	74	71		72.2
MARRERO 9 SSW	08	MAX	90		90	91	91								93	92		95	94		94	89	88			91	91		94	93		95		M
		MIN	74		69	73	74								74	76		71	75		68							74		78				M
NEW ORLEANS AP	24	MAX	88	83	89	90	89	90	86	88	88	89	91	91	92	95	91	92	91	92	87	90	92	89	92	91	92	92	95	95	88	93		90.4
		MIN	76	70	73	75	77	72	72	73	75	74	78	75	75	79	80	78	77	75	76	73	74	75	74	75	78	77	77	80	72	73		75.3
NEW ORLEANS AUDUBON	24	MAX	90	85	93	90	90	93	88	90	90	91	94	96	94	96	93	93	93	93	89	90	91	88	91	93	93	93	94	96	91	93		91.8
		MIN	75	68	73	73	78	69	70	72	72	76	76	75	75	78	78	78	75	75	73	72	72	75	75	76	77	78	78	78	74	75		74.6
NEW ORLEANS LKFRNT AP	24	MAX	89	85	92	90	89	91	87	90	87	89	93	92	92	95	93	91	90	92	88	89	92	89	92	93	89	92	94	93	89	92		90.6
		MIN	76	71	76	79	79	74	75	74	75	74	78	80	81	82	80	80	79	79	77	78	80	80	78	77	79	79	82	83	74	78		77.9
TERRYTOWN 3S	07	MAX	87	90	83	90	89	88	93	88	89	87	89	91	93	93	97	92	93	93	93	89	92	92	89	91	91	91	93	94	96	90		90.9
		MIN	75	76	72	75	77	74	71	73	72	76	77	75	76	78	78	78	77	75	74	76	73	72	74	74	77	77	77	78	78	74		75.3
THIBODAUX 4 SE	08	MAX	86	89	81	91	91	89	91	87	88	88	88	91	91	92	93	91	90	90	91	89	91	91	88	88	91	91	91	94	95	86		89.8
		MIN	71	73	69	72	74	71	69	69	67	67	73	71	71	76	74	76	74	72	71	71	69	71	71	72	72	71	73	76	71	70		71.6

LOUISIANA
201306

DAILY SOIL TEMPERATURES

STATION	DEPTH	TIME	01	02	03	04	05	06	07	08	09	10	11	12	13	14	15	16	17	18	19	20	21	22	23	24	25	26	27	28	29	30	31	AVERAGE
**LOUISIANA**																																		
**NORTHWEST 01**																																		
RED RIVER RSCH STN (IN)																																		
BARE GROUND	4	MAX	80	81	80	80	82	81	80	80	80	80	82	82	83	84	-	85	84	84	82	83	83	85	86	86	75 I	85	85	-	85	87		82.8
	4	MIN	77	79	79	78	78	79	79	79	78	79	79	80	81	81	-	81	82	82	80	81	81	82	83	82	73	83	82	-	83	83		80.1
**NORTH CENTRAL 02**																																		
CALHOUN RSCH STN (IN)																																		
SOD	4	MAX	85	87	85	85	86	85	86	82	84	86	88	89	91	90	88	89	85	84	82	87	87	89	85	89	86	88	89	90	89	88		86.8
	4	MIN	75	75	75	75	76	76	71	73	72	70	74	74	74	77	72	76	78	76	76	74	76	78	79	74	78	77	77	77	81	80		75.5
**NORTHEAST 03**																																		
ST JOSEPH 3 N (IN)																																		
BARE GROUND	2	MAX	96	96	95	91	95	103	-	97	95	98	103	102	103	106	107	109	104	102	-	94	101	102	-	106	106	108	-	-	-	-		100.8
	2	MIN	74	74	80	70	76	83	-	78	80	78	77	81	82	81	81	82	86	83	-	77	76	81	-	82	84	82	60	-	-	-		78.7
WINNSBORO 2 SE (IN)																																		
BARE GROUND	2	MAX	86	86	83	82	85	87	83	84	81	84	86	88	88	89	87	86	84	89	82	92	90	87	85	86	87	86	87	89	88	88		86.2
	2	MIN	76	73	73	73	73	76	76	73	72	73	74	76	78	78	74	74	78	76	75	76	77	78	77	78	77	78	78	79	77	75		75.7
WINNSBORO 5 SSE (IN)																																		
BARE GROUND	4	MAX	83	83	81	83	86	89	79	81	80	84	87	87	88	88	89	88	88	84	-	-	-	-	-	-	-	-	-	-	-	-		84.9
	4	MIN	72	75	74	74	74	77	75	73	74	75	74	75	77	78	77	77	79	78	-	-	-	-	-	-	-	-	-	-	-	-		75.4
**CENTRAL 05**																																		
LSU DEAN LEE RSCH STN (IN)																																		
**EAST CENTRAL 06**																																		
CLINTON 5 SE (IN)																																		
HAMMOND 5 E (IN)																																		
**SOUTHWEST 07**																																		
CROWLEY 2 NE (IN)																																		
JENNINGS (IN)																																		

SOILS REFERENCE NOTES

STATION	SOIL TYPE	SOIL COVER	SLOPE	UNITS
RED RIVER RSCH STN	SANDY LOAM	BARE GROUND	00	F
CALHOUN RSCH STN	FINE SANDY LOAM	BARE GROUND	00	F
ST JOSEPH 3 N	SHARKEY CLAY	BARE GROUND	LEVEL	F
WINNSBORO 2 SE	SANDY LOAM	BARE GROUND	0	F
WINNSBORO 5 SSE	SANDY LOAM	BARE GROUND	00	F
LSU DEAN LEE RSCH STN	SANDY	BARE GROUND	00	F
CLINTON 5 SE	FINE SANDY LOAM	BARE GROUND	LEVEL NNW	F
HAMMOND 5 E	SANDY LOAM	BARE GROUND	00	F
CROWLEY 2 NE	SAND	SOD	1 DEG S	F
JENNINGS	SILT CLAY LOAM	SOD	0	F

LOUISIANA
201306

SNOWFALL AND SNOW ON GROUND (INCHES)

STATION		01	02	03	04	05	06	07	08	09	10	11	12	13	14	15	16	17	18	19	20	21	22	23	24	25	26	27	28	29	30	31
**LOUISIANA NORTHWEST 01**																																
BENTON 5E	SNOWFALL																															
	SN ON GND																															
HOSSTON	SNOWFALL																															
JAMESTOWN	SNOWFALL																															
KEITHVILLE	SNOWFALL																															
	SN ON GND																															
KORAN	SNOWFALL																															
LOGANSPORT	SNOWFALL																															
	SN ON GND																															
MANSFIELD 7 NW	SNOWFALL																															
MINDEN	SNOWFALL																															
	SN ON GND					-																										
MOORINGSPORT 1 N	SNOWFALL								-	-						-	-	-	-	-			-							-	-	
	SN ON GND								-	-						-	-	-	-	-			-							-	-	
RED RIVER RSCH STN	SNOWFALL																															
SHREVEPORT DWTN	SNOWFALL																															
	SN ON GND																															
SHREVEPORT DWTN AP	SNOWFALL																															
SHREVEPORT AP	SNOWFALL																															
	SN ON GND																															
SHREVEPORT STHRN HILLS	SNOWFALL																															
	SN ON GND																															
SHREVEPORT WFO	SNOWFALL																															
	SN ON GND																															
SPRINGHILL	SNOWFALL																															
VIVIAN	SNOWFALL																															

(Column group header: DAY OF MONTH, over columns 01–31.)

Snowfall: Includes snow and ice. Values for NWS stations (J index note) are Mid-Mid (LST).
Snow on ground: Includes snow, sleet, ice, and hail. Values for NWS stations (J index note) are observed at 12 UTC (GMT).
Water Equivalent: Given for NWS stations (J index note) only, when snow depth is 2 inches or more, and is measured at 18 UTC (GMT)

LOUISIANA
201306

PAN EVAPORATION AND WIND

STATION		01	02	03	04	05	06	07	08	09	10	11	12	13	14	15	16	17	18	19	20	21	22	23	24	25	26	27	28	29	30	31	TOTAL OR AVERAGE
LOUISIANA																																	
NORTHWEST 01																																	
RED RIVER RSCH STN	WIND	150	72	11	120	39	39	33	17	69	35	33	45	57	42	-	78	52	60	31	38	33	59	30	54	71	70	54	-	51	21		1569E
	EVAP	0.23	0.33	0.15	0.25	0.29	0.17	0.09	0.28	0.15	0.23	0.24	0.38	0.30	0.20	-	0.60	0.23	0.39	0.11	0.30	0.20	0.29	0.23	0.31	0.30	0.30	0.28	-	0.35	0.35		8.07E
	MAX	-	-	-	-	-	-	-	-	-	-	-	-	-	-	-	-	-	-	-	-	-	-	-	-	-	-	-	-	-	-		M
	MIN	-	-	-	-	-	-	-	-	-	-	-	-	-	-	-	-	-	-	-	-	-	-	-	-	-	-	-	-	-	-		M
NORTH CENTRAL 02																																	
CALHOUN RSCH STN	WIND	-	-	-	-	-	-	-	-	-	-	-	-	-	-	-	-	-	-	-	-	-	-	-	-	-	-	-	-	-	-		M
	EVAP	-	-	-	-	-	-	-	-	-	-	-	-	-	-	-	-	-	-	-	-	-	-	-	-	-	-	-	-	-	-		M
	MAX	-	-	-	-	-	-	-	-	-	-	-	-	-	-	-	-	-	-	-	-	-	-	-	-	-	-	-	-	-	-		-
	MIN	-	-	-	-	-	-	-	-	-	-	-	-	-	-	-	-	-	-	-	-	-	-	-	-	-	-	-	-	-	-		M
NORTHEAST 03																																	
ST JOSEPH 3 N	WIND	-	-	-	-	2	11	-	-	-	-	17	14	22	15	-	-	-	47	-	-	1	-	-	-	24	17	31	24	-	-		M
	EVAP	0.00	0.00	0.00	0.12	0.38	0.32	-	0.00	0.00	0.00	0.00	0.00	0.00	0.00	0.00	0.00	0.00	0.00	-	0.00	0.00	0.00	-	0.00	0.00	0.00	0.00	0.27	-	-		1.31E
	MAX	-	-	-	-	-	-	-	-	-	-	-	-	-	-	-	-	-	-	-	-	-	-	-	-	-	-	-	-	-	-		-
	MIN	-	-	-	-	-	-	-	-	-	-	-	-	-	-	-	-	-	-	-	-	-	-	-	-	-	-	-	-	-	-		M
WEST CENTRAL 04																																	
TOLEDO BEND LAKE	WIND	183	128	43	36	40	-	39	32	74	49	53	50	45	23	42	50	51	61	45	39	38	35	53	75	91	91	69	42	42	42		1718E
	EVAP	0.27	0.44	0.15	0.25	0.27	-	0.25	0.23	0.29	0.30	0.24	0.28	0.30	0.29	0.32	0.28	0.33	0.24	0.24	0.22	0.27	0.30	0.32	0.33	0.22	0.33	0.28	0.43	0.32	0.32		8.60E
	MAX	-	-	-	-	-	-	-	-	-	-	-	-	-	-	-	-	-	-	-	-	-	-	-	-	-	-	-	-	-	-		-
	MIN	-	-	-	-	-	-	-	-	-	-	-	-	-	-	-	-	-	-	-	-	-	-	-	-	-	-	-	-	-	-		M
SOUTHWEST 07																																	
JENNINGS	WIND	60	71	33	26	34	38	39	31	45	42	35	40	46	42	52	41	52	70	68	30	35	40	44	49	41	47	58	45	69	57		1380
	EVAP	0.21	0.33	0.09	0.28	0.26	0.31	0.20	0.18	0.25	0.37	0.22	0.27	0.23	0.30	0.38	0.25	0.31	0.31	0.34	0.28	0.22	0.21	0.25	0.24	0.21	0.23	0.31	0.32	0.28	0.23		7.87
	MAX	90	99	90	99	103	103	95	96	95	101	97	101	100	103	105	104	101	98	99	94	97	98	100	98	93	99	101	102	101	102		-
	MIN	74	74	72	72	72	76	67	68	70	70	72	75	75	77	74	73	74	75	73	72	75	72	73	73	72	73	75	75	76	74		73.1

Evaporation: Is measured in hundreths of inches.
Wind: Is measured in miles.
Max and Min: The maximum and minimum tempertures (Fahrenheit) of the water in the evaporation pan.

STATION INDEX

STATION	INDEX NO.	DIVISION	COUNTY	LATITUDE	LONGITUDE	ELEVATION (IN FEET)	OBSERVATION TIME AND TABLES			
							LOCAL STD TIME			
							TEMP	PRECIP	EVAP	SPECIAL SEE (NOTES)
LOUISIANA										
ABBEVILLE	0007	07	VERMILION	29 58	92 7W	10		08		H
ABITA RVR COVINGTON	0012	06	ST. TAMMANY	30 28	90 6W	3		07		H
ABITA SPRING FIRE TWR	0021	06	ST. TAMMANY	30 26	90 3W	30		13		H
ABITA SPRINGS 1 SW	0016	06	ST. TAMMANY	30 28	90 3W	25		07		H
ALEXANDRIA	0098	05	RAPIDES	31 19	92 28W	87	08	08		H
ALEXANDRIA 5 SSE	0103	05	RAPIDES	31 15	92 27W	85	24	24		CH
ANGIE	0238	06	WASHINGTON	30 58	89 49W	130		08		H
ARCADIA	0277	02	BIENVILLE	32 33	92 55W	400		08		H
BAKER	0462	06	EAST BATON ROUGE	30 34	91 10W	70		08		H
BASTROP	0537	03	MOREHOUSE	32 46	92 0W	150	07	07		H
BATON ROUGE CONCORD	0548	06	EAST BATON ROUGE	30 25	91 8W	50		08		H
BATON ROUGE METRO AP R	0549	06	EAST BATON ROUGE	30 32	91 9W	64	24	24		HJ
BATON ROUGE SHERWOOD	0558	06	EAST BATON ROUGE	30 27	91 3W	55		08		H
BAYOU SORREL LOCK	0565	08	IBERVILLE	30 8	91 19W	15		08		H
BEAVER FIRE TWR	0617	05	EVANGELINE	30 48	92 30W	105		13		H
BELL CITY 13 SW	0658	07	CAMERON	29 58	93 5W	4		07		H
BENTON 5E	0718	01	BOSSIER	32 27	93 50W	200	08	08		H
BIENVILLE 3 NE	0800	02	BIENVILLE	32 22	92 57W	307	23	23		H
BOGALUSA	0945	06	WASHINGTON	30 47	89 51W	100	08	08		H
BOOTHVILLE ASOS R	1157	09	PLAQUEMINES	29 20	89 24W	3	24	24		H
BOYCE 3 WNW	1232	05	RAPIDES	31 24	92 43W	110	24	24		H
BUNKIE	1287	05	AVOYELLES	30 58	92 11W	80	08	08		CH
BUTTE LA ROSE	1365	08	ST. MARTIN	30 17	91 41W	5		07		H
CALHOUN RSCH STN	1411	02	OUACHITA	32 31	92 21W	180	08	08	08	GCH
CARENCRO	1535	08	LAFAYETTE	30 19	92 3W	50		07		H
CARVILLE 2 SW	1565	08	IBERVILLE	30 12	91 8W	25	24	24		H
CLAYTON	1866	05	CONCORDIA	31 43	91 32W	73		07		CH
CLINTON 5 SE	1899	06	EAST FELICIANA	30 49	90 58W	200	08	08		GCH
CLINTON FORESTRY HQ	1891	06	EAST FELICIANA	30 51	91 1W	250		13		H
COLUMBIA LOCK	1979	02	CALDWELL	32 10	92 6W	80	07	07		H
CONVENT 2S	2002	09	ST. JAMES	29 60	90 49W	25		08		H
COVINGTON 3 NE	2154	06	ST. TAMMANY	30 31	90 5W	25		07		H
CROWLEY 2 NE	2212	07	ACADIA	30 14	92 21W	25	08	08		G H
DE RIDDER	2367	07	BEAUREGARD	30 51	93 17W	190	08	08		H
DENHAM SPRINGS	2350	06	LIVINGSTON	30 29	90 58W	35		07		H
DONALDSONVILLE 4 SW	2534	08	ASSUMPTION	30 4	91 2W	30	08	08		CH
DRY CREEK 8NW	2641	07	BEAUREGARD	30 44	93 8W	95		07		H
DUTCHTOWN #2	2688	09	ASCENSION	30 15	90 59W	18		07		H
ELMER 2 SW	2812	05	RAPIDES	31 6	92 42W	230		07		H
EUNICE	2981	05	ST. LANDRY	30 29	92 26W	50	08	08		H
FARMERVILLE	3079	02	UNION	32 47	92 24W	180	07	07		H
FRANKLIN 3 NW	3313	08	ST. MARY	29 49	91 33W	12	24	24		H
GALLIANO	3433	09	LAFOURCHE	29 28	90 18W	5	08	08		H
GONZALES	3695	09	ASCENSION	30 12	90 55W	10		07		H
GORUM FIRE TWR	3741	04	NATCHITOCHES	31 26	92 53W	307		13		H
GRAND COTEAU	3800	05	ST. LANDRY	30 25	92 3W	55	17	17		H
GRAND ISLE	3807	09	JEFFERSON	29 14	89 59W	2		07		H
HACKBERRY 8 SSW	3979	07	CAMERON	29 53	93 24W	6	08	08		H
HAMMOND 5 E	4030	06	TANGIPAHOA	30 30	90 23W	35	08	08		GCH
HODGES GARDENS	4288	04	SABINE	31 22	93 23W	420	08	08		H
HOMER 1N	4355	02	CLAIBORNE	32 49	93 4W	215	07	07		H
HOSSTON	4398	01	CADDO	32 27	93 50W	246		08		H
HOUMA	4407	09	TERREBONNE	29 38	90 49W	8	08	08		H
JAMESTOWN	4592	01	BIENVILLE	32 21	93 12W	190		07		H
JEANERETTE 5 NW	4674	08	IBERIA	29 58	91 43W	20	08	08		H
JENA 4 WSW	4696	05	LA SALLE	31 38	92 12W	210	08	08		CH
JENNINGS	4700	07	JEFFERSON DAVIS	30 12	92 40W	25	08	08	08	GCH
JONESBORO 4 ENE	4732	02	JACKSON	32 15	92 39W	330		13		H
JONESVILLE LOCKS	4739	05	CATAHOULA	31 29	91 52W	70	06	06		CH
KAPLAN	4775	07	VERMILION	29 60	92 17W	15		07		H
KEITHVILLE	4816	01	CADDO	32 21	93 52W	200		07		H
KILLIAN	4878	06	LIVINGSTON	30 22	90 33W	10		08		H
KORAN	4931	01	BOSSIER	32 25	93 28W	175		08		H
LAFAYETTE	5021	08	LAFAYETTE	30 13	92 4W	25	22	22		CH
LAFAYETTE FCWOS R	5026	08	LAFAYETTE	30 12	91 59W	38	24	24		H

STATION INDEX

STATION	INDEX NO.	DIVISION	COUNTY	LATITUDE	LONGITUDE	ELEVATION (IN FEET)	OBSERVATION TIME AND TABLES			
							LOCAL STD TIME			
							TEMP	PRECIP	EVAP	SPECIAL SEE (NOTES)
LAKE ARTHUR 10 SW	5065	07	CAMERON	30 0	92 47W	10	08	08		H
LAKE CHARLES 2 N	5074	07	CALCASIEU	30 15	93 13W	5		08		H
LAKE CHARLES 7 NW	5072	07	CALCASIEU	30 18	93 16W	10		08		H
LAKE CHARLES AP R	5078	07	CALCASIEU	30 7	93 14W	9	24	24		HJ
LAKE CHARLES PORT	5076	07	CALCASIEU	30 13	93 15W	5		08		H
LAKE PROVIDENCE	5090	03	EAST CARROLL	32 48	91 10W	100	07	07		H
LEESVILLE	5266	04	VERNON	31 8	93 14W	28	08	08		H
LEESVILLE 6 SSW	5287	04	VERNON	31 3	93 17W	260		08		CH
LELAND BOWMAN LOCK	5296	07	VERMILION	29 47	92 12W	40	08	08		H
LIVERPOOL 6W	5430	06	ST. HELENA	30 56	90 34W	250		08		H
LIVINGSTON	5438	06	LIVINGSTON	30 31	90 45W	43		06		H
LOGANSPORT	5522	01	DE SOTO	31 58	94 0W	190		07		H
LSU BEN-HUR FARM	5620	06	EAST BATON ROUGE	30 22	91 10W	21	08	08		CH
LSU DEAN LEE RSCH STN	5630	05	RAPIDES	31 11	92 25W	70	08	08		G H
LUTCHER	5783	09	ST. JAMES	30 2	90 42W	20		07		H
MANSFIELD 7 NW	5875	01	DE SOTO	32 8	93 45W	255	08	08		CH
MANY 9 WSW	5896	04	SABINE	31 31	93 37W	286		07		H
MARKSVILLE	5920	05	AVOYELLES	31 8	92 4W	85		08		H
MARRERO 9 SSW	5926	09	JEFFERSON	29 47	90 7W	3	08	08		H
MINDEN	6244	01	WEBSTER	32 36	93 18W	185	07	07		CH
MONROE DELTA CC	6314	02	OUACHITA	32 30	92 2W	70		08		CH
MONROE REGIONAL AP R	6303	02	OUACHITA	32 31	92 2W	79	24	24		H
MOORINGSPORT 1 N	6364	01	CADDO	32 42	93 58W	200	08	08		H
MORGAN CITY	6394	08	ST. MARY	29 41	91 11W	5	08	08		CH
MOSS BLUFF	6431	07	CALCASIEU	30 18	93 12W	19		07		H
MOSS BLUFF 2 NNW	6434	07	CALCASIEU	30 20	93 13W	25	24	24		H
MOUNT HERMON 2W	6466	06	WASHINGTON	30 57	90 18W	320		08		H
NAPOLEONVILLE	6561	08	ASSUMPTION	29 56	91 1W	25		07		H
NATCHITOCHES #2	6584	04	NATCHITOCHES	31 49	93 5W	141	07	07		CH
NEW IBERIA AP ACADIANA RGNL R	6657	08	IBERIA	30 2	91 53W	24	24	24		H
NEW ORLEANS ALGIERS	6666	09	ORLEANS	29 57	90 3W	2		08		H
NEW ORLEANS AP R	6660	09	JEFFERSON	29 60	90 15W	4	24	24		HJ
NEW ORLEANS AUDUBON R	6664	09	ORLEANS	29 55	90 8W	20	24	24		H
NEW ORLEANS LKFRNT AP R	6667	09	ORLEANS	30 3	90 2W	9	24	24		H
NEW ROADS 5 NE	6686	05	POINTE COUPEE	30 44	91 22W	45	24	24		H
NORWOOD	6808	06	EAST FELICIANA	30 58	91 6W	102		08		H
OAK GROVE	6866	03	WEST CARROLL	32 52	91 23W	129		08		H
OAK RIDGE	6868	03	MOREHOUSE	32 37	91 47W	82		07		H
OAKDALE	6836	07	ALLEN	30 49	92 40W	110		07		H
OAKNOLIA 2N	6911	06	EAST FELICIANA	30 45	90 60W	150		07		H
OBERLIN FIRE TWR	6938	07	ALLEN	30 36	92 46W	65	09	09		H
OLD TOWN BAY	6968	07	CALCASIEU	30 17	93 9W	12		07		H
OPELOUSAS	6995	05	ST. LANDRY	30 30	92 6W	56		07		H
PINE GROVE FIRE TWR	7304	06	ST. HELENA	30 43	90 45W	190		13		H
PIONEER 6 W	7312	03	WEST CARROLL	32 45	91 32W	88		08		H
PLAQUEMINE 2 N	7366	08	IBERVILLE	30 19	91 15W	20		07		H
PONCHATOULA 4 SE	7425	06	TANGIPAHOA	30 25	90 23W	18		07		H
PORT ALLEN	7448	05	WEST BATON ROUGE	30 27	91 13W	15		07		H
RAYVILLE	7691	03	RICHLAND	32 30	91 45W	89	07	07		CH
RED RIVER LOCK # 2	7732	05	RAPIDES	31 11	92 17W	75		07		H
RED RIVER LOCK #1	7729	05	CATAHOULA	31 15	91 58W	70		07		H
RED RIVER RSCH STN	7738	01	BOSSIER	32 25	93 38W	155	07	07	07	GCH
ROCKEFELLER WL REFUGE	7932	07	CAMERON	29 44	92 49W	4	08	08		H
RUSTON LA TECH	8067	02	LINCOLN	32 32	92 41W	260	08	08		H
SAILES FIRE TWR	8094	02	BIENVILLE	32 22	93 9W	360		13		H
SHREVEPORT AP R	8440	01	CADDO	32 27	93 49W	254	24	24		HJ
SHREVEPORT DWTN	8436	01	CADDO	32 31	93 45W	180		07		H
SHREVEPORT DWTN AP R	8438	01	CADDO	32 33	93 45W	179	24	24		H
SHREVEPORT STHRN HILLS	8444	01	CADDO	32 24	93 47W	200	07	07		CH
SHREVEPORT WFO	8448	01	CADDO	32 27	93 50W	274	24	24		H
SLIDELL	8539	06	ST. TAMMANY	30 16	89 46W	10	08	08		CH
SLIDELL AP R	8543	06	ST. TAMMANY	30 21	89 49W	27	24	24		H
SPRINGHILL	8683	01	WEBSTER	32 60	93 27W	240		07		H
ST FRANCISVILLE	8136	06	WEST FELICIANA	30 47	91 23W	115		07		H
ST GABRIEL	8139	08	IBERVILLE	30 16	91 6W	30		08		H
ST JOSEPH 3 N	8163	03	TENSAS	31 57	91 14W	78	08	08	07	GCH

STATION INDEX

STATION	INDEX NO.	DIVISION	COUNTY	LATITUDE	LONGITUDE	ELEVATION (IN FEET)	OBSERVATION TIME AND TABLES LOCAL STD TIME TEMP	PRECIP	EVAP	SPECIAL SEE (NOTES)
ST MARTINVILLE 3 SW	8181	08	ST. MARTIN	30 5	91 52W	30	08	08		H
SULPHUR	8831	07	CALCASIEU	30 14	93 21W	10		24		H
SUN	8861	06	ST. TAMMANY	30 39	89 55W	75		06		H
TALISHEEK	8906	06	ST. TAMMANY	30 31	89 52W	60		08		H
TALLULAH	8923	03	MADISON	32 24	91 11W	85	08	08		H
TALLULAH VICKSBURG RGN R	8926	03	MADISON	32 21	91 2W	86	24	24		H
TERRYTOWN 3S	8941	09	JEFFERSON	29 55	90 2W	10	07	07		H
THIBODAUX 4 SE	9013	09	LAFOURCHE	29 45	90 46W	15	08	08		CH
TICKFAW 3 ENE	8945	06	TANGIPAHOA	30 36	90 27W	53		24		H
TOLEDO BEND LAKE	9074	04	SABINE	31 12	93 34W	181	08	08	08	H
VILLE PLATTE	9369	05	EVANGELINE	30 42	92 16W	70		07		H
VINTON 5W	9376	07	CALCASIEU	30 12	93 41W	11	08	08		H
VIVIAN	9392	01	CADDO	32 54	93 59W	220		07		H
WEST MONROE	9631	02	OUACHITA	32 28	92 9W	75		07		H
WINNFIELD 3 N R	9803	02	WINN	31 58	92 39W	160	24	24		H
WINNSBORO 2 SE	9804	03	FRANKLIN	32 8	91 43W	74	08	08		G H
WINNSBORO 5 SSE	9806	03	FRANKLIN	32 6	91 42W	80	07	07		GCH
ZWOLLE 2 NW	9980	04	SABINE	31 40	93 40W	209		07		H

REFERENCE NOTES

STATION NAMES: Name of the city, town or locality. Figures and letters following the station names indicate the distance in miles and direction from the post office or town community center.

DIVISIONS: Areas within a state of similar climatological characteristics. Division averages are calculated using data from stations that record temperature and/or precipitation. Station Precipitation totals flagged with an 'F' or 'M' are excluded from the Divisional Average calculations of precipitation. Stations with monthly Temperature averages flagged with an 'F' or 'M' are included in the Divisional Average if there are no more than 9 flagged or missing daily values in the month, else they are excluded from the divisional average for temperature.

NORMALS: The average value of the meteorological element over a time period. Effective 1 January 2012, the averaging period is 1981 to 2010. The normals for National Weather Service localities have been adjusted so as to be representative for the current observation site.

MONTHLY DEGREE DAY TOTALS: One heating (cooling) degree day is accumulated for each whole degree that the daily mean temperature is below (above) 65 degrees Fahrenheit.

PRECIPITATION: Values shown in hundredths of inches are water equivalent totals, i.e., total of liquid and melted frozen precipitation. In the "Monthly Summarized Data" table the total snow and sleet values shown in tenths of inches are unmelted amounts. The max depth on ground values of snow and sleet shown in whole inches are cumulative unmelted amounts. The number of days with .10, .50, 1.00 or more refers to water equivalents.

PRECIPITATION QUALITY CONTROL: The NCDC quality control process may flag precipitation data that are spatially inconsistent, exceed climatological limits, or are inconsistent with prevailing weather patterns.

TEMPERATURE: Original temperature values are given in the "Daily Temperature" table. Summary temperature information (averages, departures, extremes, monthly degree day totals) is based on the values labeled MAX/MIN.

WIND: (As shown in the "Evaporation and Wind" table) the total wind movement in miles over the evaporation pan as determined by an anemometer recorder located 6-8 inches above the pan.

SYMBOLS AND LETTERS USED IN THE STATION INDEX TABLE

C Station is equipped with recording rain gage (R) but values in this bulletin are from a non-recording rain gage unless indicated by an R.
G Observations appear in the "Soil Temperatures" table.
H Observations appear in the "Snowfall and Snow on the Ground" table.
J Station also published as a Local Climatological Data publication.
VAR Observation time varies.
SR Observation time near sunrise.
SS Observation time near sunset.

SYMBOLS AND LETTERS USED IN THE DATA TABLES

(DAILY DATA ARE FOR THE 24 HOURS IMMEDIATELY PRECEDING OBSERVATION TIME.)
BLANK Entries in the "Monthly Summarized Data" table indicate no record.
BLANK Entries in the "Daily Precipitation" and "Snowfall and Snow on the Ground" tables indicate zero.
BLANK Entries in the "Daily Temperature" table indicate a missing record

- No record. Data not recorded or not received in time for publication.
+ Precipitation or temperature extremes occurred on one or more previous dates during the month.
* Rain gage not read. Precipitation is included in the amount following the asterisks. Time distribution may not be known. A * preceding the monthly total indicates precipitation amount is being carried forward to next month's total, and may include amounts from the previous month(s).
a As a subscript, indicates accumulated total.

A Amount of precipitation is the total of observer's entries for the current month. It may include precipitation that occurred during the previous month. Refer to earlier bulletin to determine date of last reading. (Hawaii stations)
B Divisional Departure from normals are computed using 1971-2000 normals.
E Normalized HDD/CDD Calculation. E is appended to the HDD/CDD Calculation when 1-9 individual daily TMAX and/or TMIN values are missing and a Normalized HDD/CDD Calculation is provided. M appears alone if 10 or more daily values are missing.
F Monthly calculation flagged value. F is appended to average and/or total values computed which exclude one or more daily data values that have been flagged by the GHCN-Daily Dataset
M Insufficient or partial data. M is appended to average and/or total values computed with 1-9 daily values missing. M appears alone if 10 or more daily values are missing, (8 or more for wind and evaporation).
N Indicates snow fall or Snowdepth totals are computed with one or more missing days.
R Amounts from recording rain gage.
T Trace. An amount too small to measure.

SEASONAL TABLES: Monthly and seasonal snowfall and heating degree days for the 12 months ending with the June data are published in the July issue of this bulletin. Cooling degree days for the calendar year are published in the "Climatological Data Annual Summary."

Information concerning the history of changes in locations, exposure, etc. of substations is kept on file at the National Climatic Data Center. Historical information of regular National Weather Service Offices may be obtained from the "Local Climatological Data" annual publication. The contents of this publication may be reprinted or otherwise used freely, with proper credit to the National Climatic Data Center. The data are also available digitally.

Effective with the January 2011 Data-Month, COOP Observer Names are no longer included in the Monthly and Annual Climatological Data Publications. This information is not published to ensure the privacy of personal information pursuant to Section 208 of the E-Government Act of 2002 (44 USC 3601).

As of the 2011 Data-Year, Station and Climate Division Maps are no longer being included in the CD Publications. NCDC's Product Development Branch provides updated Station Maps for various data networks via the Historical Observing Metadata Repository: http://www.ncdc.noaa.gov/homr.

The GHCN-Daily Quality Control Flags shown below are displayed as superscripts with the data. For more Information on Global Historical Climatology Network - Daily and flags, see:
http://www.ncdc.noaa.gov/oa/climate/ghcn-daily/
and
Comprehensive Automated Quality Assurance of Daily Surface Observations.
Durre, Imke, Matthew J. Menne, Byron E. Gleason, Tamara G. Houston,
Russell S. Vose, 2010: J. Appl. Meteor. Climatol., 49, 16151633.
doi: 10.1175/2010JAMC2375.1

Blank = Passed All checks	N = failed naught check
D = failed duplicate check	O = failed climatological outlier check
G = failed gap check	R = failed lagged range check
I = failed internal consistency check	S = failed spatial consistency check
K = failed streak/frequent-value check	T = failed temporal consistency check
L = failed check on length of multiday period	W = temperature too warm for snow
M = failed megaconsistency check	X = failed bounds check
	Z = flagged as a result of an official Datzilla investigation

Beginning with the January 2013 CD Publication, monthly mean temperature calculations have changed to the National Data Stewardship Team standard. Monthly maximum and minimum temperatures are not rounded until after the monthly mean temperature is calculated. This is the most accurate outcome, but may be slightly different from the mean derived from rounded monthly maximum and minimum.

Processing Updates and Errata: The 2011 CD Publications were reproduced in May 2013. This update included the addition of late reports and corrections based on additional investigations of reported data issues through NCDCs Datzilla system. In addition, divisional averages for precipitation were recalculated using the method described in DIVISIONS above. Previous editions of the 2011 Publications included all precipitation stations regardless of missing data in the calculation of divisional averages. HDD/CDD values were recalculated to match the legacy method of calculation (truncation of monthly HDD/CDD values instead of rounding).

These and other publications are available from the National Climatic Data Center

Hourly Precipitation Data
This publication contains hourly precipitation amounts obtained from recording rain gages located at National Weather Service, Federal Aviation Administration, and cooperative observer stations. Published data are displayed in inches and tenths or inches and hundredths at local standard time. HPD includes maximum precipitation for nine (9) time periods from 15 minutes to 24 hours, for selected stations.

Climatological Data
Monthly editions contain station daily maximum and minimum temperatures and precipitation. Some Stations provide daily snowfall, snow depth, evaporation, and soil temperature data. Each edition also contains monthly summaries for heating and cooling degree days (65 degree F base). The July issue contains a recap of monthly heating degree days and snow data for the preceding July through June.

The Annual issue contains monthly and annual averages of temperature, precipitation, temperature extremes, freeze data, soil temperatures, evaporation, and a recap of monthly cooling degree days.

Storm Data
Monthly issues contain a chronological listing, by states, of occurrences of storms and unusual weather phenomena. Reports contain information on storm paths, deaths, injuries, and property damage. An "Outstanding storms of the month" section highlights severe weather events with photographs, illustrations, and narratives. The December issue includes annual tornado, lightning, flash flood, and tropical cyclone summaries.

Monthly Climatic Data for the World
This publication contains monthly means for temperature, pressure, precipitation, vapor pressure, and sunshine for approximately 2,000 surface data collection stations worldwide and monthly mean upper air temperatures, dew point depressions, and wind velocities for approximately 500 observing sites.

Local Climatological Data
LCD publications summmarize temperature, relative humidity, precipitation, cloudiness, wind speed and direction observations for several hundred cities in the U.S. and its territories. Each monthly publication also contains 3 hourly weather observations for that month and a hourly summary of precipitation. Annual LCD publications contain a summary of the past calendar year as well as historical averages and extremes.

For Information Call:
(828) 271-4800 Option 2
(828) 271-4010 (TDD)
(828) 271-4876 (Fax)

NOAA\National Climatic Data Center
Attn: User Engagement & Services Branch
151 Patton Avenue
Asheville, NC 28801-5001

Customer Services Number: (828) 271-4800, option 2
TDD : (828) 271-4010
Fax number: (828) 271-4876

NCDC now offers free online access to the ***Climatological Data*** **publication.**
Go to : **www.ncdc.noaa.gov** and choose Most Popular.
