# Supplementary material for: Why is the lawn buzzing?
Source: Biodivers Data J. 2014 Apr 24;(2):e1101. doi: 10.3897/BDJ.2.e1101 (PMC4040422; doi:10.3897/BDJ.2.e1101)
Supplement: Supplementary material 2 — Climatological Data for Louisiana, July 2013 [file biodiversity_data_journal-2-e1101-s002.pdf]

CLIMATOLOGICAL DATA

LOUISIANA

JULY 2013

VOLUME 118 NUMBER 07
ISSN 0145-0409
GHCND Ver: 3.12-upd-2013103005

JULY PRECIPITATION BY YEAR

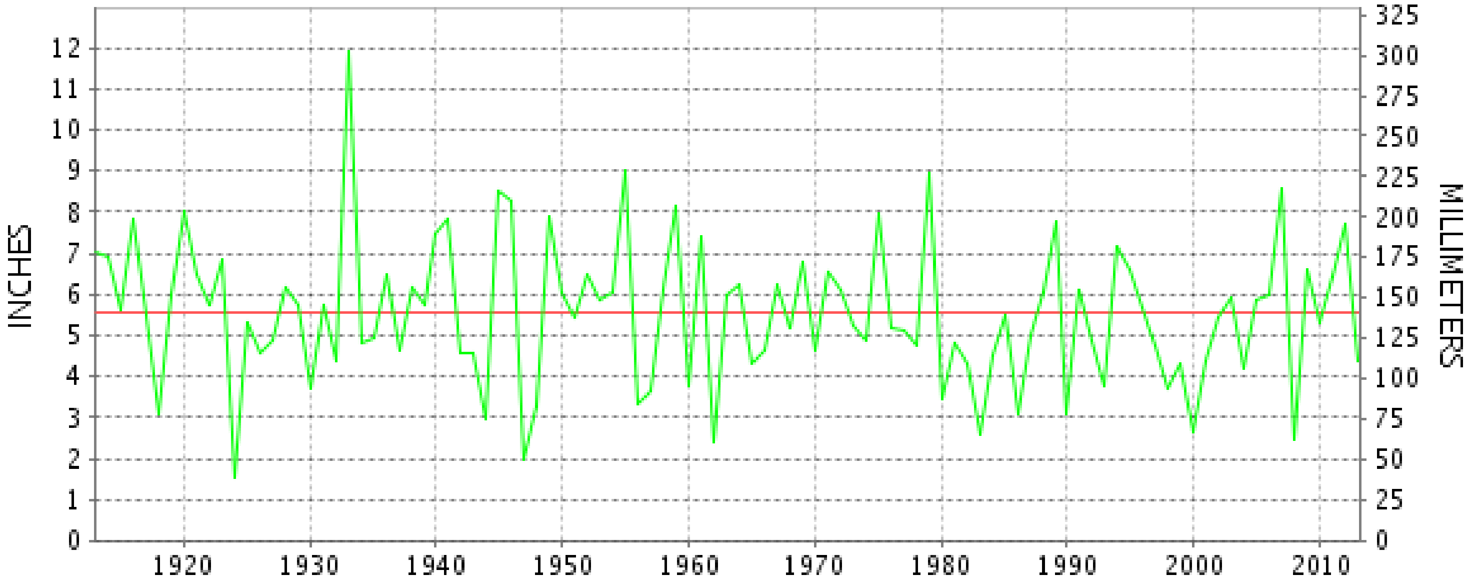

TEMPERATURE AND PRECIPITATION EXTREMES

LOUISIANA			
HIGHEST TEMPERATURE	99	JULY 11	SHREVEPORT STHRN HILLS
LOWEST TEMPERATURE	57	JULY 03	HOMER 1N
GREATEST TOTAL PRECIPITATION	10.69		SLIDELL AP
LEAST TOTAL PRECIPITATION	1.16		ALEXANDRIA 5 SSE
GREATEST 1 DAY PRECIPITATION	3.60	JULY 28	JONESVILLE LOCKS

"I certify that this is an official publication of the National Oceanic and Atmospheric Administration (NOAA) It is compiled using information from weather observing sites supervised by NOAA/National Weather Service and received at the National Climatic Data Center(NCDC), Asheville, North Carolina 28801."

Thomas R. Karl
Director
National Climatic Data Center

noaa National Oceanic and Atmospheric Administration | National Environmental Satellite, Data and Information Service | National Climatic Data Center Asheville, North Carolina

LOUISIANA
201307

MONTHLY STATION AND DIVISION SUMMARY

STATION	TEMPERATURE (°F) AVERAGE MAXIMUM	AVERAGE MINIMUM	AVERAGE	DEPARTURE FROM NORMAL	HIGHEST	DATE	LOWEST	DATE	HEATING DEG. DAYS	COOLING DEG. DAYS	NO. OF DAYS MAX >=90	MAX <=32	MIN <=32	MIN <=0	PRECIPITATION (IN) TOTAL	DEPARTURE FROM NORMAL	GREATEST 24 HOURS	DATE	ICE PELLETS, SNOW TOTAL	MAX DEPTH ON GROUND	DATE	NO. OF DAYS .10 OR MORE	.50 OR MORE	1.00 OR MORE
**LOUISIANA**																								
**NORTHWEST 01**																								
BENTON 5E	90.0M	69.4M	79.7M	-3.2	97	11	61	03	0	464E	15	0	0	0	MA 2.19	-1.45	0.27	16	M 0.0	0		1	0	0
HOSSTON															2.78		1.72	27	0.0			4	2	1
JAMESTOWN															4.36		2.58	27	0.0			5	3	1
KEITHVILLE															5.19		2.49	19	0.0	0		3	3	2
KORAN															2.97		1.40	27	0.0			4	2	1
LOGANSPORT															M 1.50		0.66	19	0.0	0		5	1	0
MANSFIELD 7 NW	90.3M	68.7M	79.5M		97	11	60	03	0	458E	18	0	0	0	M 2.31		0.83	27	0.0			4	2	0
MINDEN	90.8	69.4	80.1	-2.1	96	19+	63	14+	0	477	20	0	0	0	4.30	0.65	2.25	27	0.0	0		5	2	2
MOORINGSPORT 1 N	91.5	69.5	80.5	-2.6	98	19+	62	13+	0	488	21	0	0	0	1.28	-2.02	0.40	19	0.0	0		3	0	0
RED RIVER RSCH STN	91.0M	70.2M	80.6M	-2.5	96	19+	60	03	0	490E	22	0	0	0	M 2.24	-1.45	1.73	27	0.0			3	1	1
SHREVEPORT DWTN															A 1.49		0.24	19	0.0	0		2	0	0
SHREVEPORT DWTN AP	92.3	70.9	81.6	-1.4	98	10	63	13+	0	521	24	0	0	0	1.58	-1.91	0.95	26	0.0			3	1	0
SHREVEPORT AP	92.9	71.1	82.0	-1.0	98	18+	62	03	0	531	25	0	0	0	2.18	-1.47	1.36	26	0.0	0		3	2	1
SHREVEPORT STHRN HILLS	92.6	69.3	81.0	-1.8	99	11	62	03	0	501	26	0	0	0	1.99	-2.37	1.53	27	0.0	0		4	1	1
SHREVEPORT WFO	91.7	72.0	81.8	-1.2	97	31+	65	03	0	530	24	0	0	0	2.16	-1.30	1.33	26	0.0	0		3	2	1
SPRINGHILL															3.94		1.95	24	0.0			3	2	2
VIVIAN															M				0.0					
**--DIVISIONAL DATA------->**			80.8	-1.4B											2.85	-1.09B								
**NORTH CENTRAL 02**																								
ARCADIA															M 7.10		2.98	27	0.0	0		5	3	3
BIENVILLE 3 NE	91.5	69.4	80.5	-1.2	96	12+	61	03	0	485	24	0	0	0	5.88	1.73	3.50	27	0.0			6	3	1
CALHOUN RSCH STN	90.8M	67.5M	79.2M	-2.0	96	11	60	13	0	448E	22	0	0	0	M 3.91	0.38	1.61	27	0.0			6	3	1
COLUMBIA LOCK	90.4M	68.8M	79.6M	-2.0	96	31	64	05+	0	458E	18	0	0	0	M 2.59	-1.79	0.83	27	0.0	0		6	2	0
FARMERVILLE	89.8	69.8	79.8	-2.4	95	19	64	05+	0	467	18	0	0	0	2.80	-1.33	2.08	27	0.0	0		4	1	1
HOMER 1N	89.6M	66.5M	78.1M	-2.4	95	19	57	03	0	413E	15	0	0	0	M 4.42	0.57	2.40	27	0.0			4	3	2
JONESBORO 4 ENE															M				0.0					
MONROE REGIONAL AP	92.1	69.4	80.8	-2.2	96	17+	62	05	0	497	26	0	0	0	1.77	-1.80	1.05	27	0.0	0		4	1	1
MONROE DELTA CC															2.13	-1.61	1.57	27	0.0	0		4	1	1
RUSTON LA TECH	89.0M	68.4M	78.7M	-1.9	94	19+	60	05+	0	432E	12	0	0	0	6.13	2.75	2.49	27	0.0			6	3	2
SAILES FIRE TWR															5.33		2.55	27	0.0	0		6	3	2
WEST MONROE															2.28		1.38	27	0.0			5	1	1
WINNFIELD 3 N	M	M	M								0	0	0	0	M				0.0					
**--DIVISIONAL DATA------->**			79.5	-2.5B											3.76	-0.16B								
**NORTHEAST 03**																								
BASTROP	89.8M	68.3M	79.0M	-2.1	95	24+	61	05+	0	441E	16	0	0	0	2.75	-1.93	2.04	27	0.0	0		5	1	1
LAKE PROVIDENCE	90.0M	70.4	80.2M	-2.3	97	11	66	06+	0	478E	18	0	0	0	4.86	1.48	1.76	15	0.0	0		4	4	3

MONTHLY STATION AND DIVISION SUMMARY

STATION	TEMPERATURE (°F) AVERAGE MAXIMUM	AVERAGE MINIMUM	AVERAGE	DEPARTURE FROM NORMAL	HIGHEST	DATE	LOWEST	DATE	HEATING DEG. DAYS	COOLING DEG. DAYS	NO. OF DAYS MAX >=90	MAX <=32	MIN <=32	MIN <=0	PRECIPITATION (IN) TOTAL	DEPARTURE FROM NORMAL	GREATEST 24 HOURS	DATE	ICE PELLETS, SNOW TOTAL	MAX DEPTH ON GROUND	DATE	NO. OF DAYS .10 OR MORE	.50 OR MORE	1.00 OR MORE
OAK GROVE															M 1.99		1.45	27	0.0	0		3	1	1
OAK RIDGE															2.70		2.50	27	0.0	0		2	1	1
PIONEER 6 W															2.57		1.78	27	0.0	0		3	1	1
RAYVILLE	90.7	70.6	80.6	-1.8	96	11	66	14+	0	493	19	0	0	0	2.38	-1.64	1.68	27	0.0	0		3	1	1
ST JOSEPH 3 N	91.6M	70.3M	80.9M	-1.8	96	10	63	03	0	499E	18	0	0	0	A 7.63	3.21	1.94	27	0.0	0		5	3	1
TALLULAH	M	M	M		95	31+	64	12	0	498E	11	0	0	0	M 5.84	2.19	2.50	27	0.0	0		4	4	3
TALLULAH VICKSBURG RGN	90.6	67.8	79.2	-2.2	95	17	60	13	0	449	23	0	0	0	6.45	2.82	2.94	27	0.0	0		4	3	2
WINNSBORO 2 SE	91.9	69.7	80.8	-2.1	97	11	63	05+	0	496	25	0	0	0	A 2.76	-1.17	1.06	12	0.0	0		4	3	1
WINNSBORO 5 SSE	91.5	68.4	80.0	-2.7	95	31+	62	05+	0	473	27	0	0	0	4.40	0.47	1.40	08	0.0	0		7	4	1
**--DIVISIONAL DATA------->**			80.1	-2.2B											4.06	0.31B								
**WEST CENTRAL 04**																								
GORUM FIRE TWR															M				0.0					
HODGES GARDENS	91.3M	70.0M	80.6M	-2.0	98	13	63	02	0	492E	15	0	0	0	3.85	-0.32	0.90	22	0.0			8	3	0
LEESVILLE	92.1	68.5	80.3	-1.0	97	31+	60	02+	0	482	27	0	0	0	2.34	-1.63	0.64	20	0.0			7	1	0
LEESVILLE 6 SSW															2.27		1.00	22	0.0			5	2	1
MANY 9 WSW															3.30		1.52	16	0.0	0		5	2	1
NATCHITOCHES #2	89.3	70.3	79.8	-3.8	95	23+	65	13+	0	464	12	0	0	0	4.19	0.10	2.43	27	0.0	0		6	3	1
TOLEDO BEND LAKE	92.6M	70.3M	81.5M	-0.4	97	13+	64	03+	0	519E	27	0	0	0	M 1.36	-2.33	0.48	17	0.0	0		4	0	0
ZWOLLE 2 NW															3.09		1.38	16	0.0			5	2	1
**--DIVISIONAL DATA------->**			80.6	-1.2B											3.17	-0.96B								
**CENTRAL 05**																								
ALEXANDRIA	92.7M	71.2M	81.9M	-1.4	98	13	67	14+	0	532E	26	0	0	0	2.64	-1.75	0.93	28	M 0.0	0		6	2	0
ALEXANDRIA 5 SSE	91.8	70.9	81.4	0.3	97	30	63	01	0	514	26	0	0	0	1.16	-2.93	0.80	24	M 0.0	0		3	1	0
BEAVER FIRE TWR															1.67		0.94	19	0.0			4	1	0
BOYCE 3 WNW	89.7	71.7	80.7	-2.0	94	30+	67	03	0	497	17	0	0	0	3.97	-0.38	2.20	27	M 0.0	0		5	2	2
BUNKIE	90.4	71.1	80.8	-1.6	94	31+	65	13+	0	494	21	0	0	0	3.24	-0.64	1.16	08	0.0			7	2	1
CLAYTON															M 5.97		2.00	12	0.0	0		9	4	2
ELMER 2 SW															M				0.0					
EUNICE	91.2	71.6	81.4	-2.4	96	30+	67	03+	0	516	23	0	0	0	2.41	-2.71	0.98	25	0.0			4	2	0
GRAND COTEAU	90.0M	71.6M	80.8M	-1.1	94	31+	66	01	0	498E	18	0	0	0	5.84	1.12	2.25	09	0.0			8	4	1
JENA 4 WSW	91.3	68.1	79.7	-1.6	98	11+	61	13+	0	463	22	0	0	0	4.21	-0.68	1.50	27	0.0			7	3	2
JONESVILLE LOCKS	90.1	70.5	80.3	-2.2	94	25	67	02+	0	484	22	0	0	0	5.63	1.78	3.60	28	0.0	0		8	2	1
LSU DEAN LEE RSCH STN	M	M	M		96	15+	65	08	0	518E	8	0	0	0	M				0.0					
MARKSVILLE															3.06		0.80	12	0.0			7	4	0
NEW ROADS 5 NE	91.8	72.7	82.3	-0.6	96	30	69	04+	0	543	24	0	0	0	3.78	-0.47	1.10	27	0.0			8	3	2
OPELOUSAS															3.54		0.86	27	0.0			7	3	0
PORT ALLEN															3.21		0.85	28	0.0			6	2	0
RED RIVER LOCK #1															4.71		2.13	28	0.0	0		6	2	2

MONTHLY STATION AND DIVISION SUMMARY

STATION	TEMPERATURE (°F) AVERAGE MAXIMUM	AVERAGE MINIMUM	AVERAGE	DEPARTURE FROM NORMAL	HIGHEST	DATE	LOWEST	DATE	HEATING DEG. DAYS	COOLING DEG. DAYS	NO. OF DAYS MAX >=90	MAX <=32	MIN <=32	MIN <=0	PRECIPITATION (IN) TOTAL	DEPARTURE FROM NORMAL	GREATEST 24 HOURS	DATE	ICE PELLETS, SNOW TOTAL	MAX DEPTH ON GROUND	DATE	NO. OF DAYS .10 OR MORE	.50 OR MORE	1.00 OR MORE
RED RIVER LOCK # 2															1.55		0.66	16	0.0			6	1	0
VILLE PLATTE															2.49		1.74	21	0.0			3	1	1
**--DIVISIONAL DATA------->**			81.0	-1.3B											3.32	-1.31B								
**EAST CENTRAL 06**																								
ABITA RVR COVINGTON															M 6.54		0.98	12	0.0			11	7	0
ABITA SPRINGS 1 SW															5.93		1.91	15	M 0.0			11	4	1
ABITA SPRING FIRE TWR															5.02		0.77	27	0.0			10	4	0
ANGIE															M				0.0					
BAKER															A 6.66		1.10	16	0.0			5	2	1
BATON ROUGE CONCORD															4.35		1.35	28	M 0.0			8	3	1
BATON ROUGE METRO AP	90.5	72.1	81.3	-1.7	96	12	67	02	0	514	23	0	0	0	4.65	-0.31	1.28	27	0.0	0		7	3	1
BATON ROUGE SHERWOOD															5.06		1.14	28	0.0			11	4	1
BOGALUSA	M	M	M								0	0	0	0	M				0.0					
CLINTON FORESTRY HQ															3.72		1.00	21	0.0			8	3	1
CLINTON 5 SE	88.1	69.6	78.9	-1.2	92	30+	62	01	0	436	14	0	0	0	3.82	-0.91	1.69	28	0.0			9	1	1
COVINGTON 3 NE															M				0.0					
DENHAM SPRINGS															M 6.00		1.88	28	0.0			9	5	2
HAMMOND 5 E	88.7	71.1	79.9	-1.7	93	30+	63	01	0	467	16	0	0	0	7.29	1.12	1.91	12	0.0			10	6	2
KILLIAN															M 5.36		1.45	22	0.0			11	4	3
LIVERPOOL 6W															M				0.0					
LIVINGSTON															3.44		1.37	28	0.0			7	2	1
LSU BEN-HUR FARM	88.9	72.4	80.6	-1.5	93	30	66	02	0	490	16	0	0	0	4.96	0.33	1.58	28	0.0			9	3	2
MOUNT HERMON 2W															6.13		1.49	07	0.0			9	6	2
NORWOOD															6.56		2.14	28	0.0			11	4	2
OAKNOLIA 2N															6.32		1.27	28	0.0			11	4	2
PINE GROVE FIRE TWR															2.38		0.81	27	0.0			8	1	0
PONCHATOULA 4 SE															M 2.43		0.49	28	0.0			10	0	0
ST FRANCISVILLE															5.37		1.20	19	0.0			12	2	1
SLIDELL	87.2	73.2	80.2	-1.8	92	27	68	01	0	481	7	0	0	0	6.59	-0.24	2.67	12	0.0			10	4	1
SLIDELL AP	88.9	72.9	80.9	-0.8	94	29	66	01	0	501	14	0	0	0	10.69	4.07	2.04	19	0.0			12	7	4
SUN															M 4.70		1.39	16	0.0			7	3	2
TALISHEEK															A 6.92		1.77	08	0.0			11	6	1
TICKFAW 3 ENE															M				0.0					
**--DIVISIONAL DATA------->**			80.3	-1.3B											5.57	-0.41B								
**SOUTHWEST 07**																								
ABBEVILLE															6.79		1.42	12	0.0			9	6	2
BELL CITY 13 SW															5.43		2.70	08	0.0			6	3	2
CROWLEY 2 NE	91.1	71.2	81.1	-1.1	95	26+	67	02+	0	512	21	0	0	0	1.68	-3.91	0.68	25	0.0			4	1	0

LOUISIANA
201307

MONTHLY STATION AND DIVISION SUMMARY

STATION	TEMPERATURE (°F) AVERAGE MAXIMUM	AVERAGE MINIMUM	AVERAGE	DEPARTURE FROM NORMAL	HIGHEST	DATE	LOWEST	DATE	HEATING DEG. DAYS	COOLING DEG. DAYS	NO. OF DAYS MAX >=90	MAX <=32	MIN <=32	MIN <=0	PRECIPITATION (IN) TOTAL	DEPARTURE FROM NORMAL	GREATEST 24 HOURS	DATE	ICE PELLETS, SNOW TOTAL	MAX DEPTH ON GROUND	DATE	NO. OF DAYS .10 OR MORE	.50 OR MORE	1.00 OR MORE
DE RIDDER	91.8	70.1M	80.9M	-1.3	95	30+	67	01	0	503E	25	0	0	0	M 2.60	-2.52	1.70	20	0.0			5	1	1
DRY CREEK 8NW															M				0.0					
HACKBERRY 8 SSW	89.8	75.4	82.6	-1.2	97	01	72	08+	0	555	20	0	0	0	4.60	-1.88	1.73	27	0.0			9	3	1
JENNINGS	91.4	72.5	82.0	-0.2	95	30+	66	02	0	535	24	0	0	0	2.91	-2.03	1.36	06	0.0			7	1	1
KAPLAN															5.65		1.50	12	0.0			12	4	1
LAKE ARTHUR 10 SW	91.7	74.4	83.0	-0.5	96	01	68	03	0	569	23	0	0	0	2.54	-2.90	0.54	26	0.0			7	1	0
LAKE CHARLES 7 NW															4.62		1.44	22	0.0			11	3	2
LAKE CHARLES 2 N															3.86		1.72	20	0.0			6	2	2
LAKE CHARLES PORT															3.94		1.30	21	0.0			8	3	1
LAKE CHARLES AP	92.5	74.0	83.3	0.4	96	25+	68	02	0	574	27	0	0	0	1.71	-3.92	0.51	27	0.0	0		5	1	0
LELAND BOWMAN LOCK	89.3M	72.9M	81.1M	-1.1	94	14+	64	02	0	506E	13	0	0	0	8.52	1.22	3.28	26	M 0.0	0		9	6	2
MOSS BLUFF															2.41		1.05	20	0.0			5	2	1
MOSS BLUFF 2 NNW	92.6	71.1	81.9	-0.2	97	31+	63	04+	0	534	26	0	0	0	1.44	-4.86	0.73	21	0.0			4	1	0
OAKDALE															2.37		0.86	11	0.0			4	3	0
OBERLIN FIRE TWR	90.5	70.9	80.7	-1.2	95	13	65	01	0	496	23	0	0	0	2.03	-3.36	1.00	28	0.0			5	2	1
OLD TOWN BAY															1.42		0.70	13	0.0			5	1	0
ROCKEFELLER WL REFUGE	87.3	74.4	80.9	-0.8	92	01	70	03+	0	500	5	0	0	0	4.70	-1.54	1.25	07	0.0			8	4	1
SULPHUR															1.87		0.85	21	0.0			7	1	0
VINTON 5W	91.8M	71.3M	81.6M		97	13	66	04+	0	521E	23	0	0	0	M 1.78		0.41	21+	0.0			5	0	0
**--DIVISIONAL DATA------->**			81.7	-0.4B											3.60	-2.26B								
**SOUTH CENTRAL 08**																								
BAYOU SORREL LOCK															4.56		0.91	12	0.0			8	4	0
BUTTE LA ROSE															M				0.0					
CARENCRO															6.34		3.04	06	0.0			9	3	2
CARVILLE 2 SW	88.8	73.8	81.3	-1.0	92	31+	69	04	0	511	14	0	0	0	3.48	-1.96	1.60	27	0.0			3	2	2
DONALDSONVILLE 4 SW	88.0	72.1	80.0	-2.0	91	31+	67	01	0	474	11	0	0	0	5.58	-0.78	1.57	22	M 0.0			7	4	2
FRANKLIN 3 NW	87.6M	73.3M	80.5M	-1.0	91	30+	69	02	0	485E	5	0	0	0	M 4.69	-2.46	1.23	25	0.0			9	3	2
JEANERETTE 5 NW	88.7	72.3	80.5	-1.7	93	30	68	02	0	489	15	0	0	0	7.82	0.91	2.14	08	0.0			11	7	2
LAFAYETTE	90.5	73.6	82.0	0.3	94	31+	68	04+	0	535	22	0	0	0	4.91	-0.66	1.20	08	M 0.0	0		12	4	1
LAFAYETTE FCWOS	90.4	73.7	82.0	-1.4	95	31+	69	03+	0	535	21	0	0	0	3.83	-2.47	0.79	05	0.0	0		8	3	0
MORGAN CITY	88.6M	74.3M	81.5M	-0.7	95	24	71	09+	0	518E	12	0	0	0	M 6.66	-0.20	1.27	16	0.0			11	7	1
NAPOLEONVILLE															9.44		1.96	22	0.0			12	7	4
NEW IBERIA AP ACADIANA RGNL	91.3	74.8	83.0	0.3	96	12	71	04+	0	567	23	0	0	0	7.52	0.88	2.01	15	0.0			11	6	1
PLAQUEMINE 2 N															6.77		1.48	12	0.0			8	5	4
ST GABRIEL															3.42		0.83	08	0.0			9	2	0
ST MARTINVILLE 3 SW	89.1	71.9	80.5	-1.1	94	13	67	02+	0	487	17	0	0	0	3.05	-3.16	0.61	28	0.0			9	3	0
**--DIVISIONAL DATA------->**			81.3	-0.8B											5.56	-1.26B								
**SOUTHEAST 09**																								

LOUISIANA
201307

MONTHLY STATION AND DIVISION SUMMARY

STATION	TEMPERATURE (°F) AVERAGE MAXIMUM	AVERAGE MINIMUM	AVERAGE	DEPARTURE FROM NORMAL	HIGHEST	DATE	LOWEST	DATE	HEATING DEG. DAYS	COOLING DEG. DAYS	NO. OF DAYS MAX >=90	MAX <=32	MIN <=32	MIN <=0	PRECIPITATION (IN) TOTAL	DEPARTURE FROM NORMAL	GREATEST 24 HOURS	DATE	ICE PELLETS, SNOW TOTAL	MAX DEPTH ON GROUND	DATE	NO. OF DAYS .10 OR MORE	.50 OR MORE	1.00 OR MORE
BOOTHVILLE ASOS	86.3	77.0	81.6	-1.3	90	29+	72	03	0	527	2	0	0	0	6.48	-0.02	2.12	03	0.0			12	3	2
CONVENT 2S															5.17		1.69	22	0.0			5	4	3
DUTCHTOWN #2															5.33		1.85	22	0.0			8	3	2
GALLIANO	87.5	75.4	81.4	-0.9	92	29+	71	06+	0	515	9	0	0	0	8.17	0.16	2.22	12	0.0			11	6	5
GONZALES															6.79		2.00	22	0.0			5	4	3
GRAND ISLE															M 5.16		1.90	29	0.0			8	4	2
HOUMA	87.6M	72.8M	80.2M	-2.6	92	13	69	02+	0	478E	10	0	0	0	M 12.14	4.28	2.99	17	0.0			13	8	5
LUTCHER															2.94		1.12	16	0.0			7	1	1
MARRERO 9 SSW	M	M	M		93	31+	71	02	0	531E	6	0	0	0	M				0.0					
NEW ORLEANS AP	89.1	75.6	82.3	-1.0	93	31+	73	21+	0	546	18	0	0	0	3.97	-1.96	1.03	15	0.0	0		8	4	1
NEW ORLEANS AUDUBON	89.6M	74.7M	82.2M	-2.0	96	29	72	21+	0	538E	16	0	0	0	M 5.67	-2.27	1.58	11	0.0			9	5	1
NEW ORLEANS ALGIERS															M				0.0					
NEW ORLEANS LKFRNT AP	89.3	78.0	83.6	-0.6	94	29	74	11	0	585	18	0	0	0	4.00	-1.68	0.83	07	0.0			9	2	0
TERRYTOWN 3S	89.5	75.0	82.2	-1.9	94	31+	71	12	0	543	16	0	0	0	4.78	-1.83	2.23	12	M 0.0			6	3	1
THIBODAUX 4 SE	88.2	72.3	80.3	-1.8	93	27+	68	01	0	481	13	0	0	0	8.24	-0.05	2.33	22	0.0			10	5	3
**--DIVISIONAL DATA------->**			81.7	-0.7B											5.59	-1.38B								

LOUISIANA
201307

DAILY PRECIPITATION (INCHES)

STATION	TOTAL	DAY OF MONTH																														
		01	02	03	04	05	06	07	08	09	10	11	12	13	14	15	16	17	18	19	20	21	22	23	24	25	26	27	28	29	30	31
**LOUISIANA**																																
**NORTHWEST 01**																																
BENTON 5E	MA 2.19								0.07				0.06				0.27				-	-	-	-	-	*	1.79a					
HOSSTON	2.78												0.21				0.72										0.13	1.72				
JAMESTOWN	4.36			0.01									0.69				0.55			0.15	0.03		0.17		0.08	0.07		2.58	0.03			
KEITHVILLE	5.19								0.09	0.03			0.01			0.01	0.01			2.49			0.01			0.90		1.64				
KORAN	2.97												0.87				0.36			0.20					0.09	0.05		1.40				
LOGANSPORT	M 1.50			0.14	-				0.22				T			0.03	0.16			0.66			0.24					0.05				-
MANSFIELD 7 NW	M 2.31								0.02	0.01		T	T	-		0.01	0.33	0.30		0.03		0.05	0.05			0.68		0.83				-
MINDEN	4.30												0.41				0.30			1.02					0.32			2.25				
MOORINGSPORT 1 N	1.28								0.05							0.04	0.37			0.40	0.03							0.35	0.04			
RED RIVER RSCH STN	M 2.24								0.16				0.09				0.15	0.03		0.06					0.01			1.73	0.01	-		
SHREVEPORT DWTN	A 1.49								0.06				0.03			*	0.15a			0.24	0.12		T		0.01		*	0.88a	T			
SHREVEPORT DWTN AP	1.58							0.05	0.02			0.07				0.15		0.02	0.30	T		T		T			0.95	0.02				
SHREVEPORT AP	2.18								T			T				0.29	T		0.53	T		T		T			1.36	T				
SHREVEPORT STHRN HILLS	1.99												T			0.04	0.10	0.13		0.12	0.06				T	T		1.53	0.01			
SHREVEPORT WFO	2.16								T			T				0.28			0.51			0.01		T	0.01		1.33	0.02				
SPRINGHILL	3.94											0.23					0.06					T			1.95			1.70				
VIVIAN	M	-	-	-	-	-	-	-	-	-	-	-	-	-	-	0.05	1.10	-	-	-	-	-	-	-	-	-	-	-	-	-	-	-
**NORTH CENTRAL 02**																																
ARCADIA	M 7.10												1.04				0.04		-	2.14	0.06		0.40	-	0.39			2.98			0.05	
BIENVILLE 3 NE	5.88											0.50				0.88			0.38			0.10		0.05	0.47			3.50				
CALHOUN RSCH STN	M 3.91				-								0.28				0.71	0.13		0.99					0.19			1.61				
COLUMBIA LOCK	M 2.59								-			0.08	0.30				0.21	-		-	0.81			0.14	0.11	0.04		0.83	0.07		-	
FARMERVILLE	2.80												0.17			0.02				0.31								2.08	T		0.22	
HOMER 1N	M 4.42				0.06							0.03	0.33			-	-	-		0.57			0.02		1.01	-		2.40				
JONESBORO 4 ENE	M	-	-	-	-	-	-	-	-	-	-	-	-	-	-	-	-	-	-	-	-	-	-	-	-	-	-	-	-	-	-	-
MONROE REGIONAL AP	1.77											T			T	0.04			0.17	0.01		T		0.18	0.03		0.29	1.05				T
MONROE DELTA CC	2.13															0.04	0.10	T		0.22					0.20	T		1.57				
RUSTON LA TECH	6.13												2.01				0.73			0.19	0.02		0.36		0.33			2.49				
SAILES FIRE TWR	5.33			0.03					0.01				1.12				0.43			0.07			0.53		0.12	0.45		2.55	0.02			
WEST MONROE	2.28											0.10					0.27			0.39					0.10	0.04		1.38				
WINNFIELD 3 N	M	-	-	-	-	-	-	-	-	-	-	-	-	-	-	-	-	-	-	-	-	-	-	-	-	-	-	-	-	-	-	-
**NORTHEAST 03**																																
BASTROP	2.75												0.12				0.17			0.10			T		0.32			2.04				
LAKE PROVIDENCE	4.86															1.76				0.77			0.04			1.12		1.17				
OAK GROVE	M 1.99															0.15				-					0.09	0.30		1.45				
OAK RIDGE	2.70															T									0.20			2.50				
PIONEER 6 W	2.57															0.23				0.49					0.07			1.78				
RAYVILLE	2.38												0.02			0.28	0.09			0.08					0.15	0.08		1.68				
ST JOSEPH 3 N	A 7.63						0.40		0.62			*	3.65a			0.88				0.10								1.94	0.04			
TALLULAH	M 5.84											1.55	0.54							1.25						-		2.50				

LOUISIANA
201307

DAILY PRECIPITATION (INCHES)

STATION	TOTAL	01	02	03	04	05	06	07	08	09	10	11	12	13	14	15	16	17	18	19	20	21	22	23	24	25	26	27	28	29	30	31
TALLULAH VICKSBURG RGN	6.45					T				T		0.08			0.16	T			0.87					T	2.39		0.01	2.94				
WINNSBORO 2 SE	A 2.76								0.21				1.06			*	0.31a			0.57								0.61				
WINNSBORO 5 SSE	4.40								1.40				0.94			0.21	0.31	0.25		0.54								0.75				
**WEST CENTRAL 04**																																
GORUM FIRE TWR	M	-	-	-	-	-	-	-	-	-	-	-	-	-	-	-	-	-	-	-	-	-	-	-	-	-	-	-	-	-	-	-
HODGES GARDENS	3.85			0.02					0.31				0.02				0.06	0.27		0.17	0.87	0.20	0.90			0.88				0.15		
LEESVILLE	2.34			0.25					0.03								0.03	0.31		0.01	0.64		0.10			0.35		0.41	0.21			
LEESVILLE 6 SSW	2.27			0.02					0.21								0.02	0.12					1.00			0.12				0.78		
MANY 9 WSW	3.30								0.04	0.04		0.04					1.52	0.04		0.20	0.01		0.07			0.90	0.01	0.16	0.03	0.24		
NATCHITOCHES #2	4.19			0.60						T			0.13				0.55	T				0.02	0.11			0.33		2.43	0.02			
TOLEDO BEND LAKE	M 1.36									0.07							-	0.48		0.04			0.32					0.13	0.32			
ZWOLLE 2 NW	3.09								T	T							1.38	0.60	T		0.23							0.46		0.42		
**CENTRAL 05**																																
ALEXANDRIA	2.64																	0.15		0.22	0.15			0.07		0.42		0.70	0.93			
ALEXANDRIA 5 SSE	1.16							0.02								0.19	0.01								0.80			0.14				
BEAVER FIRE TWR	1.67								0.08	0.21			0.07			0.10				0.94		0.03	0.16			0.05		0.03				
BOYCE 3 WNW	3.97											0.03				0.01	0.17		0.15	1.00		0.04			0.37			2.20				
BUNKIE	3.24						0.06		1.16				0.15			0.26	0.31		0.68				0.01	0.05		0.01		0.16	0.39			
CLAYTON	M 5.97												2.00			-		0.29	0.26	0.15		0.01	0.35	0.79		0.81		1.16	0.15			
ELMER 2 SW	M	-	-	-	-	-	-	-	-	-	-	-	-	-	-	-	-	-	-	-	-	-	-	-	-	-	-	-	-	-	-	-
EUNICE	2.41									0.83			0.04				0.19	0.04								0.98		0.05	0.28			
GRAND COTEAU	5.84						0.80	0.86		2.25			0.91			0.23					0.35	0.25	0.07						0.12			
JENA 4 WSW	4.21								0.12				1.31			0.30	0.11	T		0.72	0.15		T					1.50				
JONESVILLE LOCKS	5.63												0.30						0.30	0.20	0.03		0.10	0.10		0.17		0.83	3.60			
LSU DEAN LEE RSCH STN	M		-	-	-	-	-	-	0.38	-	-	-	-	-	-	0.27	0.25		-	-	-	-	0.15	0.07		0.50	-	-	-	0.58		-
MARKSVILLE	3.06												0.80			0.51		0.11	0.04			0.64		0.31				0.11	0.54			
NEW ROADS 5 NE	3.78			0.23		0.07	0.01	0.27	0.20						0.50	0.18									0.02		0.20	1.10		1.00		
OPELOUSAS	3.54					0.32	0.54										0.75		0.22			0.40	0.42					0.86	0.03			
PORT ALLEN	3.21			T	0.77			0.05	0.02	T			T			T	0.07	0.40		0.06	0.32	0.20	0.42			T		0.05	0.85			
RED RIVER LOCK #1	4.71								1.50				0.08						0.15	0.18			0.45					0.22	2.13			
RED RIVER LOCK # 2	1.55																0.66			0.08	0.10		0.10			0.10		0.10	0.41			
VILLE PLATTE	2.49												0.40				0.02					1.74							0.33			
**EAST CENTRAL 06**																																
ABITA RVR COVINGTON	M 6.54						0.57	0.10	0.27	0.93	-	-	0.98	-		0.07	0.48	0.01			0.58	0.15	0.07	0.81	-				0.55			0.97
ABITA SPRINGS 1 SW	5.93			T		0.28	0.34	0.04	0.10	0.07			0.73	T		1.91	0.36	0.01	0.13	0.03	0.01	0.01	0.04	0.76				0.10	0.79			0.22
ABITA SPRING FIRE TWR	5.02					0.15	0.67	0.05	0.55	0.01			0.72	0.30	0.42	0.09	0.05		0.02		0.40	0.06	0.02	0.22				0.77	0.09			0.43
ANGIE	M	-	-	-	-	-	-	-	-	-	-	-	-	-	-	-	-	-	-	-	-	-	-	-	-	-	-	-	-	-	-	-
BAKER	A 6.66	0.39a					*	*	0.00a	0.59			0.12	*	*	1.20a	1.10			0.25	*	*	0.02a	0.19				*	2.80a			
BATON ROUGE CONCORD	4.35				0.56	T	T	0.06	0.57	T	T		0.44			0.11	0.23	0.46		0.02	T	0.09	0.41	0.03		T		0.02	1.35			
BATON ROUGE METRO AP	4.65			0.48				0.05	T			0.05	T		0.90	0.92		T	0.35	T	0.17	0.44	T		0.01		T	1.28				
BATON ROUGE SHERWOOD	5.06				0.37	T	0.03	0.03	0.14	0.59	0.29		0.32			0.83	0.41	0.50		0.04		0.20	0.14	T		0.01		0.02	1.14			
BOGALUSA	M	-	-	-	-	-	-	-	-	-	-	-	-	-	-	-	-	-	-	-	-	-	-	-	-	-	-	-	-	-	-	-

DAILY PRECIPITATION (INCHES)

STATION	TOTAL	01	02	03	04	05	06	07	08	09	10	11	12	13	14	15	16	17	18	19	20	21	22	23	24	25	26	27	28	29	30	31
CLINTON FORESTRY HQ	3.72								0.44	0.06			0.12			0.36	0.11	0.12				1.00				0.05		0.90	0.56			
CLINTON 5 SE	3.82					0.28			0.33		0.30		0.17			0.29	0.04	0.20					0.28	0.19		0.05			1.69			
COVINGTON 3 NE	M	-	-	-	-	-	-	-	-	-	-	-	-	-	-	-	-	-	-	-	-	-	-	-	-	-	-	-	-	-	-	-
DENHAM SPRINGS	M 6.00			0.52	0.35		T	0.05	0.15		T		1.00		T	0.09		0.21	-	0.02	-	0.44	0.75	0.50		0.03		0.01	1.88			
HAMMOND 5 E	7.29					0.13	0.93	0.22	1.22	0.21			1.91				0.87	0.03	0.12	0.04			0.69	T		T		T	0.92			
KILLIAN	M 5.36				0.13			T	0.20	0.15			1.04				1.00	0.15	0.22	0.17			1.45					0.20	0.65			-
LIVERPOOL 6W	M				0.13		0.25		1.20	-	-		-			-	-	-	-	-	-	-	-	-	-	-	-	-	-	-	-	-
LIVINGSTON	3.44						0.05		0.21				0.76				0.31	0.05	0.11	0.17			0.37	0.04					1.37			
LSU BEN-HUR FARM	4.96				0.55		0.08		0.31		0.04		1.26			0.21	0.08	0.12		0.26			0.30	0.15		T		0.02	1.58			
MOUNT HERMON 2W	6.13						1.06	1.49	0.56				0.84			0.34	0.24	0.08						0.36					0.51			0.65
NORWOOD	6.56					0.33	0.75						1.00			0.17	0.76	0.34		0.25	0.24		0.19	0.31		0.08			2.14			
OAKNOLIA 2N	6.32				0.14	T			0.81				1.25			0.52	0.36	0.16	T	0.43	0.07	0.06	0.36	0.45		0.36		0.08	1.27			
PINE GROVE FIRE TWR	2.38							0.18	0.02	0.16			0.45						0.36				0.13	0.13				0.81	0.14			
PONCHATOULA 4 SE	M 2.43					0.34	0.19			0.13		0.20	-			0.15	0.12		0.11	0.34			0.31	0.05					0.49			
ST FRANCISVILLE	5.37				0.06		0.43	0.02	0.03	0.34			0.08			0.15	0.06	0.37	0.15	1.20	0.08	0.20	0.40	0.53		0.42		0.36	0.49			
SLIDELL	6.59			0.09	0.01		0.09	0.02	0.73	0.01			2.67		0.14		0.35	0.42			0.12	0.14	0.81	0.06					0.78		0.15	
SLIDELL AP	10.69	T		0.05		0.01	0.41	0.94	1.75			1.72	T	1.14		0.04	0.78			2.04	0.27	0.31	0.13		T			0.27			0.82	0.01
SUN	M 4.70						0.05		-	-	-	-	-	-	-	-	1.39	0.03	0.87	0.08			0.27	0.28				0.23	0.33			1.17
TALISHEEK	A 6.92			0.08		*	0.27a	0.02	1.77	0.03			0.87	0.11	0.01	0.22	0.39	0.28	0.83	0.55	0.02	0.03	0.04	0.66				0.13	0.61			
TICKFAW 3 ENE	M	-	-	-	-	-	-	-	-	-	-	-	-	-	-	-	-	-	-	-	-	-	-	-	-	-	-	-	-	-	-	-
**SOUTHWEST 07**																																
ABBEVILLE	6.79								0.91	0.08	0.06		1.42	T			0.26	T	0.63	T	0.42		T			1.41	0.11		0.97	0.52		
BELL CITY 13 SW	5.43						1.53	0.64	2.70				0.26				0.04					0.10	0.01	0.01			0.01		0.03	0.10		
CROWLEY 2 NE	1.68						0.09	0.01					0.08				0.17	0.16	0.02			0.09				0.68			0.38			
DE RIDDER	M 2.60									0.10			-					T		0.25	1.70	T	0.40					T	0.15			
DRY CREEK 8NW	M	-	-	-	-	-	-	-	-	-	-	-	0.12	-	-	-	0.42	0.09	0.14	0.80	0.66	-	0.53	-	-	-	-	-	-	-	-	-
HACKBERRY 8 SSW	4.60	0.01	0.01	0.01					0.82				0.07			0.19	0.46	0.14	0.21			0.11	0.06					1.73	0.63	0.15		
JENNINGS	2.91						1.36	0.29	0.02		0.19		0.03				0.41	0.12	0.02	0.02	0.22	T	0.19	0.02		0.02						
KAPLAN	5.65						0.12	0.05	0.12				1.50				0.17	0.18		0.30	0.60	0.20	0.42		0.05			0.62	0.95	0.37		
LAKE ARTHUR 10 SW	2.54							0.28	0.07				0.46				0.03	0.14	0.03				0.24				0.54	0.01	0.41	0.32	0.01	
LAKE CHARLES 7 NW	4.62								1.00		0.60	0.20	T				T	0.10	0.20	0.15	0.16	0.37	1.44	0.10		0.30	T					
LAKE CHARLES 2 N	3.86								0.15		0.07			0.45				0.04	0.06	0.10	1.72	0.14	1.05			0.06		0.01		0.01		
LAKE CHARLES PORT	3.94			0.01	0.01				0.17	0.01		0.28	0.01	0.28	0.01			0.27	0.69	0.12	0.69	1.30	0.07			0.01	0.01					
LAKE CHARLES AP	1.71						0.01	0.04				0.43	0.01			0.07	0.15	T		0.11		0.38				T		0.51	T			
LELAND BOWMAN LOCK	8.52						0.04	0.12	1.42				0.05			0.02	0.45	0.07	0.02	0.10			0.56			0.80	3.28		0.79	0.80		
MOSS BLUFF	2.41			0.01				T	0.05		0.02	T	0.01	0.04				0.04	0.37	0.12	1.05	0.03	0.57			0.10			T			
MOSS BLUFF 2 NNW	1.44							0.07		0.01							T	0.27	0.12	0.17	0.03	0.73				T		0.04				
OAKDALE	2.37											0.86					0.03		0.61						0.37				0.50			
OBERLIN FIRE TWR	2.03							0.18	0.02											0.50			0.22	0.11					1.00			
OLD TOWN BAY	1.42								0.18			T	T	0.70					0.20	T	0.15		0.05				0.14					
ROCKEFELLER WL REFUGE	4.70					0.01	0.90	1.25	0.51		0.23	0.01	0.06	0.03		0.01	0.32	0.10	0.01	0.08		0.38	0.05			0.06	0.04	T	0.64	0.01		
SULPHUR	1.87							0.16					0.01				0.20	0.15	0.10	0.20	0.20	0.85										

LOUISIANA
201307

DAILY PRECIPITATION (INCHES)

STATION	TOTAL	01	02	03	04	05	06	07	08	09	10	11	12	13	14	15	16	17	18	19	20	21	22	23	24	25	26	27	28	29	30	31
VINTON 5W	M 1.78							0.06	0.31									0.21		0.41	0.25	0.41	0.07		-			0.05	0.01			
**SOUTH CENTRAL 08**																																
BAYOU SORREL LOCK	4.56						0.25	0.20	0.45	0.06			0.91				0.55	0.90		0.07			0.07	0.40				T	0.70			
BUTTE LA ROSE	M	-	-	-	-	-	-	-	-	-	-	-	-	-	-	-	-	-	-	-	-	-	-	-	-	-	-	-	-	-	-	-
CARENCRO	6.34						3.04	0.02	1.23		0.02		0.96				0.10	0.10	0.30		0.23		0.20	0.02					0.12			
CARVILLE 2 SW	3.48						0.33	1.20				0.01			0.06	0.09	0.02		0.04			0.08					0.05	1.60				
DONALDSONVILLE 4 SW	5.58							0.40	1.42				0.85			0.05	0.37			0.77		0.01	1.57						0.14			
FRANKLIN 3 NW	M 4.69						0.30	0.25			0.75	1.05	0.13			0.08	0.20				0.05	0.34				1.23		0.24		-		0.07
JEANERETTE 5 NW	7.82				0.75			1.32	2.14				0.50				0.38	0.06	0.07	0.25	0.02	0.15	0.59	0.03		0.68	0.29	0.08	0.51			
LAFAYETTE	4.91						0.58	0.23	1.20				0.05				0.80	0.05	0.30	0.50	0.23		0.35	0.01		0.18	0.18	0.11	0.14			
LAFAYETTE FCWOS	3.83					0.79	0.08	0.43		0.40		0.05				0.54	0.13	0.15		0.75		0.04	0.05			0.35		0.07	T			
MORGAN CITY	M 6.66				0.50	-	0.07	0.69	0.32	0.18			0.56	0.21			1.27	0.79		0.05		0.01	0.83	0.08			0.37		0.73			
NAPOLEONVILLE	9.44							1.95	0.75		0.01		1.01			0.81	0.51	0.10		1.31			1.96				0.17	0.30	0.46			0.10
NEW IBERIA AP ACADIANA RGNL	7.52					0.10	0.93	0.96		0.03	T	0.25			0.01	2.01	0.76	T	0.71	T	T	0.28	0.23		T	0.36	0.09	0.80				
PLAQUEMINE 2 N	6.77				1.10		0.10	0.03	0.16				1.48			0.04	1.06	T		0.05			0.07	0.05		0.26		T	0.93		1.44	
ST GABRIEL	3.42				0.13		0.11	T	0.83	0.01			0.34			0.33		0.29		0.34	0.01		0.18	0.07		0.01		0.02	0.75			
ST MARTINVILLE 3 SW	3.05							0.56	0.28								0.30	0.05	0.13	0.21			0.55			0.11	0.25		0.61			
**SOUTHEAST 09**																																
BOOTHVILLE ASOS	6.48			2.12	T	1.31	0.46	T	0.03			0.12		0.20		0.14	0.17	0.08			0.01	0.30	0.01			0.78	0.27	0.14	0.34			
CONVENT 2S	5.17								1.45								1.35			0.13			1.69	T					0.55			T
DUTCHTOWN #2	5.33				0.07		0.03	0.35	0.14				1.33	0.75		0.24	0.19			0.05		0.01	1.85	0.01				0.01	0.26			0.04
GALLIANO	8.17	0.11		0.10			1.00	0.50	1.00			0.01	2.22	1.32			0.26	0.01					1.10	0.20			0.02		0.32			
GONZALES	6.79								0.02	0.02			1.70	0.05		0.09	0.33			1.71			2.00	0.03					0.75			0.09
GRAND ISLE	M 5.16			0.50	-	-	-	-	-			0.50	-				0.20	0.40			0.01		0.10				0.10	0.05	1.40	1.90		
HOUMA	M 12.14			-		0.02			1.44	0.11		0.22	0.62			0.92	1.47	2.99					1.33				0.36		1.22	0.94	0.23	0.27
LUTCHER	2.94					T			0.25	0.02						0.08	1.12			0.49			0.47	0.19					0.10			0.22
MARRERO 9 SSW	M			-	-	-	-	-	-	-	-	-	-	-	1.49	-	-	0.05			-	-	-	0.05		-	-	-	-	-	-	-
NEW ORLEANS AP	3.97	0.54	T	0.17		0.07	0.13	0.55	0.45		T	0.05			T	1.03			0.54		T	0.37	T					0.07				
NEW ORLEANS AUDUBON	M 5.67	0.06		0.04	0.08	0.75	0.89	0.27	0.62		0.15	1.58							0.74		0.01	0.17	0.04					0.03		0.24	-	-
NEW ORLEANS ALGIERS	M	0.70		-	-	-	-	-	-	-	-	-	-	-	-	-	-	-	-	-	-	-	-	-	-	-	-	-	-	-	-	-
NEW ORLEANS LKFRNT AP	4.00	0.04	0.05	0.48		0.21	0.35	0.83	0.03			0.40			0.66	0.04	0.22				0.47	0.12	0.07					0.03				
TERRYTOWN 3S	4.78			0.58	0.01	0.33	0.79		0.06	0.01			2.23				0.03		0.41	0.01		0.05	0.01			0.03			T			0.23
THIBODAUX 4 SE	8.24			0.04		0.06	0.02	T	1.50	1.84			0.28			0.01	0.70	0.27	T	0.34		0.05	2.33	T		0.01	0.15		0.54			0.10

LOUISIANA
201307

DAILY TEMPERATURES (°F)

STATION	OB.TIME	MAX/MIN	01	02	03	04	05	06	07	08	09	10	11	12	13	14	15	16	17	18	19	20	21	22	23	24	25	26	27	28	29	30	31	AVERAGE
**LOUISIANA**																																		
**NORTHWEST 01**																																		
BENTON 5E	08	MAX	88	84	85	89	88	92	91	92	92	95	97	93	94	92	84	87	91	93	94							90	84	82	88	91	93	90.0
		MIN	63	63	61	62	63	67	71	73	75	78	77	70	63	68	70	71	72	71	72							71	70	65	67	75	77	69.4
MANSFIELD 7 NW	08	MAX	88	85	86	87	89	91	90	91	93	95	97	90		91	82	89	91	92	95	88	92	92	95	95	91	92	89	81	89	92		90.3
		MIN	62	63	60	61	62	64	68	70	72	75	77	69		65	69	68	71	71	70	70	69	71	76	72	68	71	71	65	69	73		68.7
MINDEN	07	MAX	89	86	86	87	89	91	90	92	93	95	96	94	93	93	85	91	90	93	96	88	91	92	94	94	89	91	88	88	88	91	93	90.8
		MIN	64	64	63	63	63	64	70	73	74	74	75	70	63	63	70	71	73	72	71	71	71	72	77	72	70	70	71	66	66	72	72	69.4
MOORINGSPORT 1 N	08	MAX	90	88	88	88	89	91	90	91	91	97	98	96	96	92	83	87	90	92	98	89	92	94	97	96	93	94	84	84	89	92	97	91.5
		MIN	63	62	63	63	63	67	70	73	75	76	77	69	62	63	70	69	70	70	71	71	72	73	76	73	69	72	69	66	70	73	75	69.5
RED RIVER RSCH STN	07	MAX	89	87	87	89	90	91	91	92	92	93	96	91	95	91	84	90	91	93	96	89	91	92	95	94	91	93	88	84		91	94	91.0
		MIN	63	65	60	62	63	69	71	74	74	76	78	70	61	68	71	70	74	71	72	71	72	76	77	72	67	72	71	66		74	75	70.2
SHREVEPORT DWTN AP	24	MAX	87	88	88	90	92	92	93	94	96	98	94	96	92	85	92	93	94	97	89	93	93	96	95	92	93	88	85	90	92	96	97	92.3
		MIN	67	64	63	64	64	70	73	74	75	77	72	70	63	70	71	71	73	72	72	73	73	77	73	73	70	72	69	66	75	75	77	70.9
SHREVEPORT AP	24	MAX	88	89	90	93	93	92	94	93	96	97	94	98	93	85	92	93	94	98	89	93	94	96	96	91	94	89	86	91	94	97	97	92.9
		MIN	68	67	62	65	65	68	73	73	74	76	72	72	65	71	71	71	74	72	73	72	72	76	75	74	68	73	71	67	74	74	76	71.1
SHREVEPORT STHRN HILLS	07	MAX	90	87	89	90	92	93	91	93	93	97	99	94	98	93	85	92	92	94	97	88	94	96	97	97	90	95	90	83	91	95	97	92.6
		MIN	65	65	62	63	64	65	70	72	73	73	75	71	63	63	71	70	70	72	72	71	70	71	75	73	69	69	72	67	67	73	73	69.3
SHREVEPORT WFO	24	MAX	88	86	88	90	91	91	93	93	95	97	95	97	84	86	91	91	93	95	88	93	93	95	95	90	93	90	84	90	93	97	97	91.7
		MIN	68	67	65	67	69	69	73	73	74	76	74	73	73	71	71	71	75	73	73	72	72	76	75	74	72	73	70	69	75	73	76	72.0
**NORTH CENTRAL 02**																																		
BIENVILLE 3 NE	23	MAX	88	87	89	91	92	91	93	93	94	96	92	96	92	87	93	92	95	95	94	92	91	94	94	88	92	89	80	90	92	93	93	91.5
		MIN	62	64	61	63	62	69	73	73	72	75	70	68	62	66	68	72	72	71	70	70	71	75	76	72	69	72	70	64	73	72	74	69.4
CALHOUN RSCH STN	08	MAX	87	86	87		90	92	89	91	91	95	96	95	93	90	88	91	90	93	95	95	91	92	94	93	90	91	88	80	89	91	92	90.8
		MIN	62	61	62		63	64	68	74	70	71	77	67	60	62	64	67	69	69	69	68	68	73	75	71	68	67	69	63	66	68	70	67.5
COLUMBIA LOCK	07	MAX	86	85	86	87	88	90	89		94	95	94	94	90	91	89	87		93		91	92	93	93	95	90	90	91	82	90		96	90.4
		MIN	65	65	65	64	64	67	71		74	74	75	69	65	65	65	67		71		69	70	73	73	71	71	69	69	68	68		70	68.8
FARMERVILLE	07	MAX	85	84	85	86	87	90	88	90	92	94	94	94	91	89	87	92	91	94	95	90	92	93	94	94	88	89	84	84	86	90	91	89.8
		MIN	64	66	64	64	64	68	71	73	73	75	76	68	66	66	67	72	72	74	69	71	72	73	77	71	71	69	69	66	68	72	74	69.8
HOMER 1N	07	MAX	86	85	85	86	87	90	88	90	92	94	94	93	92	88				91	95	89	91	91	94	94		89	87	81	86	90	92	89.6
		MIN	62	60	57	58	58	62	62	70	71	73	73	68	58	58				68	68	69	68	74	77	70		67	70	62	67	72	73	66.5
MONROE REGIONAL AP	24	MAX	86	88	89	90	92	90	93	92	94	96	95	94	92	91	93	91	96	95	92	93	93	95	95	91	93	89	84	91	93	95	95	92.1
		MIN	65	66	63	63	62	67	71	73	73	76	72	69	65	64	68	72	73	71	70	69	71	75	73	72	70	67	70	66	69	73	74	69.4
RUSTON LA TECH	08	MAX	86	84	86	87	88		89	89		91	94	90			92	89		92	94	88	89	90	93	93		90	85	77	88	90	91	89.0
		MIN	62	63	60	60	60		66	71		72	76	68			61	70		71	71	70	71	73	76	71		70	73	65	66	71	73	68.4
WINNFIELD 3 N	24	MAX																																M
		MIN																																M
**NORTHEAST 03**																																		
BASTROP	07	MAX		84	85	86	87	90	88	89	91	93	94	94	91	88	87	90	89	93	95	90	93	93	95	95	88	88		80	87	90	91	89.8
		MIN		64	61	62	61	62	68	71	73	75	75	69	64	62	65	69	72	71	68	68	70	71	76	73	70	66		65	66	71	72	68.3
LAKE PROVIDENCE	07	MAX	86	86	86	88	90	90	89	92	94	96	97	93	93	86	90	90	89	93	93	90	91	91	95	94		88	82	81	87	89	92	90.0
		MIN	67	66	66	66	66	66	70	73	74	76	76	72	69	67	69	69	73	73	69	69	72	72	75	74	72	67	67	68	69	73	77	70.4

LOUISIANA
201307

DAILY TEMPERATURES (°F)

STATION	OB.TIME	MAX/MIN	01	02	03	04	05	06	07	08	09	10	11	12	13	14	15	16	17	18	19	20	21	22	23	24	25	26	27	28	29	30	31	AVERAGE
RAYVILLE	07	MAX	87	86	87	87	88	90	89	90	93	94	96	94	92	89	87	90	89	93	94	91	94	93	94	95	90	90	89	83	88	94	95	90.7
		MIN	66	66	66	66	67	66	70	73	75	76	77	72	66	66	67	70	74	74	70	70	71	74	76	73	72	69	69	70	69	73	75	70.6
ST JOSEPH 3 N	08	MAX	93	88	89	90		92	92	84	94	96	95	91	92	90	89	93	91	95	89	92	93	93	94	92								91.6
		MIN	64	64	63	66		68	73	73	74	76	77	70	65	69	68	72	74	71	68	71	64	75	70	75		66	72	69	71	75	75	70.3
TALLULAH	08	MAX									93		93	89			89		93	95	93			91	95		91				95	95	95	M
		MIN									71		69	64			66		69	69	69			75	71		66				71	69	69	M
TALLULAH VICKSBURG RGN	24	MAX	86	87	89	90	89	88	91	92	93	93	92	92	90	91	91	91	95	91	91	93	91	94	93	86	90	87	85	90	92	93	94	90.6
		MIN	61	62	61	63	65	65	72	70	71	73	71	63	60	63	66	69	70	67	66	68	69	75	77	73	68	63	71	67	70	71	73	67.8
WINNSBORO 2 SE	08	MAX	88	88	87	89	90	91	91	93	94	95	97	93	91	90	92	91	91	95	95	94	94	93	95	95	89	91	92	84	91	95	96	91.9
		MIN	64	64	63	65	63	67	73	72	74	75	75	69	64	65	69	69	73	71	69	72	71	74	76	74	71	67	72	68	70	71	72	69.7
WINNSBORO 5 SSE	07	MAX	88	86	88	89	90	93	90	93	92	94	95	90	93	90	90	90	90	93	94	93	93	93	94	95	91	91	91	90	90	94	95	91.5
		MIN	63	62	62	64	62	66	70	71	72	73	75	69	63	64	68	69	71	70	67	69	70	72	75	72	70	66	69	68	67	71	71	68.4
**WEST CENTRAL 04**																																		
HODGES GARDENS	08	MAX	91	86	88	88	92	94	94	91	89	96	96	87	98	93	84	88	93	93	93	89	92	88	94	94								91.3
		MIN	65	63	68	68	71	67	67	71	72	73	73	73	68	69	72	68	72	73	68	67	71	71	75	74								70.0
LEESVILLE	08	MAX	91	90	90	87	91	92	91	91	92	97	97	92	97	92	85	86	91	92	94	91	93	90	95	95	95	94	93	84	94	96	97	92.1
		MIN	60	60	61	61	62	66	72	70	70	72	74	71	64	66	71	69	71	68	67	64	67	73	76	75	71	72	74	67	71	66	71	68.5
NATCHITOCHES #2	07	MAX	89	85	86	85	87	89	88	89	89	93	95	89	93	88	83	86	88	90	92	89	89	89	95	94	93	91	90	80	88	92	94	89.3
		MIN	65	66	65	66	65	69	70	72	73	74	77	71	65	69	69	70	72	72	71	68	70	73	75	75	70	71	70	68	72	72	73	70.3
TOLEDO BEND LAKE	08	MAX	92	92	92	90	93	96	93	93	93	95	97	89	97	93	87		94	93	92	91	91	87	94	93	94	93	92	92	92	94	95	92.6
		MIN	64	64	64	66	66	69	70	72	72	73	74	76	71	70	70		72	71	69	68	70	72	74	75	73	73	74	68	67	69	72	70.3
**CENTRAL 05**																																		
ALEXANDRIA	08	MAX	88	93	91	92	93	96	91	92	94	95	96	92	98	94	89	91	92	94	92	93	93	89	94	95	93		92	87	93	94	95	92.7
		MIN	69	68	67	67	69	71	70	74	74	75	76	73	67	67	70	70	74	73	71	71	71	70	70	71	71		76	71	72	74	73	71.2
ALEXANDRIA 5 SSE	24	MAX	91	88	89	89	92	90	90	92	94	96	91	96	91	88	91	90	91	93	93	92	90	94	96	92	93	92	86	91	93	97	96	91.8
		MIN	63	65	64	66	67	69	73	72	72	74	73	73	65	68	69	73	75	72	69	69	71	76	76	73	72	72	74	71	73	71	78	70.9
BOYCE 3 WNW	24	MAX	86	85	87	88	90	89	90	90	94	94	87	94	89	85	89	88	91	90	90	91	88	94	94	92	89	90	82	87	92	94	93	89.7
		MIN	68	68	67	68	69	69	69	73	75	76	76	73	68	70	70	73	73	73	70	70	72	74	77	74	71	71	73	70	75	74	75	71.7
BUNKIE	08	MAX	91	88	88	89	90	91	89	89	92	93	93	90	94	90	86	87	88	90	89	90	91	87	92	94	91	92	92	90	90	93	94	90.4
		MIN	65	67	65	67	68	69	69	71	73	74	77	72	65	68	70	70	73	71	69	70	72	73	76	76	73	72	76	74	72	72	75	71.1
EUNICE	08	MAX	92	88	89	90	91	91	88	90	92	93	94	92	96	92	89	87	84	92	91	91	93	88	93	94	93	93	93	88	90	96	95	91.2
		MIN	67	67	67	68	71	69	73	70	72	73	76	72	70	70	72	71	73	71	69	70	72	74	74	74	74	74	76	71	75	72	72	71.6
GRAND COTEAU	17	MAX	90	88	89	90		89	85	93	91	91	91	94	93	89	86	87	91		90	89	84	91	91	91	91	91	87	89	93	92	94	90.0
		MIN	66	67	68	68		69	74	74	71	74	73	71	69	69	71	71	73		69	71	72	73	74	75	74	72	76	72	74	74	73	71.6
JENA 4 WSW	08	MAX	92	88	90	92	93	94	92	94	96	98	98	89	93	90	86	89	88	92	92	91	89	89	94	93	91	90	90	84	89	91	93	91.3
		MIN	62	62	61	63	62	65	68	72	71	72	74	69	61	65	67	68	72	68	67	68	69	71	73	74	69	68	72	65	70	71	72	68.1
JONESVILLE LOCKS	06	MAX	90	86	88	89	90	90	92	92	91	91	91	92	88	93	90	88	88	92	92	90	92	92	91	91	94	91	90	89	82	89	90	90.1
		MIN	67	67	68	68	68	68	69	69	73	73	76	76	69	68	68	68	72	71	69	68	70	71	74	74	77	71	71	70	70	69	75	70.5
LSU DEAN LEE RSCH STN	08	MAX	96							93							96	89	89					92	92	93					93	92		M
		MIN	67							65							67	71	73					69	76	77	70				71	70		M
NEW ROADS 5 NE	24	MAX	87	89	90	89	91	87	91	92	95	95	92	95	91	94	88	90	95	93	93	95	88	93	95	93	93	95	85	91	91	96	95	91.8
		MIN	69	71	70	69	72	70	74	71	73	76	73	72	71	71	71	73	73	73	72	73	75	75	75	75	75	71	73	72	74	75	76	72.7

The table's header reads "DAY OF MONTH" above the day columns 01–31.

LOUISIANA
201307

DAILY TEMPERATURES (°F)

STATION	OB.TIME	MAX/MIN	01	02	03	04	05	06	07	08	09	10	11	12	13	14	15	16	17	18	19	20	21	22	23	24	25	26	27	28	29	30	31	AVERAGE
**EAST CENTRAL 06**																																		
BATON ROUGE METRO AP	24	MAX	87	89	90	90	85	88	88	92	92	93	91	96	92	91	90	86	92	90	92	92	86	90	93	91	92	93	84	90	94	93	94	90.5
		MIN	68	67	69	68	71	70	73	71	72	76	73	71	69	70	70	72	74	73	71	73	74	76	76	75	73	69	74	71	73	76	77	72.1
BOGALUSA	08	MAX																																M
		MIN																																M
CLINTON 5 SE	08	MAX	91	86	85	87	87	86	87	87	88	90	90	90	91	88	92	81	84	90	82	90	85	90	89	90	89	86	89	90	90	92	90	88.1
		MIN	62	64	66	67	67	69	72	69	70	72	74	69	68	69	68	72	72	70	72	69	69	69	73	76	71	66	68	68	68	73	75	69.6
HAMMOND 5 E	08	MAX	91	87	87	88	89	78	86	86	88	90	91	91	93	90	87	86	90	90	88	90	90	83	87	93	91	92	93	81	89	93	92	88.7
		MIN	63	65	67	69	71	70	72	71	71	72	76	71	69	70	70	72	72	72	70	71	73	73	73	76	73	68	74	70	73	73	74	71.1
LSU BEN-HUR FARM	08	MAX	89	86	87	88	88	83	86	86	90	91	92	91	92	92	89	85	84	90	89	91	90	83	88	91	91	91	92	84	91	93	92	88.9
		MIN	68	66	68	69	74	71	74	72	72	75	76	70	70	72	72	72	74	72	71	72	74	74	74	77	74	70	76	71	73	75	75	72.4
SLIDELL	08	MAX	91	87	87	87	88	77	83	87	87	88	90	89	89	89	86	87	83	83	88	88	87	83	89	90	90	90	92	81	88	91	89	87.2
		MIN	68	73	71	72	73	72	74	73	72	75	79	69	72	71	72	73	73	71	71	72	75	74	76	79	74	73	75	72	74	76	76	73.2
SLIDELL AP	24	MAX	89	89	88	89	78	84	88	88	89	91	89	90	91	88	89	85	90	91	91	89	84	88	92	91	92	93	84	90	94	91	91	88.9
		MIN	66	71	72	73	73	73	74	73	70	74	71	71	71	71	72	73	74	72	70	72	74	75	78	79	74	72	74	74	75	75	75	72.9
**SOUTHWEST 07**																																		
CROWLEY 2 NE	08	MAX	92	87	89	90	91	90	88	87	92	94	94	94	95	91	90	85	87	91	89	91	93	87	95	95	95	95	92	89	88	94	93	91.1
		MIN	67	67	68	69	72	70	73	72	71	72	75	72	70	70	73	72	74	70	68	68	71	71	72	72	72	72	73	76	72	71	73	71.2
DE RIDDER	08	MAX	94	95	95	89	94	93	91	90	89	95	91	88	91	91	89	88	91	93	93	93	91	87	93	93	93	93	90	91	93	95	93	91.8
		MIN	67	73	72	69	68	69	68	69	70	70	70			70	71	71	70			68	69	73	71	69	71		71	69	73	72	70	70.1
HACKBERRY 8 SSW	08	MAX	97	87	90	91	92	91	88	92	90	90	90	92	91	92	89	88	85	88	92	89	86	84	90	90	90	92	91	92	86	89	90	89.8
		MIN	72	72	72	72	75	74	75	72	79	78	77	77	76	77	77	74	75	74	75	73	73	73	80	77	78	78	76	74	77	78	77	75.4
JENNINGS	08	MAX	94	87	90	91	92	93	91	88	92	95	95	94	93	95	90	84	87	92	89	92	92	86	92	92	92	93	93	91	88	95	94	91.4
		MIN	67	66	69	69	70	70	70	70	74	74	76	77	74	72	73	69	74	72	71	71	71	74	76	77	75	75	78	74	74	73	74	72.5
LAKE ARTHUR 10 SW	08	MAX	96	90	91	95	94	90	89	88	93	93	95	94	95	90	90	86	86	92	93	90	88	88	94	94	94	94	94	89	88	95	94	91.7
		MIN	70	69	68	70	71	72	74	75	76	76	76	75	73	73	77	74	75	72	72	73	76	80	79	77	78	75	76	76	76	76	76	74.4
LAKE CHARLES AP	24	MAX	89	92	92	94	96	93	90	92	95	94	95	94	96	91	90	89	93	94	91	91	87	92	93	93	96	94	93	88	93	94	95	92.5
		MIN	69	68	69	69	72	72	76	76	75	74	76	76	73	72	76	74	74	72	73	72	74	76	79	77	79	76	78	75	74	73	74	74.0
LELAND BOWMAN LOCK	08	MAX	94	87	90	89	91	86	85	85	87	88	93	90	93	94	88	88	88	88	90	88	88	86	91	91	91				88	92	91	89.3
		MIN	70	64	70	70	71	73	74	75	75	74	76	75	75	72	74	68	68	74	72	73	74	75	74	77	76				73	75	74	72.9
MOSS BLUFF 2 NNW	24	MAX	88	90	91	92	93	91	90	94	94	96	93	97	95	89	88	89	93	94	90	93	89	95	95	94	94	95	90	90	96	96	97	92.6
		MIN	63	63	63	63	66	70	75	74	73	73	74	73	69	68	75	72	72	69	70	70	72	75	78	75	75	74	77	74	71	68	70	71.1
OBERLIN FIRE TWR	09	MAX	92	87	88	89	90	92	91	89	90	94	94	90	95	92	88	83	90	92	92	92	90	86	91	92	91	91	92	86	90	94	93	90.5
		MIN	65	66	66	66	67	69	69	72	72	73	75	73	69	69	71	71	74	71	69	68	70	73	74	75	75	74	77	72	72	70	71	70.9
ROCKEFELLER WL REFUGE	08	MAX	92	87	89	91	91	85	85	85	87	87	87	87	88	91	88	84	82	88	90	87	86	83	87	87	87	89	87	87	86	89	88	87.3
		MIN	71	70	70	71	72	71	71	75	76	76	80	77	72	72	73	73	74	73	71	73	73	76	80	79	79	77	77	73	77	77	77	74.4
VINTON 5W	08	MAX	94	89	91	91	91	94	94	86	90	92	94	93	97	96	90	91	89	91	94	89	89	86	92		93	93	95	91	89	95	96	91.8
		MIN	66	66	66	66	67	70	74	72	72	72	73	74	73	68	73	72	73	70	71	69	72	74	74		74	73	78	74	71	70	71	71.3
**SOUTH CENTRAL 08**																																		
CARVILLE 2 SW	24	MAX	86	88	90	89	87	87	90	89	91	92	91	91	90	89	84	83	89	86	89	88	89	89	91	90	90	90	82	89	90	91	92	88.8
		MIN	72	71	70	69	72	71	73	74	75	76	74	74	73	73	74	73	75	71	73	74	73	75	77	77	76	74	75	74	76	76	78	73.8
DONALDSONVILLE 4 SW	08	MAX	91	86	87	87	89	81	86	85	89	91	90	87	90	90	88	88	85	89	87	89	90	82	89	90	90	89	91	82	89	91	91	88.0

LOUISIANA
201307

DAILY TEMPERATURES (°F)

STATION	OB.TIME	MAX/MIN	01	02	03	04	05	06	07	08	09	10	11	12	13	14	15	16	17	18	19	20	21	22	23	24	25	26	27	28	29	30	31	AVERAGE
		MIN	67	68	70	71	72	71	72	71	72	73	74	71	69	70	72	73	74	72	71	72	73	73	74	74	75	72	74	72	73	74	75	72.1
FRANKLIN 3 NW	24	MAX	91	86	85	88	84	85	83	89	89	89	88	90	90	87	84	84	89	87	90	87	83	88	88	89	89	89	89	87		91	89	87.6
		MIN	71	69	71	71	73	73	74	73	73	74	73	72	71	73	74	74	74	72	72	73	73	76	78	77	71	74	75	75		75	76	73.3
JEANERETTE 5 NW	08	MAX	91	86	86	87	89	88	85	81	90	90	90	91	92	90	87	85	85	89	88	90	90	90	89	91	92	89	91	85	89	93	92	88.7
		MIN	69	68	69	69	70	72	74	72	73	74	74	72	71	71	73	72	73	71	71	71	72	72	75	75	74	72	75	74	74	74	75	72.3
LAFAYETTE	22	MAX	88	88	89	90	92	90	87	85	92	92	92	92	93	92	90	85	86	91	91	90	91	87	92	93	94	92	94	88	93	93	94	90.5
		MIN	71	69	68	68	71	71	71	74	75	74	75	74	73	72	72	74	75	73	73	74	75	75	77	77	76	76	76	75	75	76	76	73.6
LAFAYETTE FCWOS	24	MAX	89	90	92	92	90	86	84	90	91	93	93	95	91	88	86	86	90	89	90	90	86	92	92	93	92	92	88	89	94	93	95	90.4
		MIN	70	69	69	71	72	71	74	73	74	76	74	73	73	72	72	74	75	73	72	74	75	76	76	77	75	74	77	74	77	75	77	73.7
MORGAN CITY	08	MAX	92	85	86	91		88	85	84	89	90	87	88	92	91	89	89	81	89	89	89	90	82	94	95	88	91	91	87	90	87	90	88.6
		MIN	74	75	75	72		73	73	71	71	73	75	74	72	72	74	75	74	74	73	74	74	77	76	76	77	77	77	75	75	75	75	74.3
NEW IBERIA AP ACADIANA RGNL	24	MAX	89	91	92	93	91	87	85	91	93	92	94	96	93	88	88	87	92	92	92	91	87	93	93	94	93	93	88	90	95	93	94	91.3
		MIN	71	71	71	71	74	74	75	74	76	76	75	75	74	74	72	76	77	72	73	75	75	77	76	78	76	76	78	76	77	76	77	74.8
ST MARTINVILLE 3 SW	08	MAX	91	86	88	88	89	90	85	81	90	92	92	92	94	91	85	86	85	89	89	90	90	85	91	91	91	90	92	85	89	92	93	89.1
		MIN	67	67	68	68	71	71	71	71	72	73	71	71	70	70	73	73	72	70	70	72	74	77	75	75	75	72	74	73	74	74	76	71.9
**SOUTHEAST 09**																																		
BOOTHVILLE ASOS	24	MAX	87	87	85	86	82	81	84	83	87	87	83	87	90	86	86	86	86	87	86	85	84	87	87	88	88	89	86	88	90	89	89	86.3
		MIN	78	78	72	77	73	74	77	78	78	77	74	76	76	75	75	76	79	80	79	77	73	79	82	81	75	78	76	75	78	79	81	77.0
GALLIANO	08	MAX	92	90	86	85	86	78	82	85	85	87	89	88	90	89	87	87	86	87	88	89	87	84	86	90	90	89	91	85	92	90	91	87.5
		MIN	71	72	72	74	76	71	76	75	74	75	77	73	73	75	75	77	78	75	75	76	75	74	76	80	81	74	80	76	76	78	77	75.4
HOUMA	08	MAX	91	87		86	84	84	85	84	86	88	90	89	92	90	86	83	82	87	87	88	87	83	89	91	90	90	91	91	89	88	91	87.6
		MIN	69	69		72	73	72	73	72	70	73	75	71	70	72	72	73	74	71	71	72	74	72	74	78	76	71	76	74	76	75	74	72.8
MARRERO 9 SSW	08	MAX	93	89															84	89	91			91	91	92							93	M
		MIN		71															74	73	72			72	77	77							74	M
NEW ORLEANS AP	24	MAX	88	88	86	87	81	84	87	88	90	93	88	93	91	88	90	86	90	90	91	90	82	91	92	92	91	92	83	90	93	93	93	89.1
		MIN	74	77	75	77	73	74	73	75	74	76	73	73	75	76	76	78	78	75	74	75	73	78	78	77	78	77	76	75	75	78	78	75.6
NEW ORLEANS AUDUBON	24	MAX	89	91	87	87	80	84	87	87	93	93	88	90	93	89	90	87	90	91	91	93	85	94	93	93	90	93	85	89	96			89.6
		MIN	75	75	73	74	72	73	73	73	73	76	72	73	75	76	78	76	78	72	74	75	72	78	78	75	78	75	75	75	75			74.7
NEW ORLEANS LKFRNT AP	24	MAX	87	90	88	88	82	85	87	88	91	92	88	92	92	88	89	86	90	91	91	90	83	91	92	91	90	93	85	91	94	91	91	89.3
		MIN	79	75	75	77	75	76	76	76	77	81	74	76	80	75	78	78	79	80	78	78	76	79	80	80	80	81	79	79	80	81	81	78.0
TERRYTOWN 3S	07	MAX	94	90	90	87	89	79	84	87	88	91	93	89	93	92	89	89	86	87	90	89	88	83	91	93	93	93	94	85	91	93	94	89.5
		MIN	73	75	73	74	72	74	75	74	74	76	77	71	74	75	75	77	76	74	74	75	76	77	77	76	78	76	73	75	75	77	77	75.0
THIBODAUX 4 SE	08	MAX	93	88	87	87	88	77	86	86	87	90	90	89	91	91	91	84	85	89	89	89	88	82	89	90	90	90	93	82	90	91	92	88.2
		MIN	68	69	70	71	72	71	74	72	70	72	76	72	70	72	73	72	74	71	70	72	74	71	74	75	74	71	75	73	74	75	75	72.3

TOTAL SNOWFALL AND NUMBER OF DAYS WITH ONE INCH OR MORE ON GROUND

LOUISIANA
201307

STATION	JUL		AUG		SEP		OCT		NOV		DEC		JAN		FEB		MAR		APR		MAY		JUN		TOTAL		TOTAL PRECIPITATION
	SNOWFALL	1 IN ON GD	SNOWFALL	1 IN ON GD	SNOWFALL	1 IN ON GD	SNOWFALL	1 IN ON GD	SNOWFALL	1 IN ON GD	SNOWFALL	1 IN ON GD	SNOWFALL	1 IN ON GD	SNOWFALL	1 IN ON GD	SNOWFALL	1 IN ON GD	SNOWFALL	1 IN ON GD	SNOWFALL	1 IN ON GD	SNOWFALL	1 IN ON GD	SNOWFALL	1 IN ON GD	
**LOUISIANA**																											
**NORTHWEST 01**																											
BENTON 5E													M 0.0												M 0.0	M 0	M 41.94
MANSFIELD					M 0.0																				M 0.0	M 0	M 30.53
MINDEN	M 0.0																								M 0.0	M 0	M 53.35
MOORINGSPORT 1 N	M 0.0		M 0.0		M 0.0		M 0.0		M 0.0		M 0.0		M 0.0		M 0.0		M 0.0		M 0.0		M 0.0		M 0.0		M 0.0	M 0	M 37.12
RED RIVER RSCH STN							M 0.0				M 0.0								M 0.0		M 0.0				M 0.0	0	M 48.60
ROBSON																									0.0	0	6.93
SHREVEPORT DWTN AP																									0.0	0	43.98
SHREVEPORT AP	M 0.0										T		0.1				T								M 0.1	0	51.94
SHREVEPORT STHRN HILLS											T		M 0.0		M 0.0		M 0.0								M 0.0	M 0	52.65
SHREVEPORT WFO	M 0.0										T		0.1				T		T		M T				M 0.1	M 0	55.40
**NORTH CENTRAL 02**																											
BIENVILLE 3 NE																									0.0	0	M 55.46
CALHOUN RSCH STN																									0.0	0	M 56.38
COLUMBIA LOCK					M 0.0		M 0.0										M 0.0				M 0.0		M 0.0		M 0.0	M 0	M 60.72
FARMERVILLE	M 0.0		M 0.0										T								M 0.0				M 0.0	M 0	M 53.35
HOMER 1N	M 0.0		M 0.0								M T														M 0.0	M 0	M 30.17
MONROE REGIONAL AP	M 0.0						M 0.0						0.3												M 0.3	0	59.48
RUSTON LA TECH							M 0.0				M 0.0		M 0.0				M 0.0						M 0.0		M 0.0	M 0	M 41.40
WINNFIELD 3 N																									0.0	0	0.00
**NORTHEAST 03**																											
BASTROP																									0.0	0	M 51.59
LAKE PROVIDENCE			M 0.0										T												M 0.0	M 0	74.41
RAYVILLE																									0.0	0	54.59
ST JOSEPH 3 N					M 0.0																				M 0.0	M 0	M 38.69
TALLULAH													0.4												0.4	0	M 54.70
TALLULAH VICKSBURG RGN	M 0.0												M 0.0												M 0.0	0	67.00
WINNSBORO 2 SE			M 0.0																						M 0.0	M 0	M 16.43
WINNSBORO 5 SSE	M 0.0																								M 0.0	M 0	M 46.91
**WEST CENTRAL 04**																											
HODGES GARDENS																									0.0	0	M 50.21
LEESVILLE																									0.0	0	M 57.51
NATCHITOCHES #2																									0.0	0	51.06
TOLEDO BEND LAKE							M 0.0				M 0.0								M 0.0						M 0.0	M 0	M 47.84
**CENTRAL 05**																											
ALEXANDRIA	M 0.0		M 0.0		M 0.0		M 0.0		M 0.0		M 0.0		M 0.0		M 0.0				M 0.0		M 0.0				M 0.0	M 0	M 56.81
ALEXANDRIA 5 SSE	M 0.0		M 0.0		M 0.0		M 0.0		M 0.0		M 0.0		M 0.0		M 0.0		M 0.0		M 0.0		M 0.0		M 0.0		M 0.0	M 0	M 53.53
BOYCE 3 WNW	M 0.0		M 0.0		M 0.0		M 0.0		M 0.0		M 0.0		M 0.0		M 0.0		M 0.0		M 0.0		M 0.0		M 0.0		M 0.0	M 0	M 59.86
BUNKIE																									0.0	0	M 62.16
EUNICE																									0.0	0	M 76.44
GRAND COTEAU																									0.0	0	M 66.61
JENA 4 WSW																									0.0	0	60.10
JONESVILLE LOCKS																									0.0	0	M 65.76
LARTO LAKE																									0.0	0	M 8.55
LSU DEAN LEE RSCH STN																									0.0	0	0.00
NEW ROADS 5 NE																									0.0	0	84.00
**EAST CENTRAL 06**																											
BATON ROUGE METRO AP	M 0.0												M 0.0						M 0.0						M 0.0	0	77.19

TOTAL SNOWFALL AND NUMBER OF DAYS WITH ONE INCH OR MORE ON GROUND

LOUISIANA
201307

STATION	JUL		AUG		SEP		OCT		NOV		DEC		JAN		FEB		MAR		APR		MAY		JUN		TOTAL		TOTAL PRECIPITATION
	SNOWFALL	1 IN ON GD	SNOWFALL	1 IN ON GD	SNOWFALL	1 IN ON GD	SNOWFALL	1 IN ON GD	SNOWFALL	1 IN ON GD	SNOWFALL	1 IN ON GD	SNOWFALL	1 IN ON GD	SNOWFALL	1 IN ON GD	SNOWFALL	1 IN ON GD	SNOWFALL	1 IN ON GD	SNOWFALL	1 IN ON GD	SNOWFALL	1 IN ON GD	SNOWFALL	1 IN ON GD	
BOGALUSA																									0.0	0	0.00
CLINTON 5 SE																									0.0	0	40.34
HAMMOND 5 E																									0.0	0	55.85
LSU BEN-HUR FARM																									0.0	0	84.00
SLIDELL																									0.0	0	M 78.91
SLIDELL AP																									0.0	0	75.97
**SOUTHWEST 07**																											
CROWLEY 2 NE																									0.0	0	81.59
DE RIDDER																									0.0	0	M 50.03
HACKBERRY 8 SSW																									0.0	0	M 62.31
JENNINGS			M 0.0																						M 0.0	M 0	90.65
LAKE ARTHUR 10 SW																									0.0	0	71.16
LAKE CHARLES AP	M 0.0								M 0.0		M 0.0		M 0.0		M 0.0		M 0.0		M 0.0		M 0.0		M 0.0		M 0.0	M 0	70.21
LELAND BOWMAN LOCK	M 0.0		M 0.0		M 0.0		M 0.0		M 0.0		M 0.0		M 0.0		M 0.0		M 0.0		M 0.0		M 0.0		M 0.0		M 0.0	M 0	M 67.03
MOSS BLUFF 2 NNW																									0.0	0	54.83
OBERLIN FIRE TWR																									0.0	0	66.22
ROCKEFELLER WL REFUGE																									0.0	0	70.90
VINTON 5W																									0.0	0	M 62.89
**SOUTH CENTRAL 08**																											
CARVILLE 2 SW																									0.0	0	82.06
DONALDSONVILLE 4 SW	M 0.0		M 0.0		M 0.0		M 0.0		M 0.0		M 0.0		M 0.0		M 0.0		M 0.0		M 0.0		M 0.0		M 0.0		M 0.0	0	71.32
FRANKLIN 3 NW																									0.0	0	M 74.26
JEANERETTE 5 NW																									0.0	0	M 82.61
LAFAYETTE							M 0.0										M 0.0		M 0.0		M 0.0				M 0.0	M 0	M 84.40
LAFAYETTE FCWOS	M 0.0								M 0.0		M 0.0		M 0.0		M 0.0		M 0.0		M 0.0		M 0.0				M 0.0	M 0	69.74
MORGAN CITY																									0.0	0	M 70.13
NEW IBERIA AP ACADIANA RGNL	M 0.0						M 0.0		M 0.0		M 0.0		M 0.0		M 0.0		M 0.0		M 0.0		M 0.0				M 0.0	0	71.35
ST MARTINVILLE 3 SW																									0.0	0	M 69.00
**SOUTHEAST 09**																											
BOOTHVILLE ASOS																									0.0	0	M 59.94
GALLIANO																									0.0	0	M 75.30
HOUMA																									0.0	0	M 104.56
MARRERO 9 SSW																									0.0	0	M 12.85
NEW ORLEANS AP	M 0.0												M 0.0												M 0.0	0	80.36
NEW ORLEANS AUDUBON																									0.0	0	M 74.28
NEW ORLEANS LKFRNT AP																									0.0	0	86.12
TERRYTOWN 3S	M 0.0		M 0.0		M 0.0		M 0.0		M 0.0		M 0.0		M 0.0		M 0.0		M 0.0		M 0.0		M 0.0		M 0.0		M 0.0	0	76.27
THIBODAUX 4 SE																									0.0	0	M 57.27

MONTHLY AND SEASONAL HEATING DEGREE DAYS

STATION	JUL	AUG	SEP	OCT	NOV	DEC	JAN	FEB	MAR	APR	MAY	JUN	TOTAL	SEASONAL NORM
**LOUISIANA**														
**NORTHWEST 01**														
BENTON 5E	0	0	0	151	390	511	563E	499	461	189E	53	0	2817E	
MANSFIELD	0	0	0	118E	282E	465E	665E						M 1530E	
MINDEN	0	0	0	148	333E	465E	557	439	400	161	58	0	2561E	2505
MOORINGSPORT 1 N	0	0	0	144	335E	456	522E	418	380	150	42	0	2447E	
RED RIVER RSCH STN	0	0	0	121E	285	417	504	388	345	119E	39E	0	2218E	2452
SHREVEPORT DWTN AP	0	0	2	114	280	403	510	403	337	153	43	0	2245	
SHREVEPORT AP	0	0	2	100	258	393	485	376	306	123	38	0	2081	2251
SHREVEPORT STHRN HILLS	0	0	0	136	339	455	528	414	351	153	49	0	2425	
SHREVEPORT WFO	0	0	2	98	254	386	476	365	308	129	38	0	2056	
**NORTH CENTRAL 02**														
BIENVILLE 3 NE	0	0	0	125	302	382E	481E	370E	291	120	40	0	2111E	2419
CALHOUN RSCH STN	0	0				577E	561E	440E	423E	168	68E	0	M 2237E	2334
COLUMBIA LOCK	0	0	0	141	317	419E	507	389E	378	152	56	0	2359E	
FARMERVILLE	0	0	0	165E	340	420	552E	432	396	149	55	0	2509E	
HOMER 1N	0	0	0	172	389	495E	583E	479	488E	187E	75E	0	2868E	2672
MONROE REGIONAL AP	0	0	1	108	321	375	477	380	365	137	41	0	2205	2190
RUSTON LA TECH	0	0	1	109E	330	184E			496E	146E	54E	0	M 1320E	2517
**NORTHEAST 03**														
BASTROP	0		5E	172	407	464	568	487E	469	188	71E	0	M 2831E	2487
LAKE PROVIDENCE	0	0	0	128	354	407	524	455	431	154	54	0	2507	2584
RAYVILLE	0	0	0	98E	309E	410E	464	382	349	124	45	0	2181E	
ST JOSEPH 3 N	0	0	8E	103E	386	216E	376E	304E	326E	83E	33E	0	1835E	2163
TALLULAH	0	0	0	113E	322	312E	477E	410E	397E	99E	39E	0	2169E	2426
TALLULAH VICKSBURG RGN	0	0	4	151	409	379	450	383	386	124	52	0	2338	
WINNSBORO 2 SE	0	0	1	120	335	381	474	390	358	127	46	0	2232	
WINNSBORO 5 SSE	0	0	2	122E	330E	414	499	376	381	134	42	0	2300E	2357
**WEST CENTRAL 04**														
HODGES GARDENS	0	0	0	102	254	381	459	370	326	133E	44	0	2069E	
LEESVILLE	0	0	0	109	290E	354	450E	328	288	123	48	0	1990E	2048
NATCHITOCHES #2	0	0	0	105	259	360	428	355	332	135	43	0	2017	
TOLEDO BEND LAKE	0	0	0	80E	225	308	427	328	300	106E	41	0	1815E	
**CENTRAL 05**														
ALEXANDRIA	0	0	0	86E	231	335	415	323	357E	100	33	0	1880E	
ALEXANDRIA 5 SSE	0	0	0	90	280	348	417	324	299	106	38	0	1902	
BOYCE 3 WNW	0	0	0	90E	236	319	396	328	287E	109	36E	0	1801E	1961
BUNKIE	0	0	0	91	269	305E	395	294	314E	103	24	0	1795E	1882
EUNICE	0	0	0	71	212	305	394	261	288E	98	31	0	1660E	1640
GRAND COTEAU	0	0	0	58E	212	226E	341E	213E	259	77E	27E	0	1413E	1485
JENA 4 WSW	0	0	1	115	312	384E	481	380	361	138	0	0	2172E	2291
JONESVILLE LOCKS	0	0	0	96	274	341	403	335E	287	101	32	0	1869E	2146
LSU DEAN LEE RSCH STN	0	0	0	87E	270E	344E			289E	0	14E	0	M 1004E	2062
NEW ROADS 5 NE	0	0	0	72	211	304	355	269	267	74	32	0	1584	1777
**EAST CENTRAL 06**														
BATON ROUGE METRO AP	0	0	0	67	230	280	341	254	276	73	30	0	1551	1689
CLINTON 5 SE	0	0	0	105	283	270	354E	297	355	105	33	0	1802E	
HAMMOND 5 E			0	85	302	327	370	320	355	88	35	0	M 1882	1790
LSU BEN-HUR FARM	0	0	0	67	231	300	357	283	316	76	32	0	1662	1730
SLIDELL	0	0	0	67	228	275	324	263	302	76E	26	0	1561E	1652
SLIDELL AP	0	0	0	69	265	290	324	250	303	58E	30	0	1589E	
**SOUTHWEST 07**														
CROWLEY 2 NE	0	0	0	62	214	290	349	246	279	83	30	0	1553	1663
DE RIDDER	0	0	0	83E	208E	320E	392	278	293E	93	17E	0	1684E	1847
HACKBERRY 8 SSW	0	0	0	48	158	257	369	192	260E	71	21	0	1376E	1508
JENNINGS	0	0	0	58	192	274	364	238	261	77	29	0	1493	1732
LAKE ARTHUR 10 SW	0	0	0	54	168	254E	310	160	241	52	13	0	1252E	1735
LAKE CHARLES AP	0	0	0	42	164	249	327	187	213	61	24	0	1267	1546
LELAND BOWMAN LOCK	0	0	0	52E	187	268	298	205E	252E	73E	27E	0	1362E	1567
MOSS BLUFF 2 NNW	0	0	0	71	236	292	356	238	254	86	30	0	1563	
OBERLIN FIRE TWR	0	0	0	88	245	322	408	293	300	126E	38	0	1820E	1609
ROCKEFELLER WL REFUGE	0	0	0	58	196	276	393E	219	247	82	27	0	1498E	1521
VINTON 5W	0	0	0	64E	201E	288E	375E	236E	254E	83E	29	0	1530E	
**SOUTH CENTRAL 08**														
CARVILLE 2 SW	0	0	0	51	175	251	334	225	242	65	24	0	1367	1599
DONALDSONVILLE 4 SW	0	0	0	66	232	271	329	252	301	73	32	0	1556	1583
FRANKLIN 3 NW	0	0	0	57	194	246	323	221	255	46	101E	0	1443E	1522
JEANERETTE 5 NW	0	0	0	65	217	280	333	244E	281	74	31	0	1525E	1628
LAFAYETTE	0	0	0	50E	189E	274	286E	228E	274E	66E	23E	0	1390E	1531
LAFAYETTE FCWOS	0	0	0	47	186	257	318	202	235	60	27	0	1332	1493
MORGAN CITY	0	0	0	35	193E	307	321E	232E	271E	32E	34E	0	1425E	1477
NEW IBERIA AP ACADIANA RGNL	0	0	0	42	159	234	281	171	202	40	21	0	1150	1544
ST MARTINVILLE 3 SW	0	0	0	72E	212	282	325	231	277	80E	31	0	1510E	
**SOUTHEAST 09**														
BOOTHVILLE ASOS	0	0	0	24	110	160	213	184E	213E	35	11	0	950E	1149
GALLIANO	0	0	0	34E	199E	225	262E	201	278	55E	18E	0	1272E	1359

MONTHLY AND SEASONAL HEATING DEGREE DAYS

STATION	JUL	AUG	SEP	OCT	NOV	DEC	JAN	FEB	MAR	APR	MAY	JUN	TOTAL	SEASONAL NORM
HOUMA	0	0	0	62E	246	264	340E	249E	291E	74E	35E	0	1561E	1346
MARRERO 9 SSW	0	0	0	14E	154E	256E	355E	233E	253E	50E	22E	0	1337E	
NEW ORLEANS AP	0	0	0	27	162	219	277	204	209	32	16	0	1146	1417
NEW ORLEANS AUDUBON	0	0	0	38E	153E	165E	269	188	203E	30	17E	0	1063E	1208
NEW ORLEANS LKFRNT AP	0	0	0	31	137	153	206	110	127	10	9	0	783	
TERRYTOWN 3S	0	0	0	35	165	230	275	216	246	40	19	0	1226	
THIBODAUX 4 SE	0	0	0	58	225	244E	309	232E	280	62	29	0	1439E	1433

LOUISIANA
201307

DAILY SOIL TEMPERATURES

STATION	DEPTH	TIME	DAY OF MONTH																															AVERAGE
			01	02	03	04	05	06	07	08	09	10	11	12	13	14	15	16	17	18	19	20	21	22	23	24	25	26	27	28	29	30	31	
**LOUISIANA**																																		
**NORTHWEST 01**																																		
RED RIVER RSCH STN (IN)																																		
BARE GROUND	4	MAX	85	84	84	83	84	83	83	84	86	85	85	86	87	85	83	83	85	85	85	84	85	85	85	86	85	85	84	83	-	85	85	84.6
	4	MIN	82	83	81	81	82	82	81	81	82	82	83	83	83	82	82	81	82	82	82	82	82	83	82	83	83	83	82	81	-	82	82	82.1
**NORTH CENTRAL 02**																																		
CALHOUN RSCH STN (IN)																																		
SOD	4	MAX	85	84	84	-	85	86	87	86	87	89	90	89	87	86	84	88	87	88	88	88	86	88	89	89	88	88	86	82	85	87	88	86.8
	4	MIN	77	76	75	-	72	76	76	80	80	73	77	78	77	75	77	75	78	80	80	77	75	83	80	81	82	78	80	78	78	77	81	77.7
**NORTHEAST 03**																																		
ST JOSEPH 3 N (IN)																																		
BARE GROUND	2	MAX	105	103	106	107	-	107	107	109	100	105	108	98	96	92	92	96	96	109	96	103	108	106	-	86	-	-	80	89	96	98	79 I	99.9
	2	MIN	81	78	80	78	-	82	86	81	81	81	82	78	75	75	75	75	78	80	78	78	85	84	-	84	-	-	77	76	76	79	77	79.3
WINNSBORO 2 SE (IN)																																		
BARE GROUND	2	MAX	86	86	85	85	85	87	85	88	87	88	94	90	88	85	85	84	86	89	89	88	90	88	90	91	86	86	86	83	88	88	89	87.3
	2	MIN	74	74	73	74	73	74	76	77	78	78	83	79	74	74	74	76	77	77	76	77	77	78	78	80	77	75	76	75	76	77	78	76.3
WINNSBORO 5 SSE (IN)																																		
BARE GROUND	4	MAX	84	86	84	88	89	91	90	90	87	88	88	82	86	85	84	83	81	88	86	87	87	87	84	87	88	89	87	86	85	88	89	86.6
	4	MIN	76	75	74	76	76	76	77	80	79	80	79	77	75	75	75	74	75	77	77	76	78	79	78	78	77	77	76	77	75	79	78	76.8
**CENTRAL 05**																																		
LSU DEAN LEE RSCH STN (IN)																																		
**EAST CENTRAL 06**																																		
CLINTON 5 SE (IN)																																		
HAMMOND 5 E (IN)																																		
**SOUTHWEST 07**																																		
CROWLEY 2 NE (IN)																																		
JENNINGS (IN)																																		

LOUISIANA
201307

SOILS REFERENCE NOTES

STATION	SOIL TYPE	SOIL COVER	SLOPE	UNITS
RED RIVER RSCH STN	SANDY LOAM	BARE GROUND	00	F
CALHOUN RSCH STN	FINE SANDY LOAM	BARE GROUND	00	F
ST JOSEPH 3 N	SHARKEY CLAY	BARE GROUND	LEVEL	F
WINNSBORO 2 SE	SANDY LOAM	BARE GROUND	0	F
WINNSBORO 5 SSE	SANDY LOAM	BARE GROUND	00	F
LSU DEAN LEE RSCH STN	SANDY	BARE GROUND	00	F
CLINTON 5 SE	FINE SANDY LOAM	BARE GROUND	LEVEL NNW	F
HAMMOND 5 E	SANDY LOAM	BARE GROUND	00	F
CROWLEY 2 NE	SAND	SOD	1 DEG S	F
JENNINGS	SILT CLAY LOAM	SOD	0	F

LOUISIANA
201307

SNOWFALL AND SNOW ON GROUND (INCHES)

STATION		DAY OF MONTH																												
		01	02	03	04	05	06	07	08	09	10	11	12	13	14	15	16	17	18	19	20	21	22	23	24	25	26	27	28	29	30	31
**LOUISIANA NORTHWEST 01**																																
BENTON 5E	SNOWFALL																				-	-	-	-	-	-						
	SN ON GND																				-	-	-	-	-	-						
HOSSTON	SNOWFALL																															
JAMESTOWN	SNOWFALL																															
KEITHVILLE	SNOWFALL																															
	SN ON GND																															
KORAN	SNOWFALL																															
LOGANSPORT	SNOWFALL																															
	SN ON GND																															
MANSFIELD 7 NW	SNOWFALL																															
MINDEN	SNOWFALL																															
	SN ON GND																															
MOORINGSPORT 1 N	SNOWFALL																															
	SN ON GND																															
RED RIVER RSCH STN	SNOWFALL																															
SHREVEPORT DWTN	SNOWFALL																															
	SN ON GND																															
SHREVEPORT DWTN AP	SNOWFALL																															
SHREVEPORT AP	SNOWFALL																															
	SN ON GND																															
SHREVEPORT STHRN HILLS	SNOWFALL																															
	SN ON GND																															
SHREVEPORT WFO	SNOWFALL																															
	SN ON GND																															
SPRINGHILL	SNOWFALL																															
VIVIAN	SNOWFALL																															

Snowfall: Includes snow and ice. Values for NWS stations (J index note) are Mid-Mid (LST).
Snow on ground: Includes snow, sleet, ice, and hail. Values for NWS stations (J index note) are observed at 12 UTC (GMT).
Water Equivalent: Given for NWS stations (J index note) only, when snow depth is 2 inches or more, and is measured at 18 UTC (GMT)

LOUISIANA
201307

PAN EVAPORATION AND WIND

STATION		01	02	03	04	05	06	07	08	09	10	11	12	13	14	15	16	17	18	19	20	21	22	23	24	25	26	27	28	29	30	31	TOTAL OR AVERAGE
LOUISIANA																																	
NORTHWEST 01																																	
RED RIVER RSCH STN	WIND	22	20	20	39	24	29	53	58	44	39	31	38	25	34	45	31	60	49	30	31	45	56	74	70	36	38	43	22	-	52	54	1252E
	EVAP	0.34	0.22	0.26	0.30	0.27	0.00	0.00	0.00	0.79	0.33	0.25	0.21	0.29	0.29	0.14	0.10	0.29	0.28	0.31	0.26	0.29	0.25	0.30	0.28	0.23	0.25	0.00	0.00	-	0.30	0.33	7.40E
	MAX	-	-	-	-	-	-	-	-	-	-	-	-	-	-	-	-	-	-	-	-	-	-	-	-	-	-	-	-	-	-	-	M
	MIN	-	-	-	-	-	-	-	-	-	-	-	-	-	-	-	-	-	-	-	-	-	-	-	-	-	-	-	-	-	-	-	M
NORTH CENTRAL 02																																	
CALHOUN RSCH STN	WIND	-	-	-	-	-	-	-	-	-	-	-	-	-	-	-	-	-	-	-	-	-	-	-	-	-	-	-	-	-	-	-	M
	EVAP	-	-	-	-	-	-	-	-	-	-	-	-	-	-	-	-	-	-	-	-	-	-	-	-	-	-	-	-	-	-	-	M
	MAX	-	-	-	-	-	-	-	-	-	-	-	-	-	-	-	-	-	-	-	-	-	-	-	-	-	-	-	-	-	-	-	-
	MIN	-	-	-	-	-	-	-	-	-	-	-	-	-	-	-	-	-	-	-	-	-	-	-	-	-	-	-	-	-	-	-	M
NORTHEAST 03																																	
ST JOSEPH 3 N	WIND	-	9	2	-	-	-	-	-	7	0	26	19	-	-	-	4	4	2	5	-	-	-	71	41	-	-	-	-	-	5	13	M
	EVAP	0.00	0.27	0.21	0.00	-	0.00	0.00	0.00	0.22	0.00	0.00	0.00	0.00	0.00	0.00	0.00	0.00	0.00	0.00	0.00	0.00	0.00	0.25	0.25	-	0.00	0.00	0.00	0.00	0.28	0.20	1.80E
	MAX	-	-	-	-	-	-	-	-	-	-	-	-	-	-	-	-	-	-	-	-	-	-	-	-	-	-	-	-	-	-	-	-
	MIN	-	-	-	-	-	-	-	-	-	-	-	-	-	-	-	-	-	-	-	-	-	-	-	-	-	-	-	-	-	-	-	M
WEST CENTRAL 04																																	
TOLEDO BEND LAKE	WIND	45	39	40	20	41	60	76	114	58	56	63	44	40	50	52	-	72	58	44	37	52	69	95	83	42	44	112	41	56	85	85	1832E
	EVAP	0.42	0.36	0.32	0.20	0.34	0.34	0.36	0.29	0.24	0.32	0.38	0.22	0.33	0.36	0.18	-	0.40	0.31	0.24	0.25	0.32	0.11	0.35	0.35	0.20	0.33	0.33	0.24	0.27	0.33	0.43	9.42E
	MAX	-	-	-	-	-	-	-	-	-	-	-	-	-	-	-	-	-	-	-	-	-	-	-	-	-	-	-	-	-	-	-	-
	MIN	-	-	-	-	-	-	-	-	-	-	-	-	-	-	-	-	-	-	-	-	-	-	-	-	-	-	-	-	-	-	-	M
SOUTHWEST 07																																	
JENNINGS	WIND	31	27	26	39	35	53	41	55	35	45	37	34	45	55	41	94	54	41	29	20	40	70	92	94	89	30	69	68	47	45	50	1531
	EVAP	0.30	0.24	0.25	0.31	0.31	0.29	0.29	0.24	0.30	0.27	0.31	0.28	0.30	0.33	0.20	0.16	0.15	0.20	0.22	0.21	0.24	0.21	0.32	0.36	0.27	0.09	0.34	0.26	0.18	0.32	0.29	8.04
	MAX	101	94	99	98	99	97	93	96	99	99	101	101	97	104	93	87	88	96	94	99	98	89	96	96	95	100	96	95	94	103	101	101
	MIN	70	68	67	68	70	70	74	75	76	77	77	77	76	72	71	72	72	72	72	72	72	73	74	74	74	74	75	75	75	73	73	72.9

Evaporation: Is measured in hundreths of inches.
Wind: Is measured in miles.
Max and Min: The maximum and minimum tempertures (Fahrenheit) of the water in the evaporation pan.

STATION INDEX

STATION	INDEX NO.	DIVISION	COUNTY	LATITUDE	LONGITUDE	ELEVATION (IN FEET)	OBSERVATION TIME AND TABLES LOCAL STD TIME TEMP	PRECIP	EVAP	SPECIAL SEE (NOTES)
LOUISIANA										
ABBEVILLE	0007	07	VERMILION	29 58	92 7W	10		08		H
ABITA RVR COVINGTON	0012	06	ST. TAMMANY	30 28	90 6W	3		07		H
ABITA SPRING FIRE TWR	0021	06	ST. TAMMANY	30 26	90 3W	30		13		H
ABITA SPRINGS 1 SW	0016	06	ST. TAMMANY	30 28	90 3W	25		07		H
ALEXANDRIA	0098	05	RAPIDES	31 19	92 28W	87	08	08		H
ALEXANDRIA 5 SSE	0103	05	RAPIDES	31 15	92 27W	85	24	24		CH
ANGIE	0238	06	WASHINGTON	30 58	89 49W	130		08		H
ARCADIA	0277	02	BIENVILLE	32 33	92 55W	400		08		H
BAKER	0462	06	EAST BATON ROUGE	30 34	91 10W	70		08		H
BASTROP	0537	03	MOREHOUSE	32 46	92 0W	150	07	07		H
BATON ROUGE CONCORD	0548	06	EAST BATON ROUGE	30 25	91 8W	50		08		H
BATON ROUGE METRO AP R	0549	06	EAST BATON ROUGE	30 32	91 9W	64	24	24		HJ
BATON ROUGE SHERWOOD	0558	06	EAST BATON ROUGE	30 27	91 3W	55		08		H
BAYOU SORREL LOCK	0565	08	IBERVILLE	30 8	91 19W	15		08		H
BEAVER FIRE TWR	0617	05	EVANGELINE	30 48	92 30W	105		13		H
BELL CITY 13 SW	0658	07	CAMERON	29 58	93 5W	4		07		H
BENTON 5E	0718	01	BOSSIER	32 27	93 50W	200	08	08		H
BIENVILLE 3 NE	0800	02	BIENVILLE	32 22	92 57W	307	23	23		H
BOGALUSA	0945	06	WASHINGTON	30 47	89 51W	100	08	08		H
BOOTHVILLE ASOS R	1157	09	PLAQUEMINES	29 20	89 24W	3	24	24		H
BOYCE 3 WNW	1232	05	RAPIDES	31 24	92 43W	110	24	24		H
BUNKIE	1287	05	AVOYELLES	30 58	92 11W	80	08	08		CH
BUTTE LA ROSE	1365	08	ST. MARTIN	30 17	91 41W	5		07		H
CALHOUN RSCH STN	1411	02	OUACHITA	32 31	92 21W	180	08	08	08	GCH
CARENCRO	1535	08	LAFAYETTE	30 19	92 3W	50		07		H
CARVILLE 2 SW	1565	08	IBERVILLE	30 12	91 8W	25	24	24		H
CLAYTON	1866	05	CONCORDIA	31 43	91 32W	73		07		CH
CLINTON 5 SE	1899	06	EAST FELICIANA	30 49	90 58W	200	08	08		GCH
CLINTON FORESTRY HQ	1891	06	EAST FELICIANA	30 51	91 1W	250		13		H
COLUMBIA LOCK	1979	02	CALDWELL	32 10	92 6W	80	07	07		H
CONVENT 2S	2002	09	ST. JAMES	29 60	90 49W	25		08		H
COVINGTON 3 NE	2154	06	ST. TAMMANY	30 31	90 5W	25		07		H
CROWLEY 2 NE	2212	07	ACADIA	30 14	92 21W	25	08	08		G H
DE RIDDER	2367	07	BEAUREGARD	30 51	93 17W	190	08	08		H
DENHAM SPRINGS	2350	06	LIVINGSTON	30 29	90 58W	35		07		H
DONALDSONVILLE 4 SW	2534	08	ASSUMPTION	30 4	91 2W	30	08	08		CH
DRY CREEK 8NW	2641	07	BEAUREGARD	30 44	93 8W	95		07		H
DUTCHTOWN #2	2688	09	ASCENSION	30 15	90 59W	18		07		H
ELMER 2 SW	2812	05	RAPIDES	31 6	92 42W	230		07		H
EUNICE	2981	05	ST. LANDRY	30 29	92 26W	50	08	08		H
FARMERVILLE	3079	02	UNION	32 47	92 24W	180	07	07		H
FRANKLIN 3 NW	3313	08	ST. MARY	29 49	91 33W	12	24	24		H
GALLIANO	3433	09	LAFOURCHE	29 28	90 18W	5	08	08		H
GONZALES	3695	09	ASCENSION	30 12	90 55W	10		07		H
GORUM FIRE TWR	3741	04	NATCHITOCHES	31 26	92 53W	307		13		H
GRAND COTEAU	3800	05	ST. LANDRY	30 25	92 3W	55	17	17		H
GRAND ISLE	3807	09	JEFFERSON	29 14	89 59W	2		07		H
HACKBERRY 8 SSW	3979	07	CAMERON	29 53	93 24W	6	08	08		H
HAMMOND 5 E	4030	06	TANGIPAHOA	30 30	90 23W	35	08	08		GCH
HODGES GARDENS	4288	04	SABINE	31 22	93 23W	420	08	08		H
HOMER 1N	4355	02	CLAIBORNE	32 49	93 4W	215	07	07		H
HOSSTON	4398	01	CADDO	32 27	93 50W	246		08		H
HOUMA	4407	09	TERREBONNE	29 38	90 49W	8	08	08		H
JAMESTOWN	4592	01	BIENVILLE	32 21	93 12W	190		07		H
JEANERETTE 5 NW	4674	08	IBERIA	29 58	91 43W	20	08	08		H
JENA 4 WSW	4696	05	LA SALLE	31 38	92 12W	210	08	08		CH
JENNINGS	4700	07	JEFFERSON DAVIS	30 12	92 40W	25	08	08	08	GCH
JONESBORO 4 ENE	4732	02	JACKSON	32 15	92 39W	330		13		H
JONESVILLE LOCKS	4739	05	CATAHOULA	31 29	91 52W	70	06	06		CH
KAPLAN	4775	07	VERMILION	29 60	92 17W	15		07		H
KEITHVILLE	4816	01	CADDO	32 21	93 52W	200		07		H
KILLIAN	4878	06	LIVINGSTON	30 22	90 33W	10		08		H
KORAN	4931	01	BOSSIER	32 25	93 28W	175		08		H
LAFAYETTE	5021	08	LAFAYETTE	30 13	92 4W	25	22	22		CH
LAFAYETTE FCWOS R	5026	08	LAFAYETTE	30 12	91 59W	38	24	24		H

STATION INDEX

STATION	INDEX NO.	DIVISION	COUNTY	LATITUDE	LONGITUDE	ELEVATION (IN FEET)	OBSERVATION TIME AND TABLES / LOCAL STD TIME: TEMP	PRECIP	EVAP	SPECIAL SEE (NOTES)
LAKE ARTHUR 10 SW	5065	07	CAMERON	30 0	92 47W	10	08	08		H
LAKE CHARLES 2 N	5074	07	CALCASIEU	30 15	93 13W	5		08		H
LAKE CHARLES 7 NW	5072	07	CALCASIEU	30 18	93 16W	10		08		H
LAKE CHARLES AP R	5078	07	CALCASIEU	30 7	93 14W	9	24	24		HJ
LAKE CHARLES PORT	5076	07	CALCASIEU	30 13	93 15W	5		08		H
LAKE PROVIDENCE	5090	03	EAST CARROLL	32 48	91 10W	100	07	07		H
LEESVILLE	5266	04	VERNON	31 8	93 14W	28	08	08		H
LEESVILLE 6 SSW	5287	04	VERNON	31 3	93 17W	260		08		CH
LELAND BOWMAN LOCK	5296	07	VERMILION	29 47	92 12W	40	08	08		H
LIVERPOOL 6W	5430	06	ST. HELENA	30 56	90 34W	250		08		H
LIVINGSTON	5438	06	LIVINGSTON	30 31	90 45W	43		06		H
LOGANSPORT	5522	01	DE SOTO	31 58	94 0W	190		07		H
LSU BEN-HUR FARM	5620	06	EAST BATON ROUGE	30 22	91 10W	21	08	08		CH
LSU DEAN LEE RSCH STN	5630	05	RAPIDES	31 11	92 25W	70	08	08		G H
LUTCHER	5783	09	ST. JAMES	30 2	90 42W	20		07		H
MANSFIELD 7 NW	5875	01	DE SOTO	32 8	93 45W	255	08	08		CH
MANY 9 WSW	5896	04	SABINE	31 31	93 37W	286		07		H
MARKSVILLE	5920	05	AVOYELLES	31 8	92 4W	85		08		H
MARRERO 9 SSW	5926	09	JEFFERSON	29 47	90 7W	3	08	08		H
MINDEN	6244	01	WEBSTER	32 36	93 18W	185	07	07		CH
MONROE DELTA CC	6314	02	OUACHITA	32 30	92 2W	70		08		CH
MONROE REGIONAL AP R	6303	02	OUACHITA	32 31	92 2W	79	24	24		H
MOORINGSPORT 1 N	6364	01	CADDO	32 42	93 58W	200	08	08		H
MORGAN CITY	6394	08	ST. MARY	29 41	91 11W	5	08	08		CH
MOSS BLUFF	6431	07	CALCASIEU	30 18	93 12W	19		07		H
MOSS BLUFF 2 NNW	6434	07	CALCASIEU	30 20	93 13W	25	24	24		H
MOUNT HERMON 2W	6466	06	WASHINGTON	30 57	90 18W	320		08		H
NAPOLEONVILLE	6561	08	ASSUMPTION	29 56	91 1W	25		07		H
NATCHITOCHES #2	6584	04	NATCHITOCHES	31 49	93 5W	141	07	07		CH
NEW IBERIA AP ACADIANA RGNL R	6657	08	IBERIA	30 2	91 53W	24	24	24		H
NEW ORLEANS ALGIERS	6666	09	ORLEANS	29 57	90 3W	2		08		H
NEW ORLEANS AP R	6660	09	JEFFERSON	29 60	90 15W	4	24	24		HJ
NEW ORLEANS AUDUBON R	6664	09	ORLEANS	29 55	90 8W	20	24	24		H
NEW ORLEANS LKFRNT AP R	6667	09	ORLEANS	30 3	90 2W	9	24	24		H
NEW ROADS 5 NE	6686	05	POINTE COUPEE	30 44	91 22W	45	24	24		H
NORWOOD	6808	06	EAST FELICIANA	30 58	91 6W	102		08		H
OAK GROVE	6866	03	WEST CARROLL	32 52	91 23W	129		08		H
OAK RIDGE	6868	03	MOREHOUSE	32 37	91 47W	82		07		H
OAKDALE	6836	07	ALLEN	30 49	92 40W	110		07		H
OAKNOLIA 2N	6911	06	EAST FELICIANA	30 45	90 60W	150		07		H
OBERLIN FIRE TWR	6938	07	ALLEN	30 36	92 46W	65	09	09		H
OLD TOWN BAY	6968	07	CALCASIEU	30 17	93 9W	12		07		H
OPELOUSAS	6995	05	ST. LANDRY	30 30	92 6W	56		07		H
PINE GROVE FIRE TWR	7304	06	ST. HELENA	30 43	90 45W	190		13		H
PIONEER 6 W	7312	03	WEST CARROLL	32 45	91 32W	88		08		H
PLAQUEMINE 2 N	7366	08	IBERVILLE	30 19	91 15W	20		07		H
PONCHATOULA 4 SE	7425	06	TANGIPAHOA	30 25	90 23W	18		07		H
PORT ALLEN	7448	05	WEST BATON ROUGE	30 27	91 13W	15		07		H
RAYVILLE	7691	03	RICHLAND	32 30	91 45W	89	07	07		CH
RED RIVER LOCK # 2	7732	05	RAPIDES	31 11	92 17W	75		07		H
RED RIVER LOCK #1	7729	05	CATAHOULA	31 15	91 58W	70		07		H
RED RIVER RSCH STN	7738	01	BOSSIER	32 25	93 38W	155	07	07	07	GCH
ROCKEFELLER WL REFUGE	7932	07	CAMERON	29 44	92 49W	4	08	08		H
RUSTON LA TECH	8067	02	LINCOLN	32 32	92 41W	260	08	08		H
SAILES FIRE TWR	8094	02	BIENVILLE	32 22	93 9W	360		13		H
SHREVEPORT AP R	8440	01	CADDO	32 27	93 49W	254	24	24		HJ
SHREVEPORT DWTN	8436	01	CADDO	32 31	93 45W	180		07		H
SHREVEPORT DWTN AP R	8438	01	CADDO	32 33	93 45W	179	24	24		H
SHREVEPORT STHRN HILLS	8444	01	CADDO	32 24	93 47W	200	07	07		CH
SHREVEPORT WFO	8448	01	CADDO	32 27	93 50W	274	24	24		H
SLIDELL	8539	06	ST. TAMMANY	30 16	89 46W	10	08	08		CH
SLIDELL AP R	8543	06	ST. TAMMANY	30 21	89 49W	27	24	24		H
SPRINGHILL	8683	01	WEBSTER	32 60	93 27W	240		07		H
ST FRANCISVILLE	8136	06	WEST FELICIANA	30 47	91 23W	115		07		H
ST GABRIEL	8139	08	IBERVILLE	30 16	91 6W	30		08		H
ST JOSEPH 3 N	8163	03	TENSAS	31 57	91 14W	78	08	08	07	GCH

STATION INDEX

STATION	INDEX NO.	DIVISION	COUNTY	LATITUDE	LONGITUDE	ELEVATION (IN FEET)	OBSERVATION TIME AND TABLES LOCAL STD TIME TEMP	PRECIP	EVAP	SPECIAL SEE (NOTES)
ST MARTINVILLE 3 SW	8181	08	ST. MARTIN	30 5	91 52W	30	08	08		H
SULPHUR	8831	07	CALCASIEU	30 14	93 21W	10		24		H
SUN	8861	06	ST. TAMMANY	30 39	89 55W	75		06		H
TALISHEEK	8906	06	ST. TAMMANY	30 31	89 52W	60		08		H
TALLULAH	8923	03	MADISON	32 24	91 11W	85	08	08		H
TALLULAH VICKSBURG RGN R	8926	03	MADISON	32 21	91 2W	86	24	24		H
TERRYTOWN 3S	8941	09	JEFFERSON	29 55	90 2W	10	07	07		H
THIBODAUX 4 SE	9013	09	LAFOURCHE	29 45	90 46W	15	08	08		CH
TICKFAW 3 ENE	8945	06	TANGIPAHOA	30 36	90 27W	53		24		H
TOLEDO BEND LAKE	9074	04	SABINE	31 12	93 34W	181	08	08	08	H
VILLE PLATTE	9369	05	EVANGELINE	30 42	92 16W	70		07		H
VINTON 5W	9376	07	CALCASIEU	30 12	93 41W	11	08	08		H
VIVIAN	9392	01	CADDO	32 54	93 59W	220		07		H
WEST MONROE	9631	02	OUACHITA	32 28	92 9W	75		07		H
WINNFIELD 3 N R	9803	02	WINN	31 58	92 39W	160	24	24		H
WINNSBORO 2 SE	9804	03	FRANKLIN	32 8	91 43W	74	08	08		G H
WINNSBORO 5 SSE	9806	03	FRANKLIN	32 6	91 42W	80	07	07		GCH
ZWOLLE 2 NW	9980	04	SABINE	31 40	93 40W	209		07		H

REFERENCE NOTES

STATION NAMES: Name of the city, town or locality. Figures and letters following the station names indicate the distance in miles and direction from the post office or town community center.

DIVISIONS: Areas within a state of similar climatological characteristics. Division averages are calculated using data from stations that record temperature and/or precipitation. Station Precipitation totals flagged with an 'F' or 'M' are excluded from the Divisional Average calculations of precipitation. Stations with monthly Temperature averages flagged with an 'F' or 'M' are included in the Divisional Average if there are no more than 9 flagged or missing daily values in the month, else they are excluded from the divisional average for temperature.

NORMALS: The average value of the meteorological element over a time period. Effective 1 January 2012, the averaging period is 1981 to 2010. The normals for National Weather Service localities have been adjusted so as to be representative for the current observation site.

MONTHLY DEGREE DAY TOTALS: One heating (cooling) degree day is accumulated for each whole degree that the daily mean temperature is below (above) 65 degrees Fahrenheit.

PRECIPITATION: Values shown in hundredths of inches are water equivalent totals, i.e., total of liquid and melted frozen precipitation. In the "Monthly Summarized Data" table the total snow and sleet values shown in tenths of inches are unmelted amounts. The max depth on ground values of snow and sleet shown in whole inches are cumulative unmelted amounts. The number of days with .10, .50, 1.00 or more refers to water equivalents.

PRECIPITATION QUALITY CONTROL: The NCDC quality control process may flag precipitation data that are spatially inconsistent, exceed climatological limits, or are inconsistent with prevailing weather patterns.

TEMPERATURE: Original temperature values are given in the "Daily Temperature" table. Summary temperature information (averages, departures, extremes, monthly degree day totals) is based on the values labeled MAX/MIN.

WIND: (As shown in the "Evaporation and Wind" table) the total wind movement in miles over the evaporation pan as determined by an anemometer recorder located 6-8 inches above the pan.

SYMBOLS AND LETTERS USED IN THE STATION INDEX TABLE

C Station is equipped with recording rain gage (R) but values in this bulletin are from a non-recording rain gage unless indicated by an R.
G Observations appear in the "Soil Temperatures" table.
H Observations appear in the "Snowfall and Snow on the Ground" table.
J Station also published as a Local Climatological Data publication.
VAR Observation time varies.
SR Observation time near sunrise.
SS Observation time near sunset.

SYMBOLS AND LETTERS USED IN THE DATA TABLES

(DAILY DATA ARE FOR THE 24 HOURS IMMEDIATELY PRECEDING OBSERVATION TIME.)
BLANK Entries in the "Monthly Summarized Data" table indicate no record.
BLANK Entries in the "Daily Precipitation" and "Snowfall and Snow on the Ground" tables indicate zero.
BLANK Entries in the "Daily Temperature" table indicate a missing record

- No record. Data not recorded or not received in time for publication.
+ Precipitation or temperature extremes occurred on one or more previous dates during the month.
* Rain gage not read. Precipitation is included in the amount following the asterisks. Time distribution may not be known. A * preceding the monthly total indicates precipitation amount is being carried forward to next month's total, and may include amounts from the previous month(s).

a As a subscript, indicates accumulated total.
A Amount of precipitation is the total of observer's entries for the current month. It may include precipitation that occurred during the previous month. Refer to earlier bulletin to determine date of last reading. (Hawaii stations)
B Divisional Departure from normals are computed using 1971-2000 normals.
E Normalized HDD/CDD Calculation. E is appended to the HDD/CDD Calculation when 1-9 individual daily TMAX and/or TMIN values are missing and a Normalized HDD/CDD Calculation is provided. M appears alone if 10 or more daily values are missing.
F Monthly calculation flagged value. F is appended to average and/or total values computed which exclude one or more daily data values that have been flagged by the GHCN-Daily Dataset
M Insufficient or partial data. M is appended to average and/or total values computed with 1-9 daily values missing. M appears alone if 10 or more daily values are missing, (8 or more for wind and evaporation).
N Indicates snow fall or Snowdepth totals are computed with one or more missing days.
R Amounts from recording rain gage.
T Trace. An amount too small to measure.

SEASONAL TABLES: Monthly and seasonal snowfall and heating degree days for the 12 months ending with the June data are published in the July issue of this bulletin. Cooling degree days for the calendar year are published in the "Climatological Data Annual Summary."

Information concerning the history of changes in locations, exposure, etc. of substations is kept on file at the National Climatic Data Center. Historical information of regular National Weather Service Offices may be obtained from the "Local Climatological Data" annual publication. The contents of this publication may be reprinted or otherwise used freely, with proper credit to the National Climatic Data Center. The data are also available digitally.

Effective with the January 2011 Data-Month, COOP Observer Names are no longer included in the Monthly and Annual Climatological Data Publications. This information is not published to ensure the privacy of personal information pursuant to Section 208 of the E-Government Act of 2002 (44 USC 3601).

As of the 2011 Data-Year, Station and Climate Division Maps are no longer being included in the CD Publications. NCDC's Product Development Branch provides updated Station Maps for various data networks via the Historical Observing Metadata Repository: http://www.ncdc.noaa.gov/homr.

The GHCN-Daily Quality Control Flags shown below are displayed as superscripts with the data. For more Information on Global Historical Climatology Network - Daily and flags, see:
http://www.ncdc.noaa.gov/oa/climate/ghcn-daily/
and
Comprehensive Automated Quality Assurance of Daily Surface Observations.
Durre, Imke, Matthew J. Menne, Byron E. Gleason, Tamara G. Houston,
Russell S. Vose, 2010: J. Appl. Meteor. Climatol., 49, 16151633.
doi: 10.1175/2010JAMC2375.1

Blank = Passed All checks	N = failed naught check
D = failed duplicate check	O = failed climatological outlier check
G = failed gap check	R = failed lagged range check
I = failed internal consistency check	S = failed spatial consistency check
K = failed streak/frequent-value check	T = failed temporal consistency check
L = failed check on length of multiday period	W = temperature too warm for snow
M = failed megaconsistency check	X = failed bounds check
	Z = flagged as a result of an official Datzilla investigation

Beginning with the January 2013 CD Publication, monthly mean temperature calculations have changed to the National Data Stewardship Team standard. Monthly maximum and minimum temperatures are not rounded until after the monthly mean temperature is calculated. This is the most accurate outcome, but may be slightly different from the mean derived from rounded monthly maximum and minimum.

Processing Updates and Errata: The 2011 CD Publications were reproduced in May 2013. This update included the addition of late reports and corrections based on additional investigations of reported data issues through NCDCs Datzilla system. In addition, divisional averages for precipitation were recalculated using the method described in DIVISIONS above. Previous editions of the 2011 Publications included all precipitation stations regardless of missing data in the calculation of divisional averages. HDD/CDD values were recalculated to match the legacy method of calculation (truncation of monthly HDD/CDD values instead of rounding).

These and other publications are available from the National Climatic Data Center

Hourly Precipitation Data
This publication contains hourly precipitation amounts obtained from recording rain gages located at National Weather Service, Federal Aviation Administration, and cooperative observer stations. Published data are displayed in inches and tenths or inches and hundredths at local standard time. HPD includes maximum precipitation for nine (9) time periods from 15 minutes to 24 hours, for selected stations.

Climatological Data
Monthly editions contain station daily maximum and minimum temperatures and precipitation. Some Stations provide daily snowfall, snow depth, evaporation, and soil temperature data. Each edition also contains monthly summaries for heating and cooling degree days (65 degree F base). The July issue contains a recap of monthly heating degree days and snow data for the preceding July through June.

The Annual issue contains monthly and annual averages of temperature, precipitation, temperature extremes, freeze data, soil temperatures, evaporation, and a recap of monthly cooling degree days.

Storm Data
Monthly issues contain a chronological listing, by states, of occurrences of storms and unusual weather phenomena. Reports contain information on storm paths, deaths, injuries, and property damage. An "Outstanding storms of the month" section highlights severe weather events with photographs, illustrations, and narratives. The December issue includes annual tornado, lightning, flash flood, and tropical cyclone summaries.

Monthly Climatic Data for the World
This publication contains monthly means for temperature, pressure, precipitation, vapor pressure, and sunshine for approximately 2,000 surface data collection stations worldwide and monthly mean upper air temperatures, dew point depressions, and wind velocities for approximately 500 observing sites.

Local Climatological Data
LCD publications summmarize temperature, relative humidity, precipitation, cloudiness, wind speed and direction observations for several hundred cities in the U.S. and its territories. Each monthly publication also contains 3 hourly weather observations for that month and a hourly summary of precipitation. Annual LCD publications contain a summary of the past calendar year as well as historical averages and extremes.

For Information Call:
(828) 271-4800 Option 2
(828) 271-4010 (TDD)
(828) 271-4876 (Fax)

NOAA\National Climatic Data Center
Attn: User Engagement & Services Branch
151 Patton Avenue
Asheville, NC 28801-5001

Customer Services Number: (828) 271-4800, option 2
TDD : (828) 271-4010
Fax number: (828) 271-4876

NCDC now offers free online access to the ***Climatological Data*** **publication.**
Go to : **www.ncdc.noaa.gov** and choose Most Popular.
