# Supplementary material for: Why is the lawn buzzing?
Source: Biodivers Data J. 2014 Apr 24;(2):e1101. doi: 10.3897/BDJ.2.e1101 (PMC4040422; doi:10.3897/BDJ.2.e1101)
Supplement: Supplementary material 3 — Climatological Data for Louisiana, August 2013 [file biodiversity_data_journal-2-e1101-s003.pdf]

CLIMATOLOGICAL DATA

LOUISIANA

AUGUST 2013

VOLUME 118 NUMBER 08
ISSN 0145-0409
GHCND Ver: 3.12-upd-2013122706

AUGUST PRECIPITATION BY YEAR

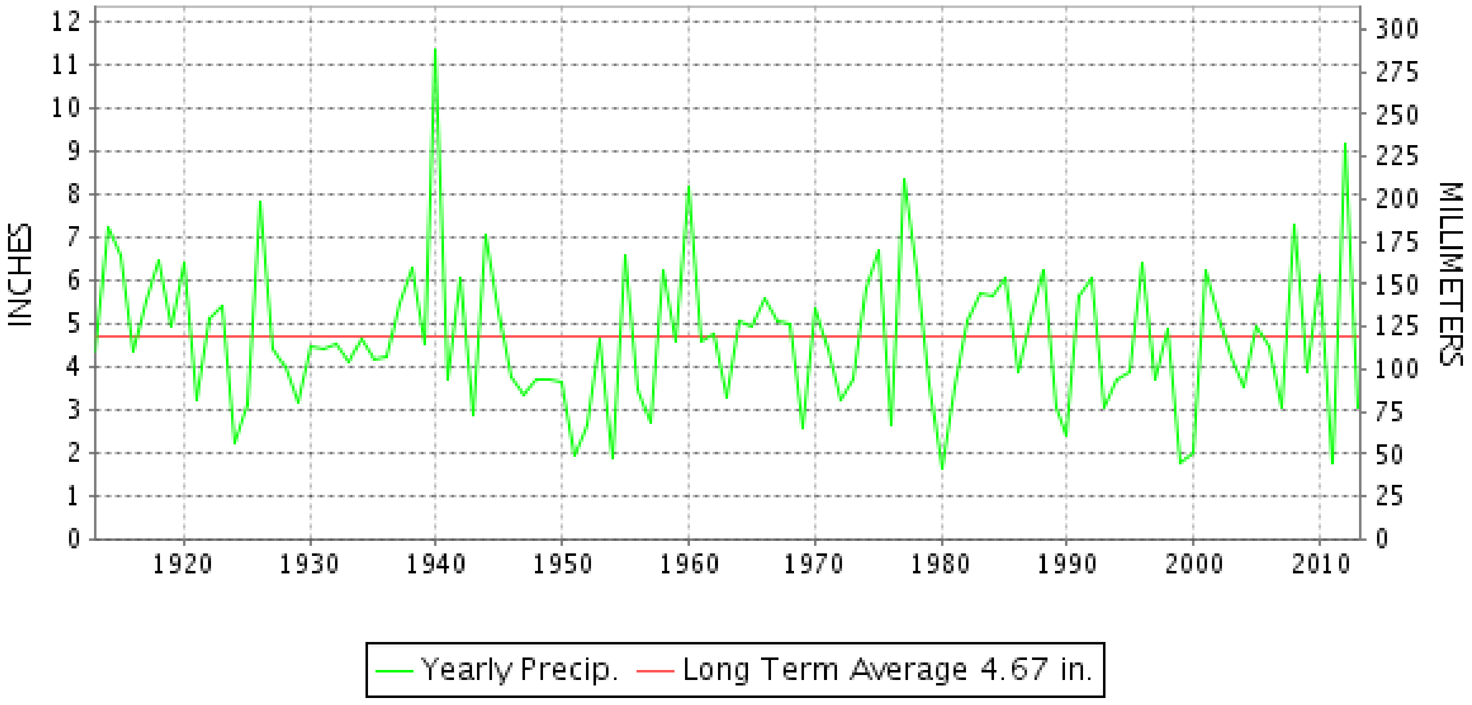

TEMPERATURE AND PRECIPITATION EXTREMES

**LOUISIANA**			
HIGHEST TEMPERATURE	102	AUGUST 09++	4 STATIONS
LOWEST TEMPERATURE	57	AUGUST 16	HOMER 1N
GREATEST TOTAL PRECIPITATION	10.92		BOOTHVILLE ASOS
LEAST TOTAL PRECIPITATION	0.00		KORAN
GREATEST 1 DAY PRECIPITATION	4.71	AUGUST 11	PINE GROVE FIRE TWR

"I certify that this is an official publication of the National Oceanic and Atmospheric Administration (NOAA) It is compiled using information from weather observing sites supervised by NOAA/National Weather Service and received at the National Climatic Data Center(NCDC), Asheville, North Carolina 28801."

Director
National Climatic Data Center

noaa

National
Oceanic and
Atmospheric Administration

National
Environmental Satellite, Data
and Information Service

National
Climatic Data Center
Asheville, North Carolina

LOUISIANA
201308

MONTHLY STATION AND DIVISION SUMMARY

STATION	TEMPERATURE (°F) AVERAGE MAXIMUM	AVERAGE MINIMUM	AVERAGE	DEPARTURE FROM NORMAL	HIGHEST	DATE	LOWEST	DATE	HEATING DEG. DAYS	COOLING DEG. DAYS	NO. OF DAYS MAX >=90	MAX <=32	MIN <=32	MIN <=0	PRECIPITATION (IN) TOTAL	DEPARTURE FROM NORMAL	GREATEST 24 HOURS	DATE	ICE PELLETS, SNOW TOTAL	MAX DEPTH ON GROUND	DATE	NO. OF DAYS .10 OR MORE	.50 OR MORE	1.00 OR MORE
**LOUISIANA**																								
**NORTHWEST 01**																								
BENTON 5E	94.0	72.0	83.0	0.2	99	10+	60	17	0	567	27	0	0	0	0.26	-2.66	0.26	14	0.0	0		1	0	0
HOSSTON															0.93		0.93	14	0.0	0		1	1	0
JAMESTOWN															A 0.25		0.09	25	0.0			0	0	0
KEITHVILLE															0.04		0.04	14	0.0	0		0	0	0
KORAN															0.00				0.0			0	0	0
LOGANSPORT															0.07		0.06	14	0.0	0		0	0	0
MANSFIELD 7 NW	M	M	M								0	0	0	0	M				0.0					
MINDEN	93.7	71.2	82.5	0.5	98	31+	61	18+	0	551	26	0	0	0	0.18	-2.51	0.18	14	0.0	0		1	0	0
MOORINGSPORT 1 N	95.4M	71.6M	83.5M	0.8	102	09+	60	18+	0	581E	26	0	0	0	M 0.76	-2.20	0.67	11	M 0.0	0		1	1	0
RED RIVER RSCH STN	94.6	71.8	83.2	0.4	98	29+	60	16	0	573	27	0	0	0	0.19	-2.57	0.09	15	M 0.0			0	0	0
SHREVEPORT DWTN															0.38		0.38	14	0.0	0		1	0	0
SHREVEPORT DWTN AP	95.7	73.4	84.5	1.3	100	31+	61	17+	0	611	27	0	0	0	0.44	-2.17	0.41	13	0.0			1	0	0
SHREVEPORT AP	97.0	73.0	85.0	1.9	102	31+	64	17+	0	629	29	0	0	0	0.18	-2.55	0.10	14	0.0	0		1	0	0
SHREVEPORT STHRN HILLS	97.1	71.2	84.1	1.4	102	09	62	18+	0	602	28	0	0	0	0.09	-2.70	0.09	15	0.0	0		0	0	0
SHREVEPORT WFO	95.6	73.5	84.5	1.4	100	31+	64	17	0	612	27	0	0	0	0.12	-2.54	0.08	13	0.0	0		0	0	0
SPRINGHILL															M				0.0					
VIVIAN															M				0.0					
**--DIVISIONAL DATA------->**			83.8	2.2B											0.24	-2.66B								
**NORTH CENTRAL 02**																								
ARCADIA															0.40		0.40	14	0.0	0		1	0	0
BIENVILLE 3 NE	94.8	71.3	83.0	1.3	99	31	61	17	0	567	27	0	0	0	0.04	-2.97	0.04	13	0.0			0	0	0
CALHOUN RSCH STN	93.3M	66.9M	80.1M	-1.0	97	31+	58	17	0	477E	25	0	0	0	M 0.70	-2.61	0.32	14	0.0			3	0	0
COLUMBIA LOCK	93.1M	70.5M	81.8M	0.3	99	09	64	30+	0	529E	25	0	0	0	0.61	-2.75	0.35	22	0.0	0		2	0	0
FARMERVILLE	92.5	71.3	81.9	0.2	97	11	59	15	0	532	25	0	0	0	0.12	-3.20	0.10	14	0.0	0		1	0	0
HOMER 1N	91.1M	68.0M	79.5M	-1.0	95	24+	57	16	0	455E	22	0	0	0	M 1.58	-1.19	1.58	14	0.0			1	1	1
JONESBORO 4 ENE															M				0.0					
MONROE REGIONAL AP	95.3	69.8	82.5	-0.1	100	09+	61	16	0	554	27	0	0	0	0.05	-3.13	0.03	21	0.0	0		0	0	0
MONROE DELTA CC															0.11	-3.11	0.08	24	0.0	0		0	0	0
RUSTON LA TECH	91.2	69.1	80.1	-0.4	95	25	60	16	0	478	24	0	0	0	1.96	-1.26	1.21	12	M 0.0	0		2	2	1
SAILES FIRE TWR															M 0.10		0.10	14	0.0	0		1	0	0
WEST MONROE															0.32		0.27	14	0.0			1	0	0
WINNFIELD 3 N	M	M	M								0	0	0	0	M				0.0					
**--DIVISIONAL DATA------->**			81.3	0.1B											0.45	-2.60B								
**NORTHEAST 03**																								
BASTROP	92.5M	69.9M	81.2M	0.4	98	10+	61	17+	0	508E	24	0	0	0	1.01	-2.00	0.73	11	0.0	0		2	1	0
LAKE PROVIDENCE	90.7	71.9	81.3	-0.8	97	07+	64	17+	0	514	21	0	0	0	1.18	-2.24	1.18	06	0.0	0		1	1	1

LOUISIANA
201308

MONTHLY STATION AND DIVISION SUMMARY

STATION	TEMPERATURE (°F)														PRECIPITATION (IN)									
											NO. OF DAYS								ICE PELLETS, SNOW			NO. OF DAYS		
											MAX		MIN											
	AVERAGE MAXIMUM	AVERAGE MINIMUM	AVERAGE	DEPARTURE FROM NORMAL	HIGHEST	DATE	LOWEST	DATE	HEATING DEG. DAYS	COOLING DEG. DAYS	>=90	<=32	<=32	<=0	TOTAL	DEPARTURE FROM NORMAL	GREATEST 24 HOURS	DATE	TOTAL	MAX DEPTH ON GROUND	DATE	.10 OR MORE	.50 OR MORE	1.00 OR MORE
OAK GROVE															0.10		0.10	14	0.0	0		1	0	0
OAK RIDGE															2.25		1.60	22	0.0	0		3	1	1
PIONEER 6 W															0.23		0.22	14	0.0	0		1	0	0
RAYVILLE	93.7	72.4	83.0	0.3	99	09+	63	17	0	564	26	0	0	0	0.37	-2.96	0.30	22	0.0	0		1	0	0
ST JOSEPH 3 N	M	71.5M	MM		95	25+	63	28	0	465E	14	0	0	0	0.80	-3.18	0.80	14	0.0	0		1	1	0
TALLULAH	M	M	M		95	26+	64	28+	0	451E	11	0	0	0	M 0.76	-2.49	0.30	22	0.0	0		4	0	0
TALLULAH VICKSBURG RGN	92.3	68.7	80.5	-0.4	97	09	60	28	0	487	24	0	0	0	1.05	-2.60	0.68	09	0.0	0		3	1	0
WINNSBORO 2 SE	95.2	70.5	82.8	0.3	102	07	64	16+	0	563	27	0	0	0	1.24	-2.29	1.24	14	0.0	0		1	1	1
WINNSBORO 5 SSE	93.9	69.5	81.7	-0.6	100	08	60	18	0	526	26	0	0	0	1.47	-1.90	0.79	14	0.0	0		3	1	0
**--DIVISIONAL DATA------->**			81.8	0.3B											0.97	-2.03B								
**WEST CENTRAL 04**																								
GORUM FIRE TWR															M				0.0					
HODGES GARDENS	94.6M	71.0M	82.8M	0.4	100	14+	62	17	0	559E	25	0	0	0	1.06	-2.87	0.51	15	0.0			3	1	0
LEESVILLE	95.2	69.7	82.5	1.3	100	14+	62	20+	0	550	28	0	0	0	1.80	-1.75	1.19	15	0.0			3	1	1
LEESVILLE 6 SSW															1.22		0.83	12	0.0			4	1	0
MANY 9 WSW															1.01		0.37	12	0.0	0		4	0	0
NATCHITOCHES #2	91.7	71.7	81.7	-1.6	98	14	62	17	0	526	25	0	0	0	0.62	-2.59	0.38	14	0.0	0		2	0	0
TOLEDO BEND LAKE	95.1	71.9	83.5	1.2	99	10+	65	18+	0	583	28	0	0	0	0.84	-2.55	0.33	10	0.0	0		3	0	0
ZWOLLE 2 NW															0.83		0.40	12	0.0	0		3	0	0
**--DIVISIONAL DATA------->**			82.6	1.4B											1.05	-2.41B								
**CENTRAL 05**																								
ALEXANDRIA	94.0	72.3	83.2	0.0	99	03	64	25	0	572	27	0	0	0	4.00	-0.11	2.47	15	M 0.0	0		4	2	1
ALEXANDRIA 5 SSE	92.3	71.6	82.0	1.0	99	06	67	19+	0	532	22	0	0	0	3.67	0.50	2.07	13	M 0.0	0		3	2	2
BEAVER FIRE TWR															1.68		0.34	12	0.0			6	0	0
BOYCE 3 WNW	90.0	73.3	81.6	-1.1	98	08	64	17	0	524	18	0	0	0	1.51	-2.00	1.31	14	M 0.0	0		2	1	1
BUNKIE	91.6	71.5	81.6	-0.6	97	14+	65	18+	0	520	19	0	0	0	3.03	-0.75	1.05	15	0.0			5	3	1
CLAYTON															M 3.25		2.50	14	0.0	0		4	1	1
EUNICE	93.0	71.7	82.4	-1.5	98	10+	65	17	0	546	25	0	0	0	1.28	-2.98	0.55	11	0.0			3	1	0
GRAND COTEAU	91.6M	71.7M	81.7M	-0.4	96	13+	67	16	0	524E	21	0	0	0	M 2.27	-2.10	0.96	14	0.0			6	1	0
JENA 4 WSW	92.6M	70.1M	81.4M	0.4	97	14+	63	16	0	514E	24	0	0	0	M 2.60	-0.50	1.75	14	0.0			4	1	1
JONESVILLE LOCKS	90.8	73.1	82.0	-0.5	96	10	66	18	0	533	20	0	0	0	4.79	1.27	3.90	15	0.0	0		2	2	1
LSU DEAN LEE RSCH STN	M	M	M		98	07	66	19	0	539E	15	0	0	0	M				0.0					
MARKSVILLE															1.71		1.44	21	0.0			2	1	1
NEW ROADS 5 NE	91.6	72.8	82.2	-0.6	100	08	67	29	0	542	22	0	0	0	4.03	-0.67	1.25	13	0.0			5	4	1
OPELOUSAS															2.52		1.24	27	0.0			6	2	1
PORT ALLEN															2.44		1.80	14	0.0			3	1	1
RED RIVER LOCK #1															1.21		0.62	15	0.0	0		3	1	0
RED RIVER LOCK # 2															3.22		1.20	21	0.0			6	3	1

LOUISIANA
201308

MONTHLY STATION AND DIVISION SUMMARY

STATION	TEMPERATURE (°F)														PRECIPITATION (IN)									
											NO. OF DAYS								ICE PELLETS, SNOW			NO. OF DAYS		
											MAX		MIN											
	AVERAGE MAXIMUM	AVERAGE MINIMUM	AVERAGE	DEPARTURE FROM NORMAL	HIGHEST	DATE	LOWEST	DATE	HEATING DEG. DAYS	COOLING DEG. DAYS	>=90	<=32	<=32	<=0	TOTAL	DEPARTURE FROM NORMAL	GREATEST 24 HOURS	DATE	TOTAL	MAX DEPTH ON GROUND	DATE	.10 OR MORE	.50 OR MORE	1.00 OR MORE
VILLE PLATTE															2.59		1.42	12	0.0			5	2	1
**--DIVISIONAL DATA------->**			82.0	0.3B											2.69	-1.41B								
**EAST CENTRAL 06**																								
ABITA RVR COVINGTON															M 5.72		1.63	22	0.0			8	3	3
ABITA SPRINGS 1 SW															F 4.35		1.77	11	M 0.0			7	2	2
ABITA SPRING FIRE TWR															3.31		1.39	14	0.0			5	2	2
ANGIE															M				0.0					
BAKER															MA 4.52		1.09	22	0.0			5	3	1
BATON ROUGE CONCORD															4.80		2.11	14	M 0.0			6	2	2
BATON ROUGE METRO AP	90.9	72.7	81.8	-1.1	96	10+	66	29+	0	529	21	0	0	0	3.36	-2.46	1.15	14	0.0	0		6	2	1
BATON ROUGE SHERWOOD															4.85		1.82	15	0.0			5	4	1
BOGALUSA	M	M	M								0	0	0	0	M				0.0					
CLINTON FORESTRY HQ															4.19		4.05	14	0.0			1	1	1
CLINTON 5 SE	88.8	69.9	79.4	-0.6	95	09	62	29+	0	456	13	0	0	0	2.94	-2.45	1.30	14	0.0			5	2	2
COVINGTON 3 NE															M				0.0					
DENHAM SPRINGS															5.39		2.50	14	M 0.0	0		6	3	2
HAMMOND 5 E	89.9	70.8	80.3	-1.3	96	07+	61	28	0	479	18	0	0	0	4.18	-2.29	1.06	12	0.0			8	5	1
KILLIAN															M 3.49		1.50	14	0.0			4	3	1
LIVERPOOL 6W															M				0.0					
LIVINGSTON															6.58		1.83	13	0.0			6	5	4
LSU BEN-HUR FARM	90.1	72.1	81.1	-0.9	97	05	65	29+	0	507	22	0	0	0	7.16	1.80	3.09	14	0.0			7	5	2
MOUNT HERMON 2W															6.40		3.64	14	0.0			7	2	2
NORWOOD															6.40		2.25	14	0.0			5	3	3
OAKNOLIA 2N															5.47		1.77	15	0.0			8	3	2
PINE GROVE FIRE TWR															6.58		4.71	11	0.0			3	3	2
PONCHATOULA 4 SE															M 7.35		2.55	15	0.0			11	4	2
ST FRANCISVILLE															4.09		1.73	14	0.0			4	3	1
SLIDELL	88.0	73.1	80.6	-1.3	93	08+	65	28	0	489	13	0	0	0	3.02	-3.90	1.16	17	0.0			8	2	1
SLIDELL AP	89.4	72.9	81.1	-0.5	96	04	64	28	0	506	15	0	0	0	5.01	-2.03	1.30	05	0.0			9	3	2
SUN															8.27		2.88	12	0.0			9	4	2
TALISHEEK															A 5.77		1.66	14	0.0			6	4	3
TICKFAW 3 ENE															M				0.0					
**--DIVISIONAL DATA------->**			80.7	-0.5B											5.15	-0.36B								
**SOUTHWEST 07**																								
ABBEVILLE															4.78		1.49	15	0.0			7	3	2
BELL CITY 13 SW															3.79		1.04	15	0.0			6	4	1
CROWLEY 2 NE	91.5	71.9	81.7	-0.6	98	05	67	19	0	526	20	0	0	0	5.54	0.56	2.22	25	0.0			6	3	2
DE RIDDER	93.3	70.4	81.8	-0.5	100	06	66	17	0	530	27	0	0	0	1.63	-2.71	0.68	27	0.0			4	2	0

LOUISIANA
201308

MONTHLY STATION AND DIVISION SUMMARY

STATION	TEMPERATURE (°F) AVERAGE MAXIMUM	AVERAGE MINIMUM	AVERAGE	DEPARTURE FROM NORMAL	HIGHEST	DATE	LOWEST	DATE	HEATING DEG. DAYS	COOLING DEG. DAYS	NO. OF DAYS MAX >=90	MAX <=32	MIN <=32	MIN <=0	PRECIPITATION (IN) TOTAL	DEPARTURE FROM NORMAL	GREATEST 24 HOURS	DATE	ICE PELLETS, SNOW TOTAL	MAX DEPTH ON GROUND	DATE	NO. OF DAYS .10 OR MORE	.50 OR MORE	1.00 OR MORE
DRY CREEK 8NW															M				0.0					
HACKBERRY 8 SSW	89.7	76.1	82.9	-0.9	94	10+	71	17	0	562	20	0	0	0	5.83	1.08	2.37	15	0.0			5	3	3
JENNINGS	92.3	73.2	82.7	0.3	98	08+	68	18+	0	557	24	0	0	0	3.43	-1.86	1.55	11	0.0			7	2	1
KAPLAN															3.02		1.76	15	0.0			5	2	1
LAKE ARTHUR 10 SW	91.8	74.4	83.1	-0.6	97	14+	67	17	0	569	25	0	0	0	2.72	-2.79	0.73	05	0.0			7	2	0
LAKE CHARLES 7 NW															4.19		0.92	11	0.0			10	3	0
LAKE CHARLES 2 N															2.56		0.97	21	0.0			9	2	0
LAKE CHARLES PORT															3.68		1.35	21	0.0			7	3	1
LAKE CHARLES AP	93.3	74.8	84.1	1.1	98	13+	71	19+	0	601	27	0	0	0	3.71	-1.15	1.88	14	0.0	0		4	3	1
LELAND BOWMAN LOCK	93.0M	73.4M	83.2M	0.9	99	09	65	18	0	573E	24	0	0	0	4.43	-1.71	1.65	15	M 0.0	0		10	2	1
MOSS BLUFF															3.07		0.78	11	0.0			7	3	0
MOSS BLUFF 2 NNW	93.5	71.8	82.6	0.3	100	06	66	19+	0	554	27	0	0	0	4.43	-0.26	2.47	10	0.0			7	2	1
OAKDALE															0.95		0.70	14	0.0			2	1	0
OBERLIN FIRE TWR	92.4	71.6	82.0	-0.1	99	10	66	19+	0	534	24	0	0	0	1.43	-3.02	0.63	21	0.0			3	2	0
OLD TOWN BAY															M 3.97		1.00	21	0.0			9	3	1
ROCKEFELLER WL REFUGE	89.0	74.2	81.6	-0.2	93	24+	69	18+	0	522	18	0	0	0	3.02	-3.37	0.73	15	0.0			7	2	0
SULPHUR															1.04		0.55	26	0.0			4	1	0
VINTON 5W	94.0M	72.3M	83.2M		100	10	66	17	0	574E	26	0	0	0	M 2.62		1.11	12	0.0			4	2	1
**--DIVISIONAL DATA------->**			82.6	0.8B											3.33	-1.84B								
**SOUTH CENTRAL 08**																								
BAYOU SORREL LOCK															3.00		1.10	11	0.0			5	3	1
CARENCRO															5.42		1.65	04	0.0			6	5	2
CARVILLE 2 SW	89.0	74.5	81.7	-0.7	95	09	69	29+	0	528	15	0	0	0	5.37	0.29	2.05	14	0.0			8	4	2
DONALDSONVILLE 4 SW	89.1	72.1	80.6	-1.2	95	09	65	29	0	490	14	0	0	0	8.38	2.88	2.90	15	M 0.0			8	6	3
FRANKLIN 3 NW	88.4	73.6	81.0	-0.5	92	08+	68	29	0	505	17	0	0	0	3.23	-4.73	1.19	14	0.0			8	2	1
JEANERETTE 5 NW	90.1	72.3	81.2	-1.0	94	08	67	29	0	508	19	0	0	0	4.77	-1.46	1.15	27	0.0			7	6	1
LAFAYETTE	91.8	74.0	82.9	1.1	96	03+	68	17+	0	563	26	0	0	0	3.23	-1.70	1.84	15	M 0.0	0		3	3	1
LAFAYETTE FCWOS	91.6	74.3	83.0	-0.6	96	13+	69	17	0	562	21	0	0	0	3.76	-0.86	1.29	26	0.0	0		4	3	3
MORGAN CITY	89.5	74.3	81.9	-0.3	95	08	70	29	0	533	17	0	0	0	4.13	-3.23	0.93	06	0.0			10	2	0
NAPOLEONVILLE															4.09		0.76	15	0.0			9	4	0
NEW IBERIA AP ACADIANA RGNL	93.1	75.6	84.4	1.5	97	07+	72	29+	0	609	26	0	0	0	3.99	-1.09	1.18	14	0.0			9	2	2
PLAQUEMINE 2 N															6.39		2.10	14	0.0			7	6	3
ST GABRIEL															5.63		1.72	14	0.0			7	4	3
ST MARTINVILLE 3 SW	89.8	71.9	80.9	-0.8	95	09+	67	29	0	498	17	0	0	0	7.13	1.68	2.98	21	0.0			8	3	3
**--DIVISIONAL DATA------->**			82.0	0.2B											4.89	-1.35B								
**SOUTHEAST 09**																								
BOOTHVILLE ASOS	86.9	76.8	81.9	-1.0	92	07	72	17	0	530	7	0	0	0	10.92	3.43	2.68	18	0.0			13	7	2
CONVENT 2S															3.70		0.96	14	0.0			7	4	0

LOUISIANA
201308

MONTHLY STATION AND DIVISION SUMMARY

STATION	TEMPERATURE (°F)										NO. OF DAYS				PRECIPITATION (IN)				ICE PELLETS, SNOW			NO. OF DAYS		
											MAX		MIN											
	AVERAGE MAXIMUM	AVERAGE MINIMUM	AVERAGE	DEPARTURE FROM NORMAL	HIGHEST	DATE	LOWEST	DATE	HEATING DEG. DAYS	COOLING DEG. DAYS	>=90	<=32	<=32	<=0	TOTAL	DEPARTURE FROM NORMAL	GREATEST 24 HOURS	DATE	TOTAL	MAX DEPTH ON GROUND	DATE	.10 OR MORE	.50 OR MORE	1.00 OR MORE
DUTCHTOWN #2															5.26		2.03	15	0.0			11	2	2
GALLIANO	87.9M	75.2M	81.6M	-0.9	94	09	69	29+	0	523E	10	0	0	0	M 5.17	-2.41	1.25	10	0.0			12	3	1
GONZALES															4.71		1.44	15	0.0			7	4	2
GRAND ISLE															M 9.14		2.30	17	0.0			10	7	5
HOUMA	M	M	M		93	08+	71	14	0	529E	9	0	0	0	M				0.0					
LUTCHER															3.91		2.35	14	0.0			6	2	1
MARRERO 9 SSW	90.3M	73.5M	81.9M	0.1	96	08	68	29+	0	531E	12	0	0	0	M				0.0					
NEW ORLEANS AP	90.0	75.4	82.7	-0.6	97	07	69	28	0	556	17	0	0	0	7.22	1.24	1.74	21	0.0	0		10	5	3
NEW ORLEANS AUDUBON	91.0M	75.3M	83.2M	-1.0	97	07	70	29+	0	569E	22	0	0	0	3.13	-3.07	0.76	14	0.0			7	2	0
NEW ORLEANS ALGIERS															M				0.0					
NEW ORLEANS LKFRNT AP	90.0	78.8	84.4	-0.1	96	07	74	19	0	610	18	0	0	0	2.46	-2.76	0.39	21	0.0			10	0	0
TERRYTOWN 3S	90.3	75.2	82.7	-1.2	97	07+	69	29+	0	557	22	0	0	0	3.86	-3.72	0.77	15	M 0.0			11	2	0
THIBODAUX 4 SE	87.9	71.7	79.8	-2.1	94	08+	65	29	0	468	10	0	0	0	6.44	-1.02	1.69	10	0.0			8	5	3
**--DIVISIONAL DATA------->**			82.3	0.1B											5.16	-1.14B								

LOUISIANA
201308

DAILY PRECIPITATION (INCHES)

STATION	TOTAL	01	02	03	04	05	06	07	08	09	10	11	12	13	14	15	16	17	18	19	20	21	22	23	24	25	26	27	28	29	30	31
**LOUISIANA**																																
**NORTHWEST 01**																																
BENTON 5E	0.26														0.26																	
HOSSTON	0.93														0.93																	
JAMESTOWN	A 0.25														*	0.16a										0.09						
KEITHVILLE	0.04														0.04																	
KORAN	0.00																															
LOGANSPORT	0.07														0.06													0.01				
MANSFIELD 7 NW	M	-	-	-	-	-	-	-	-	-	-	-	-	-	-	-	-	-	-	-	-	-	-	-	-	-	-	-	-	-	-	-
MINDEN	0.18														0.18																	
MOORINGSPORT 1 N	M 0.76											0.67			0.08	0.01							-									
RED RIVER RSCH STN	0.19														0.03	0.09									0.07							
SHREVEPORT DWTN	0.38											T			0.38											T						
SHREVEPORT DWTN AP	0.44										0.02			0.41	0.01																	
SHREVEPORT AP	0.18										T			0.08	0.10																	
SHREVEPORT STHRN HILLS	0.09														T	0.09																
SHREVEPORT WFO	0.12										T			0.08	0.04																	
SPRINGHILL	M	-	-	-	-	-	-	-	-	-	-	-	-	-	-	-	-	-	-	-	-	-	-	-	-	-	-	-	-	-	-	-
VIVIAN	M	-	-	-	-	-	-	-	-	-	-	-	-	-	-	-																
**NORTH CENTRAL 02**																																
ARCADIA	0.40														0.40																	
BIENVILLE 3 NE	0.04													0.04																		
CALHOUN RSCH STN	M 0.70													0.16	0.32				-					0.22								
COLUMBIA LOCK	0.61													0.05		0.16							0.35		0.05							
FARMERVILLE	0.12													0.02	0.10				T						T							
HOMER 1N	M 1.58		-									-			1.58											T						
JONESBORO 4 ENE	M	-	-	-	-	-	-	-	-	-	-	-	-	-	-	-	-	-	-	-	-	-	-	-	-	-	-	-	-	-	-	-
MONROE REGIONAL AP	0.05	T											T	0.02								0.03	T	T								
MONROE DELTA CC	0.11														0.03										0.08							
RUSTON LA TECH	1.96										0.01		1.21	0.01	0.73																	
SAILES FIRE TWR	M 0.10			-											0.10			-														
WEST MONROE	0.32												0.03	0.02	0.27																	
WINNFIELD 3 N	M	-	-	-	-	-	-	-	-	-	-	-	-	-	-	-	-	-	-	-	-	-	-	-	-	-	-	-	-	-	-	-
**NORTHEAST 03**																																
BASTROP	1.01											0.73	T	0.03					0.17				0.08									
LAKE PROVIDENCE	1.18						1.18																		T							
OAK GROVE	0.10						T								0.10				T													
OAK RIDGE	2.25												T		0.45				0.20				1.60									
PIONEER 6 W	0.23														0.22				0.01													
RAYVILLE	0.37														0.07								0.30									
ST JOSEPH 3 N	0.80														0.80																	
TALLULAH	M 0.76				-						0.21			0.10	0.15								0.30									

LOUISIANA
201308

DAILY PRECIPITATION (INCHES)

STATION	TOTAL	DAY OF MONTH																														
		01	02	03	04	05	06	07	08	09	10	11	12	13	14	15	16	17	18	19	20	21	22	23	24	25	26	27	28	29	30	31
TALLULAH VICKSBURG RGN	1.05	T		T	0.11	T				0.68	T		0.01	0.20	0.05			T				T			T							
WINNSBORO 2 SE	1.24														1.24																	
WINNSBORO 5 SSE	1.47											0.24		0.06	0.79	0.06							0.32									
**WEST CENTRAL 04**																																
GORUM FIRE TWR	M	-	-	-	-	-	-	-	-	-	-	-	-	-	-	-	-	-	-	-	-	-	-	-	-	-	-	-	-	-	-	-
HODGES GARDENS	1.06													0.03	0.29	0.51								0.19				0.04				
LEESVILLE	1.80	0.01										0.30	0.01	0.09		1.19											0.19	0.01				
LEESVILLE 6 SSW	1.22	0.03	0.10										0.83			0.12											0.14					
MANY 9 WSW	1.01											0.02	0.37		0.15	0.22												0.25				
NATCHITOCHES #2	0.62													T	0.38	0.03								T			T	0.21				
TOLEDO BEND LAKE	0.84										0.33	0.32				0.13												0.06				
ZWOLLE 2 NW	0.83												0.40		0.15	0.09												0.19				
**CENTRAL 05**																																
ALEXANDRIA	4.00											0.36			0.35	2.47						0.75				0.01		0.06				
ALEXANDRIA 5 SSE	3.67										0.15	0.01	0.02	2.07	1.33						0.02				0.02		0.05					
BEAVER FIRE TWR	1.68					0.18						0.19	0.34		0.28	0.04					0.19	0.05					0.09	0.32				
BOYCE 3 WNW	1.51												0.06	0.14	1.31																	
BUNKIE	3.03												0.73		0.57	1.05						0.05	0.20					0.43				
CLAYTON	M 3.25													0.35	2.50	-							0.20				0.20					
EUNICE	1.28					0.14						0.55	0.08	0.32	0.03	0.05						0.04	0.03					0.04				
GRAND COTEAU	M 2.27					0.26									0.96	0.30					0.40		0.10				-	0.25				
JENA 4 WSW	M 2.60													0.15	1.75	0.40		-	-	-			0.05				0.25					
JONESVILLE LOCKS	4.79													0.06	0.83	3.90																
LSU DEAN LEE RSCH STN	M			-	-						-	-	0.11	1.13	1.04	1.64	-	-	-			1.40	0.45		-	-		0.05			-	-
MARKSVILLE	1.71														0.27							1.44										
NEW ROADS 5 NE	4.03									0.47	0.70	0.98		1.25	0.60							0.03										
OPELOUSAS	2.52				0.52						0.23			0.04							0.15	0.03				0.11	0.20	1.24				
PORT ALLEN	2.44			T							T		0.03	T	1.80	0.05			T			0.25				T		0.31				
RED RIVER LOCK #1	1.21													0.18	0.38	0.62			0.03													
RED RIVER LOCK # 2	3.22		0.10									0.15		0.06	0.67	0.85						1.20	0.08			0.11						
VILLE PLATTE	2.59					0.35							1.42		0.07	0.10							0.50					0.15				
**EAST CENTRAL 06**																																
ABITA RVR COVINGTON	M 5.72	0.05	0.05		-	0.23					0.07	1.36	0.07	0.05	0.34	1.13		0.20				0.30	1.63		0.22	-	0.02					
ABITA SPRINGS 1 SW	F 4.35	0.08	0.04			0.05					0.19	1.77	0.33	0.03	0.14	1.15		0.19	T	T		0.55L	0.55L		0.32		0.06					
ABITA SPRING FIRE TWR	3.31	0.15	0.03							0.04	0.10	1.05		0.07	1.39	0.08		0.23	0.08						0.03		0.06					
ANGIE	M	-	-	-	-	-	-	-	-	-	-	-	-	-	-	-	-	-	-	-	-	-	-	-	-	-	-	-	-	-	-	-
BAKER	MA 4.52			-	-						-	-	0.90	*	1.30a	0.30		*	*	0.00a		0.14	1.09	0.02	*	*	0.22a	0.55				-
BATON ROUGE CONCORD	4.80			0.20							0.02		0.04	0.38	2.11	1.22			T			0.49	T		T	0.07	T	0.27				
BATON ROUGE METRO AP	3.36		0.05							T		0.09	0.32	0.60	1.15			T		0.03	0.42	0.29	T			T	0.41					
BATON ROUGE SHERWOOD	4.85										0.02		0.30	0.01	0.90	1.82						0.80	0.01				0.09	0.90				
BOGALUSA	M	-	-	-	-	-	-	-	-	-	-	-	-	-	-	-	-	-	-	-	-	-	-	-	-	-	-	-	-	-	-	-
CLINTON FORESTRY HQ	4.19									0.06					4.05	0.08																

LOUISIANA
201308

DAILY PRECIPITATION (INCHES)

STATION	TOTAL	01	02	03	04	05	06	07	08	09	10	11	12	13	14	15	16	17	18	19	20	21	22	23	24	25	26	27	28	29	30	31
		DAY OF MONTH																														
CLINTON 5 SE	2.94												0.12	0.08	1.30	1.23						0.10	0.01	0.10								
COVINGTON 3 NE	M	-	-	-	-	-	-	-	-	-	-	-	-	-	-	-	-	-	-	-	-	-	-	-	-	-	-	-	-	-	-	-
DENHAM SPRINGS	5.39		0.01										0.32	0.10	2.50	1.55	0.01			0.03		0.50	0.07	0.05			0.06	0.19				
HAMMOND 5 E	4.18										0.31		1.06	0.52	0.77	0.69						0.13	0.55		0.11		0.04	T				
KILLIAN	M 3.49							-	-	-				0.90	1.50	0.81							-				0.28					
LIVERPOOL 6W	M	-	-	-	-	-	-	-	-	-	-	-	-	-	-	-	-	-	-	-	-	-	-	-	-	-	-	-	-	-	-	-
LIVINGSTON	6.58										0.09		0.33	1.83	1.61	1.09						0.54	1.09									
LSU BEN-HUR FARM	7.16			0.97							1.20		0.19	0.01	3.09	0.75						0.61	0.01				0.03	0.30				
MOUNT HERMON 2W	6.40		0.06							0.16	0.17		0.34	1.46	3.64					0.25			0.32									
NORWOOD	6.40									0.05	1.64			0.37	2.25	1.97						0.12										
OAKNOLIA 2N	5.47										0.41	0.06	0.31	0.17	1.65	1.77						0.32	0.65	0.02				0.11				
PINE GROVE FIRE TWR	6.58										0.65	4.71			1.22																	
PONCHATOULA 4 SE	M 7.35	T	-			1.98				0.28	0.51	0.03	0.30	0.19	0.31	2.55						0.52			0.30		0.16	0.22				
ST FRANCISVILLE	4.09										0.05	0.46	0.76	0.03	1.73	0.98			0.07				T		T	T		0.01				
SLIDELL	3.02					0.17				0.02	0.35	T	0.20	T	0.16	0.52	T	1.16	0.05	T	T	0.01	0.11	0.10	0.06	0.05	0.06					
SLIDELL AP	5.01					1.30			T	0.40	0.04	0.21	0.06	1.10	0.92		0.09	0.10	T		0.28	0.01	0.16	0.02	0.29	0.03						
SUN	8.27		0.21			0.08					0.02	0.03	2.88	0.44	0.62	2.76		0.15		0.02	0.67	0.25	0.03	0.01	0.10							
TALISHEEK	A 5.77	0.03									0.16	1.50	0.09	0.08	1.66	1.11		0.02	T		0.02	0.68	*	*	0.24a		0.18					
TICKFAW 3 ENE	M	-	-	-	-	-	-	-	-	-	-	-	-	-	-	-	-	-	-	-	-	-	-	-	-	-	-	-	-	-	-	-
**SOUTHWEST 07**																																
ABBEVILLE	4.78					0.33	0.33					0.17		0.39	T	1.49			0.02		T			0.01		0.07	0.73	1.24				
BELL CITY 13 SW	3.79													0.07	0.01	1.04	0.50		0.22					0.10			0.90	0.95				
CROWLEY 2 NE	5.54					0.99							0.19			1.52						0.28				2.22	0.28	0.06				
DE RIDDER	1.63											T	0.60			T	0.10						0.25					0.68				
DRY CREEK 8NW	M	-	-	-	-	-	-	-	-	-	-	-	-	-	-	-	-	-	-	-	-	0.51	0.73	-	-	-	-	-	-	-	-	-
HACKBERRY 8 SSW	5.83							0.01				0.07	0.01			2.37	0.10					1.76					0.39	1.12				
JENNINGS	3.43					0.14					T	1.55	0.14	0.34		0.13	0.02					0.80	0.02				0.26	0.03				
KAPLAN	3.02						0.16				0.03	0.05	0.10			1.76						0.16			0.08	0.03	0.65					
LAKE ARTHUR 10 SW	2.72					0.73						0.61	0.07	0.12		0.23	0.05					0.03				0.15	0.30	0.42	0.01			
LAKE CHARLES 7 NW	4.19					0.30					0.85	0.92	0.22	0.05	0.10	0.38	0.20					0.40					0.55	0.22				
LAKE CHARLES 2 N	2.56										0.16	0.12	0.11	0.54	0.01	0.10	0.18					0.97			0.01		0.20	0.16				
LAKE CHARLES PORT	3.68					0.22					0.01	0.10	0.31	0.50	0.02	0.69	0.38					1.35	0.02				0.04	0.02	0.02			
LAKE CHARLES AP	3.71				0.05						0.04	0.07	T		1.88	0.59					0.21	T			T	0.05	0.82			T		
LELAND BOWMAN LOCK	4.43					0.10	0.31					0.26	0.12	0.38	0.05	1.65			0.26			0.10		0.05		0.20	0.90	0.05				
MOSS BLUFF	3.07					0.12					T	0.78	0.08	0.42		0.13	0.22					0.53			T		0.71	0.08				
MOSS BLUFF 2 NNW	4.43				0.22					0.03	2.47		0.36		0.13	0.22					0.60					0.40						
OAKDALE	0.95														0.70							0.04	0.06					0.15				
OBERLIN FIRE TWR	1.43											0.02	0.03									0.63					0.17	0.58				
OLD TOWN BAY	M 3.97				-						0.12	0.90	0.11	0.22		T	0.22	0.22				1.00	-	T			0.96	0.22				
ROCKEFELLER WL REFUGE	3.02												0.43		T	0.73	0.04				0.41	0.01	0.10	0.59			0.23	0.48				
SULPHUR	1.04										0.02				0.10						0.19					0.18	0.55					
VINTON 5W	M 2.62											0.13	1.11			0.90								-				0.48				

LOUISIANA
201308

DAILY PRECIPITATION (INCHES)

STATION	TOTAL	01	02	03	04	05	06	07	08	09	10	11	12	13	14	15	16	17	18	19	20	21	22	23	24	25	26	27	28	29	30	31
**SOUTH CENTRAL 08**																																
BAYOU SORREL LOCK	3.00										0.10	1.10	0.02	0.09	0.90	0.06						0.15				0.06	0.50	0.02				
CARENCRO	5.42				1.65								0.86	0.09	0.02	0.63						0.08	1.26				0.16	0.67				
CARVILLE 2 SW	5.37				0.03					0.36	0.52	0.58	0.10	0.27	2.05						1.20		0.16		0.05	0.03	0.02					
DONALDSONVILLE 4 SW	8.38					0.42					1.10		0.07	0.05	1.51	2.90					0.02	0.78				0.12	0.68	0.73				
FRANKLIN 3 NW	3.23				0.30	0.55			0.12			0.17		0.09	1.19						0.03	0.01			0.11	0.36	0.30					
JEANERETTE 5 NW	4.77					0.63					0.33		0.61	0.06	0.50	0.74	0.07					0.03				0.63	0.02	1.15				
LAFAYETTE	3.23						0.05						0.03			1.84						0.01					0.60	0.70				
LAFAYETTE FCWOS	3.76				1.14	T					T	0.14			1.05						0.03	T			0.02	0.09	1.29					
MORGAN CITY	4.13			0.18	0.52	0.01	0.93				0.08	0.35			0.45	0.35	0.08				0.01	0.12	0.30		0.04	0.29	0.02	0.40				
NAPOLEONVILLE	4.09	0.55				0.20				0.05	0.04	0.46	T	0.60	0.65	0.76				0.03	0.01	0.30		0.09	0.09	0.10	0.16	T				
NEW IBERIA AP ACADIANA RGNL	3.99	T	0.21		T					1.14	0.06	0.29	0.04	T	1.18			0.15		0.23	0.01		0.19	T	0.15	T	0.34			T		
PLAQUEMINE 2 N	6.39			0.01							1.04		0.73		2.10	0.30						1.05				0.06	0.50	0.60				
ST GABRIEL	5.63			T							0.21		1.12	0.01	1.72	1.27	0.01					0.69	0.01				0.17	0.42				
ST MARTINVILLE 3 SW	7.13			0.30			0.01				0.16	0.15	0.35	0.08		1.40					0.08	2.98					0.12	1.50				
**SOUTHEAST 09**																																
BOOTHVILLE ASOS	10.92	0.01		0.34					0.06	0.46	0.90	0.91		0.03	1.88		0.33	0.99	2.68			0.18	0.21	0.04	0.73	0.75	0.42					
CONVENT 2S	3.70												0.45		0.96		0.04					0.60	0.25		0.10	T	0.73	0.57				
DUTCHTOWN #2	5.26										0.17		0.18	0.33	1.54	2.03						0.16	0.21	0.14		0.14	0.23	0.13				
GALLIANO	M 5.17				-					0.10	1.25		0.47	0.01	0.27	0.80	0.40	0.10	0.10			0.10	0.09	0.01	0.36	0.68	0.36	0.07				
GONZALES	4.71										0.33		0.03	0.65	1.35	1.44						0.13				0.05	0.57	0.16				
GRAND ISLE	M 9.14				0.50			0.05			0.20	0.20	-		0.60	1.80		2.30	1.09						0.20	1.00	1.20					
HOUMA	M			0.72		0.01					1.26	0.04	0.40	0.81	0.40	0.92	0.62	-	-	-	-	-	-	-	-	-	-	-	-	-	-	-
LUTCHER	3.91										0.01				2.35				T		0.23	0.13	0.62		0.10	0.05	0.42					
MARRERO 9 SSW	M	0.24	0.03	-	-						-	-	-	0.02	0.97	1.20	0.02	-	-	-	0.73	0.08	0.14	0.01	-	-	-	0.02	T			-
NEW ORLEANS AP	7.22	T							0.01	0.47	0.02	0.75	T	1.10	1.33			0.07		0.65	0.30	1.74	T	T	0.17	0.38	0.23					
NEW ORLEANS AUDUBON	3.13	0.14		0.01					0.16	0.03		0.05		0.75	0.76		0.07	0.02		0.32	0.06	0.03		0.08	0.36	0.23	0.06					
NEW ORLEANS ALGIERS	M	-	-	-	-	-	-	-	-	-	-	-	-	-	-	-	-	-	-	-	-	-	-	-	-	-	-	-	-	-	-	-
NEW ORLEANS LKFRNT AP	2.46									0.24		0.05	0.20	0.23	0.27	0.01	T	0.13		0.01	T	0.39	0.16	0.01	0.22	0.37	0.17					
TERRYTOWN 3S	3.86	0.18			0.32						0.02		0.31		0.66	0.77	T	T	0.23		0.40	0.15	0.03		0.35	0.10	0.34					
THIBODAUX 4 SE	6.44	0.08		0.22	T						1.69	0.06	T	0.05	0.39	1.08	0.01	0.10			0.01	0.06	1.07		T	0.07	0.72	0.83				

LOUISIANA
201308

DAILY TEMPERATURES (°F)

STATION	OB.TIME	MAX/MIN	01	02	03	04	05	06	07	08	09	10	11	12	13	14	15	16	17	18	19	20	21	22	23	24	25	26	27	28	29	30	31	AVERAGE
**LOUISIANA**																																		
**NORTHWEST 01**																																		
BENTON 5E	08	MAX	96	93	96	96	97	99	99	97	99	99	97	97	96	92	84	84	82	85	90	90	93	95	95	96	95	94	93	93	97	97	98	94.0
		MIN	75	74	76	75	77	80	80	78	74	76	73	75	77	72	61	62	60	62	67	66	68	72	71	73	72	72	72	72	72	73	74	72.0
MANSFIELD 7 NW	08	MAX																																M
		MIN																																M
MINDEN	07	MAX	93	94	96	93	95	97	97	96	98	98	97	96	95	94	85	84	84	86	89	91	92	94	96	97	95	95	94	94	97	96	98	93.7
		MIN	74	75	76	76	75	76	77	76	73	73	73	73	74	73	63	63	61	61	69	68	68	68	71	73	72	72	71	72	72	70	70	71.2
MOORINGSPORT 1 N	08	MAX	100	96	99	98	97	102	101	101	102	100	98	95	96	96	85	85	83	86	90	92	92		95	97	95	94	93	96	101	99	97	95.4
		MIN	75	75	76	76	78	78	79	77	76	75	72	73	75	72	62	63	60	60	66	65	67		72	71	71	72	72	73	74	73	71	71.6
RED RIVER RSCH STN	07	MAX	94	96	95	94	96	97	98	96	98	98	96	96	97	98	86	88	86	88	91	92	93	95	96	98	96	94	94	95	98	97	97	94.6
		MIN	78	75	77	76	78	77	77	77	73	76	73	75	78	74	64	60	61	64	64	65	70	71	71	73	71	72	70	72	71	70	73	71.8
SHREVEPORT DWTN AP	24	MAX	97	96	96	97	99	100	99	99	99	98	97	97	98	87	87	86	87	91	93	93	96	97	99	96	96	95	95	99	99	98	100	95.7
		MIN	79	75	78	78	80	79	79	79	78	78	73	77	75	71	65	61	61	67	67	67	71	74	73	74	72	73	72	73	73	75	78	73.4
SHREVEPORT AP	24	MAX	97	98	96	98	100	100	99	101	100	99	98	99	99	88	90	89	90	93	94	95	97	99	99	98	97	96	96	102	100	99	102	97.0
		MIN	79	77	77	76	78	78	78	77	75	76	72	75	75	73	67	64	64	68	69	66	71	73	73	75	72	73	72	71	73	71	75	73.0
SHREVEPORT STHRN HILLS	07	MAX	97	97	98	96	98	101	101	100	102	101	100	99	98	101	87	90	88	89	93	94	95	97	99	101	99	97	95	97	101	99	99	97.1
		MIN	74	76	76	75	75	77	76	76	73	73	72	72	74	73	67	63	62	62	67	67	66	70	72	73	72	72	71	70	70	71	70	71.2
SHREVEPORT WFO	24	MAX	97	97	95	97	99	100	99	100	99	99	97	97	99	86	86	86	88	91	91	91	97	99	97	97	95	95	95	97	99	99	100	95.6
		MIN	80	77	77	77	77	78	78	76	74	77	73	75	76	73	67	66	64	68	70	70	71	73	70	76	73	74	73	73	75	72	74	73.5
**NORTH CENTRAL 02**																																		
BIENVILLE 3 NE	23	MAX	95	97	95	97	97	96	96	95	98	97	96	96	95	88	88	88	89	91	91	94	95	97	98	96	94	95	95	97	96	98	99	94.8
		MIN	76	76	77	75	76	79	76	76	72	75	72	75	73	73	62	62	61	65	68	65	69	71	71	73	71	72	68	71	68	69	72	71.3
CALHOUN RSCH STN	08	MAX	93	94	96	94	95	95	96	97	94	97	97	96	94	91	86	86	85		89	91	91	89	95	95	94	94	94	93	95	95	97	93.3
		MIN	72	71	70	71	72	72	70	72	70	68	71	71	71	71	61	59	58		63	63	63	63	63	68	67	67	64	64	64	64	65	66.9
COLUMBIA LOCK	07	MAX	95	95	94	97	96	97	98	98	99	98	96	98	94	93	85	86	85	86	86	90	93	94	94	93	94	93	92	91	91	91		93.1
		MIN	74	73	72	76	76	76	76	76	74	73	73	73	75	72	64	64	65	64	65	68	70	70	70	72	70	69	69	68	64	64		70.5
FARMERVILLE	07	MAX	91	93	94	91	94	94	95	96	96	96	97	96	95	88	84	83	84	84	89	90	91	94	95	96	94	94	95	93	95	96	96	92.5
		MIN	75	73	76	76	76	77	76	75	74	74	73	74	73	77	59	60	61	63	66	66	70	72	73	72	71	71	68	70	73	72	74	71.3
HOMER 1N	07	MAX	92		94	91	93	95	95	94	94	94		93	94	89	83	83	82	83	87	89	90	91	93	95	93	92	91	91	94	93	94	91.1
		MIN	72		71	71	73	75	75	73	69	71		71	72	72	59	57	60	62	64	65	65	68	69	69	69	68	65	67	66	65	68	68.0
MONROE REGIONAL AP	24	MAX	97	97	97	99	97	98	100	98	100	99	98	95	92	88	88	87	88	91	93	94	95	96	97	96	94	95	96	97	96	98	97	95.3
		MIN	76	72	76	73	75	76	76	74	71	72	73	73	71	69	63	61	65	65	65	65	68	69	72	71	69	70	65	66	65	68	71	69.8
RUSTON LA TECH	08	MAX	92	93	94	94	93	94	94	93	92	94	93	93	93	91	84	85	84	86	88	88	90	90	93	91	95	91	90	89	94	92	93	91.2
		MIN	73	74	75	73	74	75	75	74	70	73	71	72	74	73	61	60	62	62	65	65	66	66	70	68	68	69	66	67	66	66	68	69.1
WINNFIELD 3 N	24	MAX																																M
		MIN																																M
**NORTHEAST 03**																																		
BASTROP	07	MAX	90	94	93	92	94	95	97	98	98	98	96	93	93	87	84	83	83	83			93	94	94	95	95	94	93	93	93	94	94	92.5
		MIN	74	72	72	73	74	75	75	74	72	72	73	73	73	73	62	61	61	64			66	70	70	72	70	71	67	66	66	66	69	69.9
LAKE PROVIDENCE	07	MAX	92	93	93	94	91	97	97	96	93	93	93	92	93	87	85	83	85	83	86	86	89	91	92	91	92	89	90	89	90	92	94	90.7
		MIN	77	74	74	75	76	74	74	77	78	76	76	75	75	73	65	64	64	68	68	68	69	73	74	74	72	72	68	68	68	68	72	71.9

LOUISIANA
201308

DAILY TEMPERATURES (°F)

STATION	OB.TIME	MAX/MIN	01	02	03	04	05	06	07	08	09	10	11	12	13	14	15	16	17	18	19	20	21	22	23	24	25	26	27	28	29	30	31	AVERAGE
RAYVILLE	07	MAX	94	94	96	95	96	97	98	99	99	97	96	96	94	91	87	88	88	84	89	92	94	91	95	96	96	93	95	93	94	93	96	93.7
		MIN	76	76	74	77	76	78	78	78	77	76	76	76	76	73	65	64	63	66	69	68	69	73	73	74	73	72	71	69	68	68	71	72.4
ST JOSEPH 3 N	08	MAX												91	95	95	75	75			77	90	93	93		94	95	94	94	91	91	93	94	M
		MIN	78	74	72	74	77	77	77	76	78			74	76	72	66	66	67	70	68	68	71	71		71	71	73	66	63	65	69	72	71.5
TALLULAH	08	MAX										95		92	95	91	85	95		85	85	86	89	95	95	93		95	90	91				M
		MIN										73		73	74	71	65	64		64	68	68	68	64	64	71		64	64	64				M
TALLULAH VICKSBURG RGN	24	MAX	96	96	95	96	96	96	96	95	97	93	94	91	89	87	87	87	85	87	88	92	91	94	95	94	92	92	90	91	93	93	94	92.3
		MIN	74	72	72	72	76	75	74	73	74	72	72	73	73	70	65	63	65	63	65	63	67	68	70	68	67	69	62	60	62	65	66	68.7
WINNSBORO 2 SE	08	MAX	98	95	98	97	100	100	102	101	100	98	99	99	95	95	88	90	89	86	89	90	93	94	94	96	96	94	96	94	94	95	97	95.2
		MIN	75	72	75	72	75	77	77	73	74	73	72	74	75	70	64	64	66	67	66	66	70	70	72	71	70	71	67	65	65	67	69	70.5
WINNSBORO 5 SSE	07	MAX	96	96	97	97	97	99	99	100	99	96	96	96	92	93	88	89	87	86	89	91	92	91	94	96	94	93	93	93	94	94	95	93.9
		MIN	73	71	74	72	74	75	75	74	75	74	72	72	75	70	64	62	65	60	65	65	69	69	71	70	69	70	66	65	64	67	68	69.5
**WEST CENTRAL 04**																																		
HODGES GARDENS	08	MAX			97	97	96	98	100	99	100	99	97	96	97	100	81	88	89	87	90	93	92	94	95	96	94	93	91	93	97	96	97	94.6
		MIN			74	74	75	74	74	73	73	74	71	73	74	72	66	64	62	63	67	68	69	72	73	73	70	71	69	72	73	73	72	71.0
LEESVILLE	08	MAX	98	100	100	96	99	99	99	100	100	100	96	94	95	100	87	90	91	88	92	93	92	95	95	95	93	92	89	93	98	94	98	95.2
		MIN	76	71	71	72	75	76	77	74	69	72	71	71	74	74	67	63	64	66	62	62	68	68	68	70	70	71	67	68	68	67	69	69.7
NATCHITOCHES #2	07	MAX	93	94	94	92	94	95	96	96	96	95	94	93	95	98	82	86	84	86	88	90	90	91	91	92	91	90	88	91	93	92	93	91.7
		MIN	76	75	75	76	76	76	76	75	74	75	73	74	77	72	67	64	62	65	66	68	70	70	72	72	70	71	71	71	70	72	73	71.7
TOLEDO BEND LAKE	08	MAX	97	97	98	96	98	99	98	98	98	99	95	95	95	98	87	88	90	90	93	93	93	96	95	98	97	95	89	94	97	95	97	95.1
		MIN	74	74	74	75	76	76	76	76	72	72	73	74	74	75	69	66	65	65	68	68	68	71	72	74	72	72	71	71	70	73	73	71.9
**CENTRAL 05**																																		
ALEXANDRIA	08	MAX	98	96	99	95	98	98	98	98	97	96	95	97	95	96	96	89	88	90	90	88	91	93	93	94	92	91	89	92	95	94	94	94.0
		MIN	75	73	75	76	76	77	77	75	75	76	75	74	74	73	69	68	66	66	70	69	71	73	73	73	64	72	72	71	71	71	71	72.3
ALEXANDRIA 5 SSE	24	MAX	95	97	96	97	98	99	98	98	98	96	95	91	98	84	88	86	87	87	86	86	91	91	92	90	89	87	90	92	92	93	93	92.3
		MIN	75	73	74	74	74	76	78	73	74	75	73	74	72	72	68	67	67	69	67	68	72	71	73	72	72	72	70	69	68	69	70	71.6
BOYCE 3 WNW	24	MAX	92	93	91	92	96	97	97	98	95	92	91	88	93	80	85	83	84	85	84	86	89	90	91	89	89	86	88	91	90	91	93	90.0
		MIN	77	76	76	78	78	78	78	77	77	76	75	74	73	73	67	66	64	69	69	70	73	71	74	74	72	73	72	72	71	75	74	73.3
BUNKIE	08	MAX	95	94	96	94	96	96	97	97	97	97	94	93	85	97	83	87	88	88	87	87	87	89	92	93	89	89	87	91	91	91	93	91.6
		MIN	76	74	76	74	74	76	76	73	74	76	73	73	76	73	67	67	65	65	68	68	71	71	72	71	71	72	70	69	68	69	69	71.5
EUNICE	08	MAX	96	96	98	98	97	97	98	98	97	98	90	92	92	96	88	90	90	89	90	87	86	92	94	96	91	87	87	93	94	94	93	93.0
		MIN	74	74	75	75	75	75	74	73	75	75	72	74	74	73	68	68	65	67	67	70	70	70	73	72	72	73	70	70	71	70	70	71.7
GRAND COTEAU	17	MAX	94	95	96	94	93	95	95	94	95	93	90	88	96	91	90	89	88	88	86	89	92	92	93	90	88		89	89	90	93	93	91.6
		MIN	75	77	75	74	75	74	74	73	75	75	73	73	74	70	70	67	70	69	68	70	70	72	72	72	71		70	68	68	69	69	71.7
JENA 4 WSW	08	MAX	92	93	96	92	95	96	96	97	97	97	96	96	90	97	84	86				87	90	90	92	94	91	91	89	91	92	92	94	92.6
		MIN	72	73	74	73	72	74	76	72	73	73	71	72	74	71	65	63				65	66	69	70	70	69	70	69	66	65	68	69	70.1
JONESVILLE LOCKS	06	MAX	93	93	93	94	92	94	95	94	94	96	93	92	88	93	84	87	86	86	87	85	87	89	89	91	93	91	89	91	92	92	92	90.8
		MIN	74	77	77	78	77	76	77	78	77	76	76	75	76	72	68	67	67	66	70	71	71	74	74	74	74	72	73	70	69	70	70	73.1
LSU DEAN LEE RSCH STN	08	MAX	94	95			97	97	98	97	96			97	90	95	84				87	86	88	90	90			92	88	91	93			M
		MIN	77	73			74	77	78	73	73			73	74	72	68				66	68	71	71	73			71	71	69	68			M
NEW ROADS 5 NE	24	MAX	92	94	96	94	97	99	98	100	94	96	87	93	91	80	86	88	86	87	84	93	94	94	92	90	90	91	89	91	90	88	95	91.6
		MIN	75	75	76	76	76	77	77	76	77	75	75	74	71	71	70	71	70	69	71	72	73	73	74	73	72	72	71	68	67	69	70	72.8

LOUISIANA
201308

DAILY TEMPERATURES (°F)

STATION	OB.TIME	MAX/MIN	01	02	03	04	05	06	07	08	09	10	11	12	13	14	15	16	17	18	19	20	21	22	23	24	25	26	27	28	29	30	31	AVERAGE
**EAST CENTRAL 06**																																		
BATON ROUGE METRO AP	24	MAX	93	95	94	96	94	94	95	95	93	96	90	91	91	79	89	90	88	85	88	92	91	92	92	86	88	89	89	89	90	92	92	90.9
		MIN	76	73	75	76	77	76	77	76	77	76	75	75	71	72	71	71	71	70	71	72	72	73	73	73	72	73	69	66	66	69	70	72.7
BOGALUSA	08	MAX																																M
		MIN																																M
CLINTON 5 SE	08	MAX	91	91	93	93	94	92	92	92	95	94	88	94	92	89	82	86	84	81	88	85	89	86	89	83	85	89	86	86	86	88	91	88.8
		MIN	74	70	72	74	72	74	73	72	74	73	71	71	72	70	69	69	68	68	68	69	70	71	71	70	70	70	67	62	62	65	67	69.9
HAMMOND 5 E	08	MAX	94	93	95	94	96	96	96	95	95	90	92	84	92	90	80	88	94	85	80	89	91	86	91	89	84	85	85	87	87	91	92	89.9
		MIN	76	71	71	72	74	74	74	74	74	74	72	72	71	72	70	70	71	71	71	69	71	70	73	71	72	72	65	61	64	65	67	70.8
LSU BEN-HUR FARM	08	MAX	92	92	93	96	97	93	94	94	94	91	93	87	90	90	76	89	90	87	86	91	93	89	91	91	84	85	86	90	90	90	90	90.1
		MIN	76	73	73	75	75	76	76	75	76	75	74	74	75	69	70	71	72	70	70	71	72	73	72	72	72	73	68	65	65	68	69	72.1
SLIDELL	08	MAX	90	92	91	93	93	93	92	93	92	91	91	89	90	90	78	86	87	81	81	87	89	88	89	85	79	83	86	87	87	87	89	88.0
		MIN	76	72	74	76	75	77	77	79	77	75	76	75	74	74	71	72	72	71	70	72	75	72	72	73	73	72	67	65	66	73	73	73.1
SLIDELL AP	24	MAX	93	94	95	96	94	93	93	93	92	93	88	92	89	79	88	90	83	82	88	90	89	92	87	81	84	87	88	89	88	89	91	89.4
		MIN	75	72	73	75	76	77	77	78	77	75	75	74	74	73	72	72	73	73	71	71	74	72	73	73	74	72	67	64	66	69	72	72.9
**SOUTHWEST 07**																																		
CROWLEY 2 NE	08	MAX	95	96	96	95	98	95	96	96	96	92	89	96	88	97	85	88	89	89	90	87	85	90	92	93	87	85	85	90	92	91	93	91.5
		MIN	75	77	74	73	73	75	72	75	72	73	74	73	75	74	70	69	69	68	67	71	71	72	73	73	73	73	71	69	69	68	69	71.9
DE RIDDER	08	MAX	93	91	94	97	95	100	98	95	95	95	94	95	94	97	87	89	92	90	91	94	88	94	95	96	92	90	85	91	95	96	95	93.3
		MIN	69	69	71	69	70	73	71	70	70	70	70	71	77	74	67	67	66	69	67	68	71	70	72	72	71	72	70	72	71	71	71	70.4
HACKBERRY 8 SSW	08	MAX	91	94	91	91	90	91	91	92	91	94	91	91	89	93	89	84	91	91	89	90	86	89	92	93	91	82	79	87	92	88	89	89.7
		MIN	76	76	79	80	78	77	77	77	78	78	77	80	81	81	74	73	71	72	74	74	73	75	78	75	75	75	73	74	75	76	76	76.1
JENNINGS	08	MAX	95	96	97	95	96	97	98	98	97	96	94	90	89	95	87	87	92	90	90	87	87	91	94	95	90	86	84	92	93	91	93	92.3
		MIN	74	76	76	76	77	76	75	74	75	76	75	75	76	75	72	70	68	68	70	69	73	71	74	73	74	73	72	71	72	71	71	73.2
LAKE ARTHUR 10 SW	08	MAX	96	97	95	94	94	96	96	96	96	93	90	90	91	97	87	84	92	92	91	90	88	92	96	92	85	85	81	91	92	91	95	91.8
		MIN	77	77	78	78	78	77	78	76	76	76	76	77	77	78	73	70	67	71	72	72	73	73	74	74	74	74	72	72	72	72	73	74.4
LAKE CHARLES AP	24	MAX	97	96	95	94	96	96	96	96	98	94	93	90	98	92	88	94	94	94	93	85	93	95	97	94	87	84	93	96	91	92	92	93.3
		MIN	76	78	76	78	77	79	77	75	77	77	76	76	77	75	73	73	71	72	71	74	73	75	75	74	75	74	72	73	75	73	73	74.8
LELAND BOWMAN LOCK	08	MAX			94	95	98	98	98	98	99		92	89	94	98	91	90	94	91	90	90	92	92	94	97	89	84	85	93	94	91	94	93.0
		MIN			76	78	79	79	77	75	75			78	80	77	73	72	69	65	67	67	67	66	75	73	73	73	73	74	74	73	74	73.4
MOSS BLUFF 2 NNW	24	MAX	97	99	98	96	98	100	99	99	99	95	92	90	97	90	88	92	91	93	92	81	92	93	96	92	87	83	92	95	94	94	95	93.5
		MIN	73	73	73	74	74	76	77	73	72	72	73	74	74	75	69	69	66	70	66	70	70	72	72	72	73	73	69	69	72	70	70	71.8
OBERLIN FIRE TWR	09	MAX	94	95	97	95	95	96	97	97	96	99	88	93	88	96	90	88	91	89	91	90	85	91	92	94	91	88	86	90	94	94	93	92.4
		MIN	74	74	74	74	74	74	74	72	73	75	73	73	75	74	69	68	66	67	66	68	70	71	71	72	73	73	70	70	72	70	70	71.6
ROCKEFELLER WL REFUGE	08	MAX	89	90	90	90	90	90	90	91	91	93	90	89	89	91	87	85	91	90	90	89	88	88	90	93	88	80	80	89	92	87	90	89.0
		MIN	79	79	78	78	81	81	80	74	75	76	73	76	77	77	71	71	69	69	70	72	72	73	74	72	74	72	71	71	72	70	73	74.2
VINTON 5W	08	MAX	95	98	98	97	96	97	97	97	97	100	97	92	94	96	91	87	91	91	93	95	85	97		97	95	85	81	92	97	97	95	94.0
		MIN	72	75	74	75	74	74	75	72	73	75	74	73	75	78	71	69	66	68	68	70	70	72		72	74	72	70	72	74	72	71	72.3
**SOUTH CENTRAL 08**																																		
CARVILLE 2 SW	24	MAX	91	93	93	93	91	93	93	94	95	92	86	92	86	80	87	87	89	89	86	91	85	90	89	83	82	82	88	87	88	92	91	89.0
		MIN	78	77	76	77	78	78	78	78	78	77	77	77	73	73	73	74	72	71	72	73	74	74	75	74	73	74	70	69	69	75	73	74.5
DONALDSONVILLE 4 SW	08	MAX	91	91	94	93	93	92	93	93	95	92	92	89	93	87	80	87	89	86	83	87	91	89	90	89	84	85	85	87	86	88	89	89.1

LOUISIANA
201308

DAILY TEMPERATURES (°F)

STATION	OB.TIME	MAX/MIN	01	02	03	04	05	06	07	08	09	10	11	12	13	14	15	16	17	18	19	20	21	22	23	24	25	26	27	28	29	30	31	AVERAGE
		MIN	75	73	73	73	74	75	74	75	75	74	74	75	75	72	72	72	72	70	70	71	71	72	73	72	73	73	69	67	65	66	69	72.1
FRANKLIN 3 NW	24	MAX	92	91	91	90	90	91	92	92	90	90	90	91	86	82	86	88	87	84	86	90	89	90	90	82	81	87	88	87	87	90	90	88.4
		MIN	76	78	75	77	76	77	76	77	77	76	76	76	73	73	72	73	72	71	71	71	73	73	73	72	74	73	72	69	68	71	72	73.6
JEANERETTE 5 NW	08	MAX	92	93	93	93	91	92	93	94	93	93	93	93	89	90	81	87	89	88	85	88	89	90	90	91	91	91	86	87	88	88	91	90.1
		MIN	75	76	75	75	75	76	76	74	74	75	76	75	75	73	71	71	69	69	69	69	72	72	73	71	71	71	71	69	67	68	69	72.3
LAFAYETTE	22	MAX	94	96	96	95	94	94	94	95	95	92	91	92	91	95	85	89	91	89	90	90	91	92	92	93	92	86	86	92	91	92	92	91.8
		MIN	76	77	79	78	78	78	76	75	75	77	75	75	75	75	74	68	68	71	70	71	73	73	74	74	74	73	73	73	71	71	73	74.0
LAFAYETTE FCWOS	24	MAX	96	96	95	95	93	95	95	95	95	92	89	94	96	84	89	90	89	88	88	89	93	93	94	88	85	88	91	90	91	91	93	91.6
		MIN	77	80	77	77	79	78	76	75	77	77	75	76	74	74	74	72	69	71	70	73	73	74	75	73	73	74	73	72	71	72	73	74.3
MORGAN CITY	08	MAX	91	87	93	94	91	93	94	95	91	93	92	89	93	88	84	90	88	86	84	84	89	91	88	90	83	83	86	88	92	94	91	89.5
		MIN	77	76	76	75	75	77	79	78	77	78	76	75	76	75	73	72	75	72	72	71	71	74	74	75	75	73	73	71	70	71	72	74.3
NEW IBERIA AP ACADIANA RGNL	24	MAX	96	97	96	95	94	96	97	96	96	93	89	91	95	85	91	93	92	90	92	93	94	95	95	87	87	89	93	93	94	95	96	93.1
		MIN	77	78	77	78	76	77	75	76	78	79	78	78	76	75	74	74	72	73	73	76	75	76	77	76	77	76	76	73	72	73	74	75.6
ST MARTINVILLE 3 SW	08	MAX	92	93	94	94	94	91	95	95	95	94	92	86	89	92	82	87	88	88	86	88	88	90	91	91	84	83	86	87	90	88	91	89.8
		MIN	74	75	74	74	74	75	74	72	75	74	74	74	74	74	72	69	68	69	68	71	71	71	73	71	73	74	71	68	67	68	69	71.9
**SOUTHEAST 09**																																		
BOOTHVILLE ASOS	24	MAX	91	90	88	91	91	91	92	91	88	89	88	89	89	86	85	84	80	82	86	87	88	86	83	80	80	85	86	86	87	88	87	86.9
		MIN	78	77	82	81	81	81	80	78	76	75	75	81	78	73	75	77	72	75	78	79	76	77	76	74	73	77	75	73	75	76	78	76.8
GALLIANO	08	MAX	92	92	91		92	92	93	93	94	89	89	87	89	92	86	88	87	82	85	88	89	86	87	84	79	79	85	86	85	87	90	87.9
		MIN	78	77	76		78	81	81	79	77	76	77	77	78	73	73	74	72	75	74	75	75	73	77	75	75	74	74	69	69	70	74	75.2
HOUMA	08	MAX	92	91	91	90	93	91	93	93	90	85	89	89	88	87	85	87																M
		MIN	73	73	73	76	76	75	75	76	76	74	74	74	74	71	72	72																M
MARRERO 9 SSW	08	MAX	93	93			95	95	95	96	93			95	91	91	82	89			88	89	90	88	89			82	86	88	88	90		90.3
		MIN	76	75			75	77	77	77	76			74	76	70	72	74			71	73	75	73	74			73	72	68	68	70		73.5
NEW ORLEANS AP	24	MAX	93	95	94	96	95	96	97	95	92	94	90	92	91	82	89	91	86	83	87	88	88	90	88	82	84	86	87	87	89	91	92	90.0
		MIN	77	77	76	77	78	78	79	79	80	79	76	78	74	74	74	76	77	75	71	73	75	73	78	75	75	74	73	69	71	72	75	75.4
NEW ORLEANS AUDUBON	24	MAX	93	95	94	96	96	96	97	94	93	94	95	94	82		91	92	85	85	90	90	89	90	89	81	84	86	90	90	91	92	95	91.0
		MIN	78	76	76	78	78	78	78	78	79	81	78	79	72		73	76	75	73	72	75	76	73	76	73	75	75	72	70	70	72	75	75.3
NEW ORLEANS LKFRNT AP	24	MAX	92	95	95	93	95	95	96	95	93	94	91	92	90	82	88	90	84	82	87	90	89	91	88	83	85	86	89	89	88	91	92	90.0
		MIN	82	80	82	84	84	84	84	84	79	81	80	77	75	76	75	79	77	76	74	76	76	77	80	76	77	78	77	75	77	79	81	78.8
TERRYTOWN 3S	07	MAX	92	95	94	94	97	96	97	95	93	92	93	92	93	92	80	90	90	83	84	89	90	90	90	89	79	82	86	90	89	90	92	90.3
		MIN	77	77	77	77	78	78	77	78	79	77	79	76	78	72	73	75	76	74	72	74	76	74	77	74	74	74	73	69	69	72	74	75.2
THIBODAUX 4 SE	08	MAX	89	92	94	90	91	93	94	94	93	89	90	84	90	86	85	87	88	84	83	88	89	87	87	86	79	80	85	86	86	88	89	87.9
		MIN	75	77	71	74	74	74	75	75	75	73	72	73	73	70	72	72	72	71	69	70	71	71	72	71	73	72	69	66	65	67	70	71.7

LOUISIANA
201308

DAILY SOIL TEMPERATURES

STATION	DEPTH	TIME	01	02	03	04	05	06	07	08	09	10	11	12	13	14	15	16	17	18	19	20	21	22	23	24	25	26	27	28	29	30	31	AVERAGE
**LOUISIANA**																																		
**NORTHWEST 01**																																		
RED RIVER RSCH STN (IN)																																		
BARE GROUND	4	MAX	86	86	87	88	88	88	88	88	87	88	88	88	88	87	86	85	84	83	83	83	83	84	85	85	86	84	84	85	98	85	85	86.2
	4	MIN	82	83	83	85	84	85	86	85	85	84	84	85	85	85	84	83	82	81	81	81	81	82	82	83	83	83	83	83	71	83	83	82.9
**NORTH CENTRAL 02**																																		
CALHOUN RSCH STN (IN)																																		
SOD	4	MAX	88	91	92	90	91	92	93	94	92	92	90	89	90	88	86	86	84	-	86	86	86	86	88	88	88	88	86	87	87	87	88	88.6
	4	MIN	83	82	84	85	84	83	83	83	81	83	83	81	82	82	79	76	73	-	77	77	77	77	78	77	79	80	78	77	77	78	77	79.9
**NORTHEAST 03**																																		
ST JOSEPH 3 N (IN)																																		
BARE GROUND	2	MAX	102	101	104	105	106	108	109	106	104	-	-	111	103	101	92	88	93	98	100	100	108	101	-	106	108	106	107	104	105	109	104	103.2
	2	MIN	81[I]	77	84	85	85	86	85	87	63	-	-	87	85	80	76	70	76	79	81	78	79	82	-	79	81	82	82	80	81	77	82	80.3
WINNSBORO 2 SE (IN)																																		
BARE GROUND	2	MAX	90	89	90	89	91	91	90	100	100	89	88	89	95	92	86	85	84	81	83	84	85	86	87	87	85	86	86	85	86	85	87	88.1
	2	MIN	79	79	79	79	79	80	81	81	83	80	79	79	80	78	73	73	74	73	73	73	74	76	76	77	76	77	75	75	74	74	75	76.9
WINNSBORO 5 SSE (IN)																																		
BARE GROUND	4	MAX	89	90	95	92	94	95	96	98	97	96	95	91	89	86	83	85	85	85	90	89	91	87	91	91	91	93	92	93	91	94	93	91.2
	4	MIN	78	80	79	80	80	81	81	83	84	81	81	81	80	77	73	72	73	74	75	75	75	77	75	77	77	78	78	77	76	76	77	77.8
**CENTRAL 05**																																		
LSU DEAN LEE RSCH STN (IN)																																		
**EAST CENTRAL 06**																																		
CLINTON 5 SE (IN)																																		
HAMMOND 5 E (IN)																																		
**SOUTHWEST 07**																																		
CROWLEY 2 NE (IN)																																		
JENNINGS (IN)																																		

(Column group "DAY OF MONTH" spans 01–31.)

SOILS REFERENCE NOTES

STATION	SOIL TYPE	SOIL COVER	SLOPE	UNITS
RED RIVER RSCH STN	SANDY LOAM	BARE GROUND	00	F
CALHOUN RSCH STN	FINE SANDY LOAM	BARE GROUND	00	F
ST JOSEPH 3 N	SHARKEY CLAY	BARE GROUND	LEVEL	F
WINNSBORO 2 SE	SANDY LOAM	BARE GROUND	0	F
WINNSBORO 5 SSE	SANDY LOAM	BARE GROUND	00	F
LSU DEAN LEE RSCH STN	SANDY	BARE GROUND	00	F
CLINTON 5 SE	FINE SANDY LOAM	BARE GROUND	LEVEL NNW	F
HAMMOND 5 E	SANDY LOAM	BARE GROUND	00	F
CROWLEY 2 NE	SAND	SOD	1 DEG S	F
JENNINGS	SILT CLAY LOAM	SOD	0	F

SNOWFALL AND SNOW ON GROUND (INCHES)

STATION		01	02	03	04	05	06	07	08	09	10	11	12	13	14	15	16	17	18	19	20	21	22	23	24	25	26	27	28	29	30	31
**LOUISIANA NORTHWEST 01**																																
BENTON 5E	SNOWFALL																															
	SN ON GND																															
HOSSTON	SNOWFALL																															
	SN ON GND																															
JAMESTOWN	SNOWFALL																															
KEITHVILLE	SNOWFALL																															
	SN ON GND																															
KORAN	SNOWFALL																															
LOGANSPORT	SNOWFALL																															
	SN ON GND																															
MANSFIELD 7 NW	SNOWFALL																															
MINDEN	SNOWFALL																															
	SN ON GND																															
MOORINGSPORT 1 N	SNOWFALL																	-	-	-	-	-			-				-		-	-
	SN ON GND																	-	-	-	-	-			-				-		-	-
RED RIVER RSCH STN	SNOWFALL														-																	
SHREVEPORT DWTN	SNOWFALL																															
	SN ON GND																															
SHREVEPORT DWTN AP	SNOWFALL																															
SHREVEPORT AP	SNOWFALL																															
	SN ON GND																															
SHREVEPORT STHRN HILLS	SNOWFALL																															
	SN ON GND																															
SHREVEPORT WFO	SNOWFALL																															
	SN ON GND																															
SPRINGHILL	SNOWFALL																															
VIVIAN	SNOWFALL																															

Snowfall: Includes snow and ice. Values for NWS stations (J index note) are Mid-Mid (LST).
Snow on ground: Includes snow, sleet, ice, and hail. Values for NWS stations (J index note) are observed at 12 UTC (GMT).
Water Equivalent: Given for NWS stations (J index note) only, when snow depth is 2 inches or more, and is measured at 18 UTC (GMT)

LOUISIANA
201308

PAN EVAPORATION AND WIND

STATION		01	02	03	04	05	06	07	08	09	10	11	12	13	14	15	16	17	18	19	20	21	22	23	24	25	26	27	28	29	30	31	TOTAL OR AVERAGE
LOUISIANA																																	
NORTHWEST 01																																	
RED RIVER RSCH STN	WIND	43	39	43	30	33	49	51	49	46	53	47	46	44	37	32	24	22	23	26	63	36	39	35	29	54	80	66	21	39	31	39	1269
	EVAP	0.30	0.32	0.30	0.27	0.30	0.33	0.41	0.34	0.36	0.34	0.34	0.35	0.32	0.18	0.20	0.29	0.22	0.26	0.24	0.24	0.29	0.30	0.30	0.26	0.30	0.33	0.36	0.20	0.30	0.26	0.34	9.15
	MAX	-	-	-	-	-	-	-	-	-	-	-	-	-	-	-	-	-	-	-	-	-	-	-	-	-	-	-	-	-	-	-	M
	MIN	-	-	-	-	-	-	-	-	-	-	-	-	-	-	-	-	-	-	-	-	-	-	-	-	-	-	-	-	-	-	-	M
NORTH CENTRAL 02																																	
CALHOUN RSCH STN	WIND	-	-	-	-	-	-	-	-	-	-	-	-	-	-	-	-	-	-	-	-	-	-	-	-	-	-	-	-	-	-	-	M
	EVAP	-	-	-	-	-	-	-	-	-	-	-	-	-	-	-	-	-	-	-	-	-	-	-	-	-	-	-	-	-	-	-	M
	MAX	-	-	-	-	-	-	-	-	-	-	-	-	-	-	-	-	-	-	-	-	-	-	-	-	-	-	-	-	-	-	-	-
	MIN	-	-	-	-	-	-	-	-	-	-	-	-	-	-	-	-	-	-	-	-	-	-	-	-	-	-	-	-	-	-	-	M
NORTHEAST 03																																	
ST JOSEPH 3 N	WIND	11	9	-	-	-	-	125	17	22	-	-	-	45	32	7	4	-	-	-	3	6	7	-	-	-	-	5	4	3	2	-	M
	EVAP	0.17	0.24	0.00	0.00	0.00	0.33	0.28	0.00	0.00	-	-	0.00	0.00	0.00	0.00	0.00	0.00	0.00	0.00	0.00	0.00	0.00	-	0.00	0.00	0.00	0.00	0.00	0.00	0.00	0.00	1.13E
	MAX	-	-	-	-	-	-	-	-	-	-	-	-	-	-	-	-	-	-	-	-	-	-	-	-	-	-	-	-	-	-	-	-
	MIN	-	-	-	-	-	-	-	-	-	-	-	-	-	-	-	-	-	-	-	-	-	-	-	-	-	-	-	-	-	-	-	M
WEST CENTRAL 04																																	
TOLEDO BEND LAKE	WIND	53	32	56	46	60	88	108	106	84	68	59	66	61	78	26	30	39	28	28	55	65	59	32	49	72	90	83	42	65	49	62	1839
	EVAP	0.31	0.22	0.39	0.34	0.38	0.40	0.39	0.49	0.41	0.43	0.26	0.30	0.28	0.46	0.07	0.27	0.25	0.30	0.28	0.34	0.30	0.33	0.25	0.29	0.41	0.34	0.16	0.34	0.33	0.26	0.37	9.95
	MAX	-	-	-	-	-	-	-	-	-	-	-	-	-	-	-	-	-	-	-	-	-	-	-	-	-	-	-	-	-	-	-	-
	MIN	-	-	-	-	-	-	-	-	-	-	-	-	-	-	-	-	-	-	-	-	-	-	-	-	-	-	-	-	-	-	-	M
SOUTHWEST 07																																	
JENNINGS	WIND	58	37	42	51	40	73	75	66	49	48	34	39	29	74	47	17	35	33	37	42	32	51	33	75	84	62	39	28	32	30	29	1421
	EVAP	0.37	0.16	0.37	0.26	0.25	0.34	0.34	0.34	0.28	0.26	0.29	0.16	0.17	0.29	0.14	0.08	0.29	0.26	0.27	0.25	0.12	0.20	0.89	0.43	0.24	0.11	0.11	0.22	0.19	0.19	0.23	8.10
	MAX	98	102	103	99	101	99	101	100	101	99	98	92	89	96	86	90	98	96	98	99	87	94	81	99	90	86	84	96	96	95	99	99
	MIN	73	74	76	76	76	75	74	75	73	75	75	74	75	75	71	71	68	67	71	72	72	71	76	73	77	72	71	71	72	72	72	73.1

Evaporation: Is measured in hundreths of inches.
Wind: Is measured in miles.
Max and Min: The maximum and minimum tempertures (Fahrenheit) of the water in the evaporation pan.

STATION INDEX

STATION	INDEX NO.	DIVISION	COUNTY	LATITUDE	LONGITUDE	ELEVATION (IN FEET)	OBSERVATION TIME AND TABLES LOCAL STD TIME TEMP	PRECIP	EVAP	SPECIAL SEE (NOTES)
LOUISIANA										
ABBEVILLE	0007	07	VERMILION	29 58	92 7W	10		08		H
ABITA RVR COVINGTON	0012	06	ST. TAMMANY	30 28	90 6W	3		07		H
ABITA SPRING FIRE TWR	0021	06	ST. TAMMANY	30 26	90 3W	30		13		H
ABITA SPRINGS 1 SW	0016	06	ST. TAMMANY	30 28	90 3W	25		07		H
ALEXANDRIA	0098	05	RAPIDES	31 19	92 28W	87	08	08		H
ALEXANDRIA 5 SSE	0103	05	RAPIDES	31 15	92 27W	85	24	24		CH
ANGIE	0238	06	WASHINGTON	30 58	89 49W	130		08		H
ARCADIA	0277	02	BIENVILLE	32 33	92 55W	400		08		H
BAKER	0462	06	EAST BATON ROUGE	30 34	91 10W	70		08		H
BASTROP	0537	03	MOREHOUSE	32 44	91 55W	150	07	07		H
BATON ROUGE CONCORD	0548	06	EAST BATON ROUGE	30 25	91 8W	50		08		H
BATON ROUGE METRO AP R	0549	06	EAST BATON ROUGE	30 32	91 9W	64	24	24		HJ
BATON ROUGE SHERWOOD	0558	06	EAST BATON ROUGE	30 27	91 3W	55		08		H
BAYOU SORREL LOCK	0565	08	IBERVILLE	30 8	91 19W	15		08		H
BEAVER FIRE TWR	0617	05	EVANGELINE	30 48	92 30W	105		13		H
BELL CITY 13 SW	0658	07	CAMERON	29 58	93 5W	4		07		H
BENTON 5E	0718	01	BOSSIER	32 27	93 50W	200	08	08		H
BIENVILLE 3 NE	0800	02	BIENVILLE	32 22	92 57W	307	23	23		H
BOGALUSA	0945	06	WASHINGTON	30 47	89 51W	100	08	08		H
BOOTHVILLE ASOS R	1157	09	PLAQUEMINES	29 20	89 24W	3	24	24		H
BOYCE 3 WNW	1232	05	RAPIDES	31 24	92 43W	110	24	24		H
BUNKIE	1287	05	AVOYELLES	30 58	92 11W	80	08	08		CH
CALHOUN RSCH STN	1411	02	OUACHITA	32 31	92 21W	180	08	08	08	GCH
CARENCRO	1535	08	LAFAYETTE	30 19	92 3W	50		07		H
CARVILLE 2 SW	1565	08	IBERVILLE	30 12	91 8W	25	24	24		H
CLAYTON	1866	05	CONCORDIA	31 43	91 32W	73		07		CH
CLINTON 5 SE	1899	06	EAST FELICIANA	30 49	90 58W	200	08	08		GCH
CLINTON FORESTRY HQ	1891	06	EAST FELICIANA	30 51	91 1W	250		13		H
COLUMBIA LOCK	1979	02	CALDWELL	32 10	92 6W	80	07	07		H
CONVENT 2S	2002	09	ST. JAMES	29 60	90 49W	25		08		H
COVINGTON 3 NE	2154	06	ST. TAMMANY	30 31	90 5W	25		07		H
CROWLEY 2 NE	2212	07	ACADIA	30 14	92 21W	25	08	08		G H
DE RIDDER	2367	07	BEAUREGARD	30 51	93 17W	190	08	08		H
DENHAM SPRINGS	2350	06	LIVINGSTON	30 29	90 58W	35		07		H
DONALDSONVILLE 4 SW	2534	08	ASSUMPTION	30 4	91 2W	30	08	08		CH
DRY CREEK 8NW	2641	07	BEAUREGARD	30 44	93 8W	95		07		H
DUTCHTOWN #2	2688	09	ASCENSION	30 15	90 59W	18		07		H
EUNICE	2981	05	ST. LANDRY	30 29	92 26W	50	08	08		H
FARMERVILLE	3079	02	UNION	32 47	92 24W	180	07	07		H
FRANKLIN 3 NW	3313	08	ST. MARY	29 49	91 33W	12	24	24		H
GALLIANO	3433	09	LAFOURCHE	29 28	90 18W	5	08	08		H
GONZALES	3695	09	ASCENSION	30 12	90 55W	10		07		H
GORUM FIRE TWR	3741	04	NATCHITOCHES	31 26	92 53W	307		13		H
GRAND COTEAU	3800	05	ST. LANDRY	30 25	92 3W	55	17	17		H
GRAND ISLE	3807	09	JEFFERSON	29 14	89 59W	2		07		H
HACKBERRY 8 SSW	3979	07	CAMERON	29 53	93 24W	6	08	08		H
HAMMOND 5 E	4030	06	TANGIPAHOA	30 30	90 23W	35	08	08		GCH
HODGES GARDENS	4288	04	SABINE	31 22	93 23W	420	08	08		H
HOMER 1N	4355	02	CLAIBORNE	32 49	93 4W	215	07	07		H
HOSSTON	4398	01	CADDO	32 27	93 50W	246		08		H
HOUMA	4407	09	TERREBONNE	29 38	90 49W	8	08	08		H
JAMESTOWN	4592	01	BIENVILLE	32 21	93 12W	190		07		H
JEANERETTE 5 NW	4674	08	IBERIA	29 58	91 43W	20	08	08		H
JENA 4 WSW	4696	05	LA SALLE	31 38	92 12W	210	08	08		CH
JENNINGS	4700	07	JEFFERSON DAVIS	30 12	92 40W	25	08	08	08	GCH
JONESBORO 4 ENE	4732	02	JACKSON	32 15	92 39W	330		13		H
JONESVILLE LOCKS	4739	05	CATAHOULA	31 29	91 52W	70	06	06		CH
KAPLAN	4775	07	VERMILION	29 60	92 17W	15		07		H
KEITHVILLE	4816	01	CADDO	32 21	93 52W	200		07		H
KILLIAN	4878	06	LIVINGSTON	30 22	90 33W	10		08		H
KORAN	4931	01	BOSSIER	32 25	93 28W	175		08		H
LAFAYETTE	5021	08	LAFAYETTE	30 13	92 4W	25	22	22		CH
LAFAYETTE FCWOS R	5026	08	LAFAYETTE	30 12	91 59W	38	24	24		H
LAKE ARTHUR 10 SW	5065	07	CAMERON	30 0	92 47W	10	08	08		H
LAKE CHARLES 2 N	5074	07	CALCASIEU	30 15	93 13W	5		08		H

STATION INDEX

STATION	INDEX NO.	DIVISION	COUNTY	LATITUDE	LONGITUDE	ELEVATION (IN FEET)	OBSERVATION TIME AND TABLES LOCAL STD TIME TEMP	PRECIP	EVAP	SPECIAL SEE (NOTES)
LAKE CHARLES 7 NW	5072	07	CALCASIEU	30 18	93 16W	10		08		H
LAKE CHARLES AP R	5078	07	CALCASIEU	30 7	93 14W	9	24	24		HJ
LAKE CHARLES PORT	5076	07	CALCASIEU	30 13	93 15W	5		08		H
LAKE PROVIDENCE	5090	03	EAST CARROLL	32 48	91 10W	100	07	07		H
LEESVILLE	5266	04	VERNON	31 8	93 14W	28	08	08		H
LEESVILLE 6 SSW	5287	04	VERNON	31 3	93 17W	260		08		CH
LELAND BOWMAN LOCK	5296	07	VERMILION	29 47	92 12W	40	08	08		H
LIVERPOOL 6W	5430	06	ST. HELENA	30 56	90 34W	250		08		H
LIVINGSTON	5438	06	LIVINGSTON	30 31	90 45W	43		06		H
LOGANSPORT	5522	01	DE SOTO	31 58	94 0W	190		07		H
LSU BEN-HUR FARM	5620	06	EAST BATON ROUGE	30 22	91 10W	21	08	08		CH
LSU DEAN LEE RSCH STN	5630	05	RAPIDES	31 11	92 25W	70	08	08		G H
LUTCHER	5783	09	ST. JAMES	30 2	90 42W	20		07		H
MANSFIELD 7 NW	5875	01	DE SOTO	32 8	93 45W	255	08	08		CH
MANY 9 WSW	5896	04	SABINE	31 31	93 37W	286		07		H
MARKSVILLE	5920	05	AVOYELLES	31 8	92 4W	85		08		H
MARRERO 9 SSW	5926	09	JEFFERSON	29 47	90 7W	3	08	08		H
MINDEN	6244	01	WEBSTER	32 36	93 18W	185	07	07		CH
MONROE DELTA CC	6314	02	OUACHITA	32 30	92 2W	70		08		CH
MONROE REGIONAL AP R	6303	02	OUACHITA	32 31	92 2W	79	24	24		H
MOORINGSPORT 1 N	6364	01	CADDO	32 42	93 58W	200	08	08		H
MORGAN CITY	6394	08	ST. MARY	29 41	91 11W	5	08	08		CH
MOSS BLUFF	6431	07	CALCASIEU	30 18	93 12W	19		07		H
MOSS BLUFF 2 NNW	6434	07	CALCASIEU	30 20	93 13W	25	24	24		H
MOUNT HERMON 2W	6466	06	WASHINGTON	30 57	90 18W	320		08		H
NAPOLEONVILLE	6561	08	ASSUMPTION	29 56	91 1W	25		07		H
NATCHITOCHES #2	6584	04	NATCHITOCHES	31 49	93 5W	141	07	07		CH
NEW IBERIA AP ACADIANA RGNL R	6657	08	IBERIA	30 2	91 53W	24	24	24		H
NEW ORLEANS ALGIERS	6666	09	ORLEANS	29 57	90 3W	2		08		H
NEW ORLEANS AP R	6660	09	JEFFERSON	29 60	90 15W	4	24	24		HJ
NEW ORLEANS AUDUBON R	6664	09	ORLEANS	29 55	90 8W	20	24	24		H
NEW ORLEANS LKFRNT AP R	6667	09	ORLEANS	30 3	90 2W	9	24	24		H
NEW ROADS 5 NE	6686	05	POINTE COUPEE	30 44	91 22W	45	24	24		H
NORWOOD	6808	06	EAST FELICIANA	30 58	91 6W	102		08		H
OAK GROVE	6866	03	WEST CARROLL	32 52	91 23W	129		08		H
OAK RIDGE	6868	03	MOREHOUSE	32 37	91 47W	82		07		H
OAKDALE	6836	07	ALLEN	30 49	92 40W	110		07		H
OAKNOLIA 2N	6911	06	EAST FELICIANA	30 45	90 60W	150		07		H
OBERLIN FIRE TWR	6938	07	ALLEN	30 36	92 46W	65	09	09		H
OLD TOWN BAY	6968	07	CALCASIEU	30 17	93 9W	12		07		H
OPELOUSAS	6995	05	ST. LANDRY	30 30	92 6W	56		07		H
PINE GROVE FIRE TWR	7304	06	ST. HELENA	30 43	90 45W	190		13		H
PIONEER 6 W	7312	03	WEST CARROLL	32 45	91 32W	88		08		H
PLAQUEMINE 2 N	7366	08	IBERVILLE	30 19	91 15W	20		07		H
PONCHATOULA 4 SE	7425	06	TANGIPAHOA	30 25	90 23W	18		07		H
PORT ALLEN	7448	05	WEST BATON ROUGE	30 27	91 13W	15		07		H
RAYVILLE	7691	03	RICHLAND	32 30	91 45W	89	07	07		CH
RED RIVER LOCK # 2	7732	05	RAPIDES	31 11	92 17W	75		07		H
RED RIVER LOCK #1	7729	05	CATAHOULA	31 15	91 58W	70		07		H
RED RIVER RSCH STN	7738	01	BOSSIER	32 25	93 38W	155	07	07	07	GCH
ROCKEFELLER WL REFUGE	7932	07	CAMERON	29 44	92 49W	4	08	08		H
RUSTON LA TECH	8067	02	LINCOLN	32 32	92 41W	260	08	08		H
SAILES FIRE TWR	8094	02	BIENVILLE	32 22	93 9W	360		13		H
SHREVEPORT AP R	8440	01	CADDO	32 27	93 49W	254	24	24		HJ
SHREVEPORT DWTN	8436	01	CADDO	32 31	93 45W	180		07		H
SHREVEPORT DWTN AP R	8438	01	CADDO	32 33	93 45W	179	24	24		H
SHREVEPORT STHRN HILLS	8444	01	CADDO	32 24	93 47W	200	07	07		CH
SHREVEPORT WFO	8448	01	CADDO	32 27	93 50W	274	24	24		H
SLIDELL	8539	06	ST. TAMMANY	30 16	89 46W	10	08	08		CH
SLIDELL AP R	8543	06	ST. TAMMANY	30 21	89 49W	27	24	24		H
SPRINGHILL	8683	01	WEBSTER	32 60	93 27W	240		07		H
ST FRANCISVILLE	8136	06	WEST FELICIANA	30 47	91 23W	115		07		H
ST GABRIEL	8139	08	IBERVILLE	30 16	91 6W	30		08		H
ST JOSEPH 3 N	8163	03	TENSAS	31 57	91 14W	78	08	08	07	GCH
ST MARTINVILLE 3 SW	8181	08	ST. MARTIN	30 5	91 52W	30	08	08		H
SULPHUR	8831	07	CALCASIEU	30 14	93 21W	10		24		H

STATION INDEX

STATION	INDEX NO.	DIVISION	COUNTY	LATITUDE	LONGITUDE	ELEVATION (IN FEET)	OBSERVATION TIME AND TABLES: LOCAL STD TIME: TEMP	PRECIP	EVAP	SPECIAL SEE (NOTES)
SUN	8861	06	ST. TAMMANY	30 39	89 55W	75		06		H
TALISHEEK	8906	06	ST. TAMMANY	30 31	89 52W	60		08		H
TALLULAH	8923	03	MADISON	32 24	91 11W	85	08	08		H
TALLULAH VICKSBURG RGN R	8926	03	MADISON	32 21	91 2W	86	24	24		H
TERRYTOWN 3S	8941	09	JEFFERSON	29 55	90 2W	10	07	07		H
THIBODAUX 4 SE	9013	09	LAFOURCHE	29 45	90 46W	15	08	08		CH
TICKFAW 3 ENE	8945	06	TANGIPAHOA	30 36	90 27W	53		24		H
TOLEDO BEND LAKE	9074	04	SABINE	31 12	93 34W	181	08	08	08	H
VILLE PLATTE	9369	05	EVANGELINE	30 42	92 16W	70		07		H
VINTON 5W	9376	07	CALCASIEU	30 12	93 41W	11	08	08		H
VIVIAN	9392	01	CADDO	32 54	93 59W	220		07		H
WEST MONROE	9631	02	OUACHITA	32 28	92 9W	75		07		H
WINNFIELD 3 N R	9803	02	WINN	31 58	92 39W	160	24	24		H
WINNSBORO 2 SE	9804	03	FRANKLIN	32 8	91 43W	74	08	08		G H
WINNSBORO 5 SSE	9806	03	FRANKLIN	32 6	91 42W	80	07	07		GCH
ZWOLLE 2 NW	9980	04	SABINE	31 40	93 40W	209		07		H

REFERENCE NOTES

STATION NAMES: Name of the city, town or locality. Figures and letters following the station names indicate the distance in miles and direction from the post office or town community center.

DIVISIONS: Areas within a state of similar climatological characteristics. Division averages are calculated using data from stations that record temperature and/or precipitation. Station Precipitation totals flagged with an 'F' or 'M' are excluded from the Divisional Average calculations of precipitation. Stations with monthly Temperature averages flagged with an 'F' or 'M' are included in the Divisional Average if there are no more than 9 flagged or missing daily values in the month, else they are excluded from the divisional average for temperature.

NORMALS: The average value of the meteorological element over a time period. Effective 1 January 2012, the averaging period is 1981 to 2010. The normals for National Weather Service localities have been adjusted so as to be representative for the current observation site.

MONTHLY DEGREE DAY TOTALS: One heating (cooling) degree day is accumulated for each whole degree that the daily mean temperature is below (above) 65 degrees Fahrenheit.

PRECIPITATION: Values shown in hundredths of inches are water equivalent totals, i.e., total of liquid and melted frozen precipitation. In the "Monthly Summarized Data" table the total snow and sleet values shown in tenths of inches are unmelted amounts. The max depth on ground values of snow and sleet shown in whole inches are cumulative unmelted amounts. The number of days with .10, .50, 1.00 or more refers to water equivalents.

PRECIPITATION QUALITY CONTROL: The NCDC quality control process may flag precipitation data that are spatially inconsistent, exceed climatological limits, or are inconsistent with prevailing weather patterns.

TEMPERATURE: Original temperature values are given in the "Daily Temperature" table. Summary temperature information (averages, departures, extremes, monthly degree day totals) is based on the values labeled MAX/MIN.

WIND: (As shown in the "Evaporation and Wind" table) the total wind movement in miles over the evaporation pan as determined by an anemometer recorder located 6-8 inches above the pan.

SYMBOLS AND LETTERS USED IN THE STATION INDEX TABLE

C Station is equipped with recording rain gage (R) but values in this bulletin are from a non-recording rain gage unless indicated by an R.
G Observations appear in the "Soil Temperatures" table.
H Observations appear in the "Snowfall and Snow on the Ground" table.
J Station also published as a Local Climatological Data publication.
VAR Observation time varies.
SR Observation time near sunrise.
SS Observation time near sunset.

SYMBOLS AND LETTERS USED IN THE DATA TABLES

(DAILY DATA ARE FOR THE 24 HOURS IMMEDIATELY PRECEDING OBSERVATION TIME.)
BLANK Entries in the "Monthly Summarized Data" table indicate no record.
BLANK Entries in the "Daily Precipitation" and "Snowfall and Snow on the Ground" tables indicate zero.
BLANK Entries in the "Daily Temperature" table indicate a missing record

- No record. Data not recorded or not received in time for publication.
+ Precipitation or temperature extremes occurred on one or more previous dates during the month.
* Rain gage not read. Precipitation is included in the amount following the asterisks. Time distribution may not be known. A * preceding the monthly total indicates precipitation amount is being carried forward to next month's total, and may include amounts from the previous month(s).
a As a subscript, indicates accumulated total.

A Amount of precipitation is the total of observer's entries for the current month. It may include precipitation that occurred during the previous month. Refer to earlier bulletin to determine date of last reading. (Hawaii stations)
B Divisional Departure from normals are computed using 1971-2000 normals.
E Normalized HDD/CDD Calculation. E is appended to the HDD/CDD Calculation when 1-9 individual daily TMAX and/or TMIN values are missing and a Normalized HDD/CDD Calculation is provided. M appears alone if 10 or more daily values are missing.
F Monthly calculation flagged value. F is appended to average and/or total values computed which exclude one or more daily data values that have been flagged by the GHCN-Daily Dataset
M Insufficient or partial data. M is appended to average and/or total values computed with 1-9 daily values missing. M appears alone if 10 or more daily values are missing, (8 or more for wind and evaporation).
N Indicates snow fall or Snowdepth totals are computed with one or more missing days.
R Amounts from recording rain gage.
T Trace. An amount too small to measure.

SEASONAL TABLES: Monthly and seasonal snowfall and heating degree days for the 12 months ending with the June data are published in the July issue of this bulletin. Cooling degree days for the calendar year are published in the "Climatological Data Annual Summary."

Information concerning the history of changes in locations, exposure, etc. of substations is kept on file at the National Climatic Data Center. Historical information of regular National Weather Service Offices may be obtained from the "Local Climatological Data" annual publication. The contents of this publication may be reprinted or otherwise used freely, with proper credit to the National Climatic Data Center. The data are also available digitally.

Effective with the January 2011 Data-Month, COOP Observer Names are no longer included in the Monthly and Annual Climatological Data Publications. This information is not published to ensure the privacy of personal information pursuant to Section 208 of the E-Government Act of 2002 (44 USC 3601).

As of the 2011 Data-Year, Station and Climate Division Maps are no longer being included in the CD Publications. NCDC's Product Development Branch provides updated Station Maps for various data networks via the Historical Observing Metadata Repository: http://www.ncdc.noaa.gov/homr.

The GHCN-Daily Quality Control Flags shown below are displayed as superscripts with the data. For more Information on Global Historical Climatology Network - Daily and flags, see:
http://www.ncdc.noaa.gov/oa/climate/ghcn-daily/
and
Comprehensive Automated Quality Assurance of Daily Surface Observations.
Durre, Imke, Matthew J. Menne, Byron E. Gleason, Tamara G. Houston,
Russell S. Vose, 2010: J. Appl. Meteor. Climatol., 49, 16151633.
doi: 10.1175/2010JAMC2375.1

Blank = Passed All checks	N = failed naught check
D = failed duplicate check	O = failed climatological outlier check
G = failed gap check	R = failed lagged range check
I = failed internal consistency check	S = failed spatial consistency check
K = failed streak/frequent-value check	T = failed temporal consistency check
L = failed check on length of multiday period	W = temperature too warm for snow
M = failed megaconsistency check	X = failed bounds check
	Z = flagged as a result of an official Datzilla investigation

Beginning with the January 2013 CD Publication, monthly mean temperature calculations have changed to the National Data Stewardship Team standard. Monthly maximum and minimum temperatures are not rounded until after the monthly mean temperature is calculated. This is the most accurate outcome, but may be slightly different from the mean derived from rounded monthly maximum and minimum.

Processing Updates and Errata: The 2011 CD Publications were reproduced in May 2013. This update included the addition of late reports and corrections based on additional investigations of reported data issues through NCDCs Datzilla system. In addition, divisional averages for precipitation were recalculated using the method described in DIVISIONS above. Previous editions of the 2011 Publications included all precipitation stations regardless of missing data in the calculation of divisional averages. HDD/CDD values were recalculated to match the legacy method of calculation (truncation of monthly HDD/CDD values instead of rounding).

These and other publications are available from the National Climatic Data Center

Hourly Precipitation Data
This publication contains hourly precipitation amounts obtained from recording rain gages located at National Weather Service, Federal Aviation Administration, and cooperative observer stations. Published data are displayed in inches and tenths or inches and hundredths at local standard time. HPD includes maximum precipitation for nine (9) time periods from 15 minutes to 24 hours, for selected stations.

Climatological Data
Monthly editions contain station daily maximum and minimum temperatures and precipitation. Some Stations provide daily snowfall, snow depth, evaporation, and soil temperature data. Each edition also contains monthly summaries for heating and cooling degree days (65 degree F base). The July issue contains a recap of monthly heating degree days and snow data for the preceding July through June.

The Annual issue contains monthly and annual averages of temperature, precipitation, temperature extremes, freeze data, soil temperatures, evaporation, and a recap of monthly cooling degree days.

Storm Data
Monthly issues contain a chronological listing, by states, of occurrences of storms and unusual weather phenomena. Reports contain information on storm paths, deaths, injuries, and property damage. An "Outstanding storms of the month" section highlights severe weather events with photographs, illustrations, and narratives. The December issue includes annual tornado, lightning, flash flood, and tropical cyclone summaries.

Monthly Climatic Data for the World
This publication contains monthly means for temperature, pressure, precipitation, vapor pressure, and sunshine for approximately 2,000 surface data collection stations worldwide and monthly mean upper air temperatures, dew point depressions, and wind velocities for approximately 500 observing sites.

Local Climatological Data
LCD publications summmarize temperature, relative humidity, precipitation, cloudiness, wind speed and direction observations for several hundred cities in the U.S. and its territories. Each monthly publication also contains 3 hourly weather observations for that month and a hourly summary of precipitation. Annual LCD publications contain a summary of the past calendar year as well as historical averages and extremes.

For Information Call:

(828) 271-4800 Option 2
(828) 271-4010 (TDD)
(828) 271-4876 (Fax)

NOAA\National Climatic Data Center
Attn: User Engagement & Services Branch
151 Patton Avenue
Asheville, NC 28801-5001

Customer Services Number: (828) 271-4800, option 2
TDD : (828) 271-4010
Fax number: (828) 271-4876

NCDC now offers free online access to the ***Climatological Data*** **publication.**
Go to : **www.ncdc.noaa.gov**   and choose Most Popular.
