# Supplementary material for: Why is the lawn buzzing?
Source: Biodivers Data J. 2014 Apr 24;(2):e1101. doi: 10.3897/BDJ.2.e1101 (PMC4040422; doi:10.3897/BDJ.2.e1101)
Supplement: Supplementary material 4 — Climatological Data for Louisiana, September 2013 [file biodiversity_data_journal-2-e1101-s004.pdf]

CLIMATOLOGICAL DATA

LOUISIANA

SEPTEMBER 2013

VOLUME 118 NUMBER 09
ISSN 0145-0409
GHCND Ver: 3.12-upd-2013122706

SEPTEMBER PRECIPITATION BY YEAR

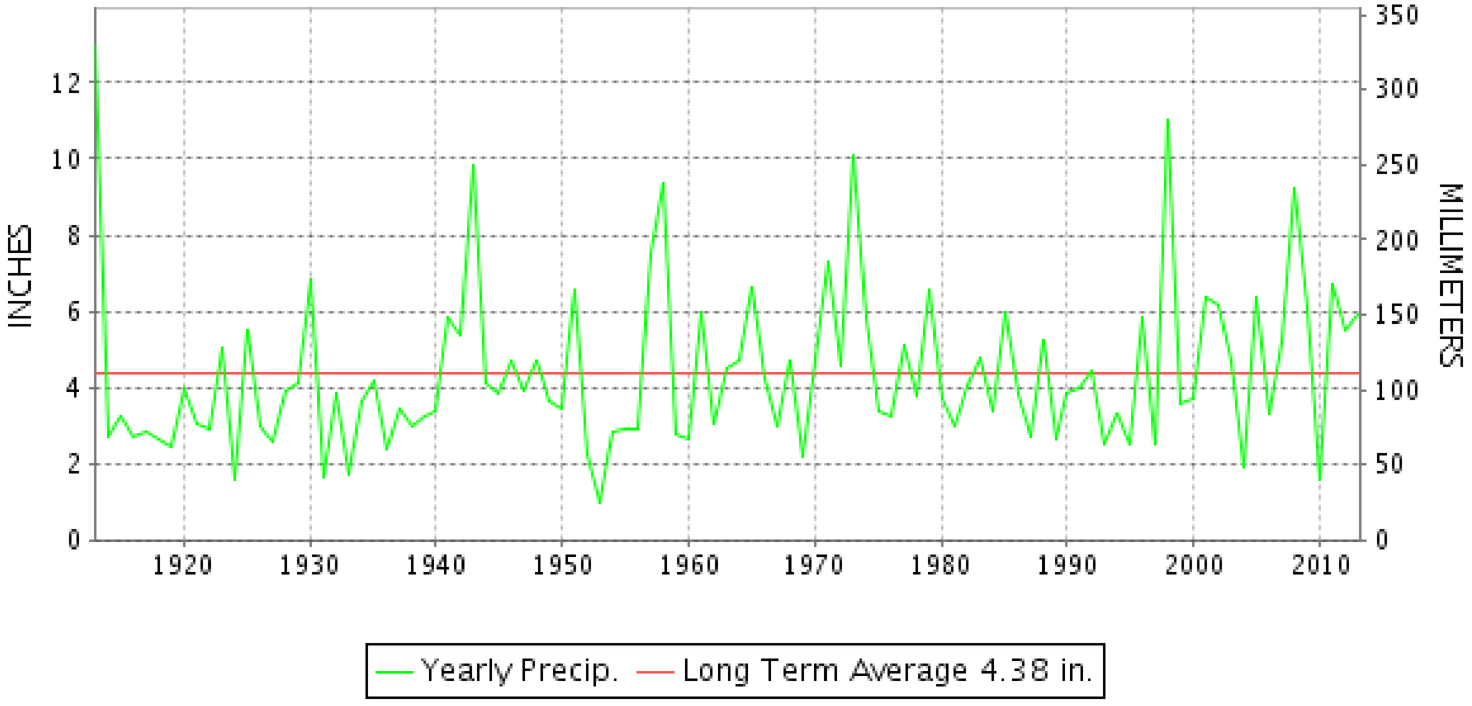

TEMPERATURE AND PRECIPITATION EXTREMES

**LOUISIANA**			
HIGHEST TEMPERATURE	104	SEPTEMBER 03	SHREVEPORT AP
LOWEST TEMPERATURE	48	SEPTEMBER 23	HOMER 1N
GREATEST TOTAL PRECIPITATION	16.01		MOSS BLUFF 2 NNW
LEAST TOTAL PRECIPITATION	1.81		RAYVILLE
GREATEST 1 DAY PRECIPITATION	11.02	SEPTEMBER 21	MOSS BLUFF

"I certify that this is an official publication of the National Oceanic and Atmospheric Administration (NOAA) It is compiled using information from weather observing sites supervised by NOAA/National Weather Service and received at the National Climatic Data Center(NCDC), Asheville, North Carolina 28801."

Director
National Climatic Data Center

noaa

National
Oceanic and
Atmospheric Administration

National
Environmental Satellite, Data
and Information Service

National
Climatic Data Center
Asheville, North Carolina

MONTHLY STATION AND DIVISION SUMMARY

STATION	TEMPERATURE (°F) AVERAGE MAXIMUM	AVERAGE MINIMUM	AVERAGE	DEPARTURE FROM NORMAL	HIGHEST	DATE	LOWEST	DATE	HEATING DEG. DAYS	COOLING DEG. DAYS	NO. OF DAYS MAX >=90	MAX <=32	MIN <=32	MIN <=0	PRECIPITATION (IN) TOTAL	DEPARTURE FROM NORMAL	GREATEST 24 HOURS	DATE	ICE PELLETS, SNOW TOTAL	MAX DEPTH ON GROUND	DATE	NO. OF DAYS .10 OR MORE	.50 OR MORE	1.00 OR MORE
**LOUISIANA**																								
**NORTHWEST 01**																								
BENTON 5E	91.4	67.2	79.3	3.6	100	02	55	22	0	436	20	0	0	0	6.72	3.05	3.94	21	0.0	0		4	3	3
HOSSTON															7.83		6.11	21	0.0			3	2	2
JAMESTOWN															5.14		3.13	21	0.0			4	3	2
KEITHVILLE															7.23		3.40	21	0.0	0		4	3	3
KORAN															5.44		3.60	21	M 0.0	0		3	3	2
LOGANSPORT															M 7.69		4.10	21	0.0	0		4	3	3
MANSFIELD 7 NW	M	M	M		91	27	58	29	0	300E	3	0	0	0	M				M 0.0	0				
MINDEN	92.1	67.4	79.7	4.2	100	02	54	22	0	450	21	0	0	0	7.51	3.94	3.75	21	0.0	0		4	3	2
MOORINGSPORT 1 N	91.9M	67.5M	79.7M	3.6	101	02	54	22	0	449E	22	0	0	0	M 6.84	3.16	5.32	21	0.0	0		5	3	1
RED RIVER RSCH STN	92.9	67.5	80.2	3.8	100	04	54	23+	0	461	25	0	0	0	6.63	3.40	3.10	29	0.0			4	2	2
SHREVEPORT DWTN															A 5.71		3.11	21	0.0	0		2	2	1
SHREVEPORT DWTN AP	92.9	68.3	80.6	4.3	101	03	54	23	0	476	24	0	0	0	4.88	1.52	2.99	20	0.0			4	3	2
SHREVEPORT AP	93.9	69.7	81.8	4.9	104	03	57	23	0	516	24	0	0	0	9.31	6.15	3.46	28	0.0	0		4	4	4
SHREVEPORT STHRN HILLS	93.9	67.3	80.6	4.3	103	02	55	24+	0	476	24	0	0	0	7.31	3.77	3.57	21	0.0	0		4	3	2
SHREVEPORT WFO	92.3	70.8	81.6	4.7	102	01	57	23	0	505	24	0	0	0	8.95	5.59	3.43	28	0.0	0		5	4	3
SPRINGHILL															M				0.0					
VIVIAN															M				M 0.0	0				
**--DIVISIONAL DATA------->**			80.4	4.8B											6.89	3.50B								
**NORTH CENTRAL 02**																								
ARCADIA															5.85		4.20	21	0.0	0		5	3	1
BIENVILLE 3 NE	93.0	67.7	80.4	4.5	101	01	57	23	0	468	22	0	0	0	3.24	-0.69	2.05	20	0.0			4	2	1
CALHOUN RSCH STN	92.6	63.2	77.9	3.2	99	10+	50	24+	0	397	19	0	0	0	4.13	0.82	3.03	21	0.0			4	2	1
COLUMBIA LOCK	91.8M	66.4M	79.1M	3.6	98	11+	56	24+	0	430E	17	0	0	0	3.08	-0.03	2.05	21	0.0	0		5	1	1
FARMERVILLE	91.4	67.7	79.5	4.0	101	02	55	23+	0	442	18	0	0	0	4.65	0.59	3.31	21	0.0	0		5	2	1
HOMER 1N	90.3M	63.7M	77.0M	3.0	98	02	48	23	1E	368E	18	0	0	0	M 5.41	1.63	2.62	21	0.0			4	3	2
JONESBORO 4 ENE															M				0.0					
MONROE REGIONAL AP	93.2	66.5	79.9	3.2	100	03	55	23	0	453	20	0	0	0	2.31	-1.32	1.66	20	0.0	0		4	1	1
MONROE DELTA CC															2.74	-0.76	1.91	21	0.0	0		4	1	1
RUSTON LA TECH	91.2	64.1	77.6	3.4	97	09+	52	26+	0	388	20	0	0	0	4.47	1.19	3.53	21	M 0.0	0		4	1	1
SAILES FIRE TWR															5.62		3.30	21	0.0	0		4	2	2
WEST MONROE															2.79		2.15	21	0.0			4	1	1
WINNFIELD 3 N	M	M	M								0	0	0	0	M				0.0					
**--DIVISIONAL DATA------->**			78.8	3.4B											3.89	0.22B								
**NORTHEAST 03**																								
BASTROP	91.1	65.5	78.3	3.6	99	02	54	23+	0	404	19	0	0	0	4.58	1.26	2.66	21	0.0			6	3	1
LAKE PROVIDENCE	90.4	67.9	79.2	3.1	97	02	57	24	0	432	19	0	0	0	3.10	-0.31	1.91	21	0.0	0		2	2	2

LOUISIANA
201309

MONTHLY STATION AND DIVISION SUMMARY

STATION	TEMPERATURE (°F) AVERAGE MAXIMUM	AVERAGE MINIMUM	AVERAGE	DEPARTURE FROM NORMAL	HIGHEST	DATE	LOWEST	DATE	HEATING DEG. DAYS	COOLING DEG. DAYS	NO. OF DAYS MAX >=90	MAX <=32	MIN <=32	MIN <=0	PRECIPITATION (IN) TOTAL	DEPARTURE FROM NORMAL	GREATEST 24 HOURS	DATE	ICE PELLETS, SNOW TOTAL	MAX DEPTH ON GROUND	DATE	NO. OF DAYS .10 OR MORE	.50 OR MORE	1.00 OR MORE
OAK GROVE															3.31		1.40	21	0.0	0		4	3	1
OAK RIDGE															3.60		1.40	21	0.0	0		4	3	2
PIONEER 6 W															2.93		1.80	21	0.0	0		4	2	1
RAYVILLE	93.3	68.2	80.7	4.7	100	02	57	24	0	479	22	0	0	0	1.81	-1.86	1.02	21	0.0	0		3	2	1
ST JOSEPH 3 N	M	67.6M	MM		98	05+	56	23	0	480E	13	0	0	0	M 6.16	2.67	3.75	21	0.0	0		5	2	2
TALLULAH	90.8M	65.2M	78.0M	1.9	96	10+	56	24+	0	409E	16	0	0	0	M 4.24	0.65	2.06	21	0.0	0		5	4	1
TALLULAH VICKSBURG RGN	91.5	64.9	78.2	3.1	97	10+	49	23	0	405	18	0	0	0	5.65	2.76	2.03	24	0.0	0		6	5	2
WINNSBORO 2 SE	95.2M	67.4M	81.3M	4.9	102	06	57	24	0	495E	24	0	0	0	M 2.34	-1.03	2.34	25	M 0.0	0		1	1	1
WINNSBORO 5 SSE	94.4M	66.0M	80.2M	3.7	100	06	54	23	0	467E	20	0	0	0	2.66	-0.79	2.09	21	0.0	0		3	1	1
**--DIVISIONAL DATA------->**			79.4	3.5B											3.46	0.41B								
**WEST CENTRAL 04**																								
GORUM FIRE TWR															M				0.0					
HODGES GARDENS	92.3	68.9	80.6	3.7	98	06+	56	23	0	474	23	0	0	0	3.78	-0.40	1.90	21	0.0			5	3	1
LEESVILLE	92.2	65.7	79.0	3.2	101	03	55	26+	0	426	23	0	0	0	7.02	2.67	3.06	21	0.0			6	3	2
LEESVILLE 6 SSW															7.51		3.26	23	0.0			6	4	3
MANY 9 WSW															3.71		2.00	21	0.0	0		5	3	1
NATCHITOCHES #2	89.8	68.0	78.9	1.5	97	04	56	23	0	425	19	0	0	0	3.71	-0.83	2.47	21	0.0	0		4	2	1
TOLEDO BEND LAKE	92.6	69.0	80.8	3.4	100	17	59	23+	0	480	24	0	0	0	4.15	0.16	1.92	21	0.0	0		3	2	2
ZWOLLE 2 NW															5.82		2.90	21	0.0	0		3	3	2
**--DIVISIONAL DATA------->**			79.8	3.9B											5.10	1.39B								
**CENTRAL 05**																								
ALEXANDRIA	91.7	69.8	80.7	2.6	98	14+	61	23	0	480	21	0	0	0	6.14	2.21	3.20	21	M 0.0	0		4	3	2
ALEXANDRIA 5 SSE	89.7	65.9F	77.8F	1.7	97	13+	51	26	0	379	19	0	0	0	5.76	1.53	3.68	20	M 0.0	0		4	4	1
BEAVER FIRE TWR															6.92		4.05	21	0.0			4	3	2
BOYCE 3 WNW	88.1	70.5	79.3	2.0	94	13+	60	23+	0	439	18	0	0	0	3.93	-0.50	2.58	20	M 0.0	0		5	2	1
BUNKIE	90.6	68.5	79.5	2.7	96	05+	61	26+	0	444	20	0	0	0	5.41	0.65	4.00	21	0.0			5	2	1
CLAYTON															M 2.60		2.40	21	M 0.0	0		2	1	1
EUNICE	91.5	69.6	80.5	1.5	97	14+	61	26	0	472	23	0	0	0	11.32	6.53	6.16	21	0.0			4	4	3
GRAND COTEAU	91.3	69.5	80.4	2.9	96	04+	60	26	0	471	23	0	0	0	9.93	5.16	2.30	19	M 0.0	0		10	6	5
JENA 4 WSW	92.7	66.5	79.6	3.8	101	04	57	23	0	444	21	0	0	0	2.25	-1.42	1.70	21	0.0			3	1	1
JONESVILLE LOCKS	91.7	69.2	80.4	3.1	97	16+	61	26+	0	470	19	0	0	0	4.39	0.64	2.70	21	0.0	0		6	3	1
LSU DEAN LEE RSCH STN	M	M	M		96	16+	60	26	0	401E	10	0	0	0	M				0.0					
MARKSVILLE															4.73		3.72	21	0.0			2	2	1
NEW ROADS 5 NE	92.1	70.9	81.5	2.9	97	10+	63	26	0	502	23	0	0	0	5.37	1.12	4.35	21	0.0			3	2	1
OPELOUSAS															4.52		2.10	21	0.0			5	4	1
PORT ALLEN															5.85		4.13	21	0.0			6	1	1
RED RIVER LOCK #1															5.50		3.65	21	0.0	0		4	2	2
RED RIVER LOCK # 2															6.37		5.60	21	0.0			2	2	1

MONTHLY STATION AND DIVISION SUMMARY

STATION	TEMPERATURE (°F) AVERAGE MAXIMUM	AVERAGE MINIMUM	AVERAGE	DEPARTURE FROM NORMAL	HIGHEST	DATE	LOWEST	DATE	HEATING DEG. DAYS	COOLING DEG. DAYS	NO. OF DAYS MAX >=90	MAX <=32	MIN <=32	MIN <=0	PRECIPITATION (IN) TOTAL	DEPARTURE FROM NORMAL	GREATEST 24 HOURS	DATE	ICE PELLETS, SNOW TOTAL	MAX DEPTH ON GROUND	DATE	NO. OF DAYS .10 OR MORE	.50 OR MORE	1.00 OR MORE
VILLE PLATTE															M				0.0					
**--DIVISIONAL DATA------->**			80.0	3.0B											5.89	1.56B								
**EAST CENTRAL 06**																								
ABITA RVR COVINGTON															7.92		2.90	22	0.0			11	6	1
ABITA SPRINGS 1 SW															5.47		3.43	22	M 0.0			4	2	2
ABITA SPRING FIRE TWR															5.66		2.82	21	0.0			7	3	2
ANGIE															M				0.0					
BAKER															MA 6.52		0.57	24	0.0			2	1	0
BATON ROUGE CONCORD															8.08		4.35	21	M 0.0			6	4	2
BATON ROUGE METRO AP	90.7	70.6	80.7	2.1	95	03	63	26	0	479	22	0	0	0	6.90	2.36	4.67	21	0.0	0		6	2	1
BATON ROUGE SHERWOOD															7.90		4.28	21	0.0			7	3	2
BOGALUSA	M	M	M								0	0	0	0	M				0.0					
CLINTON FORESTRY HQ															5.17		4.77	21	0.0			3	1	1
CLINTON 5 SE	89.1	67.0	78.1	2.4	94	04	60	26	0	399	17	0	0	0	7.88	4.24	6.00	21	0.0			5	3	2
COVINGTON 3 NE															M				0.0					
DENHAM SPRINGS															4.48		3.15	21	M 0.0	0		6	1	1
HAMMOND 5 E	89.2	68.1	78.6	1.3	95	04	59	26	0	415	16	0	0	0	4.90	0.58	2.02	22	0.0			7	3	2
KILLIAN															M 2.08		0.90	25	0.0			4	2	0
LIVERPOOL 6W															M				0.0					
LIVINGSTON															7.63		3.90	21	0.0			6	3	2
LSU BEN-HUR FARM	90.1	69.5	79.8	2.0	95	14+	62	26	0	450	19	0	0	0	7.10	2.71	3.83	21	0.0			5	4	2
MOUNT HERMON 2W															3.60		2.02	20	0.0			4	2	2
NORWOOD															5.44		3.77	21	0.0			5	2	2
OAKNOLIA 2N															7.55		5.83	21	0.0			5	3	1
PINE GROVE FIRE TWR															5.63		3.00	07	0.0			4	2	2
PONCHATOULA 4 SE															M 3.00		1.60	22	0.0			3	3	1
ST FRANCISVILLE															6.40		5.17	21	0.0			4	2	1
SLIDELL	87.6	71.0	79.3	1.3	92	04+	63	26	0	436	10	0	0	0	5.65	0.66	1.51	22	0.0			7	5	3
SLIDELL AP	89.1	70.3	79.7	2.4	94	12	62	29	0	450	16	0	0	0	5.62	1.55	1.68	21	0.0			9	4	3
SUN															4.78		2.53	22	0.0			6	3	1
TALISHEEK															3.81		1.48	21	0.0			5	2	2
TICKFAW 3 ENE															M				0.0					
**--DIVISIONAL DATA------->**			79.4	2.3B											6.07	1.31B								
**SOUTHWEST 07**																								
ABBEVILLE															4.26		2.32	21	0.0			8	2	1
BELL CITY 13 SW															10.31		3.55	21	0.0			7	6	3
CROWLEY 2 NE	91.5	69.8	80.6	2.4	96	04	61	26	0	477	23	0	0	0	4.22	-0.62	2.79	22	0.0			5	2	1
DE RIDDER	91.5	69.3	80.4	3.1	98	03	60	26	0	467	22	0	0	0	5.35	0.04	2.06	21	0.0			7	4	1

LOUISIANA
201309

MONTHLY STATION AND DIVISION SUMMARY

STATION	TEMPERATURE (°F)														PRECIPITATION (IN)									
											NO. OF DAYS								ICE PELLETS, SNOW			NO. OF DAYS		
											MAX		MIN											
	AVERAGE MAXIMUM	AVERAGE MINIMUM	AVERAGE	DEPARTURE FROM NORMAL	HIGHEST	DATE	LOWEST	DATE	HEATING DEG. DAYS	COOLING DEG. DAYS	>=90	<=32	<=32	<=0	TOTAL	DEPARTURE FROM NORMAL	GREATEST 24 HOURS	DATE	TOTAL	MAX DEPTH ON GROUND	DATE	.10 OR MORE	.50 OR MORE	1.00 OR MORE
DRY CREEK 8NW															M				0.0					
HACKBERRY 8 SSW	88.6	74.7	81.7	1.8	93	14	66	23	0	509	18	0	0	0	9.40	4.23	4.94	21	0.0			6	4	3
JENNINGS	91.0	71.1	81.0	2.7	97	04	63	27+	0	486	24	0	0	0	5.25	-0.17	2.12	21	0.0			8	3	2
KAPLAN															8.00		3.05	21	0.0			10	5	3
LAKE ARTHUR 10 SW	90.7	72.3	81.5	2.2	96	05+	66	26+	0	502	22	0	0	0	5.74	0.18	2.54	21	0.0			6	4	3
LAKE CHARLES 7 NW															12.21		10.10	21	0.0			5	3	2
LAKE CHARLES 2 N															11.40		8.48	21	0.0			7	4	1
LAKE CHARLES PORT															9.61		6.20	21	0.0			7	3	2
LAKE CHARLES AP	90.7	72.4	81.6	3.0	97	13	66	25	0	504	22	0	0	0	9.69	4.43	3.86	20	M 0.0	0		7	5	4
LELAND BOWMAN LOCK	92.2	73.4	82.8	4.4	98	04	67	24+	0	540	25	0	0	0	6.63	0.98	2.00	21	M 0.0	0		8	5	3
MOSS BLUFF															13.30		11.02	21	0.0			5	3	1
MOSS BLUFF 2 NNW	91.4	69.8	80.6	3.0	98	03	60	26	0	474	23	0	0	0	A 16.01	10.26	8.97	21	0.0			4	2	2
OAKDALE															8.30		6.00	21	0.0			3	3	3
OBERLIN FIRE TWR	90.4M	68.7M	79.6M	2.2	97	03	60	26	0	447E	20	0	0	0	8.46	3.11	6.48	21	0.0			6	2	2
OLD TOWN BAY															12.28		9.10	21	0.0			6	4	1
ROCKEFELLER WL REFUGE	88.6	71.9	80.3	2.2	93	14	64	23	0	463	17	0	0	0	4.17	-1.96	1.82	21	0.0			6	3	1
SULPHUR															11.51		7.04	20	0.0			7	5	3
VINTON 5W	92.0M	70.0M	81.0M		99	04	63	26+	0	488E	23	0	0	0	M 10.16		6.00	21	0.0			6	4	2
**--DIVISIONAL DATA------->**			81.0	3.3B											8.81	3.34B								
**SOUTH CENTRAL 08**																								
BAYOU SORREL LOCK															9.82		6.46	21	0.0			7	3	2
CARENCRO															5.28		2.50	21	0.0			5	3	2
CARVILLE 2 SW	88.3	72.0	80.2	1.8	94	04	66	26+	0	464	13	0	0	0	3.37	-0.87	2.25	21	0.0			3	2	1
DONALDSONVILLE 4 SW	88.7	69.3	79.0	1.2	93	04	62	26	0	428	18	0	0	0	4.40	-0.78	3.10	21	M 0.0			5	1	1
FRANKLIN 3 NW	88.4	71.4	79.9	2.2	92	03	65	26+	0	454	15	0	0	0	6.20	0.45	2.82	21	0.0			10	3	1
JEANERETTE 5 NW	88.8	69.7	79.3	1.2	94	04	63	27+	0	434	19	0	0	0	4.84	-1.61	2.00	21	0.0			11	2	1
LAFAYETTE	91.0	71.3	81.2	3.7	95	14	64	28+	0	492	23	0	0	0	4.46	-0.76	1.22	22	0.0			9	4	1
LAFAYETTE FCWOS	91.2	71.9	81.5	2.3	96	13	64	26	0	503	22	0	0	0	3.66	-1.06	2.23	21	0.0	0		5	1	1
MORGAN CITY	89.2	72.0	80.6	1.6	94	17+	65	30	0	474	21	0	0	0	5.41	-0.34	1.35	09	0.0			7	4	2
NAPOLEONVILLE															3.04		2.25	21	0.0			4	1	1
NEW IBERIA AP ACADIANA RGNL	93.0	73.7	83.4	4.7	98	03	65	26	0	558	24	0	0	0	4.02	-0.61	1.36	21	0.0			7	3	1
PLAQUEMINE 2 N															5.48		4.00	21	0.0			5	3	1
ST GABRIEL															8.71		3.75	21	0.0			8	5	2
ST MARTINVILLE 3 SW	89.0	69.5	79.3	1.8	94	04+	61	26	0	434	18	0	0	0	3.92	-1.73	1.60	21	0.0			5	3	2
**--DIVISIONAL DATA------->**			80.5	2.5B											5.19	-0.45B								
**SOUTHEAST 09**																								
BOOTHVILLE ASOS	87.0	77.6	82.3	2.1	90	07	73	30+	0	526	1	0	0	0	4.88	-0.80	2.27	21	0.0			3	3	3
CONVENT 2S															4.61		1.65	21	0.0			7	3	3

LOUISIANA
201309

MONTHLY STATION AND DIVISION SUMMARY

STATION	TEMPERATURE (°F)														PRECIPITATION (IN)									
											NO. OF DAYS								ICE PELLETS, SNOW			NO. OF DAYS		
											MAX		MIN											
	AVERAGE MAXIMUM	AVERAGE MINIMUM	AVERAGE	DEPARTURE FROM NORMAL	HIGHEST	DATE	LOWEST	DATE	HEATING DEG. DAYS	COOLING DEG. DAYS	>=90	<=32	<=32	<=0	TOTAL	DEPARTURE FROM NORMAL	GREATEST 24 HOURS	DATE	TOTAL	MAX DEPTH ON GROUND	DATE	.10 OR MORE	.50 OR MORE	1.00 OR MORE
DUTCHTOWN #2															9.05		4.94	21	0.0			6	4	2
GALLIANO	87.5M	73.9M	80.7M	1.7	92	03+	67	28+	0	479E	4	0	0	0	M 4.86	-1.34	3.01	22	0.0			6	2	1
GONZALES															8.66		3.37	21	0.0			6	4	3
GRAND ISLE															M 2.44		0.80	23	0.0			6	2	0
HOUMA	M	M	M								0	0	0	0	M				0.0					
LUTCHER															4.70		1.59	20	0.0			9	3	2
MARRERO 9 SSW	M	M	M		93	16+	67	30+	0	486E	12	0	0	0	M				0.0					
NEW ORLEANS AP	89.5	73.9	81.7	2.0	94	13	67	29	0	511	19	0	0	0	8.68	3.71	2.53	21	0.0	0		9	6	4
NEW ORLEANS AUDUBON	90.0M	73.4M	81.7M	1.4	96	02	64	22	0	507E	18	0	0	0	5.85	0.05	3.03	21	0.0			7	3	2
NEW ORLEANS ALGIERS															M				0.0					
NEW ORLEANS LKFRNT AP	89.6	78.1	83.8	3.2	94	13+	73	29+	0	571	18	0	0	0	6.66	2.54	2.99	21	0.0			7	3	3
TERRYTOWN 3S	90.1	74.0	82.1	2.0	95	03+	68	29	0	518	21	0	0	0	8.54	0.92	3.64	19	M 0.0			7	4	3
THIBODAUX 4 SE	87.4	69.1	78.3	0.1	93	04	63	29+	0	403	7	0	0	0	4.24	-1.87	1.29	21	0.0			5	4	2
**--DIVISIONAL DATA------->**			81.5	2.7B											6.59	0.44B								

DAILY PRECIPITATION (INCHES)

STATION	TOTAL	01	02	03	04	05	06	07	08	09	10	11	12	13	14	15	16	17	18	19	20	21	22	23	24	25	26	27	28	29	30	31
**LOUISIANA**																																
**NORTHWEST 01**																																
BENTON 5E	6.72			0.03																	1.06	3.94								1.38	0.31	
HOSSTON	7.83			0.02																		6.11								1.54	0.16	
JAMESTOWN	5.14																				0.54	3.13								1.37	0.10	
KEITHVILLE	7.23												0.02		0.01						0.40	3.40								2.00	1.40	
KORAN	5.44				0.06								0.06							0.50		3.60								1.15	0.07	
LOGANSPORT	M 7.69									T					0.39						0.05	4.10						-		1.23	1.92	
MANSFIELD 7 NW	M	-	-	-	-	-	-	-	-	-	-	-	-	-	-	-	-	-	-	-	-	-	-	-	-	-	-			1.66	-	
MINDEN	7.51				0.04																0.48	3.75								2.26	0.98	
MOORINGSPORT 1 N	M 6.84		0.13	0.50																	0.73	5.32								-	0.16	
RED RIVER RSCH STN	6.63												0.25								0.15	3.05								3.10	0.08	
SHREVEPORT DWTN	A 5.71			T									T		T						0.55	3.11								*	2.05a	
SHREVEPORT DWTN AP	4.88		T									T		T				0.01		0.22	2.99								1.05	0.58	0.03	
SHREVEPORT AP	9.31													0.06						1.41	3.18	0.01							3.46	1.18	0.01	
SHREVEPORT STHRN HILLS	7.31												T								0.43	3.57								2.65	0.66	
SHREVEPORT WFO	8.95													0.14						0.93	3.27	0.02							3.43	1.13	0.03	
SPRINGHILL	M	-	-	-	-	-	-	-	-	-	-	-	-	-	-	-	-	-	-	-	-	-	-	-	-	-	-	-	-	-	-	
VIVIAN	M	-	-	-	-	-	-	-	-	-	-	-	-	-	-	-	-	-	-	-	-	-	-	-	-	-	-	-	-	2.50		
**NORTH CENTRAL 02**																																
ARCADIA	5.85																				0.11	4.20				0.62				0.77	0.15	
BIENVILLE 3 NE	3.24													0.20						0.10	2.05	0.04								0.80	0.05	
CALHOUN RSCH STN	4.13																				T	3.03			0.55	0.07				0.38	0.10	
COLUMBIA LOCK	3.08				0.38								0.05		0.05							2.05			0.27					0.13	0.15	
FARMERVILLE	4.65			0.02															0.02		0.29	3.31			0.64					0.11	0.26	
HOMER 1N	M 5.41																		0.14		0.09	2.62			0.03		-			1.89	0.64	
JONESBORO 4 ENE	M	-	-	-	-	-	-	-	-	-	-	-	-	-	-	-	-	-	-	-	-	-	-	-	-	-	-	-	-	-	-	
MONROE REGIONAL AP	2.31		0.04								0.06	T		0.22				T			1.66	0.07			0.12					0.14	T	
MONROE DELTA CC	2.74			0.04								T			0.11				T		T	1.91			0.28	0.01					0.39	
RUSTON LA TECH	4.47																		0.26			3.53			0.04					0.42	0.22	
SAILES FIRE TWR	5.62											0.18									0.34	3.30			0.05					1.72	0.03	
WEST MONROE	2.79			0.16															0.02			2.15			0.27						0.19	
WINNFIELD 3 N	M	-	-	-	-	-	-	-	-	-	-	-	-	-	-	-	-	-	-	-	-	-	-	-	-	-	-	-	-	-	-	
**NORTHEAST 03**																																
BASTROP	4.58											0.29			T				0.21		0.15	2.66			0.74						0.53	
LAKE PROVIDENCE	3.10																					1.91			T	1.10					0.09	
OAK GROVE	3.31																				0.08	1.40			0.62	0.93					0.28	
OAK RIDGE	3.60			0.75																		1.40			1.25						0.20	
PIONEER 6 W	2.93																				0.18	1.80			0.73					T	0.22	
RAYVILLE	1.81			0.01																		1.02	0.53		0.05	0.07					0.13	
ST JOSEPH 3 N	M 6.16											-			0.42						0.14	3.75				1.65					0.20	
TALLULAH	M 4.24			0.58				-				0.50									0.20	2.06			0.05	0.85						

LOUISIANA
201309

DAILY PRECIPITATION (INCHES)

STATION	TOTAL	01	02	03	04	05	06	07	08	09	10	11	12	13	14	15	16	17	18	19	20	21	22	23	24	25	26	27	28	29	30	31
TALLULAH VICKSBURG RGN	5.65		0.19								0.59									0.60	1.47	0.77			2.03					T	T	
WINNSBORO 2 SE	M 2.34																					-			-	2.34						
WINNSBORO 5 SSE	2.66																				T	2.09			0.22	0.35						
**WEST CENTRAL 04**																																
GORUM FIRE TWR	M	-	-	-	-	-	-	-	-	-	-	-	-	-	-	-	-	-	-	-	-	-	-	-	-	-	-	-	-	-	-	
HODGES GARDENS	3.78			0.02	0.05	0.01			0.11				0.19								0.63	1.90	0.03								0.84	
LEESVILLE	7.02				0.01	0.01		0.29	0.01												0.36	3.06	0.02		0.43	1.91					0.92	
LEESVILLE 6 SSW	7.51									0.46											0.65			3.26	0.12	1.69					1.33	
MANY 9 WSW	3.71				0.21	0.06	0.01		0.01										0.20		0.53	2.00	0.02								0.67	
NATCHITOCHES #2	3.71			T	0.06				T			T			T			0.16			0.56	2.47	T			T					0.46	
TOLEDO BEND LAKE	4.15				0.02				0.05												0.41	1.92				0.02				0.01	1.72	
ZWOLLE 2 NW	5.82																			0.77		2.90								T	2.15	
**CENTRAL 05**																																
ALEXANDRIA	6.14				0.84										0.01						0.07	3.20				1.59					0.43	
ALEXANDRIA 5 SSE	5.76			0.01	0.05																3.68	0.53			0.94					0.54	0.01	
BEAVER FIRE TWR	6.92								2.02							0.05					0.14	4.05	0.07		0.07					0.02	0.50	
BOYCE 3 WNW	3.93			0.25	0.10															0.06	2.58	0.19								0.75		
BUNKIE	5.41				0.17																0.34	4.00	0.10			T					0.80	
CLAYTON	M 2.60											-			-							2.40				-					0.20	
EUNICE	11.32				2.35				0.01												2.11	6.16	0.59		0.02						0.08	
GRAND COTEAU	9.93			0.20		0.30	0.87				1.00									2.30	1.01	2.10	0.10		0.25						1.80	
JENA 4 WSW	2.25				0.05																	1.70			T					0.20	0.30	
JONESVILLE LOCKS	4.39														0.25						0.55	2.70	0.17			0.50					0.22	
LSU DEAN LEE RSCH STN	M	-		-		0.11	-	-	-					-	-	-					-	-	-	4.89	0.04	0.34			-	-	0.71	
MARKSVILLE	4.73				0.05															0.09		3.72	0.05								0.82	
NEW ROADS 5 NE	5.37				0.69				0.02					0.03								4.35			0.25					0.01	0.02	
OPELOUSAS	4.52											0.06			0.55						0.67	2.10		0.79	0.05						0.30	
PORT ALLEN	5.85				0.06	0.07	0.02	0.13	0.07	0.04			T		0.44						0.16	4.13	0.39		T	0.34					T	
RED RIVER LOCK #1	5.50											0.30										3.65	0.05			1.10					0.40	
RED RIVER LOCK # 2	6.37				0.01	0.07																5.60	0.04								0.65	
VILLE PLATTE	M				0.97									-	-	-	-	-	-	-	-	-	-	-	-	-	-	-	-	-	-	
**EAST CENTRAL 06**																																
ABITA RVR COVINGTON	7.92				0.58	0.96	0.20	0.75							0.30		0.24			0.10	0.10	0.85	2.90			0.94					T	
ABITA SPRINGS 1 SW	5.47				0.48	0.09		T		0.01					0.01		0.04				0.01	1.04	3.43			0.35					0.01	
ABITA SPRING FIRE TWR	5.66				0.24	0.05		0.95							0.11					0.01		2.82	1.00		0.20						0.28	
ANGIE	M	-	-	-	-	-	-	-	-	-	-	-	-	-	-	-	-	-	-	-	-	-	-	-	-	-	-	-	-	-	-	
BAKER	MA 6.52	*	0.00_a		0.49	0.01	0.09	*	*	0.47_a					*	*	0.06_a					*	*	4.83_a	0.57				-	-	-	
BATON ROUGE CONCORD	8.08				0.93		0.75	0.09	T						1.35						0.18	4.35	0.37		0.02	0.04						
BATON ROUGE METRO AP	6.90			0.49	0.87	0.36	T	0.11	0.03					0.02						T	0.04	4.67			0.23					T	0.08	
BATON ROUGE SHERWOOD	7.90				0.44	0.84	0.18	T	0.01			0.02	0.33		1.53						T	4.28	0.25			0.02						
BOGALUSA	M	-	-	-	-	-	-	-	-	-	-	-	-	-	-	-	-	-	-	-	-	-	-	-	-	-	-	-	-	-	-	
CLINTON FORESTRY HQ	5.17														0.02							4.77	0.20			0.18						

LOUISIANA
201309

DAILY PRECIPITATION (INCHES)

STATION	TOTAL	01	02	03	04	05	06	07	08	09	10	11	12	13	14	15	16	17	18	19	20	21	22	23	24	25	26	27	28	29	30	31
CLINTON 5 SE	7.88				0.01	0.17											1.00					6.00	0.50			0.20						
COVINGTON 3 NE	M	-	-	-	-	-	-	-	-	-	-	-	-	-	-	-	-	-	-	-	-	-	-	-	-	-	-	-	-	-	-	
DENHAM SPRINGS	4.48					0.24	0.31	0.02	0.03	0.01		0.02		0.02	0.25		0.01					3.15	0.16		0.04	0.17					0.05	
HAMMOND 5 E	4.90				0.22	0.11									0.77							1.22	2.02	0.12		0.44						
KILLIAN	M 2.08														0.02					0.10	0.03	-	0.32	0.01	0.66	0.90		0.03			0.01	
LIVERPOOL 6W	M	-	-	-	-	-	-	-	-	-	-	-	-	-	-	-	-	-	-	-	-	-	-	-	-	-	-	-	-	-	-	
LIVINGSTON	7.63							2.09		0.39					0.26							3.90	0.85			0.14						
LSU BEN-HUR FARM	7.10				0.05		0.03	0.14							1.89						0.59	3.83	0.53		0.03	0.01						
MOUNT HERMON 2W	3.60						0.14														2.02	1.23				0.21						
NORWOOD	5.44				0.10										0.08							3.77	1.16			0.22					0.11	
OAKNOLIA 2N	7.55				0.72	0.04									0.15							5.83	0.51			0.30						
PINE GROVE FIRE TWR	5.63					0.12		3.00													0.25	2.20			0.06							
PONCHATOULA 4 SE	M 3.00				T			-			-	-		-	T	T						0.70	1.60		T	0.70						
ST FRANCISVILLE	6.40				0.05	0.38														0.02		5.17	0.56			0.17					0.05	
SLIDELL	5.65				0.17	0.11	1.16	1.16	0.01											0.06	0.84	0.58	1.51			0.05						
SLIDELL AP	5.62			0.09	1.13	0.10	1.05								T	T		0.36	0.09	0.26	0.01	1.68	0.14		0.11						0.60	
SUN	4.78				0.02	0.04	0.37	0.05		0.54					0.03						0.74	0.13	2.53			0.33						
TALISHEEK	3.81				1.37	0.01	0.01	0.18							0.01				0.04		0.04	1.48	0.28	T	0.02	0.37						
TICKFAW 3 ENE	M	-	-	-	-	-	-	-	-	-	-	-	-	-	-	-	-	-	-	-	-	-	-	-	-	-	-	-	-	-	-	
**SOUTHWEST 07**																																
ABBEVILLE	4.26						0.16	0.13	0.02	0.68		0.12				T				T	0.40	2.32	0.20	T	0.03						0.20	
BELL CITY 13 SW	10.31	1.20				0.40		0.63				0.04			0.75					0.01	2.97	3.55			0.03	0.01	0.01				0.71	
CROWLEY 2 NE	4.22				0.31		0.10					0.10									0.08		2.79		0.02						0.82	
DE RIDDER	5.35				0.55	0.44	T		0.23								T			T	0.87	2.06	0.03		0.33	0.06					0.78	
DRY CREEK 8NW	M	-	-	-	0.28	0.23	-	-	-	-	-	-	-	-	-	-	-	-	-	-	-	5.28	-	-	-	0.05	-	-	-	-	0.03	
HACKBERRY 8 SSW	9.40				0.02	0.04		0.37	0.21		0.01										1.80	4.94	0.90	0.01	0.08						1.02	
JENNINGS	5.25				1.21		0.72	0.01		T						0.42					0.16	2.12	0.19	0.01						0.20	0.21	
KAPLAN	8.00				0.74	1.01	1.10			0.17		0.12			0.95					0.02	0.48	3.05	0.24								0.12	
LAKE ARTHUR 10 SW	5.74				0.15	0.03	1.06	0.01		0.02		0.03				1.05		0.01	0.02		0.54	2.54	0.14		0.05	0.01					0.08	
LAKE CHARLES 7 NW	12.21					0.02		0.14				0.20	0.02		0.04						1.00	10.10	0.04								0.65	
LAKE CHARLES 2 N	11.40				0.54		0.18					0.48									0.83	8.48	0.15								0.74	
LAKE CHARLES PORT	9.61				0.45	0.05	0.63	0.04								0.02					1.30	6.20	0.28					0.20			0.44	
LAKE CHARLES AP	9.69		0.15	0.52		0.04	0.01	0.04	T		0.01				T				T	1.87	3.86	1.62		T						0.32	1.25	
LELAND BOWMAN LOCK	6.63			0.01					1.38	0.10		0.29				0.98		0.05			1.02	2.00	0.58		0.05						0.17	
MOSS BLUFF	13.30				T							0.37	0.07			0.08			T		0.86	11.02	0.15			T					0.75	
MOSS BLUFF 2 NNW	A 16.01				0.30									0.08				0.03		*	5.08a	8.97								0.49	1.06	
OAKDALE	8.30						1.20		1.10													6.00	T		T							
OBERLIN FIRE TWR	8.46								1.23			0.10			0.12						0.33	6.48	0.05								0.15	
OLD TOWN BAY	12.28					0.03	0.32						0.02							0.97	0.97	9.10	0.24		0.03						0.60	
ROCKEFELLER WL REFUGE	4.17				0.08	0.03		0.05	0.28				0.03				0.63			0.13	0.19	1.82	0.82	0.02	0.07	0.01	0.01				T	
SULPHUR	11.51										0.09	0.10			0.50			0.02		1.26	7.04	0.30								0.90	1.30	
VINTON 5W	M 10.16						0.32		0.50			-				0.12					2.65	6.00	0.03								0.54	

LOUISIANA
201309

DAILY PRECIPITATION (INCHES)

STATION	TOTAL	DAY OF MONTH																														
		01	02	03	04	05	06	07	08	09	10	11	12	13	14	15	16	17	18	19	20	21	22	23	24	25	26	27	28	29	30	31
**SOUTH CENTRAL 08**																																
BAYOU SORREL LOCK	9.82					0.09		0.02	0.40	1.30					0.42						0.20	6.46	0.70	0.20	0.01	0.02						
CARENCRO	5.28																				1.46	2.50	0.35		0.10						0.87	
CARVILLE 2 SW	3.37				0.80			0.02	0.01					0.01					0.02	0.07	0.03	2.25	0.02	0.01	0.03						0.10	
DONALDSONVILLE 4 SW	4.40							0.11	0.05			0.09								0.08	0.19	3.10	0.45		0.26	0.07						
FRANKLIN 3 NW	6.20		0.01	0.10	0.93	0.31	0.25	0.72	0.16						0.12			0.03		0.05	T	2.82	0.07	0.20	0.43							
JEANERETTE 5 NW	4.84			0.18		0.12	0.24	0.10	0.07	0.74		0.09				0.33				0.10	0.21	2.00	0.31		0.30	0.05						
LAFAYETTE	4.46							0.06		0.21		0.10			0.27	0.04				0.90	0.91	0.55	1.22		0.10						0.10	
LAFAYETTE FCWOS	3.66	T	T	0.07		T		0.08			0.27			0.01					T	0.10	0.48	2.23		0.29	0.03					0.09	0.01	
MORGAN CITY	5.41				0.46			0.05	1.33	1.35			0.02			0.64				0.16		0.48	0.88	0.04								
NAPOLEONVILLE	3.04				0.03				0.03	0.21		0.01			T							2.25	0.25	0.01	0.25							
NEW IBERIA AP ACADIANA RGNL	4.02		0.01	T	0.05	0.06	0.04	0.42	0.60		T			T	T			T	T	0.97	0.13	1.36	T	0.16	0.18					0.03	0.01	
PLAQUEMINE 2 N	5.48				0.01	0.01	0.10								0.68						0.02	4.00	0.50		0.01	0.15						
ST GABRIEL	8.71				0.08	0.94	0.17	0.44		T					1.44				T	T	0.80	3.75	0.34		0.01	0.74						
ST MARTINVILLE 3 SW	3.92					0.03	0.01		0.01	0.60		0.08			0.11	0.04					1.00	1.60			0.38						0.06	
**SOUTHEAST 09**																																
BOOTHVILLE ASOS	4.88	T	T		T	T						T					T		0.06	0.08	0.01	2.27	1.18	0.09	0.01						1.18	
CONVENT 2S	4.61									0.25					0.11					0.10	1.00	1.65	1.06		0.44	T						
DUTCHTOWN #2	9.05					0.14	0.86	0.01							0.03						2.12	4.94	0.67		0.03	0.25						
GALLIANO	M 4.86				0.20		0.06	0.01	0.01							-			0.10		0.12	0.84	3.01	0.49	0.02					-		
GONZALES	8.66					0.13	0.17	0.05													2.08	3.37	1.85		0.07	0.94						
GRAND ISLE	M 2.44						0.01		0.10										0.10	0.01	0.40	0.02	-	0.80	0.70						0.30	
HOUMA	M	-	-	-	-	-	-	-	-	-	-	-	-	-	-	-	-	-	-	-	-	-	-	-	-	-	-	-	-	-	-	
LUTCHER	4.70						0.05	0.23	0.16	0.18		0.19			0.08					0.12	1.59	0.48	1.00		0.62	T						
MARRERO 9 SSW	M	-	-	-	0.61	T		-	-		T	0.37			-	-	-		0.19	0.04	0.95	-	-	-	0.09	0.57	0.05			-		
NEW ORLEANS AP	8.68			0.84	1.18	0.09	1.26		0.05										0.39	0.68	0.12	2.53	T	0.03	0.28						1.23	
NEW ORLEANS AUDUBON	5.85			0.52			0.09		0.01		0.02				0.43				0.25	1.20		3.03	0.10		0.16						0.04	
NEW ORLEANS ALGIERS	M	-	-	-	-	-	-	-	-	-	-	-	-	-	-	-	-	-	-	-	-	-	-	-	-	-	-	-	-	-	-	
NEW ORLEANS LKFRNT AP	6.66			1.19		0.12					T			0.11	1.45					0.49		2.99	0.23	T	0.04	T					0.04	
TERRYTOWN 3S	8.54			1.00		0.01		0.28				0.37						0.07	0.01	3.64		0.85	2.03			0.28						
THIBODAUX 4 SE	4.24				0.42	0.01	1.10	T										T			0.84	1.29	0.54	T	T	0.04						

LOUISIANA
201309

DAILY TEMPERATURES (°F)

STATION	OB.TIME	MAX/MIN	01	02	03	04	05	06	07	08	09	10	11	12	13	14	15	16	17	18	19	20	21	22	23	24	25	26	27	28	29	30	31	AVERAGE
**LOUISIANA**																																		
**NORTHWEST 01**																																		
BENTON 5E	08	MAX	99	100	90	98	96	97	96	97	97	96	95	89	97	93	89	94	94	94	96	96	76	81	81	84	90	86	90	89	87	75		91.4
		MIN	76	77	75	73	68	67	68	71	71	69	70	70	72	66	59	60	70	72	73	74	64	55	56	56	59	59	60	65	69	72		67.2
MANSFIELD 7 NW	08	MAX																											91	90	90			M
		MIN																											60	60	58			M
MINDEN	07	MAX	99	100	91	98	96	98	99	97	98	96	96	93	97	95	89	94	95	93	96	95	78	82	81	85	90	88	90	89	88	76		92.1
		MIN	72	73	75	75	71	70	70	70	69	69	69	70	70	68	63	64	70	74	74	74	68	54	55	58	58	58	59	63	66	72		67.4
MOORINGSPORT 1 N	08	MAX	100	101	90	100	96	97	98	97	97	95	95	94	97	94	89	93	97	92	96	94	75	80	81	83	91	90	90	88		76		91.9
		MIN	75	75	72	72	69	67	67	71	71	70	71	70	74	67	60	62	73	73	74	74	66	54	55	58	60	59	61	66		71		67.5
RED RIVER RSCH STN	07	MAX	98	99	93	100	98	99	99	97	96	95	95	92	96	96	91	94	96	93	97	97	78	85	83	87	93	90	91	90	90	78		92.9
		MIN	75	75	75	75	69	67	69	69	67	67	69	70	72	68	61	69	71	72	73	75	66	54	54	58	58	58	59	64	73	72		67.5
SHREVEPORT DWTN AP	24	MAX	100	93	101	98	99	100	99	96	97	97	93	98	97	91	95	96	95	97	96	77	84	83	87	93	90	93	90	90	78	83		92.9
		MIN	77	75	76	74	69	67	72	72	71	69	74	71	74	68	60	68	70	73	74	69	59	55	54	66	60	58	62	67	73	72		68.3
SHREVEPORT AP	24	MAX	103	95	104	100	101	101	99	99	98	97	93	99	98	91	96	96	96	97	97	77	85	84	87	94	92	93	91	91	79	85		93.9
		MIN	76	75	77	77	72	70	71	70	69	70	74	69	77	69	63	71	75	74	74	70	64	59	57	69	63	60	64	66	73	72		69.7
SHREVEPORT STHRN HILLS	07	MAX	102	103	95	102	100	101	101	100	98	98	98	95	99	97	91	94	97	93	96	97	76	84	82	86	92	91	92	89	90	77		93.9
		MIN	74	74	75	76	72	70	70	70	69	68	69	70	70	69	62	62	70	73	73	72	67	56	55	55	59	58	59	63	66	72		67.3
SHREVEPORT WFO	24	MAX	102	93	100	97	99	99	99	97	97	97	91	97	97	90	95	95	93	97	95	77	82	82	84	91	90	91	91	90	77	84		92.3
		MIN	76	76	78	78	73	73	73	72	70	72	75	72	76	70	65	73	76	76	75	70	67	59	57	69	63	64	64	68	73	72		70.8
**NORTH CENTRAL 02**																																		
BIENVILLE 3 NE	23	MAX	101	100	99	98	99	99	99	98	98	97	95	98	97	92	95	98	95	97	96	79	85	83	86	91	92	90	89	87	76	82		93.0
		MIN	74	73	72	73	69	68	69	69	68	67	74	68	71	67	64	68	71	73	73	69	65	58	57	68	58	58	61	63	72	71		67.7
CALHOUN RSCH STN	08	MAX	97	98	94	98	97	97	99	97	97	99	97	96	97	96	89	95	96	96	96	98	82	79	82	87	89	87	89	89	88	76		92.6
		MIN	68	71	69	69	64	64	66	65	63	63	66	65	65	64	54	65	65	65	68	69	69	53	50	50	59	56	56	58	69	69		63.2
COLUMBIA LOCK	07	MAX		98	98	98	95	98	98	96	96	97	98	93	96	89	87	93	95	94	95	94	86	78	79	86	87	88	89	89	89	84		91.8
		MIN		73	72	70	70	70	71	70	69	69	70	70	69	65	59	60	63	70	69	70	69	56	56	56	64	65	63	63	68	68		66.4
FARMERVILLE	07	MAX	98	101	93	97	88	98	99	98	98	97	93	93	97	93	87	94	96	95	95	95	79	81	80	85	89	86	87	87	87	76		91.4
		MIN	74	74	73	72	67	68	73	72	70	72	73	71	72	64	58	58	70	71	73	73	67	55	55	67	61	61	61	68	68	69		67.7
HOMER 1N	07	MAX	96	98	89	97	95	95	96	95	95	94	94	92	96	93	87	92	93	93	93	92	77	81	80	82	89		87	87	86	76		90.3
		MIN	70	71	71	69	63	63	66	66	65	64	70	67	67	65	53	63	63	68	70	73	68	49	48	59	54		54	57	61	70		63.7
MONROE REGIONAL AP	24	MAX	99	97	100	99	99	99	98	98	97	97	96	99	96	89	95	98	97	97	96	86	83	82	89	91	87	90	89	89	76	87		93.2
		MIN	70	73	71	72	68	66	69	67	66	69	72	67	72	64	59	68	69	69	70	71	60	56	55	64	64	58	61	67	69	70		66.5
RUSTON LA TECH	08	MAX	93	96	94	96	96	96	97	97	97	96	94	94	95	95	95	93	95	94	94	93	78	82	80	83	89	87	88	87	85	76		91.2
		MIN	72	72	70	71	66	66	66	66	67	67	66	66	66	66	57	60	66	69	69	72	68	53	52	52	52	52	58	60	66	70		64.1
WINNFIELD 3 N	24	MAX																																M
		MIN																																M
**NORTHEAST 03**																																		
BASTROP	07	MAX	98	99	92	96	96	96	97	96	97	97	97	95	97	92	86	92	96	94	95	94	83	80	78	84	86	85	86	86	87	75		91.1
		MIN	71	71	72	70	66	66	67	69	69	69	70	68	68	64	59	59	68	68	69	71	68	54	54	55	59	59	60	65	67	69		65.5
LAKE PROVIDENCE	07	MAX	95	97	90	94	94	95	94	95	95	93	96	93	94	94	87	92	95	94	94	94	83	80	81	87	85	87	86	87	85	76		90.4
		MIN	73	73	74	70	69	68	68	71	71	72	73	73	73	65	61	61	69	71	71	71	69	59	58	57	65	64	64	67	68	69		67.9

LOUISIANA
201309

DAILY TEMPERATURES (°F)

STATION	OB.TIME	MAX/MIN	01	02	03	04	05	06	07	08	09	10	11	12	13	14	15	16	17	18	19	20	21	22	23	24	25	26	27	28	29	30	31	AVERAGE
			DAY OF MONTH																															
RAYVILLE	07	MAX	98	100	94	97	98	99	98	98	98	99	98	95	98	94	88	94	97	96	96	95	97	79	81	88	89	88	90	89	90	77		93.3
		MIN	73	73	74	73	69	69	70	72	71	70	72	71	71	66	61	61	71	71	72	72	71	59	58	57	65	63	63	67	70	71		68.2
ST JOSEPH 3 N	08	MAX	95	97	98	98	98				95	97	97	95		95		88		97	95	95		88			89	88	88	89		85		M
		MIN	73	69	72	71	70	61	70	68	67	61	73	68		66	64	70	69	70	72	74	71	61	56		61	61	66	69	69	70		67.6
TALLULAH	08	MAX	93	94	83	96	96			95	94	96	95	92	95	93	85	93	94	94	95	95	81	79			84	86	86		85			90.8
		MIN	65	75	72	64	64			73	70	64	72	69	71	65	60	60	60	69	60	60	69	67	56	56	62	61	66		66			65.2
TALLULAH VICKSBURG RGN	24	MAX	95	96	96	97	97	97	96	96	97	97	95	96	95	88	93	95	96	93	95	87	80	81	88	84	87	88	89	87	77	86		91.5
		MIN	68	70	71	69	66	66	69	65	64	68	70	66	69	57	56	65	65	65	68	72	63	55	49	65	60	58	66	66	67	68		64.9
WINNSBORO 2 SE	08	MAX	98	99	99	99	99	102	99	100	99	99	99	96	98	100	92	97	98	98	96	96	88			89	90	90	91	89	90	75		95.2
		MIN	70	73	71	71	67	68	70	67	66	70	72	68	68	67	60	63	69	71	71	72	70			57	62	60	62	65	69	69		67.4
WINNSBORO 5 SSE	07	MAX	96	98	97	98	99	100	98	98	98	97	98	96	97	97	90	98	97	97	94	95	86	80	80	88	89							94.4
		MIN	69	72	70	68	68	69	69	67	66	69	70	67	69	66	61	59	68	68	69	73	69	56	54	55	60							66.0
**WEST CENTRAL 04**																																		
HODGES GARDENS	08	MAX	98	98	97	97	95	98	97	96	94	95	95	93	96	96	97	96	96	96	95	94	79	78	81	88	90	89	92	90	89	73		92.3
		MIN	72	72	73	74	72	72	70	70	70	71	71	73	73	67	66	70	72	74	73	73	68	57	56	57	64	67	68	68	67	68		68.9
LEESVILLE	08	MAX	97	98	101	97	94	98	95	93	94	95	95	94	96	98	92	94	96	95	96	94	81	76	82	88	88	91	93	89	90	75		92.2
		MIN	67	70	73	69	68	68	67	64	64	65	68	65	66	69	65	67	66	68	71	74	69	57	55	63	61	55	59	62	69	68		65.7
NATCHITOCHES #2	07	MAX	95	95	92	97	94	96	93	93	93	92	92	91	94	95	88	92	94	93	92	91	80	78	79	85	89	88	89	86	85	73		89.8
		MIN	73	74	74	73	72	71	71	69	69	69	72	70	73	67	64	67	71	72	71	73	68	57	56	60	62	60	62	65	67	69		68.0
TOLEDO BEND LAKE	08	MAX	96	96	98	93	95	99	98	96	94	95	96	95	94	97	94	95	100	95	97	97	80	79	84	87	89	90	92	92	90	74		92.6
		MIN	74	72	72	73	72	70	70	67	68	70	72	73	71	71	69	69	74	74	73	74	69	59	59	61	71	64	61	61	68	69		69.0
**CENTRAL 05**																																		
ALEXANDRIA	08	MAX	94	96	98	97	94	97	93	95	94	93	94	94	97	98	92	94	96	96	94	94	85	77	83	89	88	88	91	88	87	74		91.7
		MIN	73	73	75	74	74	73	73	71	71	71	72	72	73	71	69	69	73	74	74	74	62	62	61	63	65	63	64	66	70	69		69.8
ALEXANDRIA 5 SSE	24	MAX	97	97	96	95	95	92	93	93	93	95	92	94	97	90	91	93	93	93	92	85	76	80	85	85	86	85	83	83	83	78		89.7
		MIN	71	71	76	72	72	71	70	68	67	64	61	62	56	62	60	42 s	71	71	70	70	70	61	60	62	62	51	63	65	68	63		65.9
BOYCE 3 WNW	24	MAX	94	94	94	92	92	92	91	91	91	91	91	93	94	87	90	90	91	91	91	82	76	79	85	84	86	87	85	85	75	78		88.1
		MIN	74	75	78	74	74	73	73	72	71	72	74	74	75	68	67	67	74	74	73	70	67	60	60	72	68	64	66	68	70	69		70.5
BUNKIE	08	MAX	93	96	96	96	96	94	94	93	92	92	93	92	94	95	90	93	95	94	93	93	86	74	81	87	84	88	89	88	88	78		90.6
		MIN	71	73	75	71	71	71	70	67	66	69	71	69	70	69	67	69	69	71	72	75	71	63	61	63	65	61	63	64	69	69		68.5
EUNICE	08	MAX	96	95	97	97	96	92	94	95	93	93	93	93	96	97	93	94	95	95	95	93	84	72	82	83	91	91	91	89	88	82		91.5
		MIN	73	72	75	70	73	72	72	69	68	70	71	69	72	72	70	71	71	72	72	73	70	64	62	65	67	61	64	67	70	70		69.6
GRAND COTEAU	17	MAX	94	96	95	96	94	94	93	91	92	92	92	94	95	95	93	94	93	94	92	86	86	82	88	91	91	90	90	87	85	84		91.3
		MIN	73	73	75	71	71	72	72	68	67	66	71	69	70	73	70	70	70	71	72	72	70	65	63	75	66	60	63	68	69	69		69.5
JENA 4 WSW	08	MAX	94	97	100	101	97	99	97	97	97	96	96	95	98	100	93	96	98	97	96	95	86	78	82	80	87	88	90	89	89	74		92.7
		MIN	70	72	74	71	69	69	69	67	65	67	71	68	70	66	63	63	68	68	70	72	68	58	57	60	61	58	62	65	68	67		66.5
JONESVILLE LOCKS	06	MAX	92	95	95	95	96	96	96	94	94	93	93	97	96	96	87	97	95	95	95	92	86	86	80	88	85	85	87	88	88	89		91.7
		MIN	72	70	75	74	73	73	73	73	71	71	71	71	71	70	68	67	73	73	73	74	71	63	61	64	64	61	63	62	67	63		69.2
LSU DEAN LEE RSCH STN	08	MAX		96		93	94				82	93	93	93				96	94	94	93				81	84	82	86	87			86		M
		MIN		70		71	71				66	68	71	68				66	70	70	71				61	62	62	60	61			65		M
NEW ROADS 5 NE	24	MAX	97	95	97	94	93	94	94	95	96	97	95	96	94	94	95	95	95	93	96	92	80	82	91	91	89	88	91	89	81	85		92.1
		MIN	74	75	73	73	73	74	72	70	69	72	72	76	73	72	71	73	73	73	73	69	69	67	66	69	67	63	67	69	69	70		70.9

LOUISIANA
201309

DAILY TEMPERATURES (°F)

STATION	OB.TIME	MAX/MIN	01	02	03	04	05	06	07	08	09	10	11	12	13	14	15	16	17	18	19	20	21	22	23	24	25	26	27	28	29	30	31	AVERAGE
**EAST CENTRAL 06**																																		
BATON ROUGE METRO AP	24	MAX	94	94	95	93	92	92	93	92	91	93	92	94	94	91	92	93	94	91	92	92	80	83	88	91	90	89	89	88	84	85		90.7
		MIN	74	75	75	73	73	73	72	70	70	69	73	71	71	72	71	71	73	74	73	75	69	66	64	72	67	63	65	68	66	71		70.6
BOGALUSA	08	MAX																																M
		MIN																																M
CLINTON 5 SE	08	MAX	92	93	93	94	90	88	90	91	91	92	93	91	93	89	92	93	91	93	89	90	79	80	89	86	88	87	86	85	81	85		89.1
		MIN	69	71	67	69	70	70	68	66	65	67	67	67	69	69	68	68	69	71	71	71	68	65	62	63	64	60	61	66	64	64		67.0
HAMMOND 5 E	08	MAX	93	92	94	95	92	90	91	91	90	90	91	92	93	93	89	87	88	92	89	88	89	79	79	88	87	90	88	86	86	83		89.2
		MIN	70	71	73	70	71	71	69	66	67	68	69	68	68	69	69	69	70	71	71	72	72	67	63	66	65	59	63	66	62	67		68.1
LSU BEN-HUR FARM	08	MAX	92	93	93	94	95	93	92	93	93	92	92	92	94	95	89	92	92	92	90	88	89	78	78	87	89	90	88	88	87	84		90.1
		MIN	72	73	75	72	72	71	71	67	67	67	72	68	70	69	69	70	72	72	73	75	72	68	65	68	68	62	64	68	66	67		69.5
SLIDELL	08	MAX	90	91	92	92	89	87	88	91	89	89	88	90	90	90	90	89	89	91	89	82	87	78	78	87	87	86	86	84	84	84		87.6
		MIN	74	75	76	73	74	72	71	71	71	72	72	71	71	72	71	71	71	74	74	74	74	68	66	69	70	63	68	67	65	69		71.0
SLIDELL AP	24	MAX	92	93	92	90	89	91	92	90	91	90	92	94	92	89	91	92	91	87	87	90	82	81	88	89	89	89	86	85	86	82		89.1
		MIN	73	73	75	73	73	72	71	70	70	71	71	70	70	72	70	70	71	74	74	74	70	68	65	74	68	63	67	65	62	71		70.3
**SOUTHWEST 07**																																		
CROWLEY 2 NE	08	MAX	95	95	95	96	94	94	95	93	94	93	94	92	95	95	94	95	95	94	95	93	80	83	82	86	91	91	91	89	87	78		91.5
		MIN	71	71	74	71	73	72	72	67	68	70	72	69	70	72	71	71	70	73	73	74	72	66	64	68	65	61	64	68	70	71		69.8
DE RIDDER	08	MAX	96	97	98	97	95	95	95	95	94	94	95	94	96	96	97	93	97	94	94	91	80	74	83	85	85	90	91	89	89	77		91.5
		MIN	70	70	73	73	73	71	70	69	69	70	71	70	72	71	69	71	71	72	72	74	69	62	64	63	67	60	63	67	72	70		69.3
HACKBERRY 8 SSW	08	MAX	90	90	91	92	89	91	91	89	90	88	90	89	92	93	91	90	91	90	90	89	82	74	78	86	91	92	90	88	87	85		88.6
		MIN	78	78	78	78	75	77	77	76	75	76	76	76	75	76	73	75	76	77	77	74	73	68	66	68	72	73	73	75	79	72		74.7
JENNINGS	08	MAX	94	95	95	97	93	93	91	92	92	91	92	93	95	96	91	93	94	94	94	90	84	75	81	88	92	93	92	90	85	84		91.0
		MIN	73	73	73	73	73	73	73	70	70	71	72	71	73	73	72	72	73	73	74	75	73	66	65	70	68	63	63	70	72	72		71.1
LAKE ARTHUR 10 SW	08	MAX	95	94	94	96	96	94	91	91	91	91	92	92	93	93	91	91	94	93	92	89	78	81	82	88	92	95	93	88	86	85		90.7
		MIN	74	76	77	75	76	74	72	71	73	74	74	72	72	73	74	75	75	74	76	76	67	66	66	66	69	66	67	70	73	75		72.3
LAKE CHARLES AP	24	MAX	93	94	96	93	93	91	91	92	91	93	93	95	97	92	93	94	96	95	92	80	75	83	89	90	93	93	89	89	85	80		90.7
		MIN	74	74	75	75	76	75	74	70	72	73	74	73	74	75	74	73	74	75	76	73	72	67	67	71	66	67	69	71	72	72		72.4
LELAND BOWMAN LOCK	08	MAX	97	97	96	98	94	94	93	94	92	91	93	92	93	94	90	91	94	92	93	90	87	79	77	88	97	96	96	96	96	86		92.2
		MIN	76	77	77	76	76	77	76	70	72	73	74	73	74	74	74	74	74	75	76	76	75	71	67	67	72	70	70	70	70	75		73.4
MOSS BLUFF 2 NNW	24	MAX	96	97	98	96	93	93	93	92	92	94	94	97	97	92	95	95	96	95	91	80	79	84	89	90	93	92	89	91	84	76		91.4
		MIN	70	71	74	71	73	71	71	66	70	70	70	70	68	73	71	70	69	73	74	74	72	65	64	74	63	60	64	69	72	72		69.8
OBERLIN FIRE TWR	09	MAX	93		97	93	96	94	94	94	91	91	92	92	94	95	95	93	94	94	94	91	83	74	82	87	87	91	89	87	86	79		90.4
		MIN	71		75	70	72	72	71	68	68	68	70	69	69	70	68	69	69	71	73	74	68	64	63	66	65	60	64	66	70	70		68.7
ROCKEFELLER WL REFUGE	08	MAX	90	89	89	91	87	92	88	90	90	90	92	90	92	93	91	87	92	92	91	90	83	76	76	86	89	91	91	88	87	86		88.6
		MIN	73	76	76	74	74	74	74	70	72	72	73	72	72	73	72	73	72	73	74	76	71	68	64	68	69	68	69	70	71	73		71.9
VINTON 5W	08	MAX	96	96	97	99	95	98	94	88	93	92		94	95	97	92	93	95	96	96	93	78	74	83	89	91	93	93	91	91	85		92.0
		MIN	71	71	75	71	73	72	73	69	70	69		70	70	73	72	70	71	73	73	74	71	66	63	69	66	63	66	68	68	71		70.0
**SOUTH CENTRAL 08**																																		
CARVILLE 2 SW	24	MAX	92	92	93	94	92	90	90	89	89	89	89	91	91	91	90	90	91	88	86	89	79	77	86	89	88	88	86	85	84	81		88.3
		MIN	75	75	77	72	74	75	75	71	71	72	74	71	72	72	73	74	74	75	75	73	70	68	66	66	71	66	68	72	69	74		72.0
DONALDSONVILLE 4 SW	08	MAX	90	92	92	93	91	91	90	88	90	90	87	90	91	91	89	91	90	91	90	89	90	79	76	86	90	89	87	87	87	85		88.7

LOUISIANA
201309

DAILY TEMPERATURES (°F)

STATION	OB.TIME	MAX/MIN	01	02	03	04	05	06	07	08	09	10	11	12	13	14	15	16	17	18	19	20	21	22	23	24	25	26	27	28	29	30	31	AVERAGE
		MIN	70	73	73	72	72	72	72	68	67	69	70	69	68	69	71	70	70	72	74	74	72	69	66	66	67	62	63	65	66	67		69.3
FRANKLIN 3 NW	24	MAX	90	90	92	91	90	91	90	89	90	90	90	90	91	88	90	89	91	90	87	87	87	73	85	89	89	88	87	87	86	85		88.4
		MIN	72	74	74	73	74	74	71	69	71	72	72	72	71	73	72	73	72	74	75	76	71	68	65	74	69	65	66	70	69	72		71.4
JEANERETTE 5 NW	08	MAX	93	93	92	94	92	91	90	90	90	89	90	90	90	90	89	90	90	90	91	89	86	78	75	86	90	90	88	86	86	87		88.8
		MIN	71	72	72	73	73	73	73	68	68	69	71	70	70	70	72	72	72	72	75	75	72	68	65	65	69	63	63	65	65	65		69.7
LAFAYETTE	22	MAX	93	94	93	94	94	93	93	94	92	92	92	93	93	95	92	92	93	93	92	92	86	79	80	87	94	92	91	88	88	85		91.0
		MIN	74	74	75	75	75	75	73	72	69	69	72	73	73	73	73	73	74	74	74	75	75	68	65	65	68	64	64	64	70	72		71.3
LAFAYETTE FCWOS	24	MAX	95	95	93	94	94	94	94	93	92	93	92	94	96	92	93	93	94	94	93	87	79	80	88	93	93	91	89	88	86	83		91.2
		MIN	75	74	76	74	74	74	73	70	69	71	74	72	73	74	73	73	73	74	75	76	70	67	65	75	68	64	66	70	72	72		71.9
MORGAN CITY	08	MAX	91	91	94	93	91	91	92	93	94	90	90	89	91	91	90	90	94	91	90	90	90	83	75	80	82	86	90	89	88	88		89.2
		MIN	73	75	74	73	74	75	72	71	73	70	71	73	73	74	74	74	73	74	75	75	75	73	70	70	74	67	66	67	66	65		72.0
NEW IBERIA AP ACADIANA RGNL	24	MAX	97	96	98	97	97	95	96	95	93	95	94	95	96	94	94	95	96	95	94	89	82	80	89	94	93	93	92	90	89	86		93.0
		MIN	76	75	75	75	77	76	74	72	71	75	77	75	74	76	76	75	75	76	77	78	73	69	68	76	69	65	67	73	73	74		73.7
ST MARTINVILLE 3 SW	08	MAX	93	92	94	94	92	92	91	91	90	89	91	91	90	92	88	90	90	91	90	89	85	80	77	85	90	89	88	87	85	85		89.0
		MIN	73	72	74	72	72	72	68	66	67	69	70	70	69	72	71	70	70	71	74	75	73	69	64	66	65	61	63	66	69	71		69.5
**SOUTHEAST 09**																																		
BOOTHVILLE ASOS	24	MAX	88	88	88	89	88	88	90	89	88	89	88	88	89	89	88	89	89	88	87	87	83	79	86	87	85	86	86	85	86	81		87.0
		MIN	78	78	79	80	80	80	78	78	78	80	80	79	77	77	79	80	80	77	78	79	74	75	76	79	76	75	73	75	76	73		77.6
GALLIANO	08	MAX	90	92	92	90	89	87	89	88	88	88	89	89	89	89		89	89	88	87	88	87	78	74	85	89	88	88	86		86		87.5
		MIN	76	76	77	76	75	75	75	73	71	71	75	76	72	75		74	76	78	79	77	77	72	71	74	75	69	67	67		69		73.9
HOUMA	08	MAX																																M
		MIN																																M
MARRERO 9 SSW	08	MAX				92	93	90			91	91	88	90	91			93	90	89	89	89			90	88	89	90	90	89		88		M
		MIN				74	74	74			71	72	73	73	71			73	73	75	75	75			70	76	73	67	67	67		67		M
NEW ORLEANS AP	24	MAX	93	93	93	90	89	91	90	91	91	89	90	93	94	90	92	92	92	88	89	91	82	81	90	90	90	87	88	86	86	85		89.5
		MIN	75	76	75	75	75	76	73	72	72	73	75	73	74	78	76	75	76	77	76	78	73	73	73	77	74	68	70	69	67	74		73.9
NEW ORLEANS AUDUBON	24	MAX	95	96	95	93	91	91	93	91	91	90	91	93	95			91	91	88	88	90	82	82	88	91	87	88	90	88	88	82		90.0
		MIN	76	76	76	75	75	75	74	73	73	75	75	75	73				76	78	75	78	72	64	70	76	73	69	69	69	69	74		73.4
NEW ORLEANS LKFRNT AP	24	MAX	93	94	94	92	90	93	92	91	92	90	91	93	94	90	91	90	91	89	88	91	83	82	89	88	89	86	87	86	86	83		89.6
		MIN	81	81	76	80	81	81	80	80	80	80	80	79	80	77	79	79	81	81	77	79	74	73	74	78	77	74	75	75	73	77		78.1
TERRYTOWN 3S	07	MAX	94	95	95	94	93	91	91	92	90	91	90	90	91	93	90	92	91	91	89	89	90	79	80	89	91	91	89	89	87	87		90.1
		MIN	75	77	77	76	77	77	76	74	74	75	74	75	74	75	75	75	76	77	75	77	74	70	71	74	73	69	69	70	68	71		74.0
THIBODAUX 4 SE	08	MAX	90	91	90	93	91	87	87	89	89	88	88	88	89	90	88	90	88	89	87	86	87	76	74	85	89	88	87	86	86	85		87.4
		MIN	72	72	72	70	71	71	72	67	68	69	70	70	67	70	69	70	70	71	73	72	73	70	66	70	69	63	63	65	63	66		69.1

LOUISIANA
201309

DAILY SOIL TEMPERATURES

STATION	DEPTH	TIME	DAY OF MONTH																															AVERAGE
			01	02	03	04	05	06	07	08	09	10	11	12	13	14	15	16	17	18	19	20	21	22	23	24	25	26	27	28	29	30	31	
**LOUISIANA**																																		
**NORTHWEST 01**																																		
RED RIVER RSCH STN (IN)																																		
BARE GROUND	4	MAX	85	86	85	86	86	86	86	86	86	85	85	85	86	85	85	84	84	84	84	84	83	81	81	80	81	80	80	80	80	80		83.6
	4	MIN	83	83	83	83	84	83	84	83	84	83	83	83	83	83	83	81	82	83	82	82	80	79	79	78	79	78	78	78	78	79		81.5
**NORTH CENTRAL 02**																																		
CALHOUN RSCH STN (IN)																																		
SOD	4	MAX	88	89	86	88	88	86	88	86	87	87	87	86	86	85	84	84	85	85	85	86	83	80	78	78	80	80	80	81	80	79		84.2
	4	MIN	75	78	79	78	77	76	77	78	79	78	81	79	78	78	75	74	75	77	78	79	76	72	72	72	70	72	74	72	74	76		76.0
**NORTHEAST 03**																																		
ST JOSEPH 3 N (IN)																																		
BARE GROUND	2	MAX	105	104	103	101	103	108	106	104	102	107	101	104	-	103	103	98	102	104	103	104	88	82	87	-	93	90	94	93	96	81		98.9
	2	MIN	86	76	84	83	83	82	83	82	82	82	82	82	-	75	75	79	79	80	79	81	75	67	66	-	66	70	71	70	76	73		77.5
WINNSBORO 2 SE (IN)																																		
BARE GROUND	2	MAX	89	90	90	89	89	90	90	90	91	91	90	89	99	88	86	87	94	89	88	89	83	-	-	79	80	81	82	80	82	76		87.2
	2	MIN	77	77	78	78	77	77	78	78	77	77	78	77	77	75	73	73	76	77	77	78	76	-	-	67	71	70	70	71	74	74		75.3
WINNSBORO 5 SSE (IN)																																		
BARE GROUND	4	MAX	93	95	93	98	99	99	93	93	96	95	92	93	91	91	91	92	91	94	91	93	82	77	76	72	77	-	-	-	-	-		90.3
	4	MIN	79	80	80	79	79	79	79	80	80	79	80	80	80	80	74	75	80	79	79	79	76	68	66	65	70	-	-	-	-	-		77.0
**CENTRAL 05**																																		
LSU DEAN LEE RSCH STN (IN)																																		
**EAST CENTRAL 06**																																		
CLINTON 5 SE (IN)																																		
HAMMOND 5 E (IN)																																		
**SOUTHWEST 07**																																		
CROWLEY 2 NE (IN)																																		
JENNINGS (IN)																																		

LOUISIANA
201309

SOILS REFERENCE NOTES

STATION	SOIL TYPE	SOIL COVER	SLOPE	UNITS
RED RIVER RSCH STN	SANDY LOAM	BARE GROUND	00	F
CALHOUN RSCH STN	FINE SANDY LOAM	BARE GROUND	00	F
ST JOSEPH 3 N	SHARKEY CLAY	BARE GROUND	LEVEL	F
WINNSBORO 2 SE	SANDY LOAM	BARE GROUND	0	F
WINNSBORO 5 SSE	SANDY LOAM	BARE GROUND	00	F
LSU DEAN LEE RSCH STN	SANDY	BARE GROUND	00	F
CLINTON 5 SE	FINE SANDY LOAM	BARE GROUND	LEVEL NNW	F
HAMMOND 5 E	SANDY LOAM	BARE GROUND	00	F
CROWLEY 2 NE	SAND	SOD	1 DEG S	F
JENNINGS	SILT CLAY LOAM	SOD	0	F

LOUISIANA
201309

SNOWFALL AND SNOW ON GROUND (INCHES)

STATION		01	02	03	04	05	06	07	08	09	10	11	12	13	14	15	16	17	18	19	20	21	22	23	24	25	26	27	28	29	30	31
**LOUISIANA NORTHWEST 01**																																
BENTON 5E	SNOWFALL																															
	SN ON GND																															
HOSSTON	SNOWFALL																															
JAMESTOWN	SNOWFALL																															
KEITHVILLE	SNOWFALL																															
	SN ON GND																															
KORAN	SNOWFALL	-	-	-	-	-	-	-	-	-	-	-	-	-	-	-	-	-	-	-	-	-	-	-	-	-	-	-	-		-	
	SN ON GND	-	-	-	-	-	-	-	-	-	-	-	-	-	-	-	-	-	-	-	-	-	-	-	-	-	-	-	-		-	
LOGANSPORT	SNOWFALL																															
	SN ON GND																															
MANSFIELD 7 NW	SNOWFALL																														-	
	SN ON GND																														-	
MINDEN	SNOWFALL																															
	SN ON GND																															
MOORINGSPORT 1 N	SNOWFALL																															
	SN ON GND																															
RED RIVER RSCH STN	SNOWFALL																															
SHREVEPORT DWTN	SNOWFALL																															
	SN ON GND																															
SHREVEPORT DWTN AP	SNOWFALL																															
SHREVEPORT AP	SNOWFALL																															
	SN ON GND																															
SHREVEPORT STHRN HILLS	SNOWFALL																															
	SN ON GND																															
SHREVEPORT WFO	SNOWFALL																															
	SN ON GND																															
SPRINGHILL	SNOWFALL																															
VIVIAN	SNOWFALL	-	-	-	-	-	-	-	-	-	-	-	-	-	-	-	-	-	-	-	-	-	-	-	-	-	-	-	-			
	SN ON GND	-	-	-	-	-	-	-	-	-	-	-	-	-	-	-	-	-	-	-	-	-	-	-	-	-	-	-	-			

Snowfall: Includes snow and ice. Values for NWS stations (J index note) are Mid-Mid (LST).
Snow on ground: Includes snow, sleet, ice, and hail. Values for NWS stations (J index note) are observed at 12 UTC (GMT).
Water Equivalent: Given for NWS stations (J index note) only, when snow depth is 2 inches or more, and is measured at 18 UTC (GMT)

LOUISIANA
201309

PAN EVAPORATION AND WIND

STATION		01	02	03	04	05	06	07	08	09	10	11	12	13	14	15	16	17	18	19	20	21	22	23	24	25	26	27	28	29	30	31	TOTAL OR AVERAGE
LOUISIANA NORTHWEST 01																																	
RED RIVER RSCH STN	WIND	42	34	27	33	35	20	-	22	25	37	29	14	19	48	48	25	20	22	52	76	37	21	29	33	19	18	78	32	102	37		1070E
	EVAP	0.32	0.30	0.22	0.32	0.30	0.30	0.27	0.28	0.29	0.32	0.23	0.25	0.15	0.33	0.33	0.25	0.23	0.19	0.29	0.15	0.00	0.22	0.23	0.30	0.17	0.24	0.25	0.27	0.00	0.10		7.10
	MAX	-	-	-	-	-	-	-	-	-	-	-	-	-	-	-	-	-	-	-	-	-	-	-	-	-	-	-	-	-	-		M
	MIN	-	-	-	-	-	-	-	-	-	-	-	-	-	-	-	-	-	-	-	-	-	-	-	-	-	-	-	-	-	-		M
NORTH CENTRAL 02																																	
CALHOUN RSCH STN	WIND	-	-	-	-	-	-	-	-	-	-	-	-	-	-	-	-	-	-	-	-	-	-	-	-	-	-	-	-	-	-		M
	EVAP	-	-	-	-	-	-	-	-	-	-	-	-	-	-	-	-	-	-	-	-	-	-	-	-	-	-	-	-	-	-		M
	MAX	-	-	-	-	-	-	-	-	-	-	-	-	-	-	-	-	-	-	-	-	-	-	-	-	-	-	-	-	-	-		-
	MIN	-	-	-	-	-	-	-	-	-	-	-	-	-	-	-	-	-	-	-	-	-	-	-	-	-	-	-	-	-	-		M
NORTHEAST 03																																	
ST JOSEPH 3 N	WIND	-	-	-	5	3	4	-	-	-	9	7	6	-	-	-	-	1	1	7	28	-	-	-	-	-	13	3	-	-	-		M
	EVAP	0.00	0.00	0.00	0.00	0.00	0.00	0.00	0.00	0.00	0.00	0.00	0.00	-	0.00	0.00	0.00	0.00	0.00	0.00	0.00	0.00	0.00	0.00	-	0.00	0.19	0.19	0.00	0.00	0.00		0.41E
	MAX	-	-	-	-	-	-	-	-	-	-	-	-	-	-	-	-	-	-	-	-	-	-	-	-	-	-	-	-	-	-		-
	MIN	-	-	-	-	-	-	-	-	-	-	-	-	-	-	-	-	-	-	-	-	-	-	-	-	-	-	-	-	-	-		M
WEST CENTRAL 04																																	
TOLEDO BEND LAKE	WIND	80	69	40	49	39	58	60	48	57	82	84	40	41	51	-	59	45	48	116	122	102	37	32	47	-	38	48	82	115	51		1864E
	EVAP	0.43	0.39	0.29	0.26	0.22	0.34	0.27	0.25	0.37	0.43	0.38	0.33	0.27	0.35	-	0.24	0.32	0.28	0.38	0.31	0.00	0.22	0.26	0.31	-	0.39	0.29	0.32	0.24	0.10		8.83E
	MAX	-	-	-	-	-	-	-	-	-	-	-	-	-	-	-	-	-	-	-	-	-	-	-	-	-	-	-	-	-	-		-
	MIN	-	-	-	-	-	-	-	-	-	-	-	-	-	-	-	-	-	-	-	-	-	-	-	-	-	-	-	-	-	-		M
SOUTHWEST 07																																	
JENNINGS	WIND	43	53	53	36	26	-	39	39	40	40	47	39	35	38	34	36	41	32	69	69	32	38	38	43	24	25	30	42	28	35		1183E
	EVAP	0.23	0.31	0.29	0.26	0.19	-	0.27	0.20	0.25	0.23	0.22	0.23	0.27	0.26	0.18	0.22	0.22	0.21	0.27	0.26	0.00	0.05	0.21	0.09	0.19	0.24	0.19	0.13	0.22	0.07		6.17E
	MAX	95	95	94	99	97	-	98	98	98	95	94	96	96	97	92	96	95	96	95	93	82	77	82	88	95	94	92	93	86	83		-
	MIN	75	73	76	72	72	-	72	71	70	70	72	71	71	73	70	70	73	72	73	72	72	65	64	64	70	64	64	67	71	70		70.3

Evaporation: Is measured in hundreths of inches.
Wind: Is measured in miles.
Max and Min: The maximum and minimum tempertures (Fahrenheit) of the water in the evaporation pan.

STATION INDEX

STATION	INDEX NO.	DIVISION	COUNTY	LATITUDE	LONGITUDE	ELEVATION (IN FEET)	OBSERVATION TIME AND TABLES LOCAL STD TIME TEMP	PRECIP	EVAP	SPECIAL SEE (NOTES)
LOUISIANA										
ABBEVILLE	0007	07	VERMILION	29 58	92 7W	10		08		H
ABITA RVR COVINGTON	0012	06	ST. TAMMANY	30 28	90 6W	3		07		H
ABITA SPRING FIRE TWR	0021	06	ST. TAMMANY	30 26	90 3W	30		13		H
ABITA SPRINGS 1 SW	0016	06	ST. TAMMANY	30 28	90 3W	25		07		H
ALEXANDRIA	0098	05	RAPIDES	31 19	92 28W	87	08	08		H
ALEXANDRIA 5 SSE	0103	05	RAPIDES	31 15	92 27W	85	24	24		CH
ANGIE	0238	06	WASHINGTON	30 58	89 49W	130		08		H
ARCADIA	0277	02	BIENVILLE	32 33	92 55W	400		08		H
BAKER	0462	06	EAST BATON ROUGE	30 34	91 10W	70		08		H
BASTROP	0537	03	MOREHOUSE	32 44	91 55W	150	07	07		H
BATON ROUGE CONCORD	0548	06	EAST BATON ROUGE	30 25	91 8W	50		08		H
BATON ROUGE METRO AP R	0549	06	EAST BATON ROUGE	30 32	91 9W	64	24	24		HJ
BATON ROUGE SHERWOOD	0558	06	EAST BATON ROUGE	30 27	91 3W	55		08		H
BAYOU SORREL LOCK	0565	08	IBERVILLE	30 8	91 19W	15		08		H
BEAVER FIRE TWR	0617	05	EVANGELINE	30 48	92 30W	105		13		H
BELL CITY 13 SW	0658	07	CAMERON	29 58	93 5W	4		07		H
BENTON 5E	0718	01	BOSSIER	32 27	93 50W	200	08	08		H
BIENVILLE 3 NE	0800	02	BIENVILLE	32 22	92 57W	307	23	23		H
BOGALUSA	0945	06	WASHINGTON	30 47	89 51W	100	08	08		H
BOOTHVILLE ASOS R	1157	09	PLAQUEMINES	29 20	89 24W	3	24	24		H
BOYCE 3 WNW	1232	05	RAPIDES	31 24	92 43W	110	24	24		H
BUNKIE	1287	05	AVOYELLES	30 58	92 11W	80	08	08		CH
CALHOUN RSCH STN	1411	02	OUACHITA	32 31	92 21W	180	08	08	08	GCH
CARENCRO	1535	08	LAFAYETTE	30 19	92 3W	50		07		H
CARVILLE 2 SW	1565	08	IBERVILLE	30 12	91 8W	25	24	24		H
CLAYTON	1866	05	CONCORDIA	31 43	91 32W	73		07		CH
CLINTON 5 SE	1899	06	EAST FELICIANA	30 49	90 58W	200	08	08		GCH
CLINTON FORESTRY HQ	1891	06	EAST FELICIANA	30 51	91 1W	250		13		H
COLUMBIA LOCK	1979	02	CALDWELL	32 10	92 6W	80	07	07		H
CONVENT 2S	2002	09	ST. JAMES	29 60	90 49W	25		08		H
COVINGTON 3 NE	2154	06	ST. TAMMANY	30 31	90 5W	25		07		H
CROWLEY 2 NE	2212	07	ACADIA	30 14	92 21W	25	08	08		G H
DE RIDDER	2367	07	BEAUREGARD	30 51	93 17W	190	08	08		H
DENHAM SPRINGS	2350	06	LIVINGSTON	30 29	90 58W	35		07		H
DONALDSONVILLE 4 SW	2534	08	ASSUMPTION	30 4	91 2W	30	08	08		CH
DRY CREEK 8NW	2641	07	BEAUREGARD	30 44	93 8W	95		07		H
DUTCHTOWN #2	2688	09	ASCENSION	30 15	90 59W	18		07		H
EUNICE	2981	05	ST. LANDRY	30 29	92 26W	50	08	08		H
FARMERVILLE	3079	02	UNION	32 47	92 24W	180	07	07		H
FRANKLIN 3 NW	3313	08	ST. MARY	29 49	91 33W	12	24	24		H
GALLIANO	3433	09	LAFOURCHE	29 28	90 18W	5	08	08		H
GONZALES	3695	09	ASCENSION	30 12	90 55W	10		07		H
GORUM FIRE TWR	3741	04	NATCHITOCHES	31 26	92 53W	307		13		H
GRAND COTEAU	3800	05	ST. LANDRY	30 25	92 3W	55	17	17		H
GRAND ISLE	3807	09	JEFFERSON	29 14	89 59W	2		07		H
HACKBERRY 8 SSW	3979	07	CAMERON	29 53	93 24W	6	08	08		H
HAMMOND 5 E	4030	06	TANGIPAHOA	30 30	90 23W	35	08	08		GCH
HODGES GARDENS	4288	04	SABINE	31 22	93 23W	420	08	08		H
HOMER 1N	4355	02	CLAIBORNE	32 49	93 4W	215	07	07		H
HOSSTON	4398	01	CADDO	32 27	93 50W	246		08		H
HOUMA	4407	09	TERREBONNE	29 38	90 49W	8	08	08		H
JAMESTOWN	4592	01	BIENVILLE	32 21	93 12W	190		07		H
JEANERETTE 5 NW	4674	08	IBERIA	29 58	91 43W	20	08	08		H
JENA 4 WSW	4696	05	LA SALLE	31 38	92 12W	210	08	08		CH
JENNINGS	4700	07	JEFFERSON DAVIS	30 12	92 40W	25	08	08	08	GCH
JONESBORO 4 ENE	4732	02	JACKSON	32 15	92 39W	330		13		H
JONESVILLE LOCKS	4739	05	CATAHOULA	31 29	91 52W	70	06	06		CH
KAPLAN	4775	07	VERMILION	29 60	92 17W	15		07		H
KEITHVILLE	4816	01	CADDO	32 21	93 52W	200		07		H
KILLIAN	4878	06	LIVINGSTON	30 22	90 33W	10		08		H
KORAN	4931	01	BOSSIER	32 25	93 28W	175		08		H
LAFAYETTE	5021	08	LAFAYETTE	30 13	92 4W	25	22	22		CH
LAFAYETTE FCWOS R	5026	08	LAFAYETTE	30 12	91 59W	38	24	24		H
LAKE ARTHUR 10 SW	5065	07	CAMERON	30 0	92 47W	10	08	08		H
LAKE CHARLES 2 N	5074	07	CALCASIEU	30 15	93 13W	5		08		H

STATION INDEX

STATION	INDEX NO.	DIVISION	COUNTY	LATITUDE	LONGITUDE	ELEVATION (IN FEET)	OBSERVATION TIME AND TABLES			
							LOCAL STD TIME			
							TEMP	PRECIP	EVAP	SPECIAL SEE (NOTES)
LAKE CHARLES 7 NW	5072	07	CALCASIEU	30 18	93 16W	10		08		H
LAKE CHARLES AP R	5078	07	CALCASIEU	30 7	93 14W	9	24	24		HJ
LAKE CHARLES PORT	5076	07	CALCASIEU	30 13	93 15W	5		08		H
LAKE PROVIDENCE	5090	03	EAST CARROLL	32 48	91 10W	100	07	07		H
LEESVILLE	5266	04	VERNON	31 8	93 14W	28	08	08		H
LEESVILLE 6 SSW	5287	04	VERNON	31 3	93 17W	260		08		CH
LELAND BOWMAN LOCK	5296	07	VERMILION	29 47	92 12W	40	08	08		H
LIVERPOOL 6W	5430	06	ST. HELENA	30 56	90 34W	250		08		H
LIVINGSTON	5438	06	LIVINGSTON	30 31	90 45W	43		06		H
LOGANSPORT	5522	01	DE SOTO	31 58	94 0W	190		07		H
LSU BEN-HUR FARM	5620	06	EAST BATON ROUGE	30 22	91 10W	21	08	08		CH
LSU DEAN LEE RSCH STN	5630	05	RAPIDES	31 11	92 25W	70	08	08		G H
LUTCHER	5783	09	ST. JAMES	30 2	90 42W	20		07		H
MANSFIELD 7 NW	5875	01	DE SOTO	32 8	93 45W	255	08	08		CH
MANY 9 WSW	5896	04	SABINE	31 31	93 37W	286		07		H
MARKSVILLE	5920	05	AVOYELLES	31 8	92 4W	85		08		H
MARRERO 9 SSW	5926	09	JEFFERSON	29 47	90 7W	3	08	08		H
MINDEN	6244	01	WEBSTER	32 36	93 18W	185	07	07		CH
MONROE DELTA CC	6314	02	OUACHITA	32 30	92 2W	70		08		CH
MONROE REGIONAL AP R	6303	02	OUACHITA	32 31	92 2W	79	24	24		H
MOORINGSPORT 1 N	6364	01	CADDO	32 42	93 58W	200	08	08		H
MORGAN CITY	6394	08	ST. MARY	29 41	91 11W	5	08	08		CH
MOSS BLUFF	6431	07	CALCASIEU	30 18	93 12W	19		07		H
MOSS BLUFF 2 NNW	6434	07	CALCASIEU	30 20	93 13W	25	24	24		H
MOUNT HERMON 2W	6466	06	WASHINGTON	30 57	90 18W	320		08		H
NAPOLEONVILLE	6561	08	ASSUMPTION	29 56	91 1W	25		07		H
NATCHITOCHES #2	6584	04	NATCHITOCHES	31 49	93 5W	141	07	07		CH
NEW IBERIA AP ACADIANA RGNL R	6657	08	IBERIA	30 2	91 53W	24	24	24		H
NEW ORLEANS ALGIERS	6666	09	ORLEANS	29 57	90 3W	2		08		H
NEW ORLEANS AP R	6660	09	JEFFERSON	29 60	90 15W	4	24	24		HJ
NEW ORLEANS AUDUBON R	6664	09	ORLEANS	29 55	90 8W	20	24	24		H
NEW ORLEANS LKFRNT AP R	6667	09	ORLEANS	30 3	90 2W	9	24	24		H
NEW ROADS 5 NE	6686	05	POINTE COUPEE	30 44	91 22W	45	24	24		H
NORWOOD	6808	06	EAST FELICIANA	30 58	91 6W	102		08		H
OAK GROVE	6866	03	WEST CARROLL	32 52	91 23W	129		08		H
OAK RIDGE	6868	03	MOREHOUSE	32 37	91 47W	82		07		H
OAKDALE	6836	07	ALLEN	30 49	92 40W	110		07		H
OAKNOLIA 2N	6911	06	EAST FELICIANA	30 45	90 60W	150		07		H
OBERLIN FIRE TWR	6938	07	ALLEN	30 36	92 46W	65	09	09		H
OLD TOWN BAY	6968	07	CALCASIEU	30 17	93 9W	12		07		H
OPELOUSAS	6995	05	ST. LANDRY	30 30	92 6W	56		07		H
PINE GROVE FIRE TWR	7304	06	ST. HELENA	30 43	90 45W	190		13		H
PIONEER 6 W	7312	03	WEST CARROLL	32 45	91 32W	88		08		H
PLAQUEMINE 2 N	7366	08	IBERVILLE	30 19	91 15W	20		07		H
PONCHATOULA 4 SE	7425	06	TANGIPAHOA	30 25	90 23W	18		07		H
PORT ALLEN	7448	05	WEST BATON ROUGE	30 27	91 13W	15		07		H
RAYVILLE	7691	03	RICHLAND	32 30	91 45W	89	07	07		CH
RED RIVER LOCK # 2	7732	05	RAPIDES	31 11	92 17W	75		07		H
RED RIVER LOCK #1	7729	05	CATAHOULA	31 15	91 58W	70		07		H
RED RIVER RSCH STN	7738	01	BOSSIER	32 25	93 38W	155	07	07	07	GCH
ROCKEFELLER WL REFUGE	7932	07	CAMERON	29 44	92 49W	4	08	08		H
RUSTON LA TECH	8067	02	LINCOLN	32 32	92 41W	260	08	08		H
SAILES FIRE TWR	8094	02	BIENVILLE	32 22	93 9W	360		13		H
SHREVEPORT AP R	8440	01	CADDO	32 27	93 49W	254	24	24		HJ
SHREVEPORT DWTN	8436	01	CADDO	32 31	93 45W	180		07		H
SHREVEPORT DWTN AP R	8438	01	CADDO	32 33	93 45W	179	24	24		H
SHREVEPORT STHRN HILLS	8444	01	CADDO	32 24	93 47W	200	07	07		CH
SHREVEPORT WFO	8448	01	CADDO	32 27	93 50W	274	24	24		H
SLIDELL	8539	06	ST. TAMMANY	30 16	89 46W	10	08	08		CH
SLIDELL AP R	8543	06	ST. TAMMANY	30 21	89 49W	27	24	24		H
SPRINGHILL	8683	01	WEBSTER	32 60	93 27W	240		07		H
ST FRANCISVILLE	8136	06	WEST FELICIANA	30 47	91 23W	115		07		H
ST GABRIEL	8139	08	IBERVILLE	30 16	91 6W	30		08		H
ST JOSEPH 3 N	8163	03	TENSAS	31 57	91 14W	78	08	08	07	G H
ST MARTINVILLE 3 SW	8181	08	ST. MARTIN	30 5	91 52W	30	08	08		H
SULPHUR	8831	07	CALCASIEU	30 14	93 21W	10		24		H

STATION INDEX

STATION	INDEX NO.	DIVISION	COUNTY	LATITUDE	LONGITUDE	ELEVATION (IN FEET)	OBSERVATION TIME AND TABLES LOCAL STD TIME TEMP	PRECIP	EVAP	SPECIAL SEE (NOTES)
SUN	8861	06	ST. TAMMANY	30 39	89 55W	75		06		H
TALISHEEK	8906	06	ST. TAMMANY	30 31	89 52W	60		08		H
TALLULAH	8923	03	MADISON	32 24	91 11W	85	08	08		H
TALLULAH VICKSBURG RGN R	8926	03	MADISON	32 21	91 2W	86	24	24		H
TERRYTOWN 3S	8941	09	JEFFERSON	29 55	90 2W	10	07	07		H
THIBODAUX 4 SE	9013	09	LAFOURCHE	29 45	90 46W	15	08	08		CH
TICKFAW 3 ENE	8945	06	TANGIPAHOA	30 36	90 27W	53		24		H
TOLEDO BEND LAKE	9074	04	SABINE	31 12	93 34W	181	08	08	08	H
VILLE PLATTE	9369	05	EVANGELINE	30 42	92 16W	70		07		H
VINTON 5W	9376	07	CALCASIEU	30 12	93 41W	11	08	08		H
VIVIAN	9392	01	CADDO	32 54	93 59W	220		07		H
WEST MONROE	9631	02	OUACHITA	32 28	92 9W	75		07		H
WINNFIELD 3 N R	9803	02	WINN	31 58	92 39W	160	24	24		H
WINNSBORO 2 SE	9804	03	FRANKLIN	32 8	91 43W	74	08	08		G H
WINNSBORO 5 SSE	9806	03	FRANKLIN	32 6	91 42W	80	07	07		GCH
ZWOLLE 2 NW	9980	04	SABINE	31 40	93 40W	209		07		H

REFERENCE NOTES

STATION NAMES: Name of the city, town or locality. Figures and letters following the station names indicate the distance in miles and direction from the post office or town community center.

DIVISIONS: Areas within a state of similar climatological characteristics. Division averages are calculated using data from stations that record temperature and/or precipitation. Station Precipitation totals flagged with an 'F' or 'M' are excluded from the Divisional Average calculations of precipitation. Stations with monthly Temperature averages flagged with an 'F' or 'M' are included in the Divisional Average if there are no more than 9 flagged or missing daily values in the month, else they are excluded from the divisional average for temperature.

NORMALS: The average value of the meteorological element over a time period. Effective 1 January 2012, the averaging period is 1981 to 2010. The normals for National Weather Service localities have been adjusted so as to be representative for the current observation site.

MONTHLY DEGREE DAY TOTALS: One heating (cooling) degree day is accumulated for each whole degree that the daily mean temperature is below (above) 65 degrees Fahrenheit.

PRECIPITATION: Values shown in hundredths of inches are water equivalent totals, i.e., total of liquid and melted frozen precipitation. In the "Monthly Summarized Data" table the total snow and sleet values shown in tenths of inches are unmelted amounts. The max depth on ground values of snow and sleet shown in whole inches are cumulative unmelted amounts. The number of days with .10, .50, 1.00 or more refers to water equivalents.

PRECIPITATION QUALITY CONTROL: The NCDC quality control process may flag precipitation data that are spatially inconsistent, exceed climatological limits, or are inconsistent with prevailing weather patterns.

TEMPERATURE: Original temperature values are given in the "Daily Temperature" table. Summary temperature information (averages, departures, extremes, monthly degree day totals) is based on the values labeled MAX/MIN.

WIND: (As shown in the "Evaporation and Wind" table) the total wind movement in miles over the evaporation pan as determined by an anemometer recorder located 6-8 inches above the pan.

SYMBOLS AND LETTERS USED IN THE STATION INDEX TABLE

C Station is equipped with recording rain gage (R) but values in this bulletin are from a non-recording rain gage unless indicated by an R.
G Observations appear in the "Soil Temperatures" table.
H Observations appear in the "Snowfall and Snow on the Ground" table.
J Station also published as a Local Climatological Data publication.
VAR Observation time varies.
SR Observation time near sunrise.
SS Observation time near sunset.

SYMBOLS AND LETTERS USED IN THE DATA TABLES

(DAILY DATA ARE FOR THE 24 HOURS IMMEDIATELY PRECEDING OBSERVATION TIME.)
BLANK Entries in the "Monthly Summarized Data" table indicate no record.
BLANK Entries in the "Daily Precipitation" and "Snowfall and Snow on the Ground" tables indicate zero.
BLANK Entries in the "Daily Temperature" table indicate a missing record

- No record. Data not recorded or not received in time for publication.
+ Precipitation or temperature extremes occurred on one or more previous dates during the month.
* Rain gage not read. Precipitation is included in the amount following the asterisks. Time distribution may not be known. A * preceding the monthly total indicates precipitation amount is being carried forward to next month's total, and may include amounts from the previous month(s).
a As a subscript, indicates accumulated total.

A Amount of precipitation is the total of observer's entries for the current month. It may include precipitation that occurred during the previous month. Refer to earlier bulletin to determine date of last reading. (Hawaii stations)
B Divisional Departure from normals are computed using 1971-2000 normals.
E Normalized HDD/CDD Calculation. E is appended to the HDD/CDD Calculation when 1-9 individual daily TMAX and/or TMIN values are missing and a Normalized HDD/CDD Calculation is provided. M appears alone if 10 or more daily values are missing.
F Monthly calculation flagged value. F is appended to average and/or total values computed which exclude one or more daily data values that have been flagged by the GHCN-Daily Dataset
M Insufficient or partial data. M is appended to average and/or total values computed with 1-9 daily values missing. M appears alone if 10 or more daily values are missing, (8 or more for wind and evaporation).
N Indicates snow fall or Snowdepth totals are computed with one or more missing days.
R Amounts from recording rain gage.
T Trace. An amount too small to measure.

SEASONAL TABLES: Monthly and seasonal snowfall and heating degree days for the 12 months ending with the June data are published in the July issue of this bulletin. Cooling degree days for the calendar year are published in the "Climatological Data Annual Summary."

Information concerning the history of changes in locations, exposure, etc. of substations is kept on file at the National Climatic Data Center. Historical information of regular National Weather Service Offices may be obtained from the "Local Climatological Data" annual publication. The contents of this publication may be reprinted or otherwise used freely, with proper credit to the National Climatic Data Center. The data are also available digitally.

Effective with the January 2011 Data-Month, COOP Observer Names are no longer included in the Monthly and Annual Climatological Data Publications. This information is not published to ensure the privacy of personal information pursuant to Section 208 of the E-Government Act of 2002 (44 USC 3601).

As of the 2011 Data-Year, Station and Climate Division Maps are no longer being included in the CD Publications. NCDC's Product Development Branch provides updated Station Maps for various data networks via the Historical Observing Metadata Repository: http://www.ncdc.noaa.gov/homr.

The GHCN-Daily Quality Control Flags shown below are displayed as superscripts with the data. For more Information on Global Historical Climatology Network - Daily and flags, see:
http://www.ncdc.noaa.gov/oa/climate/ghcn-daily/
and
Comprehensive Automated Quality Assurance of Daily Surface Observations.
Durre, Imke, Matthew J. Menne, Byron E. Gleason, Tamara G. Houston,
Russell S. Vose, 2010: J. Appl. Meteor. Climatol., 49, 16151633.
doi: 10.1175/2010JAMC2375.1

Blank = Passed All checks	N = failed naught check
D = failed duplicate check	O = failed climatological outlier check
G = failed gap check	R = failed lagged range check
I = failed internal consistency check	S = failed spatial consistency check
K = failed streak/frequent-value check	T = failed temporal consistency check
L = failed check on length of multiday period	W = temperature too warm for snow
M = failed megaconsistency check	X = failed bounds check
	Z = flagged as a result of an official Datzilla investigation

Beginning with the January 2013 CD Publication, monthly mean temperature calculations have changed to the National Data Stewardship Team standard. Monthly maximum and minimum temperatures are not rounded until after the monthly mean temperature is calculated. This is the most accurate outcome, but may be slightly different from the mean derived from rounded monthly maximum and minimum.

Processing Updates and Errata: The 2011 CD Publications were reproduced in May 2013. This update included the addition of late reports and corrections based on additional investigations of reported data issues through NCDCs Datzilla system. In addition, divisional averages for precipitation were recalculated using the method described in DIVISIONS above. Previous editions of the 2011 Publications included all precipitation stations regardless of missing data in the calculation of divisional averages. HDD/CDD values were recalculated to match the legacy method of calculation (truncation of monthly HDD/CDD values instead of rounding).

These and other publications are available from the National Climatic Data Center

Hourly Precipitation Data
This publication contains hourly precipitation amounts obtained from recording rain gages located at National Weather Service, Federal Aviation Administration, and cooperative observer stations. Published data are displayed in inches and tenths or inches and hundredths at local standard time. HPD includes maximum precipitation for nine (9) time periods from 15 minutes to 24 hours, for selected stations.

Climatological Data
Monthly editions contain station daily maximum and minimum temperatures and precipitation. Some Stations provide daily snowfall, snow depth, evaporation, and soil temperature data. Each edition also contains monthly summaries for heating and cooling degree days (65 degree F base). The July issue contains a recap of monthly heating degree days and snow data for the preceding July through June.

The Annual issue contains monthly and annual averages of temperature, precipitation, temperature extremes, freeze data, soil temperatures, evaporation, and a recap of monthly cooling degree days.

Storm Data
Monthly issues contain a chronological listing, by states, of occurrences of storms and unusual weather phenomena. Reports contain information on storm paths, deaths, injuries, and property damage. An "Outstanding storms of the month" section highlights severe weather events with photographs, illustrations, and narratives. The December issue includes annual tornado, lightning, flash flood, and tropical cyclone summaries.

Monthly Climatic Data for the World
This publication contains monthly means for temperature, pressure, precipitation, vapor pressure, and sunshine for approximately 2,000 surface data collection stations worldwide and monthly mean upper air temperatures, dew point depressions, and wind velocities for approximately 500 observing sites.

Local Climatological Data
LCD publications summmarize temperature, relative humidity, precipitation, cloudiness, wind speed and direction observations for several hundred cities in the U.S. and its territories. Each monthly publication also contains 3 hourly weather observations for that month and a hourly summary of precipitation. Annual LCD publications contain a summary of the past calendar year as well as historical averages and extremes.

For Information Call:
(828) 271-4800 Option 2
(828) 271-4010 (TDD)
(828) 271-4876 (Fax)

NOAA\National Climatic Data Center
Attn: User Engagement & Services Branch
151 Patton Avenue
Asheville, NC 28801-5001

Customer Services Number: (828) 271-4800, option 2
TDD : (828) 271-4010
Fax number: (828) 271-4876

NCDC now offers free online access to the ***Climatological Data*** **publication.**
Go to : **www.ncdc.noaa.gov**   and choose Most Popular.
