# Supplementary material for: Why is the lawn buzzing?
Source: Biodivers Data J. 2014 Apr 24;(2):e1101. doi: 10.3897/BDJ.2.e1101 (PMC4040422; doi:10.3897/BDJ.2.e1101)
Supplement: Supplementary material 5 — Climatological Data for Louisiana, October 2013 [file biodiversity_data_journal-2-e1101-s005.pdf]

CLIMATOLOGICAL DATA

LOUISIANA

OCTOBER 2013

VOLUME 118 NUMBER 10
ISSN 0145-0409
GHCND Ver: 3.12-upd-2014012106

OCTOBER PRECIPITATION BY YEAR

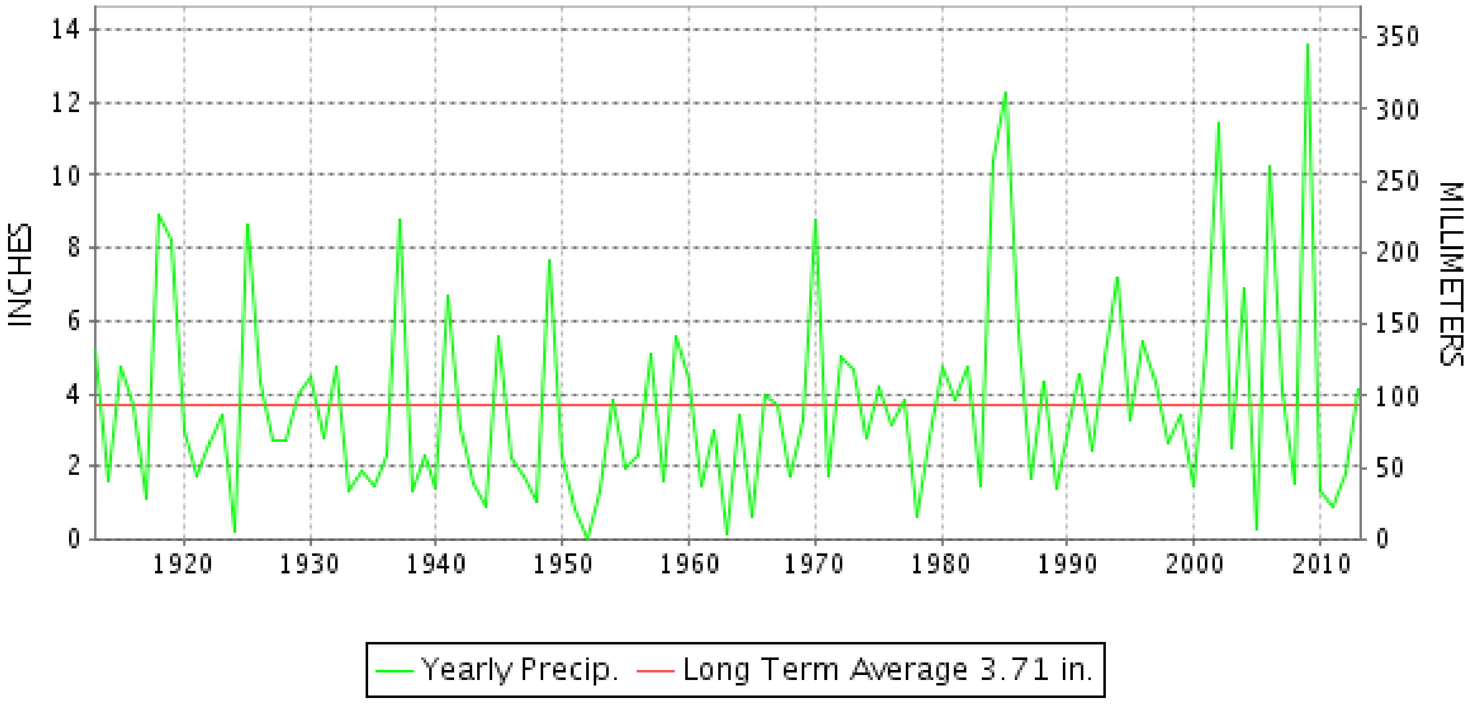

TEMPERATURE AND PRECIPITATION EXTREMES

**LOUISIANA**			
HIGHEST TEMPERATURE	95	OCTOBER 03	LELAND BOWMAN LOCK
LOWEST TEMPERATURE	30	OCTOBER 26	ALEXANDRIA 5 SSE
GREATEST TOTAL PRECIPITATION	9.78		SAILES FIRE TWR
LEAST TOTAL PRECIPITATION	0.65		MOUNT HERMON 2W
GREATEST 1 DAY PRECIPITATION	7.11	OCTOBER 31	ZWOLLE 2 NW

"I certify that this is an official publication of the National Oceanic and Atmospheric Administration (NOAA) It is compiled using information from weather observing sites supervised by NOAA/National Weather Service and received at the National Climatic Data Center(NCDC), Asheville, North Carolina 28801."

Director
National Climatic Data Center

noaa

National
Oceanic and
Atmospheric Administration

National
Environmental Satellite, Data
and Information Service

National
Climatic Data Center
Asheville, North Carolina

MONTHLY STATION AND DIVISION SUMMARY

STATION	TEMPERATURE (°F) AVERAGE MAXIMUM	AVERAGE MINIMUM	AVERAGE	DEPARTURE FROM NORMAL	HIGHEST	DATE	LOWEST	DATE	HEATING DEG. DAYS	COOLING DEG. DAYS	NO. OF DAYS MAX >=90	MAX <=32	MIN <=32	MIN <=0	PRECIPITATION (IN) TOTAL	DEPARTURE FROM NORMAL	GREATEST 24 HOURS	DATE	ICE PELLETS, SNOW TOTAL	MAX DEPTH ON GROUND	DATE	NO. OF DAYS .10 OR MORE	.50 OR MORE	1.00 OR MORE
**LOUISIANA**																								
**NORTHWEST 01**																								
BENTON 5E	74.6	56.2	65.4	0.6	85	05+	42	20	83	104	0	0	0	0	M 7.58	2.55	3.90	31	0.0	0		6	4	3
HOSSTON															8.43		4.06	31	0.0			7	3	3
JAMESTOWN															8.17		2.48	31	0.0			8	5	4
KEITHVILLE															6.93		2.56	31	0.0	0		7	3	3
KORAN															7.88		2.70	15	0.0			4	4	3
LOGANSPORT															M 6.90		2.56	31	0.0	0		4	3	3
MANSFIELD 7 NW	M	M	M								0	0	0	0	M				0.0					
MINDEN	76.6	55.9	66.3	1.8	87	04+	40	21+	77	125	0	0	0	0	4.94	-0.11	2.25	16	0.0	0		6	3	2
MOORINGSPORT 1 N	75.5	56.1	65.8	0.5	86	05	42	20	78	114	0	0	0	0	8.33	3.45	4.63	31	0.0	0		8	3	3
RED RIVER RSCH STN	77.5	57.1	67.3	1.4	87	05+	41	20	54	134	0	0	0	0	9.14	4.38	2.64	13	M 0.0			5	5	3
SHREVEPORT DWTN															MA 3.92		0.99	13	0.0	0		3	1	0
SHREVEPORT DWTN AP	76.5	56.5	66.5	0.5	87	04	41	20	57	111	0	0	0	0	7.32	2.27	2.10	15	0.0			6	5	3
SHREVEPORT AP	77.9	57.5	67.7	1.3	89	04	42	20	38	131	0	0	0	0	7.02	2.06	1.76	31	0.0	0		7	6	3
SHREVEPORT STHRN HILLS	76.5	55.9	66.2	0.9	86	05+	41	21+	75	118	0	0	0	0	7.72	2.58	2.47	13	0.0	0		5	4	3
SHREVEPORT WFO	77.0	59.1	68.1	2.0	88	04+	44	20	30	133	0	0	0	0	7.11	1.81	1.65	31	0.0	0		7	6	4
SPRINGHILL															8.08		3.82	31	0.0			6	4	2
VIVIAN															M				M 0.0	0				
**--DIVISIONAL DATA------->**			66.7	1.8B											7.59	3.19B								
**NORTH CENTRAL 02**																								
ARCADIA															M 7.13		3.30	31	0.0	0		6	4	2
BIENVILLE 3 NE	77.2	56.6	66.9	1.9	87	01	39	20	55	122	0	0	0	0	9.14	3.99	3.27	31	0.0			9	6	3
CALHOUN RSCH STN	77.2M	52.3M	64.8M	1.1	90	05+	37	20	107E	106E	2	0	0	0	M 4.93	0.21	2.46	31	0.0			5	2	2
COLUMBIA LOCK	77.5M	56.2M	66.9M	1.9	89	04	41	27	70E	132E	0	0	0	0	M 2.69	-2.05	1.56	13	0.0	0		5	2	1
FARMERVILLE	74.9	57.1	66.0	0.6	85	06+	43	26+	77	116	0	0	0	0	7.52	2.27	3.00	31	0.0	0		8	4	2
HOMER 1N	74.3M	52.1M	63.2M	-0.1	89	03	35	20	135E	88E	0	0	0	0	M 7.82	2.63	3.46	31	0.0			5	4	3
JONESBORO 4 ENE															M				0.0					
MONROE REGIONAL AP	78.0	55.2	66.6	0.4	90	02	39	20	66	122	1	0	0	0	7.56	2.78	4.38	31	0.0	0		7	3	1
MONROE DELTA CC															4.57	-0.51	1.61	31	0.0	0		6	3	2
RUSTON LA TECH	76.1	54.8	65.4	1.6	86	03	40	20	90	109	0	0	0	0	7.28	2.39	2.80	31	0.0			6	3	3
SAILES FIRE TWR															9.78		3.45	31	0.0	0		7	5	4
WEST MONROE															5.00		2.97	31	0.0			4	2	2
WINNFIELD 3 N	M	M	M								0	0	0	0	M				0.0					
**--DIVISIONAL DATA------->**			65.7	1.1B											7.26	3.02B								
**NORTHEAST 03**																								
BASTROP	74.8M	54.7	64.7M	0.6	86	06+	40	26+	109E	113E	0	0	0	0	6.07	0.87	2.52	13	0.0	0		6	3	2
LAKE PROVIDENCE	76.3	57.0	66.6	0.9	88	06	43	27+	65	124	0	0	0	0	3.61	-1.77	1.70	06	0.0	0		6	2	1

LOUISIANA
201310

MONTHLY STATION AND DIVISION SUMMARY

STATION	TEMPERATURE (°F) AVERAGE MAXIMUM	AVERAGE MINIMUM	AVERAGE	DEPARTURE FROM NORMAL	HIGHEST	DATE	LOWEST	DATE	HEATING DEG. DAYS	COOLING DEG. DAYS	NO. OF DAYS MAX >=90	MAX <=32	MIN <=32	MIN <=0	PRECIPITATION (IN) TOTAL	DEPARTURE FROM NORMAL	GREATEST 24 HOURS	DATE	ICE PELLETS, SNOW TOTAL	MAX DEPTH ON GROUND	DATE	NO. OF DAYS .10 OR MORE	.50 OR MORE	1.00 OR MORE
OAK GROVE															A 7.95		5.74	06	0.0	0		3	3	1
OAK RIDGE															MA 4.82		2.42	06	0.0	0		2	2	2
PIONEER 6 W															6.57		4.20	06	0.0	0		6	3	2
RAYVILLE	78.9	58.1	68.5	2.2	91	06+	43	26+	50	168	5	0	0	0	3.44	-1.80	1.49	06	0.0	0		4	3	2
ST JOSEPH 3 N	81.0M	58.7M	69.8M	2.9	90	06+	39	27	36E	221E	3	0	0	0	M 1.79	-2.18	1.02	06	0.0	0		4	1	1
TALLULAH	78.9M	57.4M	68.1M	2.2	91	04	40	26	49E	155E	1	0	0	0	M 3.55	-1.10	1.43	06	0.0	0		5	2	2
TALLULAH VICKSBURG RGN	78.6	52.7	65.7	0.2	90	05+	35	26+	101	132	2	0	0	0	3.54	-1.13	2.02	31	0.0	0		4	3	1
WINNSBORO 2 SE	80.0	55.9	68.0	1.6	91	03	37	20	67	166	3	0	0	0	1.28	-3.80	0.45	13	0.0	0		3	0	0
WINNSBORO 5 SSE	79.4	53.9	66.7	0.5	90	05+	36	20	89	149	4	0	0	0	M 0.49	-4.17	0.19	06	0.0	0		2	0	0
**--DIVISIONAL DATA------->**			67.3	1.9B											4.64	0.67B								
**WEST CENTRAL 04**																								
GORUM FIRE TWR															M				0.0					
HODGES GARDENS	77.3	58.2	67.7	0.3	87	06+	47	21+	44	136	0	0	0	0	9.53	4.66	6.70	31	0.0			5	2	2
LEESVILLE	78.9	56.1	67.5	1.5	88	13+	37	20	66	153	0	0	0	0	4.19	-0.79	1.72	31	0.0			5	2	2
LEESVILLE 6 SSW															3.30		2.06	31	0.0			5	1	1
MANY 9 WSW															7.71		6.78	31	0.0	0		4	1	1
NATCHITOCHES #2	76.0	56.9	66.5	-0.2	87	06+	42	26+	72	123	0	0	0	0	7.53	2.86	5.55	31	0.0	0		4	2	2
TOLEDO BEND LAKE	78.4	58.6	68.5	0.4	89	04	44	22+	43	159	0	0	0	0	6.00	0.84	4.32	31	0.0	0		6	2	1
ZWOLLE 2 NW															8.82		7.11	31	0.0	0		6	2	1
**--DIVISIONAL DATA------->**			67.6	2.3B											6.73	2.68B								
**CENTRAL 05**																								
ALEXANDRIA	79.2	59.3	69.2	1.2	88	05+	46	27+	39	177	0	0	0	0	2.61	-2.66	1.60	13	M 0.0	0		4	2	1
ALEXANDRIA 5 SSE	76.8	51.1	64.0	-2.4	90	02	30	26	130	106	1	0	2	0	4.23	-1.19	1.97	31	M 0.0	0		6	3	1
BEAVER FIRE TWR															7.68		3.91	13	0.0			5	4	2
BOYCE 3 WNW	75.8	59.5	67.6	-0.1	85	05+	46	27+	46	134	0	0	0	0	7.87	2.95	3.27	31	M 0.0	0		7	5	2
BUNKIE	78.7	57.8	68.3	0.6	89	05+	43	27+	60	171	0	0	0	0	2.08	-3.33	0.95	13	0.0			5	2	0
CLAYTON															M 1.20		1.20	13	0.0	0		1	1	1
EUNICE	80.4	58.8	69.6	-0.2	89	13+	46	26	32	183	0	0	0	0	M 3.88	-1.53	1.47	01	0.0			4	4	2
GRAND COTEAU	80.8	59.7	70.3	1.5	89	12	44	26	16	188	0	0	0	0	4.95	-0.67	1.90	13	M 0.0	0		8	2	2
JENA 4 WSW	M	M	M		91	05	46	09	22E	242E	1	0	0	0	M				0.0					
JONESVILLE LOCKS	80.6M	58.8	69.7M	2.1	89	10+	45	28+	37E	200E	0	0	0	0	1.90	-3.13	0.85	13	0.0	0		4	2	0
LSU DEAN LEE RSCH STN	M	M	M		87	07	41	28+	47E	133E	0	0	0	0	M				0.0					
MARKSVILLE															2.80		0.84	19	0.0			5	3	0
NEW ROADS 5 NE	79.6	60.5	70.1	1.1	90	12+	45	26	20	187	2	0	0	0	4.06	-0.58	2.00	31	0.0			5	3	1
OPELOUSAS															2.51		1.15	13	0.0			5	2	1
PORT ALLEN															3.90		1.22	19	0.0			7	3	1
RED RIVER LOCK #1															2.30		1.00	28	0.0	0		4	2	1
RED RIVER LOCK # 2															2.09		0.50	28+	0.0			7	2	0

LOUISIANA
201310

MONTHLY STATION AND DIVISION SUMMARY

STATION	TEMPERATURE (°F)														PRECIPITATION (IN)									
											NO. OF DAYS								ICE PELLETS, SNOW			NO. OF DAYS		
											MAX		MIN											
	AVERAGE MAXIMUM	AVERAGE MINIMUM	AVERAGE	DEPARTURE FROM NORMAL	HIGHEST	DATE	LOWEST	DATE	HEATING DEG. DAYS	COOLING DEG. DAYS	>=90	<=32	<=32	<=0	TOTAL	DEPARTURE FROM NORMAL	GREATEST 24 HOURS	DATE	TOTAL	MAX DEPTH ON GROUND	DATE	.10 OR MORE	.50 OR MORE	1.00 OR MORE
VILLE PLATTE															1.73		0.90	13	0.0			4	2	0
**--DIVISIONAL DATA------->**			68.6	1.6B											3.62	-0.76B								
**EAST CENTRAL 06**																								
ABITA RVR COVINGTON															M 1.71		0.56	19	0.0			4	1	0
ABITA SPRINGS 1 SW															2.24		1.34	01	M 0.0			4	1	1
ABITA SPRING FIRE TWR															1.38		0.70	19	0.0			2	2	0
ANGIE															M				0.0					
BAKER															MA 3.27		0.02	03	0.0			0	0	0
BATON ROUGE CONCORD															3.08		0.97	19	M 0.0			6	2	0
BATON ROUGE METRO AP	80.4	59.8	70.1	0.8	89	13+	44	26	19	185	0	0	0	0	3.87	-0.83	1.26	31	0.0	0		5	3	2
BATON ROUGE SHERWOOD															2.42		1.02	19	0.0			5	1	1
BOGALUSA	M	M	M								0	0	0	0	M				0.0					
CLINTON FORESTRY HQ															1.44		0.64	17	0.0			3	2	0
CLINTON 5 SE	78.0	57.3	67.7	1.3	86	14+	42	28+	53	145	0	0	0	0	1.45	-3.43	0.73	19	0.0			4	1	0
COVINGTON 3 NE															M				0.0					
DENHAM SPRINGS															2.06		0.90	19	M 0.0	0		9	1	0
HAMMOND 5 E	79.4	57.1	68.2	0.3	88	14+	38	26	51	160	0	0	0	0	1.26	-2.94	1.01	19	0.0			3	1	1
KILLIAN															2.04		0.91	19	M 0.0	0		5	2	0
LIVERPOOL 6W															M				0.0					
LIVINGSTON															1.13		0.50	06	0.0			3	1	0
LSU BEN-HUR FARM	80.0	59.8	69.9	1.3	88	13+	45	26	23	185	0	0	0	0	2.05	-2.30	1.01	19	0.0			4	1	1
MOUNT HERMON 2W															0.65		0.49	20	0.0			2	0	0
NORWOOD															2.48		1.24	13	0.0			5	1	1
OAKNOLIA 2N															1.28		0.73	19	0.0			3	1	0
PINE GROVE FIRE TWR															1.23		0.92	19	0.0			2	1	0
PONCHATOULA 4 SE															M				0.0					
ST FRANCISVILLE															2.77		1.02	17+	0.0			4	2	2
SLIDELL	79.7M	61.5M	70.6M	1.5	87	05+	44	26	23E	203E	0	0	0	0	M 1.39	-2.43	0.56	19	0.0			4	1	0
SLIDELL AP	81.1	59.6	70.3	2.0	89	04	40	26	26	201	0	0	0	0	1.43	-2.14	0.75	19	0.0			2	1	0
SUN															1.14		0.57	20	0.0			2	1	0
TALISHEEK															1.00		0.69	19	0.0			1	1	0
TICKFAW 3 ENE															M				0.0					
**--DIVISIONAL DATA------->**			69.5	2.2B											1.82	-1.78B								
**SOUTHWEST 07**																								
ABBEVILLE															3.72		1.50	13	0.0			6	2	2
BELL CITY 13 SW															M 3.88		1.93	17	0.0			3	3	1
CROWLEY 2 NE	79.7	60.3	70.0	0.4	89	04	46	26	22	183	0	0	0	0	2.27	-3.04	0.74	13	0.0			4	2	0
DE RIDDER	78.0	59.0	68.5	0.6	89	13	43	20	44	161	0	0	0	0	5.25	0.09	2.14	31	0.0			8	3	2

MONTHLY STATION AND DIVISION SUMMARY

STATION	TEMPERATURE (°F) AVERAGE MAXIMUM	AVERAGE MINIMUM	AVERAGE	DEPARTURE FROM NORMAL	HIGHEST	DATE	LOWEST	DATE	HEATING DEG. DAYS	COOLING DEG. DAYS	NO. OF DAYS MAX >=90	MAX <=32	MIN <=32	MIN <=0	PRECIPITATION (IN) TOTAL	DEPARTURE FROM NORMAL	GREATEST 24 HOURS	DATE	ICE PELLETS, SNOW TOTAL	MAX DEPTH ON GROUND	DATE	NO. OF DAYS .10 OR MORE	.50 OR MORE	1.00 OR MORE
DRY CREEK 8NW															M				0.0					
HACKBERRY 8 SSW	79.7	64.9	72.3	0.6	88	06+	53	21	9	243	0	0	0	0	2.35	-2.63	0.90	13	0.0			4	2	0
JENNINGS	80.5	60.3	70.4	0.8	89	13	47	27+	21	193	0	0	0	0	2.14	-3.15	0.77	13	0.0			6	1	0
KAPLAN															4.24		1.92	31	0.0			4	3	2
LAKE ARTHUR 10 SW	81.9	61.8	71.9	0.9	90	16+	50	20+	5	227	3	0	0	0	3.18	-1.87	1.20	19	0.0			5	2	1
LAKE CHARLES 7 NW															M 3.35		1.22	19	0.0			6	3	1
LAKE CHARLES 2 N															2.72		0.75	19	0.0			5	3	0
LAKE CHARLES PORT															4.22		1.05	14	0.0			7	4	2
LAKE CHARLES AP	80.5	60.6	70.6	0.5	89	12+	47	26	18	198	0	0	0	0	3.54	-1.36	2.10	31	0.0	0		5	2	1
LELAND BOWMAN LOCK	82.1	63.0	72.5	2.5	95	03	50	27+	6	249	2	0	0	0	3.88	-1.20	1.30	13	M 0.0	0		6	3	2
MOSS BLUFF															2.58		0.85	01	0.0			4	3	0
MOSS BLUFF 2 NNW	80.4	57.9	69.1	0.5	90	05+	42	26+	38	171	3	0	0	0	4.92	-0.63	3.36	31	0.0			4	2	1
OAKDALE															M				0.0					
OBERLIN FIRE TWR	78.3	58.0	68.1	-0.2	87	13+	44	26	53	158	0	0	0	0	2.28	-3.17	1.14	13	0.0			2	2	1
OLD TOWN BAY															M 3.36		1.41	13	0.0			6	2	1
ROCKEFELLER WL REFUGE	80.1	61.8	71.0	1.2	88	05+	49	27+	13	204	0	0	0	0	3.69	-1.22	1.03	20	0.0			5	3	1
SULPHUR															4.43		2.90	31	0.0			6	2	1
VINTON 5W	81.1M	58.3M	69.7M		91	13+	44	21+	31E	185E	2	0	0	0	M 2.90		0.66	13	0.0			7	3	0
**--DIVISIONAL DATA------->**			70.4	1.9B											3.46	-0.88B								
**SOUTH CENTRAL 08**																								
BAYOU SORREL LOCK															1.79		0.97	19	0.0			4	1	0
CARENCRO															4.96		2.06	13	0.0			5	3	2
CARVILLE 2 SW	78.6	61.5	70.1	0.7	87	12	45	31	16	184	0	0	0	0	3.26	-1.84	1.31	31	0.0			5	3	1
DONALDSONVILLE 4 SW	79.2	59.4	69.3	0.5	88	13+	46	27+	28	169	0	0	0	0	2.83	-2.09	1.24	19	M 0.0			6	1	1
FRANKLIN 3 NW	78.8	61.6	70.2	0.8	87	12	48	26	12	180	0	0	0	0	3.16	-1.09	0.91	31	0.0			8	2	0
JEANERETTE 5 NW	79.8	60.0	69.9	0.4	87	04	47	27+	20	179	0	0	0	0	4.97	0.07	2.25	13	0.0			6	3	2
LAFAYETTE	80.5	60.0	70.2	1.4	89	04	47	27+	19	189	0	0	0	0	4.86	-0.81	2.05	19	0.0			5	3	2
LAFAYETTE FCWOS	81.1	61.0	71.1	0.8	89	03	47	26	11	208	0	0	0	0	5.68	0.45	1.91	12	0.0	0		6	4	3
MORGAN CITY	80.9	61.6	71.3	0.3	90	04	45	25	12	213	1	0	0	0	1.94	-2.13	0.75	20	0.0			6	1	0
NAPOLEONVILLE															3.05		1.45	19	0.0			5	3	1
NEW IBERIA AP ACADIANA RGNL	83.1	62.4	72.7	2.6	91	03+	48	26	6	254	3	0	0	0	4.75	-0.21	1.35	31	0.0			7	3	3
PLAQUEMINE 2 N															2.22		0.90	19	0.0			4	2	0
ST GABRIEL															1.72		1.13	19	0.0			3	1	1
ST MARTINVILLE 3 SW	79.2	60.1	69.6	0.8	87	04	44	26	29	179	0	0	0	0	4.35	-1.25	1.62	19	0.0			5	3	2
**--DIVISIONAL DATA------->**			70.5	1.5B											3.54	-0.39B								
**SOUTHEAST 09**																								
BOOTHVILLE ASOS	79.5	69.8	74.6	1.7	86	03	61	25	0	306	0	0	0	0	3.24	-1.09	1.04	19	0.0			6	4	1
CONVENT 2S															4.18		1.65	01	0.0			7	2	1

LOUISIANA
201310

MONTHLY STATION AND DIVISION SUMMARY

STATION	TEMPERATURE (°F) AVERAGE MAXIMUM	AVERAGE MINIMUM	AVERAGE	DEPARTURE FROM NORMAL	HIGHEST	DATE	LOWEST	DATE	HEATING DEG. DAYS	COOLING DEG. DAYS	NO. OF DAYS MAX >=90	MAX <=32	MIN <=32	MIN <=0	PRECIPITATION (IN) TOTAL	DEPARTURE FROM NORMAL	GREATEST 24 HOURS	DATE	ICE PELLETS, SNOW TOTAL	MAX DEPTH ON GROUND	DATE	NO. OF DAYS .10 OR MORE	.50 OR MORE	1.00 OR MORE
DUTCHTOWN #2															M 1.93		0.81	19	0.0			5	1	0
GALLIANO	79.6M	64.4M	72.0M	1.2	86	07	51	25+	6E	230E	0	0	0	0	M 3.64	-1.03	1.04	19	0.0			7	2	2
GONZALES															1.98		1.10	19	0.0			4	1	1
GRAND ISLE															3.02		1.20	20	0.0			7	2	1
HOUMA	M	M	M								0	0	0	0	M				0.0					
LUTCHER															4.13		1.64	01	0.0			7	3	1
MARRERO 9 SSW	M	M	M		88	18	51	25+	6E	149E	0	0	0	0	M				0.0					
NEW ORLEANS AP	80.6	65.4	73.0	1.7	90	03	51	26	5	261	1	0	0	0	2.29	-1.25	1.24	19	0.0	0		5	1	1
NEW ORLEANS AUDUBON	81.9M	65.7M	73.8M	1.6	90	12+	52	26	1E	281E	2	0	0	0	M 2.26	-1.53	1.20	19	0.0			5	1	1
NEW ORLEANS ALGIERS															M				0.0					
NEW ORLEANS LKFRNT AP	80.6	68.7	74.6	2.2	89	05	57	27+	0	304	0	0	0	0	1.83	-1.27	0.83	19	0.0			5	1	0
TERRYTOWN 3S	81.4	64.5	72.9	1.4	89	07	51	26	8	260	0	0	0	0	1.96	-1.54	0.55	06	M 0.0			5	1	0
THIBODAUX 4 SE	78.5	59.6	69.1	-0.6	87	04	44	26	30	165	0	0	0	0	4.21	-0.74	1.33	19	0.0			6	4	1
**--DIVISIONAL DATA------->**			72.9	2.9B											2.98	-0.37B								

DAILY PRECIPITATION (INCHES)

STATION	TOTAL	DAY OF MONTH																														
		01	02	03	04	05	06	07	08	09	10	11	12	13	14	15	16	17	18	19	20	21	22	23	24	25	26	27	28	29	30	31
**LOUISIANA**																																
**NORTHWEST 01**																																
BENTON 5E	M 7.58	T	1.21		-	-	-	0.41						0.61			1.27	0.12		T			0.06									3.90
HOSSTON	8.43		0.32				1.41							0.34			1.90	0.30					0.10									4.06
JAMESTOWN	8.17	0.11	1.02	0.01		0.22	0.50							1.66		T	1.99	0.18														2.48
KEITHVILLE	6.93		0.34				0.27							2.03		0.03	1.45	0.13		0.02								0.10				2.56
KORAN	7.88					1.57								0.94		2.70							0.02									2.65
LOGANSPORT	M 6.90	0.02					0.48							1.33		0.01	2.31	0.07					0.08					-	0.02		0.02	2.56
MANSFIELD 7 NW	M	-	-	-	-	-	-	-	-	-	-	-	-	-	-	-	-	-	-	-	-	-	-	-	-	-	-	-	-	-	-	-
MINDEN	4.94		0.10			0.02	0.10							0.79			2.25	0.21														1.47
MOORINGSPORT 1 N	8.33		0.20				1.30							0.10		0.10	1.66	0.15					0.05					0.14				4.63
RED RIVER RSCH STN	9.14	0.04	0.72			0.03	0.84							2.64		0.01	2.36	0.09		0.02								0.01				2.38
SHREVEPORT DWTN	MA 3.92	0.02	0.29				0.48							0.99		0.01	*	2.10a		T			T				-	0.03				-
SHREVEPORT DWTN AP	7.32	0.06			T	0.42							0.58		0.01	2.10	0.76						T				T	0.02			1.41	1.96
SHREVEPORT AP	7.02	0.22	0.09	0.03		1.10							0.76	T	0.02	0.82	0.86	T	T				T				0.02	0.02			1.32	1.76
SHREVEPORT STHRN HILLS	7.72		T				0.84							2.47		T	1.79	0.15		T			T					0.07				2.40
SHREVEPORT WFO	7.11	0.23	0.03	0.02		1.43							0.54	T	0.02	0.74	1.01	0.01	T				T				0.03	0.02			1.38	1.65
SPRINGHILL	8.08	0.02	0.02			0.67	0.41						0.61	0.07	0.02	0.02	2.12	0.07		0.03			0.20									3.82
VIVIAN	M	-	-	-	-	-	-	-	-	-	-	-	-	-	-	-	3.25	-	-	-	-	-	-	-	-	-	-	-	-	-	-	-
**NORTH CENTRAL 02**																																
ARCADIA	M 7.13	0.13	0.01			0.58								0.78		-	2.13	0.19		0.01												3.30
BIENVILLE 3 NE	9.14	0.12			0.85	0.45	0.40						1.55			0.70	1.15														0.65	3.27
CALHOUN RSCH STN	M 4.93	-	0.08				0.24			-				1.47			0.38	0.30														2.46
COLUMBIA LOCK	M 2.69	-		0.12			0.11				-	-		1.56	-			0.28														0.62
FARMERVILLE	7.52	0.10	0.17			0.18	2.32							0.46		0.01	0.74	0.52		0.02												3.00
HOMER 1N	M 7.82	0.13	0.01	0.09		0.07	1.20							0.58	T		2.20	0.07					0.01					T		-		3.46
JONESBORO 4 ENE	M	-	-	-	-	-	-	-	-	-	-	-	-	-	-	-	-	-	-	-	-	-	-	-	-	-	-	-	-	-	-	-
MONROE REGIONAL AP	7.56	0.11	0.02			0.43	0.90						0.99			T	0.41											0.03			0.29	4.38
MONROE DELTA CC	4.57	T	0.13	0.04			1.57							0.77		T	0.22	0.20		T									0.03			1.61
RUSTON LA TECH	7.28	0.15	0.02			0.37	0.44							1.44				2.01					0.05									2.80
SAILES FIRE TWR	9.78	0.03	0.83	0.10		0.47		1.00						1.60			2.25	0.05														3.45
WEST MONROE	5.00		0.02			0.04	0.32	0.01						1.27	0.03		0.33												0.01			2.97
WINNFIELD 3 N	M	-	-	-	-	-	-	-	-	-	-	-	-	-	-	-	-	-	-	-	-	-	-	-	-	-	-	-	-	-	-	-
**NORTHEAST 03**																																
BASTROP	6.07	0.06	T	0.10		T	0.94							2.52			0.32	0.21											T			1.92
LAKE PROVIDENCE	3.61	0.11					1.70							0.95			0.20												0.25			0.40
OAK GROVE	A 7.95	0.08					5.74							0.98			*	0.29a											0.05			0.81
OAK RIDGE	MA 4.82		-				2.42							2.00			*	0.40a											T			-
PIONEER 6 W	6.57						4.20							0.50			0.27	0.10											0.10			1.40
RAYVILLE	3.44						1.49							1.02			0.03	0.14											0.02			0.74
ST JOSEPH 3 N	M 1.79						1.02							0.40				0.08		0.11									0.18			-
TALLULAH	M 3.55	-					1.43							1.25				0.29											0.13			0.45

LOUISIANA
201310

DAILY PRECIPITATION (INCHES)

STATION	TOTAL	01	02	03	04	05	06	07	08	09	10	11	12	13	14	15	16	17	18	19	20	21	22	23	24	25	26	27	28	29	30	31
TALLULAH VICKSBURG RGN	3.54		T	0.01			0.51						0.66				0.29	T		0.03								0.01	T		0.01	2.02
WINNSBORO 2 SE	1.28						0.44							0.45																		0.39
WINNSBORO 5 SSE	M 0.49	0.02	0.06				0.19							-				0.04		0.18												-
**WEST CENTRAL 04**																																
GORUM FIRE TWR	M	-	-	-	-	-	-	-	-	-	-	-	-	-	-	-	-	-	-	-	-	-	-	-	-	-	-	-	-	-	-	-
HODGES GARDENS	9.53	0.06		0.09		0.19	0.45							1.90	0.02			0.10										0.02				6.70
LEESVILLE	4.19	0.36	0.01	0.05		0.17	0.05							1.49			0.01	0.03	0.01	0.29									T			1.72
LEESVILLE 6 SSW	3.30	0.40		0.04				0.19							0.19			0.04				0.33							0.05			2.06
MANY 9 WSW	7.71	0.05				0.10	0.06							0.47			0.10	0.07	0.02				0.02					0.02			0.02	6.78
NATCHITOCHES #2	7.53	T	T	0.05		0.10	1.28							0.46			0.01	0.06		0.01								0.01	T			5.55
TOLEDO BEND LAKE	6.00	0.47				0.26	0.70							0.12						0.12			0.01									4.32
ZWOLLE 2 NW	8.82		0.07	0.18			0.43							0.66			0.18	0.19														7.11
**CENTRAL 05**																																
ALEXANDRIA	2.61			0.03		0.08	0.10							1.60						0.10												0.70
ALEXANDRIA 5 SSE	4.23				0.30		0.25						0.79						0.01	0.02								0.63			0.26	1.97
BEAVER FIRE TWR	7.68	0.03	0.70			0.05	0.89							3.91				0.07		0.47												1.56
BOYCE 3 WNW	7.87	0.26		0.04	0.72		0.67						1.80				0.09	0.02			0.22										0.78	3.27
BUNKIE	2.08	0.06	0.12		0.07		0.17							0.95				0.13	0.08	0.50												
CLAYTON	M 1.20						-							1.20				-		-									-			-
EUNICE	M 3.88	1.47	0.03				0.58						-	1.18						0.58									0.04		-	
GRAND COTEAU	4.95	0.10	0.02		0.13	0.45		0.10						1.90		0.02	0.10	0.02	0.02	0.45								0.04				1.60
JENA 4 WSW	M		0.22	T			T				-		-	-	-	-	-	-	-	-	-	-	-	-	-	-	-	-	-	-	-	-
JONESVILLE LOCKS	1.90						0.21	0.04						0.85				0.07		0.10									0.60			0.03
LSU DEAN LEE RSCH STN	M	0.15	0.04		-	-	-	0.39	-			-	-	-	0.44			0.02	-	-	-	-	0.15				-	-	1.38			-
MARKSVILLE	2.80	0.50		0.36										0.75						0.84									0.33			0.02
NEW ROADS 5 NE	4.06			0.10	0.01	0.20							0.72				0.95		0.02	0.04									0.02			2.00
OPELOUSAS	2.51		0.60	0.10			0.33							1.15						0.30			0.03									
PORT ALLEN	3.90	0.28	0.84	T		0.17	0.21							0.85				0.25		1.22	0.05		0.03						T			
RED RIVER LOCK #1	2.30						0.08							0.80				0.15		0.25									1.00			0.02
RED RIVER LOCK # 2	2.09	0.10		0.09		0.15	0.38							0.50				0.02		0.18									0.50			0.17
VILLE PLATTE	1.73	0.03	0.03		0.10		0.10							0.90				0.03		0.54												
**EAST CENTRAL 06**																																
ABITA RVR COVINGTON	M 1.71	0.25	0.02	0.47		0.05	0.05	-									-			0.56	0.24		0.05						0.02			
ABITA SPRINGS 1 SW	2.24	1.34	0.02	0.10	T		0.01	0.01										T		0.33	0.39		0.03						0.01			
ABITA SPRING FIRE TWR	1.38	0.51		0.08			0.04													0.70			0.05									
ANGIE	M	-	-	-	-	-	-	-	-	-	-	-	-	-	-	-	-	-	-	-	-	-	-	-	-	-	-	-	-	-	-	-
BAKER	MA 3.27	*	1.58_{a}	0.02		*	*	0.00_{a}					*	*	0.85_{a}					*	*	0.82_{a}					-	-				
BATON ROUGE CONCORD	3.08	0.22	0.81			0.40	0.34							0.25				0.05		0.97	0.02		0.01						0.01			
BATON ROUGE METRO AP	3.87	0.52	T	0.02	T	0.45	T						0.33				0.03	T	0.02	1.24								T	T			1.26
BATON ROUGE SHERWOOD	2.42		0.40			0.35	0.33							0.23				0.08		1.02	T		0.01									
BOGALUSA	M	-	-	-	-	-	-	-	-	-	-	-	-	-	-	-	-	-	-	-	-	-	-	-	-	-	-	-	-	-	-	-
CLINTON FORESTRY HQ	1.44					0.30												0.64		0.50												

LOUISIANA
201310

DAILY PRECIPITATION (INCHES)

STATION	TOTAL	01	02	03	04	05	06	07	08	09	10	11	12	13	14	15	16	17	18	19	20	21	22	23	24	25	26	27	28	29	30	31
CLINTON 5 SE	1.45	0.03						0.20							0.23	0.02		0.15		0.73			0.08						0.01			
COVINGTON 3 NE	M	-	-	-	-	-	-	-	-	-	-	-	-	-	-	-	-	-	-	-	-	-	-	-	-	-	-	-	-	-	-	-
DENHAM SPRINGS	2.06	0.14	0.10	0.10		0.10	0.20							0.24	0.02			0.11		0.90	0.05									0.10		
HAMMOND 5 E	1.26	0.11	0.13	T			T													1.01	0.01											
KILLIAN	2.04	0.12			0.20		0.50						0.01							0.91	0.23	0.02							0.05			
LIVERPOOL 6W	M	-	-	-	-	-	-	-	-	-	-	-	-	-	-	-	-	-	-	-	-	-	-	-	-	-	-	-	-	-	-	-
LIVINGSTON	1.13						0.50							0.01						0.41	0.21											
LSU BEN-HUR FARM	2.05	0.38	0.02			0.27	0.02						0.01	0.23				0.07		1.01	0.01		0.01						0.02			
MOUNT HERMON 2W	0.65			0.16																	0.49											
NORWOOD	2.48		0.41				0.27							1.24				0.17		0.39												
OAKNOLIA 2N	1.28	0.04				0.04	0.16							0.27				0.04		0.73												
PINE GROVE FIRE TWR	1.23					0.31												T		0.92												
PONCHATOULA 4 SE	M	0.05		T		0.03	0.06	-	-	-	-	-	-	-	-	-	-	-	-	-	-	-	-	-	-	-	-	-	-	-	-	-
ST FRANCISVILLE	2.77	0.06	0.01	T		0.05	0.22							1.02				1.02	0.03	0.36												
SLIDELL	M 1.39	0.01	0.02	0.06		0.21	0.01	0.23										0.02	T	0.56	0.27		T					-	T			
SLIDELL AP	1.43	0.01	0.04	T	0.01	0.01	0.01							0.08			T	0.01	0.02	0.75		0.02	T					0.04				0.43
SUN	1.14	0.06	0.25	0.06		0.04	0.05													0.08	0.57		0.03									
TALISHEEK	1.00	0.08	0.03	0.04	0.01	0.09	0.02	T												0.69	0.03								0.01			
TICKFAW 3 ENE	M	-	-	-	-	-	-	-	-	-	-	-	-	-	-	-	-	-	-	-	-	-	-	-	-	-	-	-	-	-	-	-
**SOUTHWEST 07**																																
ABBEVILLE	3.72	0.23	T			T	0.29							1.50	0.01		0.17	0.18		1.34	T								T			
BELL CITY 13 SW	M 3.88																	1.93					0.93	-	-	-	-	-	0.05	0.96	0.01	
CROWLEY 2 NE	2.27	0.23	0.06				0.47							0.74			0.05			0.61			0.02						0.05	0.04		
DE RIDDER	5.25	0.12	1.31		0.02	0.27	0.23	0.02						0.62	T			T		0.42			T					T	T	0.10		2.14
DRY CREEK 8NW	M	0.56	0.11	-	-	-	-	-	-	-	-	-	-	0.62	-	-	-	0.14	-	0.49	-	-	-	-	-	-	-	-	-	-	-	0.63
HACKBERRY 8 SSW	2.35	0.27				0.01								0.90	0.06	0.01		0.10		0.88			0.07						0.05			
JENNINGS	2.14	0.21	0.02		T	0.03	0.20	T						0.77						0.41	0.02	0.01	0.02						0.31	0.14		
KAPLAN	4.24	0.28				0.04							0.70							1.26									0.04			1.92
LAKE ARTHUR 10 SW	3.18	0.38	0.23					0.47						0.70				0.01	0.01	1.20	0.09		0.02	0.01					0.03	0.02	0.01	
LAKE CHARLES 7 NW	M 3.35	0.50	0.80		-	0.30								0.32				0.02		1.22			0.02					T	0.10	0.05		0.02
LAKE CHARLES 2 N	2.72	0.40	0.57			0.26								0.58				0.01		0.75			0.03						0.09	0.03		
LAKE CHARLES PORT	4.22	0.39	0.60	0.13	0.01	0.05	0.01			0.01					1.05	0.02		0.03	0.01	0.62	0.01		0.04		0.01				1.00	0.23		
LAKE CHARLES AP	3.54	T	0.01	T	0.25		T						0.26	T			0.01	T	0.01	0.74		0.02						0.14	T		T	2.10
LELAND BOWMAN LOCK	3.88	1.00					0.10							1.30			0.05	0.13		0.95									0.05	0.30		
MOSS BLUFF	2.58	0.85	0.53			0.04								0.40				T		0.63			0.03						0.09	0.01		T
MOSS BLUFF 2 NNW	4.92	0.25			0.07								0.21	0.01					0.01	0.87		0.01						0.09	0.04		T	3.36
OAKDALE	M	-	-	-	-	-	0.27	-	-	-	-	-	-	0.87	-	-	-	-	-	-	0.57	-	-	-	-	-	-	-	-	-	-	-
OBERLIN FIRE TWR	2.28	0.01	0.09				0.05							1.14				0.01		0.89												0.09
OLD TOWN BAY	M 3.36	0.30	0.30	0.05	0.05	0.30								1.41	T		-	0.03	0.03	0.73	T		0.04						0.12	-		-
ROCKEFELLER WL REFUGE	3.69	0.45	0.01		0.08	T	0.74							0.45	0.85			0.01			1.03		0.03						0.04			
SULPHUR	4.43	0.30	0.02			0.05							0.30						0.01	0.50		0.02						0.10	0.20		0.03	2.90
VINTON 5W	M 2.90	0.64		-	0.09	0.11	0.10	-			-			0.66						0.56			-						0.41			0.33

LOUISIANA
201310

DAILY PRECIPITATION (INCHES)

STATION	TOTAL	DAY OF MONTH 01	02	03	04	05	06	07	08	09	10	11	12	13	14	15	16	17	18	19	20	21	22	23	24	25	26	27	28	29	30	31
**SOUTH CENTRAL 08**																																
BAYOU SORREL LOCK	1.79	0.02				0.17	0.20							0.30				0.08		0.97			0.02						0.03			
CARENCRO	4.96	1.45	0.39				0.46							2.06			0.03			0.57												
CARVILLE 2 SW	3.26		0.10		0.87	0.09	0.01										0.02		0.10	0.70								0.04	0.02			1.31
DONALDSONVILLE 4 SW	2.83	0.18	0.03	0.24		0.36	0.38	0.04						0.04				0.02	T	1.24	0.28		0.01						0.01			
FRANKLIN 3 NW	3.16	0.01		0.19		0.33								0.27			0.28	0.09	0.14	0.84		0.10										0.91
JEANERETTE 5 NW	4.97		0.13		0.18	0.03	0.79						0.02	2.25	0.05			0.37		1.10	0.02								0.03			
LAFAYETTE	4.86	0.05			0.12		0.54							1.95						2.05	0.05								0.10			
LAFAYETTE FCWOS	5.68	0.13		T	0.03	0.28	T						1.91				0.03		0.02	1.14								0.01	0.50			1.63
MORGAN CITY	1.94	0.43	0.04		0.02	0.12	0.11	0.02						0.11				0.04		0.26	0.75		0.04									
NAPOLEONVILLE	3.05	0.56	0.02			0.52	0.23											0.25		1.45			0.02									
NEW IBERIA AP ACADIANA RGNL	4.75	0.04		0.17	0.14	0.22	T						1.27	0.04			0.37		0.01	1.14		T						T				1.35
PLAQUEMINE 2 N	2.22	0.74	T	0.20		T	0.01							0.30				0.04		0.90	0.03		T						T			
ST GABRIEL	1.72	0.04	T	T		0.17	0.14							0.08				0.05		1.13	0.06		0.03						0.02			
ST MARTINVILLE 3 SW	4.35					0.08	0.83							1.30			0.30	0.21		1.62									0.01			
**SOUTHEAST 09**																																
BOOTHVILLE ASOS	3.24	0.04	0.60	0.73	0.07	0.11	0.51											T	T	1.04		T	T									0.14
CONVENT 2S	4.18	1.65	0.05	0.09			0.33						0.21	0.48						0.99	0.28		0.10						T			
DUTCHTOWN #2	M 1.93	0.26	0.05	0.03		0.15	0.27							0.03						0.81	0.30								0.03		-	
GALLIANO	M 3.64	1.00	0.20	0.40	0.03	0.29	0.05	0.01				0.10						0.03		1.04	0.47		0.02				-	-				
GONZALES	1.98	0.09		0.11		0.24	0.33										0.03			1.10	0.06								0.02			
GRAND ISLE	3.02	0.30	0.60		0.20	0.10	0.40									0.02				0.20	1.20											
HOUMA	M	-	-	-	-	-	-	-	-	-	-	-	-	-	-	-	-	-	-	-	-	-	-	-	-	-	-	-	-	-	-	-
LUTCHER	4.13	1.64	0.54	0.07		0.12	0.36						0.30	0.03						0.70	0.30		0.05						0.02			
MARRERO 9 SSW	M	0.04	-	-	-	-	-	-	-	-	-	-	-	-	-	-	-	-	-	-	-	-	0.03	T			-	-	-			
NEW ORLEANS AP	2.29	T	0.20	T	0.24	0.19						0.08					T		0.01	1.24		0.01						T				0.32
NEW ORLEANS AUDUBON	M 2.26	0.03	0.10		0.47	0.21	0.02					0.10	0.09			0.03				1.20		0.01										-
NEW ORLEANS ALGIERS	M	-	-	-	-	-	-	-	-	-	-	-	-	-	-	-	-	-	-	-	-	-	-	-	-	-	-	-	-	-	-	-
NEW ORLEANS LKFRNT AP	1.83	0.03	0.13	T	0.12	0.32	0.02	T				0.08					0.01		0.01	0.83		T						T				0.28
TERRYTOWN 3S	1.96	0.08	0.04	0.10	0.01	0.08	0.55	0.02					0.17	0.09						0.36	0.46		T									
THIBODAUX 4 SE	4.21	0.42	0.56	0.17		0.60	0.09						0.90	0.01				T		1.33	0.08		0.05									

LOUISIANA
201310

DAILY TEMPERATURES (°F)

STATION	OB.TIME	MAX/MIN	01	02	03	04	05	06	07	08	09	10	11	12	13	14	15	16	17	18	19	20	21	22	23	24	25	26	27	28	29	30	31	AVERAGE
**LOUISIANA**																																		
**NORTHWEST 01**																																		
BENTON 5E	08	MAX	80	83	85	85	85	82	73	76	74	77	80	82	79	75	75	79	64	69	70	63	68	69	68	72	75	70	67	67	75	76	69	74.6
		MIN	70	70	70	70	72	55	50	51	49	49	54	62	62	65	64	62	56	48	50	42	43	49	48	48	44	44	56	56	57	60	66	56.2
MANSFIELD 7 NW	08	MAX																																M
		MIN																																M
MINDEN	07	MAX	82	87	86	87	84	83	75	77	75	78	81	84	85	77	79	83	65	71	73	65	69	75	70	74	77	62	73	73	76	79	71	76.6
		MIN	71	71	70	70	70	56	49	49	49	49	52	57	68	67	67	64	59	50	50	40	40	49	47	47	47	42	42	56	58	62	65	55.9
MOORINGSPORT 1 N	08	MAX	80	83	85	85	86	84	74	77	78	76	80	82	83	78	76	82	64	70	71	64	68	71	70	73	76	62	73	69	75	77	69	75.5
		MIN	71	70	70	70	72	55	52	50	49	50	53	59	66	65	65	62	54	49	53	42	45	51	49	48	45	44	45	54	56	62	64	56.1
RED RIVER RSCH STN	07	MAX	83	87	87	86	87	85	76	79	76	79	81	84	86	77	79	83	67	72	72	66	68	76	71	76	79	64	75	72	78	81	72	77.5
		MIN	72	71	71	70	71	57	51	51	50	52	53	58	66	67	67	64	58	51	51	41	49	51	47	49	46	43	50	54	57	66	66	57.1
SHREVEPORT DWTN AP	24	MAX	86	86	86	87	83	75	77	76	79	80	83	82	76	78	82	65	72	72	65	68	74	71	76	78	64	75	71	77	81	70	75	76.5
		MIN	71	72	71	70	61	57	51	51	50	52	56	67	66	66	64	60	53	49	46	41	47	51	47	55	46	43	56	53	59	66	54	56.5
SHREVEPORT AP	24	MAX	86	87	88	89	86	77	79	77	80	83	85	84	77	79	85	66	72	73	67	71	76	72	77	80	66	77	72	79	81	70	75	77.9
		MIN	71	71	71	69	61	56	52	52	51	53	58	67	67	68	65	61	53	51	47	42	50	53	50	52	47	46	57	57	62	66	55	57.5
SHREVEPORT STHRN HILLS	07	MAX	83	84	86	86	86	85	75	78	76	78	81	83	84	76	78	82	65	77	71	65	68	74	69	73	76	62	73	70	76	79	71	76.5
		MIN	70	70	70	69	68	57	50	50	50	50	52	57	66	67	67	64	57	50	51	41	41	49	47	47	48	43	44	56	57	61	65	55.9
SHREVEPORT WFO	24	MAX	84	86	88	88	86	75	77	74	78	81	84	82	77	79	84	66	70	72	64	70	75	70	75	77	77	75	70	77	81	71	75	77.0
		MIN	71	71	71	71	61	57	53	55	55	55	60	68	68	68	65	61	56	53	50	44	51	58	52	55	55	46	58	58	62	67	57	59.1
**NORTH CENTRAL 02**																																		
BIENVILLE 3 NE	23	MAX	87	86	86	81	84	75	77	80	79	82	83	86	86	80	80	67	72	72	66	74	76	70	74	78	64	74	72	77	80	71	73	77.2
		MIN	71	70	71	71	72	54	49	48	48	50	54	67	67	66	65	62	55	49	47	39	45	53	46	53	45	42	55	57	62	63	59	56.6
CALHOUN RSCH STN	08	MAX		89	90	89	90	88	74	77		80	81	83	85	80	80	81	66	72	72	62	69	74	72	74	78	63	74	72	76	79	70	77.2
		MIN		65	65	69	70	58	46	46		44	51	50	64	63	62	65	60	48	48	37	39	39	44	44	42	38	40	52	54	54	61	52.3
COLUMBIA LOCK	07	MAX		84	88	89	87	88	85	73	74			84	88		82	82	67	71	70	66	71	74	70	72	72	77	73	72	80	81	72	77.5
		MIN		68	68	69	69	61	53	51	51			65	66		64	66	61	52	53	44	44	47	52	50	46	42	41	56	60	57	62	56.2
FARMERVILLE	07	MAX	84	85	85	85	83	85	71	75	73	77	78	81	81	77	77	80	64	70	70	61	66	71	69	69	74	76	67	70	72	78	69	74.9
		MIN	69	69	70	71	71	55	50	51	50	55	57	65	66	63	68	63	58	51	54	43	43	55	48	49	43	43	46	57	59	60	67	57.1
HOMER 1N	07	MAX	82	83	89	84	80	82	72	74	73	76	79	79	80	75	77	82	64	69	71	64	67	69	68	71	75	60	67	70		78	69	74.3
		MIN	68	68	67	67	67	54	45	45	44	44	47	53	62	64	64	62	57	45	46	35	37	44	43	43	43	39	38	53		56	63	52.1
MONROE REGIONAL AP	24	MAX	87	90	88	88	89	74	77	78	82	82	84	85	82	83	81	68	74	73	67	71	74	73	74	79	63	74	73	78	81	73	73	78.0
		MIN	71	67	67	70	69	57	50	51	48	54	53	63	65	61	67	60	51	51	43	39	43	52	48	45	41	40	51	57	56	60	60	55.2
RUSTON LA TECH	08	MAX	82	85	86	84	81	83	72	76	74	77	79	82	83	79	78	79	80	72	71	62	69	74	71	73	77	63	71	73	75	77	70	76.1
		MIN	70	68	69	71	70	55	48	48	47	47	52	54	67	65	65	64	60	50	50	40	41	44	47	47	44	43	42	53	57	58	62	54.8
WINNFIELD 3 N	24	MAX																																M
		MIN																																M
**NORTHEAST 03**																																		
BASTROP	07	MAX	84	86	83	84	84	86		72	73	77	77	80	83	78	79	79	67	69	70	63	65	77	68	68	73	59	69	68	72	78	72	74.8
		MIN	69	69	69	70	69	58	48	48	48	48	57	58	64	63	63	64	58	50	50	40	40	44	46	46	41	40	41	53	59	61	61	54.7
LAKE PROVIDENCE	07	MAX	84	86	87	86	87	88	71	73	76	79	80	83	83	80	83	77	68	72	70	65	69	72	73	71	76	62	72	68	74	77	73	76.3
		MIN	69	70	71	72	71	61	53	53	53	53	53	53	65	64	64	66	59	53	54	46	46	48	50	48	45	43	43	57	60	61	63	57.0

LOUISIANA
201310

DAILY TEMPERATURES (°F)

STATION	OB.TIME	MAX/MIN	01	02	03	04	05	06	07	08	09	10	11	12	13	14	15	16	17	18	19	20	21	22	23	24	25	26	27	28	29	30	31	AVERAGE
RAYVILLE	07	MAX	86	91	91	90	91	91	74	76	76	82	83	86	86	81	83	81	70	73	74	68	72	74	74	72	78	63	75	71	78	83	74	78.9
		MIN	71	72	72	72	72	62	53	51	53	53	62	62	68	65	65	67	61	54	54	46	43	48	51	50	46	43	44	56	61	61	64	58.1
ST JOSEPH 3 N	08	MAX	85	89	90	89	90	90	75	71	77	81	82	85	88	85	87	84	80		73	73	71	76		73				72	79			81.0
		MIN	71	71	71	72	71	63	52	52	50	62	63	67	69	63	65	67	61		51	44	46	50		44		41	39	60	60			58.7
TALLULAH	08	MAX		88	87	91	89	85	76	77	82	79	86	84	86	82	84	83	73				69	75	72	71	77	62	74	68	76	80	74	78.9
		MIN		69	69	68	62	51	51	50	55	58	67	64	62	62	64	67	60				55	55	47	47	42	40	43	58	60	61	63	57.4
TALLULAH VICKSBURG RGN	24	MAX	88	89	88	90	90	77	75	77	81	84	84	87	84	86	83	75	71	73	72	71	75	73	73	79	65	73	68	76	82	76	73	78.6
		MIN	71	68	71	71	66	50	46	48	45	56	55	58	63	61	63	62	48	48	42	35	39	42	40	41	37	35	43	52	54	61	64	52.7
WINNSBORO 2 SE	08	MAX	86	89	91	90	90	89	75	77	78	83	84	86	87	82	86	83	74	72	74	70	73	77	73	75	83	65	78	73	80	83	75	80.0
		MIN	70	69	70	73	70	62	51	51	48	51	58	63	68	65	65	68	61	48	52	37	39	45	45	45	45	38	39	57	58	60	62	55.9
WINNSBORO 5 SSE	07	MAX	86	90	90	90	90	89	70	75	76	81	83	86	86	82	85	84	74	72	73	70	73	75	73	74	81	65	76	76	79	83	75	79.4
		MIN	67	68	68	71	69	60	48	48	40	49	57	63	65	63	63	67	59	47	48	36	39	42	45	44	42	37	41	50	56	59	61	53.9
**WEST CENTRAL 04**																																		
HODGES GARDENS	08	MAX	81	87	86	87	85	87	72	76	76	80	80	84	85	85	80	82	71	72	68	63	70	72	70	75	81	66	76	73	75	79	72	77.3
		MIN	69	69	69	71	70	57	50	51	54	57	56	56	64	65	67	70	60	54	55	47	47	52	53	53	48	48	48	57	58	64	65	58.2
LEESVILLE	08	MAX	83	86	88	87	85	84	74	76	78	81	82	86	88	81	81	86	81	76	68	62	73	71	72	78	83	67	77	76	81	82	74	78.9
		MIN	70	69	68	68	70	60	50	47	46	48	54	61	65	67	68	69	61	47	55	37	47	51	42	44	49	40	45	55	57	63	67	56.1
NATCHITOCHES #2	07	MAX	82	87	86	84	82	87	72	76	73	77	80	84	86	77	77	82	68	72	67	61	69	71	69	74	81	64	73	73	74	78	71	76.0
		MIN	69	70	70	70	70	58	52	52	51	53	55	59	66	66	67	63	60	52	53	42	43	49	47	49	47	42	44	58	60	63	63	56.9
TOLEDO BEND LAKE	08	MAX	83	88	87	89	85	88	74	76	75	79	82	85	87	79	81	84	73	71	71	65	71	74	69	76	82	71	76	75	79	81	74	78.4
		MIN	70	71	71	71	71	58	56	56	54	55	59	61	66	66	68	70	61	51	51	47	44	44	53	52	51	48	47	58	58	64	66	58.6
**CENTRAL 05**																																		
ALEXANDRIA	08	MAX	81	87	88	87	88	87	74	78	78	82	80	85	86	85	83	85	85	74	69	70	72	72	72	72	83	68	76	73	78	80	78	79.2
		MIN	69	71	71	72	72	64	54	54	53	53	59	62	68	68	69	69	62	55	55	46	46	52	50	49	50	47	46	59	64	64	64	59.3
ALEXANDRIA 5 SSE	24	MAX	88	90	87	85	83	73	73	75	78	79	84	84	80	81	81	76	72	67	69	70	70	69	76	83	65	72	71	76	77	74	74	76.8
		MIN	69	67	70	71	68	55	49	50	50	53	53	64	55	69	66	60	46	40	42	32	35	40	36	33	35	30	39	55	50	54	49	51.1
BOYCE 3 WNW	24	MAX	83	85	83	83	85	76	74	73	77	77	82	83	78	79	83	77	72	66	64	69	70	70	73	81	64	74	74	74	78	71	73	75.8
		MIN	69	70	70	73	71	59	54	54	53	57	60	67	67	67	69	62	59	54	52	46	52	57	53	55	49	46	46	64	63	63	62	59.5
BUNKIE	08	MAX	78	85	87	89	89	87	75	76	76	81	80	86	87	87	83	86	84	68	68	69	72	70	68	74	81	66	75	77	79	81	77	78.7
		MIN	71	69	71	72	71	66	52	51	51	54	57	60	68	68	67	67	62	52	52	43	44	51	47	48	48	43	43	54	62	64	65	57.8
EUNICE	08	MAX	83	88	89	89	88	88	77	77	77	82	82	87	89	83	85	87	88	70	73	70	74	70	73	77	82	69	77	74	82	81	81	80.4
		MIN	70	70	70	70	71	64	52	52	52	54	58	63	63	68	67	69	63	53	55	47	48	55	48	49	51	46	47	57	61	64	65	58.8
GRAND COTEAU	17	MAX	85	88	87	87	87	78	77	77	82	82	86	89	85	86	84	86	85	74	72	74	72	74	75	80	80	76	76	81	82	82	77	80.8
		MIN	71	70	70	73	72	66	53	51	52	55	58	66	69	68	65	67	63	53	59	48	54	59	50	49	49	44	51	58	61	62	66	59.7
JENA 4 WSW	08	MAX	81	89	89	87	91	86	75	79	77		82																					M
		MIN	69	68	67	69	69	61	48	48	46		55																					M
JONESVILLE LOCKS	06	MAX	81	81	88	87	89	89	85	75	77	89	81	86	88	88	85	84	84		71	69	70	71	72		82		75	76	77	80	76	80.6
		MIN	69	69	70	71	72	72	55	53	52	51	63	62	68	62	67	67	68	61	53	53	46	46	51	46	51	46	45	45	62	64	64	58.8
LSU DEAN LEE RSCH STN	08	MAX	75	85	85				87		77	80				85	79	84	79					69	71	76	82			74	74	77		M
		MIN	68	68	69				53		50	51				59	64	66	60					41	47	49	50			41	60	60		M
NEW ROADS 5 NE	24	MAX	88	90	84	89	84	75	77	75	80	83	88	90	88	87	85	84	69	73	73	71	72	74	74	79	68	76	74	82	83	79	74	79.6
		MIN	71	71	71	73	73	60	55	56	55	63	61	67	69	68	65	68	60	56	57	50	54	56	50	48	51	45	53	62	62	65	62	60.5

LOUISIANA
201310

DAILY TEMPERATURES (°F)

STATION	OB.TIME	MAX/MIN	01	02	03	04	05	06	07	08	09	10	11	12	13	14	15	16	17	18	19	20	21	22	23	24	25	26	27	28	29	30	31	AVERAGE
**EAST CENTRAL 06**																																		
BATON ROUGE METRO AP	24	MAX	86	86	88	87	85	76	76	78	82	82	86	89	89	86	85	83	75	75	74	74	72	78	76	79	70	77	73	83	82	80	81	80.4
		MIN	71	72	72	73	72	57	54	54	53	62	62	65	70	67	64	68	59	55	56	51	53	56	51	47	49	44	51	61	60	63	63	59.8
BOGALUSA	08	MAX																																M
		MIN																																M
CLINTON 5 SE	08	MAX	83	83	83	85	85	74	86	73	76	78	80	85	86	86	82	82	80	68	71	73	73	69	75	73	77	73	71	73	80	79	76	78.0
		MIN	68	70	70	71	71	61	52	52	51	51	64	65	68	65	61	63	65	52	53	48	48	52	48	44	48	42	49	42	58	61	64	57.3
HAMMOND 5 E	08	MAX	82	83	83	87	88	86	84	76	79	83	82	84	85	88	85	84	81	74	73	71	74	69	78	75	80	68	74	69	79	79	78	79.4
		MIN	68	70	70	71	71	71	54	49	49	54	63	63	66	62	60	64	67	55	56	50	50	51	46	42	46	38	41	48	54	57	63	57.1
LSU BEN-HUR FARM	08	MAX	84	84	85	88	86	85	76	76	78	83	81	87	88	87	86	84	81	74	74	74	75	71	77	76	82	70	76	71	81	81	79	80.0
		MIN	70	71	71	72	73	71	56	55	51	56	61	64	68	68	63	67	66	55	58	51	52	54	49	47	51	45	48	53	60	64	65	59.8
SLIDELL	08	MAX	81	84	84	87	87	86	86	75	77	81	80	84	86	85	84	83	83	80	73	69	73	73	78	73	77	67		74	80	80	80	79.7
		MIN	69	73	71	72	73	73	56	53	55	60	64	68	69	66	65	67	69	61	59	53	54	59	52	47	51	44		54	58	62	69	61.5
SLIDELL AP	24	MAX	84	85	88	89	88	88	77	79	83	82	86	87	88	87	84	84	81	75	71	75	75	80	76	77	70	75	74	81	81	81	82	81.1
		MIN	69	72	71	72	73	64	56	52	53	64	63	66	68	64	63	65	65	60	58	52	55	57	49	44	46	40	46	55	55	61	69	59.6
**SOUTHWEST 07**																																		
CROWLEY 2 NE	08	MAX	82	86	87	89	86	81	74	76	76	81	81	87	87	83	84	85	86	71	72	72	74	74	74	76	81	75	76	72	81	80	81	79.7
		MIN	72	70	70	72	73	65	54	54	58	56	60	65	69	69	67	69	65	55	50	55	55	50	50	50	53	46	50	58	61	63	66	60.3
DE RIDDER	08	MAX	77	86	87	85	83	88	72	75	77	79	82	85	89	82	81	85	82	76	65	63	71	69	69	77	80	68	75	76	79	80	75	78.0
		MIN	70	70	70	71	72	60	52	53	52	54	56	62	66	67	69	69	62	62	57	43	48	56	48	50	51	45	50	55	59	64	67	59.0
HACKBERRY 8 SSW	08	MAX	85	85	86	87	88	88	76	78	78	80	82	85	87	84	85	85	84	70	73	68	72	71	73	79	80	72	75	75	81	80	80	79.7
		MIN	76	75	75	77	76	76	58	58	59	62	66	69	70	71	72	73	66	61	60	54	53	64	55	55	58	54	56	62	64	68	70	64.9
JENNINGS	08	MAX	81	86	88	88	88	88	78	77	78	83	83	87	89	84	85	86	87	71	74	71	74	71	74	78	83	71	77	73	81	82	81	80.5
		MIN	72	71	72	72	73	64	54	52	53	53	58	64	69	70	69	70	65	55	56	49	52	60	52	50	51	47	47	57	61	64	66	60.3
LAKE ARTHUR 10 SW	08	MAX	82	84	88	89	87	81	90	79	83	84	87	90	85	87	87	90	85	76	73	75	75	71	78	80	85	78	74	72	84	81	80	81.9
		MIN	71	71	72	72	72	55	54	54	54	55	66	66	70	70	71	67	67	57	50	50	62	61	52	52	56	51	60	63	63	65	66	61.8
LAKE CHARLES AP	24	MAX	86	88	89	89	89	77	79	79	82	83	86	89	83	84	86	85	72	72	69	73	73	74	79	83	72	77	76	81	82	81	78	80.5
		MIN	73	72	73	74	74	58	55	54	55	58	63	69	69	69	69	67	57	56	52	49	55	53	49	51	52	47	57	59	63	67	60	60.6
LELAND BOWMAN LOCK	08	MAX	85	85	95	89	89	88	82	78	80	86	85	86	86	85	86	85	91	78	77	78	76	72	79	80	82	73	74	73	81	81	81	82.1
		MIN	73	73	76	74	75	75	54	56	57	58	60	67	69	66	69	70	69	59	58	53	53	61	56	55	56	50	50	57	67	67	69	63.0
MOSS BLUFF 2 NNW	24	MAX	86	89	90	90	90	75	78	78	81	83	87	88	81	85	88	85	72	73	65	74	73	71	79	82	71	79	78	81	81	81	78	80.4
		MIN	72	71	71	72	72	57	48	48	49	53	58	70	66	69	67	65	60	53	51	42	51	51	45	46	52	42	52	56	62	66	58	57.9
OBERLIN FIRE TWR	09	MAX	76	84	87	87	87	86	73	76	76	81	81	84	87	82	83	85	83	69	69	65	72	70	68	75	81	68	76	74	80	81	81	78.3
		MIN	70	70	69	71	72	63	52	51	50	50	55	63	68	69	67	66	62	53	56	45	46	53	47	48	50	44	46	56	59	63	63	58.0
ROCKEFELLER WL REFUGE	08	MAX	84	86	86	88	88	86	79	77	77	80	83	84	86	83	85	83	84	75	75	73	73	71	77	78	78	72	75	76	81	80	81	80.1
		MIN	71	72	73	74	73	65	55	55	55	57	61	68	69	69	68	70	66	57	57	51	51	62	53	54	56	49	49	60	63	66	68	61.8
VINTON 5W	08	MAX	80	87		89	89	91		77	78		84	89	91	78	85	88	88	73	71	69	75		73	79	82	76	78	77	82	83	78	81.1
		MIN	70	71		70	72	62		51	52		52	62	68	68	66	66	66	54	56	44	44		48	50	50	45	45	57	58	58	68	58.3
**SOUTH CENTRAL 08**																																		
CARVILLE 2 SW	24	MAX	83	84	85	85	83	77	75	77	81	80	84	87	86	84	85	78	72	71	71	74	70	77	72	80	70	75	71	80	81	79	79	78.6
		MIN	74	71	72	72	75	62	58	55	56	63	64	64	63	69	68	69	62	62	60	60	56	56	51	54	53	46	56	62	64	66	45	61.5
DONALDSONVILLE 4 SW	08	MAX	83	83	84	88	87	84	79	74	76	81	81	87	88	85	84	85	79	74	73	71	73	71	77	75	79	69	75	70	81	80	79	79.2

LOUISIANA
201310

DAILY TEMPERATURES (°F)

STATION	OB.TIME	MAX/MIN	01	02	03	04	05	06	07	08	09	10	11	12	13	14	15	16	17	18	19	20	21	22	23	24	25	26	27	28	29	30	31	AVERAGE
			DAY OF MONTH																															
		MIN	70	70	70	70	71	70	54	55	52	53	61	63	68	66	66	66	66	58	58	52	53	56	50	48	48	46	46	49	59	61	65	59.4
FRANKLIN 3 NW	24	MAX	82	85	86	85	84	79	74	75	80	81	85	87	84	85	82	77	73	74	70	73	70	76	75	80	70	77	71	81	81	81	81	78.8
		MIN	72	71	71	73	74	61	56	54	56	58	63	63	72	68	68	70	63	57	60	53	58	58	52	51	54	48	53	62	62	65	65	61.6
JEANERETTE 5 NW	08	MAX	84	84	86	87	85	84	79	75	77	80	80	86	86	86	84	86	83	74	73	73	73	69	76	76	81	70	78	72	83	83	82	79.8
		MIN	72	72	71	71	72	71	54	53	53	53	56	60	68	68	67	67	67	57	57	52	52	57	51	50	50	47	47	54	61	63	66	60.0
LAFAYETTE	22	MAX	86	86	88	89	88	88	78	78	79	82	85	87	87	86	85	85	75	73	74	74	74	72	72	80	80	71	76	72	82	80	82	80.5
		MIN	74	75	71	71	72	74	56	54	54	55	55	62	68	68	65	68	56	55	55	51	51	59	53	51	54	47	47	52	61	62	64	60.0
LAFAYETTE FCWOS	24	MAX	88	88	89	87	88	80	78	79	82	82	88	88	86	85	86	86	74	76	75	74	71	78	78	82	71	77	73	82	81	81	82	81.1
		MIN	73	73	71	74	74	59	56	54	55	57	61	68	70	69	67	70	59	55	56	51	57	57	52	52	52	47	53	59	63	64	64	61.0
MORGAN CITY	08	MAX	87	82	84	90	89	82	79	75	75	82	83	83	83	84	87	87	82	74	75	71	74	72	77	83	86	72	75	80	86	86	83	80.9
		MIN	69	72	71	71	68	68	60	60	60	60	61	67	70	68	66	69	70	64	61	58	58	62	47	46	45	47	47	53	64	64	64	61.6
NEW IBERIA AP ACADIANA RGNL	24	MAX	88	91	91	90	89	82	79	81	86	86	89	89	89	89	88	86	78	77	77	77	73	79	79	83	72	78	74	85	83	83	84	83.1
		MIN	75	75	73	76	76	60	56	56	57	58	62	69	72	70	69	70	62	61	58	56	59	56	51	50	53	48	52	60	63	66	66	62.4
ST MARTINVILLE 3 SW	08	MAX	82	85	86	87	85	85	78	75	76	81	80	86	86	84	84	85	82	72	71	72	73	69	76	75	81	68	75	72	82	82	80	79.2
		MIN	71	71	70	71	72	71	53	52	52	55	60	63	67	67	66	68	65	56	53	51	52	58	48	49	53	44	51	61	62	64	66	60.1
**SOUTHEAST 09**																																		
BOOTHVILLE ASOS	24	MAX	85	85	86	84	85	84	74	74	79	81	84	83	83	84	83	83	83	75	74	73	76	76	73	76	68	74	77	79	80	81	81	79.5
		MIN	76	74	75	75	76	71	68	64	65	69	74	73	73	75	71	73	73	72	66	67	71	68	66	63	61	64	62	67	68	70	74	69.8
GALLIANO	08	MAX	82	81	82	85	83	84	86	75	77	80	82	83	84	85	83	84	84	81	73	69	70	73	78	75	77			74	79	79	79	79.6
		MIN	73	73	73	72	75	74	59	59	56	56	62	69	69	70	69	69	69	65	64	58	58	65	57	51	51			53	61	66	71	64.4
HOUMA	08	MAX																																M
		MIN																																M
MARRERO 9 SSW	08	MAX	85																	88			74	74	78	75	79			75	80	80	81	M
		MIN	73																	56			55	64	56	51	51			61	61	64	68	M
NEW ORLEANS AP	24	MAX	84	83	90	89	89	86	77	77	82	81	84	88	87	85	86	84	80	73	72	72	74	79	74	80	68	74	73	82	82	81	83	80.6
		MIN	75	75	73	73	75	68	64	63	59	65	68	71	73	71	68	71	66	66	63	60	60	62	61	53	57	51	55	61	61	70	69	65.4
NEW ORLEANS AUDUBON	24	MAX	87	85	90	88	87	88	76	77	76	84	85	90	89	86	87	88	82	76	71	76	74	79	76	82	70	79	80	83	83	82		81.9
		MIN	75	72	76	76	75	67	64	60	61	66	70	72	72	69	69	72	66	64	62	56	61	66	59	53	57	52	58	66	64	70		65.7
NEW ORLEANS LKFRNT AP	24	MAX	84	85	88	88	89	85	77	75	81	82	86	86	86	85	85	85	80	74	73	73	74	77	76	77	70	73	75	82	82	81	84	80.6
		MIN	76	74	77	76	77	70	65	65	70	72	73	74	76	74	73	73	67	67	65	60	66	67	66	61	59	57	57	64	68	71	70	68.7
TERRYTOWN 3S	07	MAX	84	84	85	88	88	87	89	77	78	84	83	86	87	88	86	86	86	82	75	70	74	74	79	76	80	70	76	76	82	81	81	81.4
		MIN	74	73	75	73	76	74	63	58	58	61	66	71	71	70	68	70	72	65	63	55	57	65	59	52	54	51	52	55	62	66	69	64.5
THIBODAUX 4 SE	08	MAX	83	78	84	87	83	84	82	74	76	80	80	83	84	85	83	84	82	79	70	69	72	72	77	75	78	66	74	72	80	79	79	78.5
		MIN	70	70	68	70	72	70	54	53	53	55	59	66	68	66	65	65	66	60	59	52	53	59	52	48	50	44	46	50	58	60	67	59.6

LOUISIANA
201310

DAILY SOIL TEMPERATURES

STATION	DEPTH	TIME	01	02	03	04	05	06	07	08	09	10	11	12	13	14	15	16	17	18	19	20	21	22	23	24	25	26	27	28	29	30	31	AVERAGE
**LOUISIANA**																																		
**NORTHWEST 01**																																		
RED RIVER RSCH STN (IN)																																		
BARE GROUND	4	MAX	80	80	81	81	81	81	80	80	78	78	77	77	76	76	76	77	76	76	76	74	74	73	73	72	71[I]	72	71	70	71	71	70	75.9
	4	MIN	79	79	79	80	80	80	79	76	76	75	75	75	75	75	75	75	75	75	74	73	72	72	71	71	72[I]	71	70	70	69	69	70	74.5
**NORTH CENTRAL 02**																																		
CALHOUN RSCH STN (IN)																																		
SOD	4	MAX	-	81	82	83	83	81	78	75	-	74	74	78	77	78	75	75	74	75	73	70	69	68	70	68	67	68	66	67	68	70	71	73.7
	4	MIN	-	76	74	75	76	75	71	68	-	67	67	69	67	72	69	72	70	71	68	63	62	63	62	61	60	57	60	58	62	64	65	67.0
**NORTHEAST 03**																																		
ST JOSEPH 3 N (IN)																																		
BARE GROUND	2	MAX	82	91	102	95	94	95	83	83	86	88	89	90	92	97	88	84	80	-	76	79	81	77	-	82	-	82	81	72	77	-	-	85.6
	2	MIN	69	74	75	77	77	73	62	61	62	70	71	72	73	70	67	70	68	-	62	58	59	59	-	61	-	56	59	63	64	-	-	66.6
WINNSBORO 2 SE (IN)																																		
BARE GROUND	2	MAX	79	80	81	82	80	80	77	75	73	75	77	77	77	77	79	76	75	72	70	70	68	68	68	68	70	63	67	66	70	71	70	73.6
	2	MIN	74	74	74	76	75	75	66	65	63	63	67	68	70	71	71	72	69	63	63	58	58	58	59	58	58	55	55	60	64	64	66	65.5
WINNSBORO 5 SSE (IN)																																		
BARE GROUND	4	MAX	77	80	82	83	78	86	73	76	77	80	80	77	77	76	79	75	72	69	68	68	72	69	68	71	72	67	68	69	71	72	69	74.2
	4	MIN	73	72	73	74	74	73	64	63	62	62	68	68	71	71	70	71	69	62	62	62	58	59	60	60	60	57	57	58	65	64	67	65.5
**CENTRAL 05**																																		
LSU DEAN LEE RSCH STN (IN)																																		
**EAST CENTRAL 06**																																		
CLINTON 5 SE (IN)																																		
HAMMOND 5 E (IN)																																		
**SOUTHWEST 07**																																		
CROWLEY 2 NE (IN)																																		
JENNINGS (IN)																																		

LOUISIANA
201310

SOILS REFERENCE NOTES

STATION	SOIL TYPE	SOIL COVER	SLOPE	UNITS
RED RIVER RSCH STN	SANDY LOAM	BARE GROUND	00	F
CALHOUN RSCH STN	FINE SANDY LOAM	BARE GROUND	00	F
ST JOSEPH 3 N	SHARKEY CLAY	BARE GROUND	LEVEL	F
WINNSBORO 2 SE	SANDY LOAM	BARE GROUND	0	F
WINNSBORO 5 SSE	SANDY LOAM	BARE GROUND	00	F
LSU DEAN LEE RSCH STN	SANDY	BARE GROUND	00	F
CLINTON 5 SE	FINE SANDY LOAM	BARE GROUND	LEVEL NNW	F
HAMMOND 5 E	SANDY LOAM	BARE GROUND	00	F
CROWLEY 2 NE	SAND	SOD	1 DEG S	F
JENNINGS	SILT CLAY LOAM	SOD	0	F

LOUISIANA
201310

SNOWFALL AND SNOW ON GROUND (INCHES)

DAY OF MONTH

STATION		01	02	03	04	05	06	07	08	09	10	11	12	13	14	15	16	17	18	19	20	21	22	23	24	25	26	27	28	29	30	31
**LOUISIANA NORTHWEST 01**																																
BENTON 5E	SNOWFALL																															
	SN ON GND																															
HOSSTON	SNOWFALL																															
JAMESTOWN	SNOWFALL																															
KEITHVILLE	SNOWFALL																															
	SN ON GND																															
KORAN	SNOWFALL																															
LOGANSPORT	SNOWFALL																															
	SN ON GND																															
MANSFIELD 7 NW	SNOWFALL																															
MINDEN	SNOWFALL																															
	SN ON GND																															
MOORINGSPORT 1 N	SNOWFALL																															
	SN ON GND																															
RED RIVER RSCH STN	SNOWFALL					-																										
SHREVEPORT DWTN	SNOWFALL																															
	SN ON GND																															
SHREVEPORT DWTN AP	SNOWFALL																															
SHREVEPORT AP	SNOWFALL																															
	SN ON GND																															
SHREVEPORT STHRN HILLS	SNOWFALL																															
	SN ON GND																															
SHREVEPORT WFO	SNOWFALL																															
	SN ON GND																															
SPRINGHILL	SNOWFALL																															
VIVIAN	SNOWFALL	-	-	-	-	-	-	-	-	-	-	-	-	-	-	-		-	-	-	-	-	-	-	-	-	-	-	-	-	-	-
	SN ON GND	-	-	-	-	-	-	-	-	-	-	-	-	-	-	-		-	-	-	-	-	-	-	-	-	-	-	-	-	-	-

Snowfall: Includes snow and ice. Values for NWS stations (J index note) are Mid-Mid (LST).
Snow on ground: Includes snow, sleet, ice, and hail. Values for NWS stations (J index note) are observed at 12 UTC (GMT).
Water Equivalent: Given for NWS stations (J index note) only, when snow depth is 2 inches or more, and is measured at 18 UTC (GMT)

LOUISIANA
201310

PAN EVAPORATION AND WIND

STATION		DAY OF MONTH 01	02	03	04	05	06	07	08	09	10	11	12	13	14	15	16	17	18	19	20	21	22	23	24	25	26	27	28	29	30	31	TOTAL OR AVERAGE
LOUISIANA																																	
NORTHWEST 01																																	
RED RIVER RSCH STN	WIND	58	68	59	55	89	-	72	20	17	17	45	69	50	24	49	58	10	19	50	25	44	29	24	27	32	52	60	16	42	81	94	1400E
	EVAP	0.10	0.22	0.19	0.19	0.20	0.20	0.18	0.19	0.15	0.18	0.17	0.18	0.00	0.06	0.10	0.00	0.01	0.19	0.11	0.11	0.15	0.15	0.04	0.15	0.17	0.17	0.13	0.05	0.13	0.11	0.00	3.98
	MAX	-	-	-	-	-	-	-	-	-	-	-	-	-	-	-	-	-	-	-	-	-	-	-	-	-	-	-	-	-	-	-	M
	MIN	-	-	-	-	-	-	-	-	-	-	-	-	-	-	-	-	-	-	-	-	-	-	-	-	-	-	-	-	-	-	-	M
NORTH CENTRAL 02																																	
CALHOUN RSCH STN	WIND	-	-	-	-	-	-	-	-	-	-	-	-	-	-	-	-	-	-	-	-	-	-	-	-	-	-	-	-	-	-	-	M
	EVAP	-	-	-	-	-	-	-	-	-	-	-	-	-	-	-	-	-	-	-	-	-	-	-	-	-	-	-	-	-	-	-	M
	MAX	-	-	-	-	-	-	-	-	-	-	-	-	-	-	-	-	-	-	-	-	-	-	-	-	-	-	-	-	-	-	-	-
	MIN	-	-	-	-	-	-	-	-	-	-	-	-	-	-	-	-	-	-	-	-	-	-	-	-	-	-	-	-	-	-	-	M
NORTHEAST 03																																	
ST JOSEPH 3 N	WIND	47	20	12	15	-	-	-	15	5	7	5	-	-	-	29	27	20	-	-	-	-	3	-	-	-	-	-	-	10	-	-	M
	EVAP	0.05	0.23	0.18	0.15	0.00	0.00	0.00	0.11	0.16	0.09	0.23	0.00	0.00	0.00	0.12	0.00	0.00	-	0.00	0.00	0.00	0.00	-	0.00	-	0.00	0.00	0.00	0.00	-	-	1.57E
	MAX	-	-	-	-	-	-	-	-	-	-	-	-	-	-	-	-	-	-	-	-	-	-	-	-	-	-	-	-	-	-	-	-
	MIN	-	-	-	-	-	-	-	-	-	-	-	-	-	-	-	-	-	-	-	-	-	-	-	-	-	-	-	-	-	-	-	M
WEST CENTRAL 04																																	
TOLEDO BEND LAKE	WIND	95	109	70	68	100	68	66	-	34	33	60	82	56	21	52	70	33	36	-	30	52	54	44	-	-	39	90	22	46	121	173	1979E
	EVAP	0.21	0.16	0.22	0.19	0.29	0.31	0.30	-	0.20	0.18	0.23	0.12	0.19	0.04	0.10	0.18	0.06	0.20	-	0.06	0.19	0.16	0.00	-	-	0.19	0.16	-0.01x	0.12	0.15	0.00	5.02E
	MAX	-	-	-	-	-	-	-	-	-	-	-	-	-	-	-	-	-	-	-	-	-	-	-	-	-	-	-	-	-	-	-	-
	MIN	-	-	-	-	-	-	-	-	-	-	-	-	-	-	-	-	-	-	-	-	-	-	-	-	-	-	-	-	-	-	-	M
SOUTHWEST 07																																	
JENNINGS	WIND	42	41	33	32	46	43	38	27	20	23	35	42	41	21	33	33	39	26	63	42	40	23	29	22	33	49	41	27	24	52	77	1137
	EVAP	0.07	0.13	0.18	0.12	0.12	0.12	0.21	0.22	0.09	0.17	0.17	0.09	0.18	0.08	0.12	0.19	0.16	0.08	0.11	0.13	0.12	0.01	0.07	0.13	0.13	0.18	0.14	0.06	0.08	0.12	0.08	3.86
	MAX	79	88	92	91	81	91	81	79	82	84	85	87	89	87	88	89	89	74	73	73	76	71	68	78	81	75	77	69	81	84	79	79
	MIN	70	70	70	70	71	69	54	54	50	56	59	63	68	68	68	69	67	56	56	51	51	58	57	52	54	47	48	55	62	63	63	60.3

Evaporation: Is measured in hundreths of inches.
Wind: Is measured in miles.
Max and Min: The maximum and minimum tempertures (Fahrenheit) of the water in the evaporation pan.

STATION INDEX

STATION	INDEX NO.	DIVISION	COUNTY	LATITUDE	LONGITUDE	ELEVATION (IN FEET)	OBSERVATION TIME AND TABLES LOCAL STD TIME TEMP	PRECIP	EVAP	SPECIAL SEE (NOTES)
LOUISIANA										
ABBEVILLE	0007	07	VERMILION	29 58	92 7W	10		08		H
ABITA RVR COVINGTON	0012	06	ST. TAMMANY	30 28	90 6W	3		07		H
ABITA SPRING FIRE TWR	0021	06	ST. TAMMANY	30 26	90 3W	30		13		H
ABITA SPRINGS 1 SW	0016	06	ST. TAMMANY	30 28	90 3W	25		07		H
ALEXANDRIA	0098	05	RAPIDES	31 19	92 28W	87	08	08		H
ALEXANDRIA 5 SSE	0103	05	RAPIDES	31 15	92 27W	85	24	24		CH
ANGIE	0238	06	WASHINGTON	30 58	89 49W	130		08		H
ARCADIA	0277	02	BIENVILLE	32 33	92 55W	400		08		H
BAKER	0462	06	EAST BATON ROUGE	30 34	91 10W	70		08		H
BASTROP	0537	03	MOREHOUSE	32 44	91 55W	150	07	07		H
BATON ROUGE CONCORD	0548	06	EAST BATON ROUGE	30 25	91 8W	50		08		H
BATON ROUGE METRO AP R	0549	06	EAST BATON ROUGE	30 32	91 9W	64	24	24		HJ
BATON ROUGE SHERWOOD	0558	06	EAST BATON ROUGE	30 27	91 3W	55		08		H
BAYOU SORREL LOCK	0565	08	IBERVILLE	30 8	91 19W	15		08		H
BEAVER FIRE TWR	0617	05	EVANGELINE	30 48	92 30W	105		13		H
BELL CITY 13 SW	0658	07	CAMERON	29 58	93 5W	4		07		H
BENTON 5E	0718	01	BOSSIER	32 42	93 40W	200	08	08		H
BIENVILLE 3 NE	0800	02	BIENVILLE	32 22	92 57W	307	23	23		H
BOGALUSA	0945	06	WASHINGTON	30 47	89 51W	100	08	08		H
BOOTHVILLE ASOS R	1157	09	PLAQUEMINES	29 20	89 24W	3	24	24		H
BOYCE 3 WNW	1232	05	RAPIDES	31 24	92 43W	110	24	24		H
BUNKIE	1287	05	AVOYELLES	30 58	92 11W	80	08	08		CH
CALHOUN RSCH STN	1411	02	OUACHITA	32 31	92 21W	180	08	08	08	GCH
CARENCRO	1535	08	LAFAYETTE	30 19	92 3W	50		07		H
CARVILLE 2 SW	1565	08	IBERVILLE	30 12	91 8W	25	24	24		H
CLAYTON	1866	05	CONCORDIA	31 43	91 32W	73		07		H
CLINTON 5 SE	1899	06	EAST FELICIANA	30 49	90 58W	200	08	08		GCH
CLINTON FORESTRY HQ	1891	06	EAST FELICIANA	30 51	91 1W	250		13		H
COLUMBIA LOCK	1979	02	CALDWELL	32 10	92 6W	80	07	07		H
CONVENT 2S	2002	09	ST. JAMES	29 60	90 49W	25		08		H
COVINGTON 3 NE	2154	06	ST. TAMMANY	30 31	90 5W	25		07		H
CROWLEY 2 NE	2212	07	ACADIA	30 14	92 21W	25	08	08		G H
DE RIDDER	2367	07	BEAUREGARD	30 51	93 17W	190	08	08		H
DENHAM SPRINGS	2350	06	LIVINGSTON	30 29	90 58W	35		07		H
DONALDSONVILLE 4 SW	2534	08	ASSUMPTION	30 4	91 2W	30	08	08		CH
DRY CREEK 8NW	2641	07	BEAUREGARD	30 44	93 8W	95		07		H
DUTCHTOWN #2	2688	09	ASCENSION	30 15	90 59W	18		07		H
EUNICE	2981	05	ST. LANDRY	30 29	92 26W	50	08	08		H
FARMERVILLE	3079	02	UNION	32 47	92 24W	180	07	07		H
FRANKLIN 3 NW	3313	08	ST. MARY	29 49	91 33W	12	24	24		H
GALLIANO	3433	09	LAFOURCHE	29 28	90 18W	5	08	08		H
GONZALES	3695	09	ASCENSION	30 12	90 55W	10		07		H
GORUM FIRE TWR	3741	04	NATCHITOCHES	31 26	92 53W	307		13		H
GRAND COTEAU	3800	05	ST. LANDRY	30 25	92 3W	55	17	17		H
GRAND ISLE	3807	09	JEFFERSON	29 14	89 59W	2		07		H
HACKBERRY 8 SSW	3979	07	CAMERON	29 53	93 24W	6	08	08		H
HAMMOND 5 E	4030	06	TANGIPAHOA	30 30	90 23W	35	08	08		GCH
HODGES GARDENS	4288	04	SABINE	31 22	93 23W	420	08	08		H
HOMER 1N	4355	02	CLAIBORNE	32 49	93 4W	215	07	07		H
HOSSTON	4398	01	CADDO	32 27	93 50W	246		08		H
HOUMA	4407	09	TERREBONNE	29 38	90 49W	8	08	08		H
JAMESTOWN	4592	01	BIENVILLE	32 21	93 12W	190		07		H
JEANERETTE 5 NW	4674	08	IBERIA	29 58	91 43W	20	08	08		H
JENA 4 WSW	4696	05	LA SALLE	31 38	92 12W	210	08	08		CH
JENNINGS	4700	07	JEFFERSON DAVIS	30 12	92 40W	25	08	08	08	GCH
JONESBORO 4 ENE	4732	02	JACKSON	32 15	92 39W	330		13		H
JONESVILLE LOCKS	4739	05	CATAHOULA	31 29	91 52W	70	06	06		CH
KAPLAN	4775	07	VERMILION	29 60	92 17W	15		07		H
KEITHVILLE	4816	01	CADDO	32 21	93 52W	200		07		H
KILLIAN	4878	06	LIVINGSTON	30 22	90 33W	10		08		H
KORAN	4931	01	BOSSIER	32 25	93 28W	175		08		H
LAFAYETTE	5021	08	LAFAYETTE	30 13	92 4W	25	22	22		CH
LAFAYETTE FCWOS R	5026	08	LAFAYETTE	30 12	91 59W	38	24	24		H
LAKE ARTHUR 10 SW	5065	07	CAMERON	30 0	92 47W	10	08	08		H
LAKE CHARLES 2 N	5074	07	CALCASIEU	30 15	93 13W	5		08		H

STATION INDEX

STATION	INDEX NO.	DIVISION	COUNTY	LATITUDE	LONGITUDE	ELEVATION (IN FEET)	OBSERVATION TIME AND TABLES LOCAL STD TIME TEMP	PRECIP	EVAP	SPECIAL SEE (NOTES)
LAKE CHARLES 7 NW	5072	07	CALCASIEU	30 18	93 16W	10		08		H
LAKE CHARLES AP R	5078	07	CALCASIEU	30 7	93 14W	9	24	24		HJ
LAKE CHARLES PORT	5076	07	CALCASIEU	30 13	93 15W	5		08		H
LAKE PROVIDENCE	5090	03	EAST CARROLL	32 48	91 10W	100	07	07		H
LEESVILLE	5266	04	VERNON	31 8	93 14W	28	08	08		H
LEESVILLE 6 SSW	5287	04	VERNON	31 3	93 17W	260		08		CH
LELAND BOWMAN LOCK	5296	07	VERMILION	29 47	92 12W	40	08	08		H
LIVERPOOL 6W	5430	06	ST. HELENA	30 56	90 34W	250		08		H
LIVINGSTON	5438	06	LIVINGSTON	30 31	90 45W	43		06		H
LOGANSPORT	5522	01	DE SOTO	31 58	94 0W	190		07		H
LSU BEN-HUR FARM	5620	06	EAST BATON ROUGE	30 22	91 10W	21	08	08		CH
LSU DEAN LEE RSCH STN	5630	05	RAPIDES	31 11	92 25W	70	08	08		G H
LUTCHER	5783	09	ST. JAMES	30 2	90 42W	20		07		H
MANSFIELD 7 NW	5875	01	DE SOTO	32 8	93 45W	255	08	08		CH
MANY 9 WSW	5896	04	SABINE	31 31	93 37W	286		07		H
MARKSVILLE	5920	05	AVOYELLES	31 8	92 4W	85		08		H
MARRERO 9 SSW	5926	09	JEFFERSON	29 47	90 7W	3	08	08		H
MINDEN	6244	01	WEBSTER	32 36	93 18W	185	07	07		CH
MONROE DELTA CC	6314	02	OUACHITA	32 30	92 2W	70		08		CH
MONROE REGIONAL AP R	6303	02	OUACHITA	32 31	92 2W	79	24	24		H
MOORINGSPORT 1 N	6364	01	CADDO	32 42	93 58W	200	08	08		H
MORGAN CITY	6394	08	ST. MARY	29 41	91 11W	5	08	08		CH
MOSS BLUFF	6431	07	CALCASIEU	30 18	93 12W	19		07		H
MOSS BLUFF 2 NNW	6434	07	CALCASIEU	30 20	93 13W	25	24	24		H
MOUNT HERMON 2W	6466	06	WASHINGTON	30 57	90 18W	320		08		H
NAPOLEONVILLE	6561	08	ASSUMPTION	29 56	91 1W	25		07		H
NATCHITOCHES #2	6584	04	NATCHITOCHES	31 49	93 5W	141	07	07		CH
NEW IBERIA AP ACADIANA RGNL R	6657	08	IBERIA	30 2	91 53W	24	24	24		H
NEW ORLEANS ALGIERS	6666	09	ORLEANS	29 57	90 3W	2		08		H
NEW ORLEANS AP R	6660	09	JEFFERSON	29 60	90 15W	4	24	24		HJ
NEW ORLEANS AUDUBON R	6664	09	ORLEANS	29 55	90 8W	20	24	24		H
NEW ORLEANS LKFRNT AP R	6667	09	ORLEANS	30 3	90 2W	9	24	24		H
NEW ROADS 5 NE	6686	05	POINTE COUPEE	30 44	91 22W	45	24	24		H
NORWOOD	6808	06	EAST FELICIANA	30 58	91 6W	102		08		H
OAK GROVE	6866	03	WEST CARROLL	32 52	91 23W	129		08		H
OAK RIDGE	6868	03	MOREHOUSE	32 37	91 47W	82		07		H
OAKDALE	6836	07	ALLEN	30 49	92 40W	110		07		H
OAKNOLIA 2N	6911	06	EAST FELICIANA	30 45	90 60W	150		07		H
OBERLIN FIRE TWR	6938	07	ALLEN	30 36	92 46W	65	09	09		H
OLD TOWN BAY	6968	07	CALCASIEU	30 17	93 9W	12		07		H
OPELOUSAS	6995	05	ST. LANDRY	30 30	92 6W	56		07		H
PINE GROVE FIRE TWR	7304	06	ST. HELENA	30 43	90 45W	190		13		H
PIONEER 6 W	7312	03	WEST CARROLL	32 45	91 32W	88		08		H
PLAQUEMINE 2 N	7366	08	IBERVILLE	30 19	91 15W	20		07		H
PONCHATOULA 4 SE	7425	06	TANGIPAHOA	30 25	90 23W	18		07		H
PORT ALLEN	7448	05	WEST BATON ROUGE	30 27	91 13W	15		07		H
RAYVILLE	7691	03	RICHLAND	32 30	91 45W	89	07	07		CH
RED RIVER LOCK # 2	7732	05	RAPIDES	31 11	92 17W	75		07		H
RED RIVER LOCK #1	7729	05	CATAHOULA	31 15	91 58W	70		07		H
RED RIVER RSCH STN	7738	01	BOSSIER	32 25	93 38W	155	07	07	07	GCH
ROCKEFELLER WL REFUGE	7932	07	CAMERON	29 44	92 49W	4	08	08		H
RUSTON LA TECH	8067	02	LINCOLN	32 32	92 41W	260	08	08		H
SAILES FIRE TWR	8094	02	BIENVILLE	32 22	93 9W	360		13		H
SHREVEPORT AP R	8440	01	CADDO	32 27	93 49W	254	24	24		HJ
SHREVEPORT DWTN	8436	01	CADDO	32 31	93 45W	180		07		H
SHREVEPORT DWTN AP R	8438	01	CADDO	32 33	93 45W	179	24	24		H
SHREVEPORT STHRN HILLS	8444	01	CADDO	32 24	93 47W	200	07	07		CH
SHREVEPORT WFO	8448	01	CADDO	32 27	93 50W	274	24	24		H
SLIDELL	8539	06	ST. TAMMANY	30 16	89 46W	10	08	08		CH
SLIDELL AP R	8543	06	ST. TAMMANY	30 21	89 49W	27	24	24		H
SPRINGHILL	8683	01	WEBSTER	32 60	93 27W	240		07		H
ST FRANCISVILLE	8136	06	WEST FELICIANA	30 47	91 23W	115		07		H
ST GABRIEL	8139	08	IBERVILLE	30 16	91 6W	30		08		H
ST JOSEPH 3 N	8163	03	TENSAS	31 57	91 14W	78	08	08	07	G H
ST MARTINVILLE 3 SW	8181	08	ST. MARTIN	30 5	91 52W	30	08	08		H
SULPHUR	8831	07	CALCASIEU	30 14	93 21W	10		24		H

STATION INDEX

STATION	INDEX NO.	DIVISION	COUNTY	LATITUDE	LONGITUDE	ELEVATION (IN FEET)	OBSERVATION TIME AND TABLES LOCAL STD TIME TEMP	PRECIP	EVAP	SPECIAL SEE (NOTES)
SUN	8861	06	ST. TAMMANY	30 39	89 55W	75		06		H
TALISHEEK	8906	06	ST. TAMMANY	30 31	89 52W	60		08		H
TALLULAH	8923	03	MADISON	32 24	91 11W	85	08	08		H
TALLULAH VICKSBURG RGN R	8926	03	MADISON	32 21	91 2W	86	24	24		H
TERRYTOWN 3S	8941	09	JEFFERSON	29 55	90 2W	10	07	07		H
THIBODAUX 4 SE	9013	09	LAFOURCHE	29 45	90 46W	15	08	08		CH
TICKFAW 3 ENE	8945	06	TANGIPAHOA	30 36	90 27W	53		24		H
TOLEDO BEND LAKE	9074	04	SABINE	31 12	93 34W	181	08	08	08	H
VILLE PLATTE	9369	05	EVANGELINE	30 42	92 16W	70		07		H
VINTON 5W	9376	07	CALCASIEU	30 12	93 41W	11	08	08		H
VIVIAN	9392	01	CADDO	32 54	93 59W	220		07		H
WEST MONROE	9631	02	OUACHITA	32 28	92 9W	75		07		H
WINNFIELD 3 N R	9803	02	WINN	31 58	92 39W	160	24	24		H
WINNSBORO 2 SE	9804	03	FRANKLIN	32 8	91 43W	74	08	08		G H
WINNSBORO 5 SSE	9806	03	FRANKLIN	32 6	91 42W	80	07	07		GCH
ZWOLLE 2 NW	9980	04	SABINE	31 40	93 40W	209		07		H

REFERENCE NOTES

STATION NAMES: Name of the city, town or locality. Figures and letters following the station names indicate the distance in miles and direction from the post office or town community center.

DIVISIONS: Areas within a state of similar climatological characteristics. Division averages are calculated using data from stations that record temperature and/or precipitation. Station Precipitation totals flagged with an 'F' or 'M' are excluded from the Divisional Average calculations of precipitation. Stations with monthly Temperature averages flagged with an 'F' or 'M' are included in the Divisional Average if there are no more than 9 flagged or missing daily values in the month, else they are excluded from the divisional average for temperature.

NORMALS: The average value of the meteorological element over a time period. Effective 1 January 2012, the averaging period is 1981 to 2010. The normals for National Weather Service localities have been adjusted so as to be representative for the current observation site.

MONTHLY DEGREE DAY TOTALS: One heating (cooling) degree day is accumulated for each whole degree that the daily mean temperature is below (above) 65 degrees Fahrenheit.

PRECIPITATION: Values shown in hundredths of inches are water equivalent totals, i.e., total of liquid and melted frozen precipitation. In the "Monthly Summarized Data" table the total snow and sleet values shown in tenths of inches are unmelted amounts. The max depth on ground values of snow and sleet shown in whole inches are cumulative unmelted amounts. The number of days with .10, .50, 1.00 or more refers to water equivalents.

PRECIPITATION QUALITY CONTROL: The NCDC quality control process may flag precipitation data that are spatially inconsistent, exceed climatological limits, or are inconsistent with prevailing weather patterns.

TEMPERATURE: Original temperature values are given in the "Daily Temperature" table. Summary temperature information (averages, departures, extremes, monthly degree day totals) is based on the values labeled MAX/MIN.

WIND: (As shown in the "Evaporation and Wind" table) the total wind movement in miles over the evaporation pan as determined by an anemometer recorder located 6-8 inches above the pan.

SYMBOLS AND LETTERS USED IN THE STATION INDEX TABLE

C Station is equipped with recording rain gage (R) but values in this bulletin are from a non-recording rain gage unless indicated by an R.
G Observations appear in the "Soil Temperatures" table.
H Observations appear in the "Snowfall and Snow on the Ground" table.
J Station also published as a Local Climatological Data publication.
VAR Observation time varies.
SR Observation time near sunrise.
SS Observation time near sunset.

SYMBOLS AND LETTERS USED IN THE DATA TABLES

(DAILY DATA ARE FOR THE 24 HOURS IMMEDIATELY PRECEDING OBSERVATION TIME.)
BLANK Entries in the "Monthly Summarized Data" table indicate no record.
BLANK Entries in the "Daily Precipitation" and "Snowfall and Snow on the Ground" tables indicate zero.
BLANK Entries in the "Daily Temperature" table indicate a missing record

- No record. Data not recorded or not received in time for publication.
+ Precipitation or temperature extremes occurred on one or more previous dates during the month.
* Rain gage not read. Precipitation is included in the amount following the asterisks. Time distribution may not be known. A * preceding the monthly total indicates precipitation amount is being carried forward to next month's total, and may include amounts from the previous month(s).
a As a subscript, indicates accumulated total.

A Amount of precipitation is the total of observer's entries for the current month. It may include precipitation that occurred during the previous month. Refer to earlier bulletin to determine date of last reading. (Hawaii stations)
B Divisional Departure from normals are computed using 1971-2000 normals.
E Normalized HDD/CDD Calculation. E is appended to the HDD/CDD Calculation when 1-9 individual daily TMAX and/or TMIN values are missing and a Normalized HDD/CDD Calculation is provided. M appears alone if 10 or more daily values are missing.
F Monthly calculation flagged value. F is appended to average and/or total values computed which exclude one or more daily data values that have been flagged by the GHCN-Daily Dataset
M Insufficient or partial data. M is appended to average and/or total values computed with 1-9 daily values missing. M appears alone if 10 or more daily values are missing, (8 or more for wind and evaporation).
N Indicates snow fall or Snowdepth totals are computed with one or more missing days.
R Amounts from recording rain gage.
T Trace. An amount too small to measure.

SEASONAL TABLES: Monthly and seasonal snowfall and heating degree days for the 12 months ending with the June data are published in the July issue of this bulletin. Cooling degree days for the calendar year are published in the "Climatological Data Annual Summary."

Information concerning the history of changes in locations, exposure, etc. of substations is kept on file at the National Climatic Data Center. Historical information of regular National Weather Service Offices may be obtained from the "Local Climatological Data" annual publication. The contents of this publication may be reprinted or otherwise used freely, with proper credit to the National Climatic Data Center. The data are also available digitally.

Effective with the January 2011 Data-Month, COOP Observer Names are no longer included in the Monthly and Annual Climatological Data Publications. This information is not published to ensure the privacy of personal information pursuant to Section 208 of the E-Government Act of 2002 (44 USC 3601).

As of the 2011 Data-Year, Station and Climate Division Maps are no longer being included in the CD Publications. NCDC's Product Development Branch provides updated Station Maps for various data networks via the Historical Observing Metadata Repository: http://www.ncdc.noaa.gov/homr.

The GHCN-Daily Quality Control Flags shown below are displayed as superscripts with the data. For more Information on Global Historical Climatology Network - Daily and flags, see:
http://www.ncdc.noaa.gov/oa/climate/ghcn-daily/
and
Comprehensive Automated Quality Assurance of Daily Surface Observations.
Durre, Imke, Matthew J. Menne, Byron E. Gleason, Tamara G. Houston,
Russell S. Vose, 2010: J. Appl. Meteor. Climatol., 49, 16151633.
doi: 10.1175/2010JAMC2375.1

Blank = Passed All checks	N = failed naught check
D = failed duplicate check	O = failed climatological outlier check
G = failed gap check	R = failed lagged range check
I = failed internal consistency check	S = failed spatial consistency check
K = failed streak/frequent-value check	T = failed temporal consistency check
L = failed check on length of multiday period	W = temperature too warm for snow
M = failed megaconsistency check	X = failed bounds check
	Z = flagged as a result of an official Datzilla investigation

Beginning with the January 2013 CD Publication, monthly mean temperature calculations have changed to the National Data Stewardship Team standard. Monthly maximum and minimum temperatures are not rounded until after the monthly mean temperature is calculated. This is the most accurate outcome, but may be slightly different from the mean derived from rounded monthly maximum and minimum.

Processing Updates and Errata: The 2011 CD Publications were reproduced in May 2013. This update included the addition of late reports and corrections based on additional investigations of reported data issues through NCDCs Datzilla system. In addition, divisional averages for precipitation were recalculated using the method described in DIVISIONS above. Previous editions of the 2011 Publications included all precipitation stations regardless of missing data in the calculation of divisional averages. HDD/CDD values were recalculated to match the legacy method of calculation (truncation of monthly HDD/CDD values instead of rounding).

These and other publications are available from the National Climatic Data Center

Hourly Precipitation Data
This publication contains hourly precipitation amounts obtained from recording rain gages located at National Weather Service, Federal Aviation Administration, and cooperative observer stations. Published data are displayed in inches and tenths or inches and hundredths at local standard time. HPD includes maximum precipitation for nine (9) time periods from 15 minutes to 24 hours, for selected stations.

Climatological Data
Monthly editions contain station daily maximum and minimum temperatures and precipitation. Some Stations provide daily snowfall, snow depth, evaporation, and soil temperature data. Each edition also contains monthly summaries for heating and cooling degree days (65 degree F base). The July issue contains a recap of monthly heating degree days and snow data for the preceding July through June.

The Annual issue contains monthly and annual averages of temperature, precipitation, temperature extremes, freeze data, soil temperatures, evaporation, and a recap of monthly cooling degree days.

Storm Data
Monthly issues contain a chronological listing, by states, of occurrences of storms and unusual weather phenomena. Reports contain information on storm paths, deaths, injuries, and property damage. An "Outstanding storms of the month" section highlights severe weather events with photographs, illustrations, and narratives. The December issue includes annual tornado, lightning, flash flood, and tropical cyclone summaries.

Monthly Climatic Data for the World
This publication contains monthly means for temperature, pressure, precipitation, vapor pressure, and sunshine for approximately 2,000 surface data collection stations worldwide and monthly mean upper air temperatures, dew point depressions, and wind velocities for approximately 500 observing sites.

Local Climatological Data
LCD publications summmarize temperature, relative humidity, precipitation, cloudiness, wind speed and direction observations for several hundred cities in the U.S. and its territories. Each monthly publication also contains 3 hourly weather observations for that month and a hourly summary of precipitation. Annual LCD publications contain a summary of the past calendar year as well as historical averages and extremes.

For Information Call:
(828) 271-4800 Option 2
(828) 271-4010 (TDD)
(828) 271-4876 (Fax)

NOAA\National Climatic Data Center
Attn: User Engagement & Services Branch
151 Patton Avenue
Asheville, NC 28801-5001

Customer Services Number: (828) 271-4800, option 2
TDD : (828) 271-4010
Fax number: (828) 271-4876

NCDC now offers free online access to the ***Climatological Data*** **publication.**
Go to : **www.ncdc.noaa.gov** and choose Most Popular.
